# Synthesis and Study of the Structure–Activity Relationship of Antiproliferative *N*-Substituted Isosteviol-Based 1,3-Aminoalcohols

**DOI:** 10.3390/ph17020262

**Published:** 2024-02-19

**Authors:** Dániel Ozsvár, Noémi Bózsity, István Zupkó, Zsolt Szakonyi

**Affiliations:** 1 Interdisciplinary Excellence Center, Institute of Pharmaceutical Chemistry, University of Szeged, Eötvös utca 6, H-6720 Szeged, Hungary; ozsmozs88@gmail.com; 2 Institute of Pharmacodynamics and Biopharmacy, University of Szeged, Eötvös utca 6, H-6720 Szeged, Hungary; bozsity-farago.noemi@szte.hu (N.B.); zupko.istvan@szte.hu (I.Z.); 3 Interdisciplinary Centre of Natural Products, University of Szeged, Eötvös utca 6, H-6720 Szeged, Hungary

**Keywords:** isosteviol, diterpene, chiral, aminoalcohol, antiproliferative, SAR study

## Abstract

Starting from isosteviol, a series of diterpenoid 1,3-aminoalcohol derivatives were prepared via stereoselective transformations. The acid-catalysed hydrolysis and rearrangement of natural stevioside produced isosteviol, which was transformed into the key intermediate methyl ester. In the next step, an 1,3-aminoalcohol library was prepared by the reductive amination of the intermediate 3-hydroxyaldehyde obtained from isosteviol in a two-step synthesis. To study the effect of the carboxylate ester function at position 4, the free carboxylic acid, benzyl ester and acryloyl ester analogues were prepared as elongated derivatives in comparison with our earlier results in this field. The antiproliferative activity of compounds against human tumour cell lines (A2780, HeLa, MCF-7 and MDA-MB-231) was investigated. In our preliminary study, the 1,3-aminoalcohol function with *N*-benzyl or (1*H*-imidazol-1-yl)-propyl substitution and benzyl ester moiety seemed essential for the reliable antiproliferative activity. The results obtained could be a good starting point to further functionalisation towards more efficient antiproliferative diterpenes.

## 1. Introduction

Nowadays, particular attention has been paid toward the glycosides of the plant *Stevia rebaudiana*, not only because they are much sweeter than sucrose and applied as a zero-calory artificial sweetener [1], but because they have a wide range of biological activities, including antibacterial, antiviral, and anticancer properties [2,3]. In addition to the glycosides, isosteviol, a derivative of stevioside aglycone steviol, has also been found to exhibit several biological activities, such as hypoglycemic, anti-inflammatory, antihypertensive, and anticancer [4,5]. Hence, to access new derivatives with remarkable biological activities, they are often structurally modified, and the resulting derivatives exhibit antiproliferative [6], antiviral [7], antimitotic [8], anticarcinogenic [9], cardioprotective [10], acetylcholinesterase inhibitor [11], antibacterial [12], and antituberculotic [13] activities.

The increasing number of cancer cases is a severe health problem worldwide and it is expected to reach 24 million by 2035 [14]. Despite modern therapies, current cancer treatment has several limitations, including side effects and the high costs of anticancer agents [15,16,17]. Therefore, developing new compounds, such as cheaper anticancer agents with higher bioactivities and weaker side effects, is imperative. Natural products and their modifications have been vital as anticancer agents, and metabolites of diterpenoids have increasingly become a significant part of anticancer drug research in the past decade [18,19]. Recently, microbial transformations and chemical modifications have been used to synthesise cytotoxic isosteviol derivatives, which have garnered significant attention [20,21,22]. In particular, hundreds of isosteviol derivatives are synthesised by chemical modifications on their reactive groups, and some of the compounds exhibit good cytotoxic activity and can become potential drug candidates [23]. Isosteviol derivatives containing the cyclopentanone ring with an *exo*-methylene bridge were synthesised by Zhang et al. [24], and their antiproliferative activities were evaluated against human gastric carcinoma MGC-803, HepG-2, and breast carcinoma MDA-MB-231 human cell lines.

The 1,3-oxoallyl derivative was certified as one of the most potent and most selective anticancer compounds in this group, which was more effective than Adriamycin (IC_50_: 2.53, 2.08, and 2.26 μM) with an IC_50_ value of 1.58 μM. At C-19 of the diterpenoid skeleton of isosteviol, some structural modifications were carried out by Khaybullin et al. They synthesised 15 hybrids of nitric oxide donor functionality and an isosteviol core, resulting in several compounds with antiproliferative properties [4]. The C-19 conjugated derivative was found to be the most potent against B16-F10 melanoma cells with an IC_50_ of 0.02 μM.

A series of 1,2- and 1,3-aminoalcohol derivatives of isosteviol were synthesised by Zhang et al., and they investigated their in vitro antitumour activities [25]. Their results demonstrated that both the hydroxyl and the amino functionalities proved to be advantageous considering the anticancer activities of the desired isosteviol derivatives. When the antiproliferative activities of diastereoisomeric and regioisomeric 1,3-aminoalcohols were compared, it was observed that there was no significant difference in the cytotoxicity between the epimers and regioisomers (IC_50_ values were observed between 2.47 µM and 12.25 µM against HCT-116, EC9706, and Eca109 cells), but any modification of the amino alcohol moiety resulted in decreased activity [23,25]. Similarly, the reaction of isosteviol-based 1,3-aminoalcohols with isothiocyanates resulted in thiourea derivatives with remarkable antiproliferative activity, whereas the *p*-nitrophenyl-substituted derivative showed the strongest cytotoxic activity with IC_50_ values of 1.45 µM, 2.35 µM, and 3.54 µM against HCT-116, HGC-27, and JEKO-1 cells, respectively [9].

In our recent work, we reported the synthesis of a series of isosteviol-based 1,3-aminoalcohols with the advantage of an *N*-benzyl substitution of the amino function on the antiproliferative activity of the prepared compounds [26]. In our present study, our aim is to extend the structure–activity examination of the effect of the *N*-substitution, as well as the changing in the ester function at position 4 either to a free carboxylic acid function or other ester groups, such as a benzyl functionality. We plan to extend the antiproliferative activity by combining the aminoalcohol function with other bioactive functions, like acroyl moieties at position 4 of the *ent*-caurane moiety.

## 2. Results and Discussion

### 2.1. Synthesis of Isosteviol-Based 1,3-Aminoalcohols

In our previous study, the structure–activity study revealed that the isosteviol methyl ester with a 1,3-aminoalcohol group and an *N*-(4-fluorobenzyl) moiety were the most effective against cancer cell lines. Consequently, this compound was chosen for further modifications [26]. Two possibilities were considered: building a number of new moieties on the NH group or forming an alternative functional group on the carboxyl function at position C-19. The second substitution of the amino function was excluded as the NH group was found in our previous study to be essential for cytotoxic activity. Therefore, only an additional modification of the benzylamino group seemed to be promising. In the literature, the carboxyl group of isosteviol was not found to play any role in the cytotoxic activity, making it a good starting point.

**Scheme 1:**
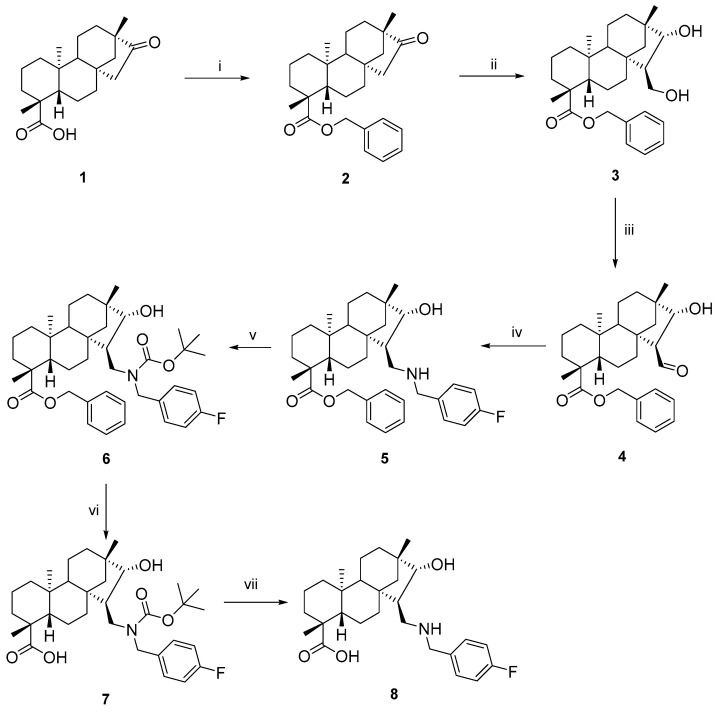
Synthesis of isosteviol-based *N*-(4-fluorobenzyl)-1,3-aminoalcohol **8**. (i) BnBr, K_2_CO_3_, dry acetone, 4 h, 60 °C, 87%; (ii) HCHO, NaOEt, dry EtOH, 1 h, 60 °C, 78%; (iii) 10 mol% TEMPO, NBS, TBAB, DCM/H_2_O, 12 h, reflux, 83%; (iv) (1) 4-FBnNH_2_, dry EtOH, 3 h, 25 °C; (2) MeOH, NaBH_4_, 4 h, 25 °C, 70%; (v) Boc_2_O, dry DCM, 1 h, 25 °C, 79%; (vi) EtOAc/*n*-hexane, 5% Pd/C, H_2_ (1 atm), 24 h, 25 °C, 82%; (vii) (1) TFA, dry DCM, 3 h, 25 °C; (2) Et_3_N, dry DCM, 3 min, 25 °C, 77%.

We desired to prepare a derivative containing a free carboxyl group by the removal of the methyl ester group. However, in our experiments, this proved to be unsuccessful, and this led to a change in the entire synthesis route. The use of a free carboxyl group in the synthesis was not feasible due to cross-reactions during the building of amino functionality, and the resulting products were not purifiable. The benzyl ester functional group was chosen since it could be easily removed by a debenzylation reaction, and the purification of the resulting intermediates seemed to be easier by column chromatography. The desired key intermediate **7** was produced in six steps with an acceptable yield (Scheme 1).

Isosteviol **1** was obtained by acid-catalysed hydrolysis and the Wagner–Meerwein rearrangement of a natural mixture of stevioside according to a literature process [9]. The benzyl ester derivative of isosteviol **2** was prepared with benzyl bromide in a good yield.

Benzyl ester diol **3** was prepared with a good yield using a one-pot aldol-Cannizzaro process, a well-known stereoselective method according to the literature [27]. Compound **4** was prepared in a regioselective manner in an excellent yield by the TBAB-catalysed oxidation of **3** with TEMPO and NBS. The synthesis of *N*-(4-fluorobenzyl)-1,3-aminoalcohol benzyl ester **5** was carried out by a two-step synthesis. First, the reductive amination of hydroxyaldehyde **4** with 4-fluorobenzylamine formed a Schiff base, followed by its reduction with NaBH_4_ under mild conditions.

The synthesis of **8** required a debenzylation reaction, but it also removed the *N*-benzyl unit. To avoid this unwanted reaction, the nitrogen was protected with the Boc group. Boc-protected **7** with a free carboxyl function was obtained in an excellent yield during the debenzylation of **6** by hydrogenolysis over Pd/C in a 1:1 mixture of EtOAc/*n*-hexane without the elimination of the 4-fluorobenzyl function.

The removal of the *N*-Boc-protecting group under acidic conditions was accomplished by TFA treatment, resulting in the corresponding salt of **8**, which was dissolved in DCM, neutralised with TEA to produce free-base **8** in a pure form (Scheme 1).

### 2.2. (Acryloyloxy)butyl and Propargyl Ester Derivatives of N-Substituted 1,3-Aminoalcohols

**Scheme 2:**
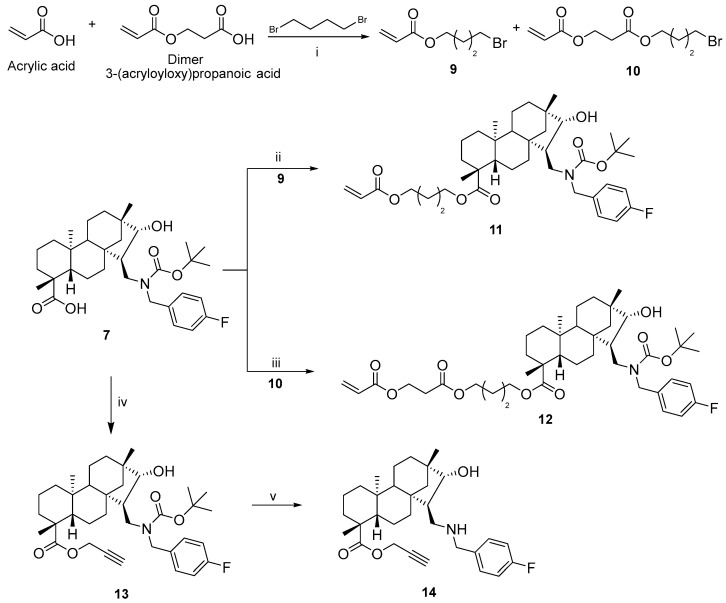
Synthesis of allyl (**11, 12**) and acetylene derivatives (**13, 14**). (i) Acrylic acid, K_2_CO_3_, 1,4-dibromobutane, dry acetone, 24 h, 25 °C, 61% (**9**), 26% (**10**); (ii) 4-bromobutyl acrylate, K_2_CO_3_, dry acetone, 24 h, 25 °C, 83%; (iii) 3-(4-bromobutoxy)-3-oxopropyl acrylate, K_2_CO_3_, dry acetone, 24 h, 25 °C, 77%; (iv) propargyl bromide, K_2_CO_3_, dry acetone, 24 h, 25 °C, 88%; (v) (1) TFA, dry DCM, 3 h, 25 °C; (2) Et_3_N, dry DCM, 3 min, 25 °C, 79%.

In the next step of the synthesis, a linker containing an acrylic acid unit was prepared through a simple esterification reaction between acrylic acid and 1,4-dibromobutane. The excess use of 1,4-dibromobutane was necessary to suppress diester formation. However, because of the fast dimerisation of acrylic acid and the elongated reaction time, both an esterified monomer and dimer products were formed and isolated in the esterification reaction (Scheme 2). This provided an opportunity to investigate the contribution of the carbon chain length to the development of cytotoxic effects.

During the synthetic process, acrylic acid esters containing one or two acrylic units were coupled to product **7** via a simple ester formation, resulting in **11** and **12**. *N*-Boc protection was necessary to avoid the *N*-alkylation reaction. Unfortunately, the removal of the Boc groups could not be carried out due to the formation of many side reactions. It was surmised that these products were formed in inter- or intramolecular aza-Michael reaction between the free NH group and the acryloyloxy function (Scheme 2).

Finally, compound **14** with an alkyne function on the C-19 was prepared by reacting **7** with propargyl bromide, followed by removing the Boc-protecting group (Scheme 2).

### 2.3. Isosteviol-Based 1,3-Aminoalcohols with Diverse O and N Functions

**Scheme 3:**
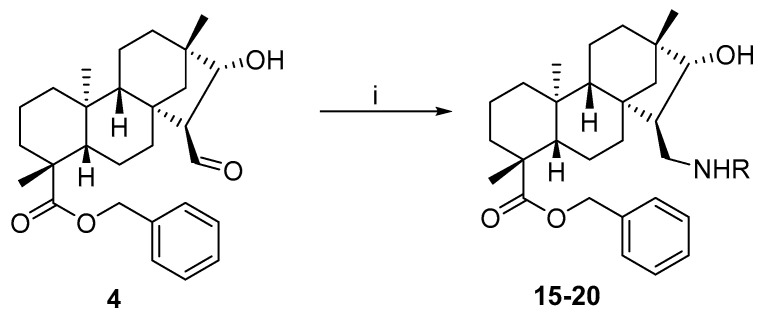
Synthesis of isosteviol-based 1,3-aminoalcohols. (i) (1) RNH_2_ (1 equ.), dry EtOH, 3 h, 25 °C; (2) MeOH, NaBH_4_ (2 equ.), 4 h, 25 °C, 21–68%.

The isosteviol-based 1,3-aminoalcohols were found to be the most effective products against cancer cell lines among those synthesised to date. Thus, it was decided to expand the library of 1,3-aminoalcohols by reacting hydroxyaldehyde benzyl ester **4** with six different primary amines. In the method for synthesising **15**–**20**, the results of the *N*-substituted isosteviol-based 1,3-aminoalcohol experiment were utilised. The syntheses were performed in two parts. First, hydroxyaldehyde **4** was subjected to reductive amination with primary amines to form Schiff bases. The second step involved a reduction with NaBH_4_ under mild conditions. The desired *N*-substituted 1,3-aminoalcohols **15**–**20** were obtained in moderate yields. Crystallisation was employed to purify each product, resulting in a 98% purity of the aminoalcohols with low product recovery. Scheme 3 and Table 1 show the synthesised products and the corresponding yields.

**Scheme 4:**
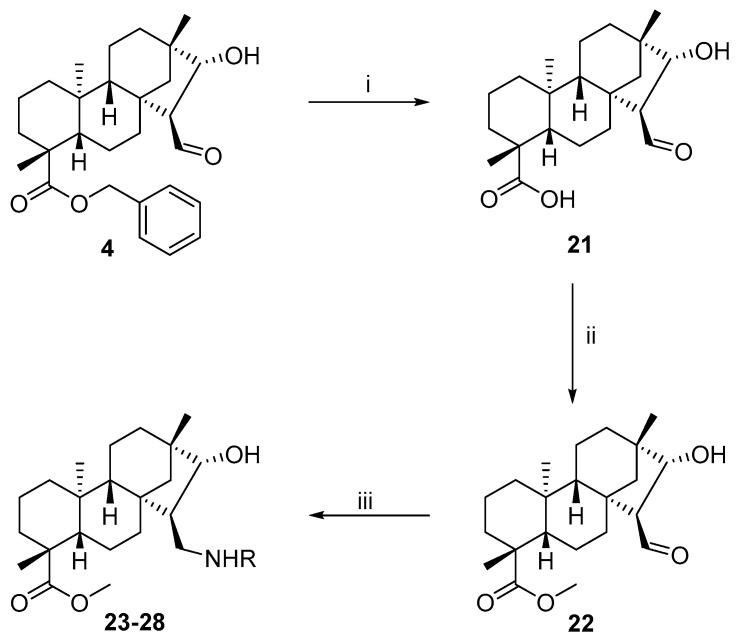
Synthesis of the library of **23**–**28**. (i) EtOAc/*n*-hexane 1:1, 5% Pd/C, H_2_ (1 atm), 24 h, 25 °C, 63%; (ii) CH_2_N_2_, Et_2_O, 5 min, 25 °C, 81%; (iii) (1) RNH_2_ (1 equ.), dry EtOH, 3 h, 25 °C; (2) MeOH, NaBH_4_ (2 equ.), 4 h, 25 °C, 38–88%.

To determine the extent to which the development of the cytotoxic effect was contributed to by the various NH-linked units, a 6-membered 1,3-aminoalcohol library **23**–**28** was prepared, whereas benzyl ester **4** served as a key intermediate in the first step. Acid **21** was prepared from **4** by the debenzylation over 5% Pd/C in the 1:1 mixture of *n*-hexane/EtOAc, followed by re-esterification with diazomethane to obtain **22** (Scheme 4).

The 1,3-aminoalcohol library **23**–**28** was prepared by reacting hydroxyaldehyde **22** with 6 primary amines, followed by a reduction of the resulting Schiff bases (Scheme 4 and Table 2).

### 2.4. In Vitro Antiproliferative Studies of Steviol-Based Aminoalcohols and Structure–Activity Relationship

**Figure 1:**
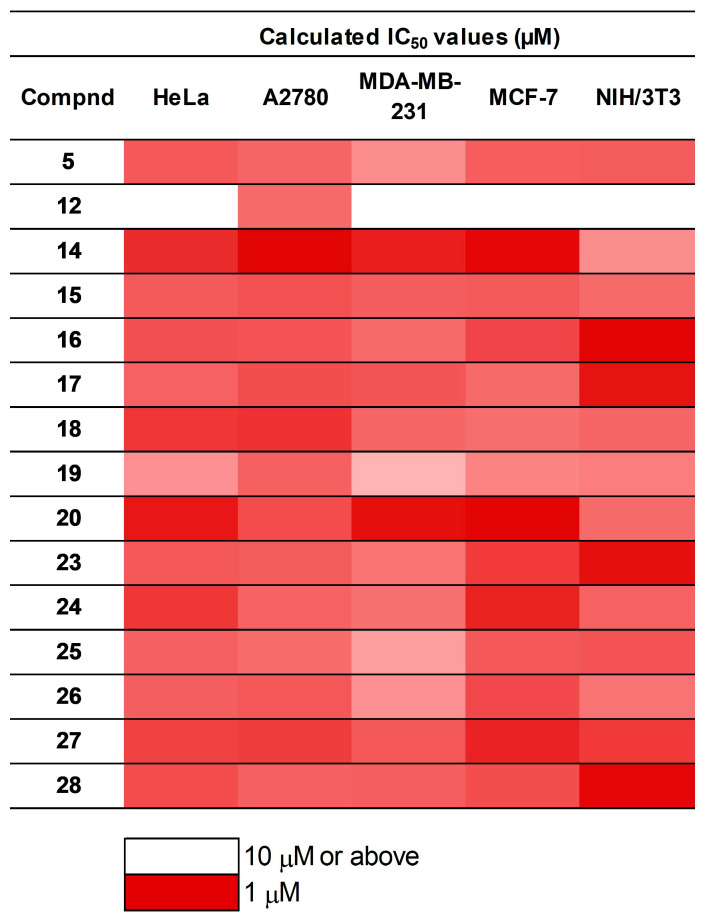
Antiproliferative properties showing IC_50_ values of the prepared isosteviol analogues against cancer cells and NIH/3T3 fibroblasts. The numeric values are given in Table S1 in the Supplementary Materials.

The antiproliferative activities of the prepared diterpene analogues were studied using an MTT assay on a panel of human adherent cancer lines, including cells from cervical (HeLa), breast (MDA-MB-231, MCF-7), and ovary cancers (A2780), as given in Figure 1 and Table S1 in the Supplementary Materials. A non-malignant fibroblast cell line (NIH/3T3) was also utilised to obtain preliminary information on the cancer selectivity of the tested compounds. Based on the obtained activities, some conclusions could be drawn regarding the structure–activity relationships (SARs). Since neither the key intermediate benzyl ester of hydroxylaldehyde **4** nor the *N*-Boc protected aminoalcohols (**6**, **7**, **11**, **12**) or even compound **8** with a free carboxylic function exerted remarkable antiproliferative effects (except for **12** against A2780 ovarian cancer cells), we could conclude that the simultaneous presence of the ester function and the basic secondary amino function were essential for the inhibition of cell growth. No consistent and substantial difference was recognised comparing the activity of the benzyl esters of the secondary amines (**15**–**20**) and their methyl analogues (**23**–**28**), indicating that the alcohol had no crucial impact on the antiproliferative properties. All active molecules inhibited considerably the proliferation of non-malignant cells, which meant that most currently presented agents were not optimal for further investigations of their anticancer properties.

Changing the methyl ester function of our most active compound produced during our previous work (methyl ester and *N*-4-fluorobenzyl-substituted aminoalcohol, (best IC_50_ value: 2.14 µM for MCF-7 cells) [26], and propargyl (**14**, best IC_50_: 1.54 µM for MCF-7 cells)) increased the antiproliferative activity, while the benzyl ester presented similar results. This was probably due to their π–π overlapping properties. Note, however, that in the case of the free carboxylic function, the antiproliferative activity was lost.

The further change in the 4-fluorobenzyl substituent into other aromatic systems did not increase the activity. Surprisingly, compound **20** substituted with the *N*-(1*H*-imidazol-1-yl)propyl group was proven to be the most active derivative (best IC_50_ value: 1.37 µM for MCF-7 cells), despite our previous observation that an *N*-alkyl substitution reduced the antiproliferative activity [26,28]. Since this latter molecule seems to be superior to the clinically utilised cisplatin, it can be regarded as a potential hit compound and it can be investigated further.

## 3. Materials and Methods

### 3.1. Chemistry

General methods: commercially available reagents were used as obtained from the suppliers (Molar Chemicals Ltd., Halásztelek, Hungary; Merck Ltd., Budapest, Hungary and VWR International Ltd., Debrecen, Hungary), while solvents were dried according to standard procedures. Optical rotations were measured in MeOH at 20 °C with a Perkin-Elmer 341 polarimeter (PerkinElmer Inc., Shelton, CT, USA). Chromatographic separations and monitoring of the reactions were carried out on Merck Kieselgel 60 (Merck Ltd., Budapest, Hungary). Melting points were determined on a Kofler apparatus (Nagema, Dresden, Germany). ^1^H- and ^13^C-NMR spectra were recorded on a Brucker Avance DRX 500 spectrometer (500 MHz (^1^H) and 125 MHz (^13^C), δ = 0 (TMS)). Chemical shifts were expressed in ppm (δ) relative to the TMS as an internal reference. *J* values were presented as Hz. All ^1^H/^13^C NMR, NOESY, 2D-HMBC, and 2D-HSQC spectra are available in the Supplementary Materials. An HRMS flow injection analysis was performed with a Thermo Scientific Q Exactive Plus hybrid quadrupole-Orbitrap (Thermo Fisher Scientific, Waltham, MA, USA) mass spectrometer coupled to a Waters Acquity I-Class UPLC™ (Waters, Manchester, UK).

Starting materials: stevioside was obtained from Molar Chemicals Ltd., Halásztelek, Hungary. Isosteviol **1** was prepared from commercially available stevioside or a mixture of steviol glycosides in a one-step synthesis according to the literature, and all spectroscopic data were the same as described in the literature [29].

Compounds **2** [30], **3** [31], **9** [32], **21** [33], and **22** [26] were prepared by the methods presented in the literature. Their spectroscopic data and physical and chemical properties were similar to those reported in the literature.

#### 3.1.1. (4*R*,4a*S*,6a*S*,7*S*,8*R*,9*S*,11b*S*)-Benzyl 7-formyl-8-hydroxy-4,9,11b-trimethyltetradecahydro-6a,9-methanocyclohepta[a]naphthalene-4-carboxylate (**4**)

To a solution of **3** (6.81 mmol, 3.00 g) in 1/1 DCM/H_2_O (300 mL), TEMPO (10 mol%, 106 mg), NBS (13.62 mmol, 2.42 g), and TBAB (6.81 mmol, 2.20 g) were added. After a 12 h reflux, the reaction was found to be completed (indicated by TLC), and the mixture was extracted with DCM (3 × 100 mL). The combined organic phase was extracted with water (1 × 100 mL), dried (Na_2_SO_4_), filtered, and concentrated. The purification of the crude product was accomplished by column chromatography on silica gel with an appropriate solvent mixture (*n*-hexane/EtOAc = 3:1). Yield: 2.48 g (83%); colourless oil; [*α*]_D_^20^ = −69 (*c* 0.71 MeOH); ^1^H-NMR (500 MHz, CDCl_3_) δ (ppm): 0.79 (s, 3H), 0.86–0.91 (m, 1H), 0.95 (s, 3H), 0.99–1.04 (m, 3H), 1.12–1.15 (m, 1H), 1.19 (s, 3H), 1.22–1.23 (m, 1H), 1.33–1.36 (m, 1H), 1.41–1.45 (m, 1H), 1.50–1.4 (m, 1H), 1.64–1.85 (m, 8H), 2.22 (d, 1H, *J* = 13.2 Hz), 2.85–2.86 (m, 1H), 4.27 (d, 1H, *J* = 4.9 Hz), 5.03 (d, 1H, *J* = 12.2 Hz), 5.14 (d, 1H, *J* = 12.2 Hz), 7.31–7.39 (m, 5H), 9.77 (d, 1H, *J* = 2.2 Hz); ^13^C-NMR (125 MHz, CDCl_3_) δ (ppm) 13.1 (CH_3_), 18.8 (CH_2_), 19.7 (CH_2_), 21.8 (CH_2_), 24.5 (CH_3_), 28.8 (CH_3_), 33.0 (CH_2_), 36.0 (CH_2_), 38.1 (C_q_), 38.4 (C_q_), 39.6 (CH_2_), 41.1 (C_q_), 43.9 (C_q_), 46.5 (C_q_), 53.9 (CH_2_), 56.9 (CH), 57.3 (CH), 61.7 (CH), 66.3 (CH_2_), 78.2 (CH), 128.3 (CH), 128.6 (2 × CH), 128.6 (2 × CH), 135.9 (C_q_), 177.1 (C=O), 204.0 (CHO). C_28_H_38_O_4_: 438.5989. HRMS (ESI+): *m*/*z* calcd. for C_28_H_39_O_4_ [M + H]^+^ 439.2848; found 439.28230.

#### 3.1.2. (4*R*,4a*S*,6a*S*,7*R*,8*R*,9*S*,11b*S*)-Benzyl 7-(((4-fluorobenzyl)amino)methyl)-8-hydroxy-4,9,11b-trimethyltetradecahydro-6a,9-methanocyclohepta[a]naphthalene-4-carboxylate (**5**)

To a solution of **4** (1.14 mmol, 500 mg) in dry EtOH (20 mL), 4-fluorobenzylamine (1.14 mmol, 130 µL) was added in one portion, and the solution was stirred at room temperature for 3 h and then evaporated to dryness. The residue was dissolved in dry EtOH (20 mL), stirred for a further 1 h, and evaporated to dryness again. The product was dissolved in dry MeOH (20 mL), and NaBH_4_ (2.28 mmol, 90 mg) was added in small portions to the mixture under ice cooling. After stirring for 4 h at room temperature, the mixture was evaporated to dryness, and the residue was dissolved in H_2_O (50 mL) and extracted with DCM (3 × 50 mL). The combined organic layer was dried (Na_2_SO_4_), filtered, and evaporated to dryness. The crude product obtained was purified by column chromatography on silica gel (CHCl_3_/MeOH = 19:1). Yield: 440 mg (70%); colourless oil; [*α*]_D_^20^ = −38 (*c* 1.6 MeOH); ^1^H-NMR (500 MHz, CDCl_3_) δ (ppm): 0.65 (s, 3H), 0.83–0.88 (m, 2H), 0.90 (s, 3H), 0.93–1.07 (m, 5H), 1.13–1.15 (m, 1H), 1.19 (s, 3H), 1.31 (d, 1H, *J* = 11.5 Hz), 1.40 (d, 1H, *J* = 14.2 Hz), 1.53–1.84 (m, 9H), 2.20 (d, 1H, *J* = 13.4 Hz), 2.33 (t, 1H, *J* = 11.9 Hz), 2.89 (dd, 1H, *J* = 3.7 Hz, 11.2 Hz), 3.44 (d, 1H, *J* = 4.8 Hz), 3.65 (d, 1H, *J* = 13.1 Hz), 3.85 (d, 1H, *J* = 13.1 Hz), 5.07 (dd, 2H, *J* = 12.4 Hz, *J* = 12.4 Hz), 7.01 (t, 2H, *J* = 8.5 Hz), 7.30–7.36 (m, 7H); ^13^C-NMR (125 MHz, CDCl_3_) δ (ppm): 13.3 (CH_3_), 18.9 (CH_2_), 19.5 (CH_2_), 22.2 (CH_2_), 25.1 (CH_3_), 29.0 (CH_3_), 33.0 (CH_2_), 35.0 (CH_2_), 38.0 (CH_2_), 38.2 (C_q_), 39.6 (CH_2_), 40.7 (C_q_), 42.3 (C_q_), 43.9 (C_q_), 48.0 (CH), 51.6 (CH_2_), 53.2 (CH_2_), 54.2 (CH_2_), 57.2 (CH), 57.7 (CH), 66.0 (CH_2_), 88.3 (CH), 115.2 (CH), 115.3 (CH), 128.0 (CH), 128.3 (CH), 128.4 (2 × CH), 129.8 (2 × CH), 129.9 (CH), 135.4 (C_q-F_), 136.1 (2 × C_q_), 136.2 (C_q-F_), 161.1 (C_q-F_), 163.0 (C_q-F_), 177.1 (C=O); ^19^F-NMR (470 MHz, CDCl_3_) δ (ppm): −116.2 (C_q-F_). C_35_H_46_FNO_3_: 547.7430. HRMS (ESI+): *m*/*z* calcd. for C_35_H_47_FNO_3_ [M + H]^+^ 548.3540; found 548.3533.

#### 3.1.3. (4*R*,4a*S*,6aS,7*R*,8*R*,9*S*,11b*S*)-Benzyl 7-(((*tert*-butoxycarbonyl)(4-fluorobenzyl)amino)methyl)-8-hydroxy-4,9,11b-trimethyltetradecahydro-6a,9-methanocyclohepta[a]naphthalene-4-carboxylate (**6**)

To a solution of **5** (0.91 mmol, 500 mg) in dry DCM (10 mL), *tert*-butyloxycarbonyl anhydride (0.91 mmol, 200 mg) was added in one portion. The solution was stirred at room temperature for 2 h and then evaporated to dryness, and the residue was dissolved in DCM (10 mL) and extracted with H_2_O (3 × 10 mL). The organic layer was dried (Na_2_SO_4_), filtered, and evaporated to dryness. The crude product obtained was purified by column chromatography on silica gel (*n*-hexane/EtOAc = 4:1). Yield: 470 mg (79%); colourless oil; [*α*]_D_^20^ = −1 (*c* 2.80 MeOH); ^1^H-NMR (500 MHz, CDCl_3_) δ (ppm): 0.70 (s, 3H), 0.83–0.87 (m, 1H), 0.90 (s, 3H), 0.92–1.06 (m, 5H), 1.12–1.18 (m, 4H), 1.37–1.42 (m, 2H), 1.47 (s, 9H), 1.56–1.84 (m, 8H), 2.14–2.21 (m, 2H), 3.03 (dd, 1H, *J* = 4.5 Hz, *J* = 13.8 Hz), 3.55 (t, 1H, *J* = 13.1 Hz), 3.64 (d, 1H, *J* = 4.4 Hz), 4.18 (d, 1H, *J* = 15.5 Hz), 4.66 (d, 1H, *J* = 15.5 Hz), 4.87 (d, 1H, *J* = 12.5 Hz), 5.18 (d, 1H, *J* = 12.5 Hz), 6.99 (t, 2H, *J* = 8.6 Hz), 7.23–7.31 (m, 7H); ^13^C-NMR (125 MHz, CDCl_3_) δ (ppm): 13.5 (CH_3_), 19.0 (CH_2_), 19.5 (CH_2_), 22.0 (CH_2_), 25.1 (CH_3_), 28.5 (3 × CH_3_), 29.0 (CH_3_), 33.2 (CH_2_), 34.9 (CH_2_), 37.9 (CH_2_), 38.2 (C_q_), 39.5 (CH_2_), 41.0 (C_q_), 42.9 (C_q_), 43.8 (C_q_), 45.6 (CH), 47.5 (CH_2_), 49.3 (CH_2_), 53.9 (CH_2_), 57.3 (CH), 57.7 (CH), 65.8 (CH_2_), 80.0 (C_q_), 85.6 (CH), 115.3 (CH), 115.5 (CH), 127.9 (CH), 127.9 (2 × CH), 128.4 (2 × CH), 129.4 (CH), 129.5 (CH), 134.3 (2 × C_q_), 136.3 (C_q_), 156.5 (C_q-F_), 161.1 (C_q-F_), 163.1 (C_q-F_), 176.8 (C=O); ^19^F-NMR (470 MHz, CDCl_3_) δ (ppm): −115.0 (C_q-F_). C_40_H_54_FNO_5_: 647.3986. HRMS (ESI+): *m*/*z* calcd. for C_40_H_55_FNO_5_ [M + H]^+^ 648.4064; found 648.4074.

#### 3.1.4. (4*R*,4a*S*,6a*S*,7*R*,8*R*,9*S*,11b*S*)-7-(((*tert*-butoxycarbonyl)(4-fluorobenzyl)amino)methyl)-8-hydroxy-4,9,11b-trimethyltetradecahydro-6a,9-methanocyclohepta[a]naphthalene-4-carboxylic Acid (**7**)

To a suspension of palladium on carbon (5% Pd/C, 120 mg) in *n*-hexane/EtOAc (1:1, 20 mL) we added **6** (0.77 mmol, 500 mg) in *n*-hexane/EtOAc (1:1, 20 mL), and the mixture was stirred in an H_2_ atmosphere (1 atm) at room temperature. After the completion of the reaction (monitored by TLC for 24 h), the mixture was filtered through a Celite pad, and the solution was evaporated to dryness. The crude product was purified by column chromatography on silica gel (CHCl_3_/MeOH = 9:1). Yield: 350 mg (82%), colourless oil; [*α*]_D_^20^ = −9 (*c* 0.67 MeOH); ^1^H-NMR (500 MHz, CDCl_3_) δ (ppm): 0.85–0.89 (m, 1H), 0.90 (s, 3H), 0.92 (s, 3H), 0.94–1.05 (m, 4H), 1.09–1.11 (m, 1H), 1.13–1.18 (m, 1H), 1.20 (s, 3H), 1.37–1.42 (m, 1H), 1.49 (s, 9H), 1.58–1.81 (m, 9H), 2.16–2.19 (m, 2H), 2.99–3.02 (m, 1H), 3.56 (t, 1H, *J* = 12.5 Hz), 3.76 (d, 1H, *J* = 4.4 Hz), 3.95 (d, 1H, *J* = 15.3 Hz), 4.86–4.88 (m, 1H), 7.00 (t, 2H, *J* = 8.6 Hz), 7.24–7.26 (m, 2H); ^13^C-NMR (125 MHz, CDCl_3_) δ (ppm): 13.6 (CH_3_), 18.8 (CH_2_), 19.6 (CH_2_), 21.9 (CH_2_), 25.2 (CH_3_), 28.5 (3 × CH_3_), 29.0 (CH_3_), 33.2 (CH_2_), 34.8 (CH_2_), 37.8 (CH_2_), 38.4 (C_q_), 39.6 (CH_2_), 41.0 (C_q_), 42.9 (C_q_), 43.7 (C_q_), 45.9 (CH), 46.8 (CH_2_), 48.6 (CH_2_), 53.9 (CH_2_), 57.2 (CH), 57.7 (CH), 80.1 (C_q_), 115.4 (CH), 115.5 (CH), 129.8 (2 × CH), 156.4 (C_q-F_), 161.2 (C_q-F_), 163.2 (C_q-F_), 171.3 (C_q_), 183.4 (C=O); ^19^F-NMR (470 MHz, CDCl_3_) δ (ppm): −116.2 (C_q-F_). C_33_H_48_FNO_5_: 557.7363. HRMS (ESI+): *m*/*z* calcd. for C_33_H_49_FNO_5_ [M + H]^+^ 558.3595; found 558.3607.

#### 3.1.5. (4*R*,4a*S*,6a*S*,7*R*,8*R*,9*S*,11b*S*)-7-(((4-Fluorobenzyl)amino)methyl)-8-hydroxy-4,9,11b-trimethyltetradecahydro-6a,9-methanocyclohepta[a]naphthalene-4-carboxylic Acid (**8**)

To a solution of **7** (0.54 mmol, 300 mg) in dry DCM (15 mL) at 0 °C we added TFA (6.53 mmol, 0.50 mL). The ice bath was removed and the reaction mixture was stirred at room temperature for 3 h. The solvent and TFA were removed in vacuo and the residue was diluted with dry DCM. The mixture was cooled to 0 °C and we added TEA (1.23 mmol, 171.10 µL). The resulting homogeneous mixture was allowed to warm to room temperature and stirred for 1 h. The solution was evaporated to dryness and the crude product was purified by column chromatography on silica gel (CHCl_3_/MeOH = 9:1). Yield: 190 mg (77%); white crystals; m.p. 149–150 °C; [*α*]_D_^20^ = −39 (*c* 0.28 MeOH); ^1^H-NMR (500 MHz, CDCl_3_) δ (ppm): 0.77 (s, 3H), 0.80–0.84 (m, 2H), 0.90 (s, 3H), 0.94–1.03 (m, 3H), 1.06–1.09 (m, 4H), 1.13–1.18 (m, 1H), 1.29 (d, 1H, *J* = 11.4 Hz), 1.35 (d, 1H, *J* = 14.1 Hz), 1.52–1.85 (m, 8H), 2.09 (d, 1H, *J* = 13.1 Hz), 2.21 (d, 1H, *J* = 12.0 Hz), 2.52 (d, 1H, *J* = 11.6 Hz), 3.10 (d, 1H, *J* = 8.4 Hz), 3.42 (s, 1H), 3.86 (d, 1H, *J* = 12.4 Hz), 3.97 (d, 1H, *J* = 12.4 Hz), 7.04 (t, 2H, *J* = 8.4 Hz), 7.39 (t, 2H, *J* = 6.9 Hz); ^13^C-NMR (125 MHz, CDCl_3_) δ (ppm): 14.6 (CH_3_), 18.9 (CH_2_), 19.4 (CH_2_), 22.0 (CH_2_), 25.1 (CH_3_), 29.2 (CH_3_), 33.0 (CH_2_), 34.8 (CH_2_), 37.8 (CH_2_), 38.1 (C_q_), 39.6 (CH_2_), 41.5 (C_q_), 43.3 (C_q_), 43.9 (C_q_), 46.0 (CH), 51.1 (CH_2_), 52.3 (CH_2_), 56.7 (CH), 56.9 (CH), 86.1 (CH), 115.6 (CH), 115.8 (CH), 130.1 (C_q_), 131.4 (CH), 131.5 (CH), 161.8 (C_q-F_), 163.8 (C_q-F_), 182.5 (C=O). C_28_H_40_FNO_3_: 457.6205. HRMS (ESI+): *m*/*z* calcd. for C_28_H_41_FNO_3_ [M + H]^+^ 458.3070; found 458.3060.

#### 3.1.6. 4-Bromobutyl Acrylate (**9**) and 3-(4-Bromobutoxy)-3-oxopropyl Acrylate (**10**)

To a suspension of potassium carbonate (2.78 mmol, 380 mg) in dry acetone (50 mL), acrylic acid containing approx. 20% dimer (2.78 mmol, 200 mg) and 1,4-dibromobutane (2.78 mmol, 332 µL) was added and the mixture was stirred at room temperature for 1 day. After the completion of the reaction (monitored by TLC for 24 h), the mixture was filtered through filter paper and the solution was evaporated to dryness. The crude product was purified by column chromatography on silica gel (*n*-hexane/EtOAc = 3:1). Yield: 351 mg (product **9**, 61%), 202 mg (product **10**, 26%); colourless oil; ^1^H-NMR (500 MHz, CDCl_3_) δ (ppm): 1.18–1.84 (m, 2H), 1.91–1.96 (m, 2H), 2.69 (t, 2H, *J* = 6.3 Hz), 3.42 (t, 2H, *J* = 6.6 Hz), 4.16 (t, 2H, *J* = 6.2 Hz), 4.44 (t, 2H, *J* = 6.4 Hz), 5.83 (d, 1H *J* = 10.5 Hz), 6.11 (dd, 1H, *J* = 10.5 Hz, *J* = 17.0 Hz), 6.40 (d, 1H, *J* = 17,1 Hz); ^13^C-NMR (125 MHz, CDCl_3_) δ (ppm): 27.3 (CH_2_), 29.2 (CH_2_), 32.7 (CH_2_), 34.0 (CH_2_), 59.9 (CH_2_), 63.8 (CH_2_), 128.1 (CH), 131.0 (CH_2_), 165.8 (C_q_), 170.5 (C_q_); C_10_H_15_BrO_4_: 279.1277. HRMS (ESI+): *m*/*z* calcd. for C_10_H_16_BrO_4_ [M + H]^+^ 280.1356; found 280.1368.

#### 3.1.7. (4*R*,*4aS*,6a*S*,7*R*,8*R*,9*S*,11b*S*)-4-(Acryloyloxy)butyl 7-(((*tert*-butoxycarbonyl)(4-fluorobenzyl)amino)methyl)-8-hydroxy-4,9,11b-trimethyltetradecahydro-6a,9-methanocyclohepta[a]naphthalene-4-carboxylate (**11**)

To a suspension of potassium carbonate (0.44 mmol, 30 mg) in dry acetone (15 mL) we added **7** (0.22 mmol, 100 mg) and 4-bromobutyl acrylate (0.22 mmol, 35 µL), and the mixture was stirred at room temperature for one day. After the completion of the reaction (monitored by TLC, 24 h), the mixture was filtered through filter paper, and the solution was evaporated to dryness. The crude product was purified by column chromatography on silica gel (*n*-hexane/EtOAc = 3:1). Yield: 120 mg (83%); colourless oil; [*α*]_D_^20^ = −17 (*c* 0.05 MeOH); ^1^H-NMR (500 MHz, CDCl_3_) δ (ppm): 0.72 (s, 3H), 0.84–0.87 (m, 1H), 0.90 (s, 3H), 0.95–1.06 (m, 5H), 1.13 (s, 3H), 1.15–1.20 (m, 1H), 1.39–1.43 (m, 3H), 1.47 (s, 9H), 1.56–1.83 (m, 11H), 2.13–2.20 (m, 2H), 3.02 (dd, 1H, *J* = 4.6 Hz, *J* = 13.8 Hz), 3.58–3.65 (m, 2H), 3.81–3.86 (m, 1H), 4.08–4.17 (m, 4H), 4.78 (d, 1H, *J* = 15.6 Hz), 5.81 (d, 1H, *J* = 10.5 Hz), 6.11 (dd, 1H, *J* = 10.5 Hz, *J* = 17.6 Hz), 6.39 (d, 1H, *J* = 17.6 Hz), 7.01 (t, 2H, *J* = 8.7 Hz), 7.22–7.25 (m, 2H); ^13^C-NMR (125 MHz, CDCl_3_) δ (ppm): 13.4 (CH_3_), 19.0 (CH_2_), 19.5 (CH_2_), 22.1 (CH_2_), 25.1 (CH_3_), 25.3 (CH_2_), 25.6 (CH_2_), 28.5 (3 × CH_3_), 29.0 (CH_3_), 33.3 (CH_2_), 34.9 (CH_2_), 38.0 (CH_2_), 38.2 (C_q_), 39.5 (CH_2_), 41.0 (C_q_), 42.8 (C_q_), 43.8 (C_q_), 45.5 (CH), 47.1 (CH_2_), 48.8 (CH_2_), 54.0 (CH_2_), 57.2 (CH), 57.8 (CH), 63.4 (CH_2_), 64.0 (CH_2_), 80.1 (C_q_), 85.7 (CH), 115.4 (CH), 115.6 (CH), 128.5 (CH), 129.3 (2 × CH), 130.6 (CH_2_), 134.2 (C_q_), 134.3 (C_q_), 156.5 (C_q-F_), 161.2 (C_q-F_), 163.1 (C_q-F_), 166.2 (C=O), 177.1 (C=O); ^19^F-NMR (470 MHz, CDCl_3_) δ (ppm): −115.0 (C_q-F_). C_40_H_58_FNO_7_: 683.8894. HRMS (ESI+): *m*/*z* calcd. for C_40_H_59_FNO_7_ [M + H]^+^ 684.4064; found 684.4283.

#### 3.1.8. (4*R*,4a*S*,6a*S*,7*R*,8*R*,9*S*,11b*S*)-4-((3-(Acryloyloxy)propanoyl)oxy)butyl 7-(((*tert*-butoxycarbonyl)(4-fluorobenzyl)amino)methyl)-8-hydroxy-4,9,11b-trimethyltetradecahydro-6a,9-methanocyclohepta[a]naphthalene-4-carboxylate (**12**)

To a suspension of potassium carbonate (0.44 mmol, 30 mg) in dry acetone (15 mL) we added **7** (0.22 mmol, 100 mg) and 3-(4-bromobutoxy)-3-oxopropyl acrylate-product **10** (0.22 mmol, 60 mg), and the mixture was stirred at room temperature for one day. After the completion of the reaction (monitored by TLC for 24 h), the mixture was filtered through filter paper, and the solution was evaporated to dryness. The crude product was purified by column chromatography on silica gel (*n*-hexane/EtOAc = 3:1). Yield: 130 mg (77%); colourless oil; [*α*]_D_^20^ = +8.7 (*c* 0.11 MeOH); ^1^H-NMR (500 MHz, CDCl_3_) δ (ppm): 0.72 (s, 3H), 0.84–0.88 (m, 1H), 0.90 (s, 3H), 0.95–1.06 (m, 5H), 1.12 (s, 3H), 1.15–1.20 (m, 1H), 1.39–1.43 (m, 3H), 1.47 (s, 9H), 1.57–1.82 (m, 11H), 2.12–2.20 (m, 2H), 2.68 (t, 2H, *J* = 6.3 Hz), 3.02 (dd, 1H, *J* = 4.6 Hz, *J* = 13.8 Hz), 3.57–3.65 (m, 2H), 3.80–3.84 (m, 1H), 4.07–4.16 (m, 4H), 4.43 (t, 2H, *J* = 6.5 Hz), 4.79 (d, 1H, *J* = 15.6 Hz), 5.81 (d, 1H, *J* = 10.5 Hz), 6.10 (dd, 1H, *J* = 10.5 Hz, *J* = 17.6 Hz), 6.39 (d, 1H, *J* = 17.6 Hz), 7.01 (t, 2H, *J* = 8.9 Hz), 7.22–7.25 (m, 2H); ^13^C-NMR (125 MHz, CDCl_3_) δ (ppm): 13.4 (CH_3_), 19.0 (CH_2_), 19.5 (CH_2_), 22.1 (CH_2_), 25.1 (CH_3_), 25.2 (CH_2_), 25.5 (CH_2_), 28.5 (3 × CH_3_), 29.1 (CH_3_), 33.3 (CH_2_), 34.0 (CH_2_), 34.9 (CH_2_), 38.0 (CH_2_), 38.2 (C_q_), 39.5 (CH_2_), 41.0 (C_q_), 42.8 (C_q_), 43.8 (C_q_), 45.5 (CH), 47.1 (CH_2_), 48.8 (CH_2_), 54.0 (CH_2_), 57.2 (CH), 57.8 (CH), 59.9 (CH_2_), 63.3 (CH_2_), 64.3 (CH_2_), 80.1 (C_q_), 85.6 (CH), 115.4 (CH), 115.5 (CH), 128.2 (CH), 129.3 (2 × CH), 131.0 (CH_2_), 134.2 (C_q_), 134.3 (C_q_), 156.5 (C_q-F_), 161.2 (C_q-F_), 163.1 (C_q-F_), 165.8 (C=O), 170.5 (C=O), 177.1 (C=O); ^19^F-NMR (470 MHz, CDCl_3_) δ (ppm): −115.0 (C_q-F_). ^19^F-NMR (470 MHz, CDCl_3_) δ (ppm): −115.3 (C_q-F_). C_43_H_62_FNO_9_: 755.9521. HRMS (ESI+): *m*/*z* calcd. for C_43_H_63_FNO_9_ [M + H]^+^ 756.9600; found 756.4495.

#### 3.1.9. (4*R*,4a*S*,6a*S*,7*R*,8*R*,9*S*,11b*S*)-Prop-2-yn-1-yl 7-(((*tert*-butoxycarbonyl)(4-fluorobenzyl)amino)methyl)-8-hydroxy-4,9,11b-trimethyltetradecahydro-6a,9-methanocyclohepta[a]naphthalene-4-carboxylate (**13**)

To a suspension of potassium carbonate (0.72 mmol, 100 mg) in dry acetone (15 mL) we added **7** (0.36 mmol, 200 mg) and propargyl bromide (0.36 mmol, 28 µL), and the mixture was stirred at room temperature for one day. After the completion of the reaction (monitored by TLC for 24 h), the mixture was filtered through filter paper, and the solution was evaporated to dryness. The crude product was purified by column chromatography on silica gel (n-hexane/EtOAc = 4:1). Yield: 190 mg (88%); colourless oil; [*α*]_D_^20^ = −17 (*c* 0.23 MeOH); ^1^H-NMR (500 MHz, CDCl_3_) δ (ppm): 0.75 (s, 3H), 0.84–0.88 (m, 1H), 0.93 (s, 3H), 0.95–1.08 (m, 5H), 1.13–1.16 (m, 4H), 1.57–1.80 (m, 7H), 2.15–2.19 (m, 2H), 2.26 (s, 1H), 2.26–2.30 (m, 2H), 3.06 (dd, 1H, *J* = 4.2 Hz, *J* = 13.5 Hz), 3.60 (t, 1H, *J* = 12.9 Hz), 3.67 (m, 1H), 4.16 (d, 1H, *J* = 15.5 Hz), 4.51 (d, 1H, J = 15.6 Hz), 4.66 (d, 1H, *J* = 15.5 Hz), 4.74 (d, 1H, *J* = 15.7 Hz), 7.03 (t, 2H, *J* = 9.1 Hz), 7.25–7.28 (m, 2H); ^13^C-NMR (125 MHz, CDCl_3_) δ (ppm): 13.6 (CH_3_), 18.9 (CH_2_), 19.5 (CH_2_), 21.9 (CH_2_), 25.1 (CH_3_), 28.4 (3 × CH_3_), 28.8 (CH_3_), 33.2 (CH_2_), 34.9 (CH_2_), 37.9 (CH_2_), 38.2 (C_q_), 39.5 (CH_2_), 41.0 (C_q_), 42.9 (C_q_), 43.8 (C_q_), 45.7 (CH), 47.5 (CH_2_), 49.1 (CH_2_), 51.3 (CH_2_), 53.9 (CH_2_), 57.2 (CH), 57.7 (CH), 74.4 (C_q_), 80.0 (C_q_), 85.6 (CH), 115.4 (CH), 115.5 (CH), 129.3 (CH), 129.4 (CH), 134.3 (2 × C_q_), 156.5 (C_q_), 161.1 (C_q-F_), 163.1 (C_q-F_), 176.2 (C=O). C_36_H_50_FNO_5_: 595.3673. HRMS (ESI+): *m*/*z* calcd. for C_36_H_51_FNO_3_ [M + H]^+^ 596.3751; found 596.3759.

#### 3.1.10. (4*R*,4a*S*,6a*S*,7*R*,8*R*,9*S*,11b*S*)-Prop-2-yn-1-yl 7-(((4-fluorobenzyl)amino)methyl)-8-hydroxy-4,9,11b-trimethyltetradecahydro-6a,9-methanocyclohepta[a]naphthalene-4-carboxylate (**14**)

To a solution of **13** (0.34 mmol, 200 mg) in dry DCM (15 mL) at 0 °C we added TFA (6.53 mmol, 0.50 mL). The ice bath was removed and the reaction mixture was stirred at RT for 3 h. The solvent and TFA were removed in vacuo and the residue was diluted with dry DCM. The mixture was cooled to °C and TEA (0.68 mmol, 95 µL) was added. The resulting homogeneous mixture was allowed to warm to RT and stirred for 1 h, and the solution was evaporated to dryness. The crude product was purified by column chromatography on silica gel (CHCl_3_/MeOH = 19:1). Yield: 130 mg (79%); white crystals; m.p. 104–105 °C; [*α*]_D_^20^ = −52 (*c* 1.06 MeOH); ^1^H-NMR (500 MHz, CDCl_3_) δ (ppm): 0.73 (s, 3H), 0.83–0.87 (m, 1H), 0.90 (s, 3H), 0.95–1.08 (m, 5H), 1.14–1.20 (m, 4H), 1.31 (d, 1H, *J* = 10.7 Hz), 1.42 (m, 1H, *J* = 14.2 Hz), 1.51–1.63 (m, 4H), 1.69–1.84 (m, 4H), 2.06 (d, 1H, *J* = 12.0 Hz), 2.17 (d, 1H, *J* = 13.3 Hz), 2.52–2.56 (m, 2H), 3.03 (dd, 1H, *J* = 3.4 Hz, *J* = 11.4 Hz), 3.53 (d, 1H, *J* = 4.7 Hz), 3.84 (d, 1H, *J* = 13.1 Hz), 4.01 (d, 1H, *J* = 13.1 Hz), 4.61–4.69 (m, 2H), 7.05 (t, 2H, *J* = 8.6 Hz), 7.42–7.45 (m, 2H); ^13^C-NMR (125 MHz, CDCl_3_) δ (ppm): 13.5 (CH_3_), 18.8 (CH_2_), 19.5 (CH_2_), 21.9 (CH_2_), 24.9 (CH_3_), 28.8 (CH_3_), 33.0 (CH_2_), 34.9 (CH_2_), 37.8 (CH_2_), 38.1 (C_q_), 39.4 (CH_2_), 41.0 (C_q_), 42.5 (C_q_), 43.8 (C_q_), 46.2 (CH), 50.7 (CH_2_), 51.4 (CH_2_), 51.9 (CH_2_), 53.8 (CH_2_), 57.0 (CH), 57.6 (CH), 74.8 (C_q_), 77.9 (C_q_), 87.1 (CH), 115.5 (CH), 115.7 (CH), 130.9 (2 × CH), 131.8 (C_q_), 161.5 (C_q-F_), 163.5 (C_q-F_), 176.3 (C=O); ^19^F-NMR (470 MHz, CDCl_3_) δ (ppm): −113.9 (C_q-F_). C_31_H_42_FNO_3_: 495.6685. HRMS (ESI+): *m*/*z* calcd. for C_31_H_43_FNO_3_ [M + H]^+^ 496.3227; found 496.3233.

#### 3.1.11. General Procedure for the Preparation of Aminoalcohol with Primary Amines and Benzyl Ester Aldehydes

To a solution of **4** (0.23 mmol, 100 mg) in dry EtOH (10 mL), the appropriate primary amine (0.23 mmol) was added in one portion, and the solution was stirred at room temperature for 3 h and then evaporated to dryness. The residue was dissolved in dry EtOH (10 mL), stirred for a further 1 h, and evaporated to dryness again. The product was dissolved in dry MeOH (10 mL), and NaBH_4_ (0.46 mmol, 0.02 g) was added in small portions to the mixture under ice cooling. After stirring for 4 h at room temperature, the mixture was evaporated to dryness, and the residue was dissolved in H_2_O (20 mL) and extracted with DCM (3 × 20 mL). The combined organic layer was dried (Na_2_SO_4_), filtered, and evaporated to dryness. The crude product obtained was purified by column chromatography on silica gel (CHCl_3_/MeOH = 19:1).

##### (4*R*,4a*S*,6a*S*,7*R*,8*R*,9*S*,11b*S*)-Benzyl 7-((((*R*)-1-(4-fluorophenyl)ethyl)amino)methyl)-8-hydroxy-4,9,11b-trimethyltetradecahydro-6a,9-methanocyclohepta[a]naphthalene-4-carboxylate (**15**)

The reaction was performed starting from **4** with (*R*)-4-fluoro-*α*-methylbenzylamine (0.23 mmol, 38 µL) according to the general procedure. Yield: 30 mg (21%); white crystals; m.p. 135–136 °C; [*α*]_D_^20^ = −33 (*c* 0.97 MeOH); ^1^H-NMR (500 MHz, CDCl_3_) δ (ppm): 0.58 (s, 3H), 0.81–0.83 (m, 1H), 0.87 (s, 3H), 0.93–1.06 (m, 5H), 1.15 (s, 3H), 1.30–1.44 (m, 6H), 1.55–1.57 (m, 2H), 1.66 (d, 3H, *J* = 6.7 Hz), 1.72–1.80 (m, 3H), 2.13–2.19 (m, 2H), 2.82 (t, 1H, *J* = 12.5 Hz), 3.00 (d, 1H, *J* = 9.4 Hz), 3.70 (d, 1H, *J* = 4.5 Hz), 4.32–4.33 (m, 1H), 5.02 (d, 1H, *J* = 12.7 Hz), 5.20 (d, 1H, *J* = 12.7 Hz), 7.07 (t, 2H, *J* = 8.4 Hz), 7.31–7.39 (m, 5H), 7.56–7.58 (m, 2H); ^13^C-NMR (125 MHz, CDCl_3_) δ (ppm): 13.2 (CH_3_), 18.9 (CH_2_), 19.2 (CH_3_), 19.4 (CH_2_), 21.7 (CH_2_), 24.7 (CH_3_), 29.0 (CH_3_), 33.0 (CH_2_), 34.8 (CH_2_), 37.8 (CH_2_), 38.0 (C_q_), 39.5 (CH_2_), 41.4 (C_q_), 42.9 (C_q_), 43.7 (C_q_), 44.4 (CH), 48.4 (CH_2_), 53.4 (CH_2_), 57.0 (CH), 57.5 (CH), 57.7 (C_q_), 65.8 (CH_2_), 85.3 (CH), 116.2 (CH), 116.4 (CH), 127.8 (2 × CH), 128.0 (CH), 128.6 (2 × CH), 129.9 (CH), 129.9 (CH), 132.9 (C_q_) 136.4 (Cq), 162.1 (C_q-F_), 164.0 (C_q-F_), 176.6 (C=O). C_36_H_48_FNO_3_: 561.7696. HRMS (ESI+): *m*/*z* calcd. for C_36_H_49_FNO_3_ [M + H]^+^ 562.3696; found 562.3702.

##### (4*R*,4a*S*,6a*S*,7*R*,8*R*,9*S*,11b*S*)-Benzyl 8-hydroxy-4,9,11b-trimethyl-7-((((*R*)-1-phenylpropyl)amino)methyl)tetradecahydro-6a,9-methanocyclohepta[a]naphthalene-4-carboxylate (**16**)

The reaction was performed starting from **4** with (*R*)-(+)-*α*-ethylbenzylamine (0.23 mmol, 37 µL) according to the general procedure. Yield: 60 mg (52%); white crystals; m.p. 154–155 °C; [*α*]_D_^20^ = −24 (*c* 0.26 MeOH); ^1^H-NMR (500 MHz, CDCl_3_) δ (ppm): 0.56 (s, 3H), 0.79 (t, 3H, *J* = 7.4 Hz), 0.82–0.90 (m, 3H), 0.92 (s, 3H), 0.95–1.03 (m, 3H), 1.13 (s, 3H), 1.15–1.17 (m, 1H), 1.33–1.39 (m, 2H), 1.52–1.81 (m, 11H), 2.16 (d, 1H, *J* = 13.2 Hz), 2.32 (t, 1H, *J* = 11.6 Hz), 2.82 (dd, 1H, *J* = 4.1 Hz, *J* = 11.0 Hz), 3.42–3.48 (m, 2H), 4.94 (d, 1H, *J* = 12.7 Hz), 5.12 (d, 1H, *J* = 12.7 Hz), 7.18–7.22 (m, 1H), 7.25–7.33 (m, 9H); ^13^C-NMR (125 MHz, CDCl_3_) δ (ppm): 10.5 (CH_3_), 13.0 (CH_3_), 18.9 (CH_2_), 19.5 (CH_2_), 22.2 (CH_2_), 25.1 (CH_3_), 28.9 (CH_3_), 30.2 (CH_2_), 33.0 (CH_2_), 35.0 (CH_2_), 38.1 (CH_2_), 38.1 (C_q_), 39.6 (CH_2_), 40.6 (C_q_), 42.3 (C_q_), 43.8 (C_q_), 49.0 (CH), 50.2 (CH_2_), 54.4 (CH_2_), 57.2 (CH), 57.9 (CH), 65.7 (CH), 65.7 (CH_2_), 88.6 (CH), 127.0 (CH), 127.1 (2 × CH), 127.9 (CH), 128.1 (2 × CH), 128.4 (2 × CH), 128.4 (2 × CH), 136.3 (Cq), 144.4 (C_q_), 177.0 (C=O). C_37_H_51_NO_3_: 557.8057. HRMS (ESI+): *m*/*z* calcd. for C_37_H_52_NO_3_ [M + H]^+^ 558.8137; found 558.3951.

##### (4*R*,4a*S*,6a*S*,7*R*,8*R*,9*S*,11b*S*)-Benzyl 8-hydroxy-4,9,11b-trimethyl-7-((((*S*)-1-phenylpropyl)amino)methyl)tetradecahydro-6a,9-methanocyclohepta[a]naphthalene-4-carboxylate (**17**)

The reaction was performed starting from **4** with (*S*)-(+)-*α*-ethylbenzylamine (0.23 mmol, 37 µL) according to the general procedure. Yield: 80 mg (68%); white crystals; m.p. 62–63 °C; [*α*]_D_^20^ = −32 (*c* 0.19 MeOH); ^1^H-NMR (500 MHz, CDCl_3_) δ (ppm): 0.67 (s, 3H), 0.78–0.82 (m, 4H), 0.84 (s, 3H), 0.84 (m, 1H), 0.90–1.04 (m, 4H), 1.15 (m, 1H), 1.16 (s, 3H), 1.39–1.43 (m, 2H), 1.56–1.79 (m, 11H), 2.02 (t, 1H, *J* = 11.9 Hz), 2.19 (d, 1H, *J* = 13.6 Hz), 2.75 (dd, 1H, *J* = 3.7 Hz, *J* = 10.8 Hz), 3.30 (d, 1H, *J* = 4.9 Hz), 3.48 (t, 1H, *J* = 6.8 Hz), 5.06 (d, 1H, *J* = 12.5 Hz), 5.10 (d, 1H, *J* = 12.5 Hz), 7.23–7.38 (m, 10H); ^13^C-NMR (125 MHz, CDCl_3_) δ (ppm): 10.7 (CH_3_), 13.3 (CH_3_), 19.0 (CH_2_), 19.6 (CH_2_), 22.2 (CH_2_), 25.1 (CH_3_), 29.0 (CH_3_), 31.7 (CH_2_), 33.0 (CH_2_), 35.0 (CH_2_), 38.0 (CH_2_), 38.2 (C_q_), 39.6 (CH_2_), 40.4 (C_q_), 42.1 (C_q_), 43.9 (C_q_), 48.7 (CH), 49.8 (CH_2_), 54.2 (CH_2_), 57.0 (CH), 57.8 (CH), 65.3 (CH), 66.0 (CH_2_), 88.5 (CH), 127.0 (CH), 127.6 (2 × CH), 128.0 (CH), 128.2 (2 × CH), 128.3 (2 × CH), 128.4 (2 × CH), 136.2 (Cq), 143.8 (C_q_), 177.2 (C=O). C_37_H_51_NO_3_: 557.8057. HRMS (ESI+): *m*/*z* calcd. for C_37_H_52_NO_3_ [M + H]^+^ 558.8137; found 558.3947.

##### (4*R*,4a*S*,6a*S*,7*R*,8*R*,9*S*,11b*S*)-Benzyl 8-hydroxy-4,9,11b-trimethyl-7-((((*R*)-1-(naphthalen-1-yl)ethyl)amino)methyl)tetradecahydro-6a,9-methanocyclohepta[a]naphthalene-4-carboxylate (**18**)

The reaction was performed starting from **4** with (*R*)-(+)-1-(1-naphthyl)ethylamine (0.23 mmol, 37 µL) according to the general procedure. Yield: 60 mg (40%); white crystals; m.p. 118–119 °C; [*α*]_D_^20^ = −36 (*c* 0.43 MeOH); ^1^H-NMR (500 MHz, CDCl_3_) δ (ppm): 0.64 (s, 3H), 0.82–0.90 (m, 2H), 0.93 (s, 3H), 0.95–1.04 (m, 4H), 1.13 (s, 3H), 1.16–1.20 (m, 1H), 1.37 (t, 3H, *J* = 15.7 Hz), 1.49 (d, 3H, *J* = 6.5 Hz), 1.56–1.79 (m, 9H), 2.16 (d, 1H, *J* = 13.1 Hz), 2.41 (t, 1H, *J* = 11.5 Hz), 2.94 (dd, 1H, *J* = 3.1 Hz, *J* = 10.6 Hz), 3.52 (d, 1H, *J* = 4.4 Hz), 3.58–3.60 (m, 1H), 4.89 (d, 1H, *J* = 12.2 Hz), 5.09 (d, 1H, *J* = 12.2 Hz), 7.13–7.15 (m, 1H), 7.21–7.26 (m, 4H), 7.43–7.53 (m, 3H), 7.62 (d, 1H, *J* = 6.8 Hz), 7.72 (d, 1H, *J* = 7.9 Hz), 7.85 (d, 1H, *J* = 7.9 Hz), 8.18 (d, 1H, *J* = 8.4 Hz); ^13^C-NMR (125 MHz, CDCl_3_) δ (ppm): 13.1 (CH_3_), 18.9 (CH_2_), 19.5 (CH_2_), 22.3 (CH_2_), 23.1 (CH_3_), 25.2 (CH_3_), 28.9 (CH_3_), 33.0 (CH_2_), 35.1 (CH_2_), 38.1 (CH), 38.2 (C_q_), 39.6 (CH_2_), 40.6 (C_q_), 42.3 (C_q_), 43.8 (C_q_), 49.2 (CH), 50.3 (CH_2_), 54.3 (CH), 54.4 (CH_2_), 57.1 (CH), 57.8 (CH), 65.8 (CH_2_), 88.8 (CH), 122.5 (CH), 123.1 (CH), 125.4 (CH), 125.7 (CH), 125.8 (CH), 127.3 (CH), 127.9 (CH), 128.2 (2 × CH), 128.4 (2 × CH), 129.0 (CH), 131.0 (C_q_), 134.0 (C_q_), 136.2 (C_q_), 141.7 (C_q_), 177.1 (C=O); C_40_H_51_NO_3_: 593.8378. HRMS (ESI+): *m*/*z* calcd. for C_40_H_52_NO_3_ [M + H]^+^ 594.3947; found 594.3953.

##### (4*R*,4a*S*,6a*S*,7*R*,8*R*,9*S*,11b*S*)-Benzyl 8-hydroxy-4,9,11b-trimethyl-7-((((*S*)-1-(naphthalen-1-yl)ethyl)amino)methyl)tetradecahydro-6a,9-methanocyclohepta[a]naphthalene-4-carboxylate (**19**)

The reaction was performed starting from **4** with (*S*)-(+)-1-(1-naphthyl)ethylamine (0.23 mmol, 37 µL) according to the general procedure. Yield: 60 mg (46%); white crystals; m.p. 126–127 °C; [*α*]_D_^20^ = −31 (*c* 0.12 MeOH); ^1^H-NMR (500 MHz, CDCl_3_) δ (ppm): 0.04 (s, 3H), 0.69–0.73 (m, 1H), 0.85–0.90 (m, 5H), 0.91 (s, 3H), 1.05 (s, 3H), 1.09–1.17 (m, 2H), 1.24–1.30 (m, 3H), 1.53–1.64 (m, 5H), 1.82 (d, 1H, *J* = 12.3 Hz), 2.05–2.07 (m, 4H), 2.26 (d, 1H, *J* = 12.8 Hz), 2.89 (t, 1H, *J* = 12.5 Hz), 3.15–3.17 (m, 1H), 3.87–3.88 (m, 1H), 4.86 (d, 1H, *J* = 12.5 Hz), 5.05 (m, 1H, *J* = 12.5 Hz), 5.60 (s, 1H), 7.36–7.63 (m, 8H), 7.84 (d, 2H, *J* = 8.2 Hz), 8.00 (d, 1H, *J* = 8.5 Hz), 8.24 (d, 1H, *J* = 6.9 Hz); ^13^C-NMR (125 MHz, CDCl_3_) δ (ppm): 12.3 (CH_3_), 18.8 (CH_2_), 19.3 (CH_2_), 21.7 (CH_2_), 22.1 (CH_3_), 24.6 (CH_3_), 28.9 (CH_3_), 33.1 (CH_2_), 34.6 (CH_2_), 37.7 (CH), 37.8 (C_q_), 39.4 (CH_2_), 41.4 (C_q_), 42.8 (C_q_), 43.0 (CH), 43.5 (C_q_), 48.7 (CH_2_), 53.0 (CH), 53.4 (CH_2_), 57.1 (CH), 58.0 (CH), 65.8 (CH_2_), 84.6 (CH), 121.9 (CH), 124.5 (2 × CH), 126.1 (2 × CH), 127.1 (CH), 128.2 (2 × CH), 128.5 (2 × CH), 129.1 (CH), 129.3 (CH), 130.7 (C_q_), 133.0 (C_q_) 134.0 (C_q_), 136.6 (C_q_), 178.0 (C=O); C_40_H_51_NO_3_: 593.8378. HRMS (ESI+): *m*/*z* calcd. for C_40_H_52_NO_3_ [M + H]^+^ 594.3947; found 594.3945.

##### (4*R*,4a*S*,7*R*,8*R*,11b*S*)-Benzyl 7-(((3-(1H-imidazol-1-yl)propyl)amino)methyl)-8-hydroxy-4,9,11b-trimethyltetradecahydro-6a,9-methanocyclohepta[a]naphthalene-4-carboxylate (**20**)

The reaction was performed starting from **4** with 1-(3-aminopropyl)imidazole (0.23 mmol, 27 µL) according to the general procedure. Yield: 30 mg (33%); colourless oil; [*α*]_D_^20^ = −11 (*c* 0.25 MeOH); ^1^H-NMR (500 MHz, CDCl_3_) δ (ppm): 0.67 (s, 3H), 0.84–0.89 (m, 1H), 0.91 (s, 3H), 0.93–1.08 (m, 5H), 1.16–1.20 (m, 4H), 1.32–1.35 (m, 1H), 1.39–1.43 (m, 1H), 1.56–1.85 (m, 9H), 1.91–1.95 (m, 2H), 2.19–2.25 (m, 2H), 2.47–2.52 (m, 1H), 2.66–2.71 (m, 1H), 2.83 (dd, 1H, *J* = 4.0 Hz, *J* = 10.7 Hz), 3.40 (d, 1H, *J* = 5.0 Hz), 3.97–4.07 (m, 2H), 5.03 (d, 1H, *J* = 12.5 Hz), 5.10 (d, 1H, *J* = 12.5 Hz), 6.91 (s, 1H), 7.05 (s, 1H), 7.27–7.35 (m, 5H), 7.48 (s, 1H); ^13^C-NMR (125 MHz, CDCl_3_) δ (ppm): 13.3 (CH_3_), 19.0 (CH_2_), 19.5 (CH_2_), 22.2 (CH_2_), 25.1 (CH_3_), 29.0 (CH_3_), 31.5 (CH_2_), 33.0 (CH_2_), 35.0 (CH_2_), 38.0 (CH_2_) 38.2 (C_q_), 39.6 (CH_2_), 40.7 (C_q_), 42.4 (C_q_), 43.9 (C_q_), 44.9 (CH_2_), 46.9 (CH_2_), 48.4 (CH), 52.4 (CH_2_), 54.3 (CH_2_), 57.2 (CH), 57.8 (CH), 66.0 (CH_2_), 88.6 (CH), 118.8 (CH), 127.9 (CH), 128.2 (2 × CH), 128.4 (2 × CH), 129.5 (C_q_), 136.2 (C_q_), 137.2 (CH), 177.1 (C=O). C_34_H_49_N_3_O_3_: 547.7712. HRMS (ESI+): *m*/*z* calcd. For C_34_H_50_N_3_O_3_ [M + H]^+^ 548.7791; found 548.3862.

#### 3.1.12. General Procedure for the Preparation of Aminoalcohols by the Reaction of Aldehyde **22** with Primary Amines

To a solution of **22** (100 mg, 0.28 mmol) in dry EtOH (10 mL), the appropriate primary amine (0.28 mmol) was added in one portion, and the solution was stirred at room temperature for 3 h and then evaporated to dryness. The residue was dissolved in dry EtOH (10 mL), stirred for a further 1 h, and evaporated to dryness. The product was dissolved in dry MeOH (10 mL), and NaBH_4_ (0.56 mmol, 20 mg) was added in small portions to the mixture under ice cooling. After stirring for 4 h at room temperature, the mixture was evaporated to dryness, and the residue was dissolved in H_2_O (20 mL) and extracted with DCM (3 × 20 mL). The combined organic layer was dried (Na_2_SO_4_), filtered, and evaporated to dryness. The crude product obtained was purified by column chromatography on silica gel (CHCl_3_/MeOH = 19:1).

##### (4*R*,4a*S*,6a*S*,7*R*,8*R*,9*S*,11b*S*)-Methyl 7-((((*R*)-1-(4-fluorophenyl)ethyl)amino)methyl)-8-hydroxy-4,9,11b-trimethyltetradecahydro-6a,9-methanocyclohepta[a]naphthalene-4-carboxylate (**23**)

The reaction was performed starting from **22** with (*R*)-4-fluoro-*α*-methylbenzylamine (0.28 mmol, 38 µL) according to the general procedure. Yield: 60 mg (50%); white crystals; m.p. 129–130 °C; [*α*]_D_^20^ = −56 (*c* 0.17 MeOH); ^1^H-NMR (500 MHz, CDCl_3_) δ (ppm): 0.58 (s, 3H), 0.81–0.91 (m, 3H), 0.93 (s, 3H), 0.95–1.01 (m, 3H), 1.11 (s, 3H), 1.14–1.19 (m, 1H), 1.33 (d, 3H, *J* = 6.5 Hz), 1.35 (m, 1H), 1.38–1.47 (m, 2H), 1.51–1.59 (m, 3H), 1.64–1.69 (m, 2H), 1.75–1.79 (m, 3H), 2.13 (d, 1H, *J* = 13.5 Hz), 2.34 (t, 1H, *J* = 11.6 Hz), 2.78 (dd, 1H, *J* = 4.3 Hz, *J* = 11.0 Hz), 3.47 (d, 1H, *J* = 5.0 Hz), 3.56 (s, 3H), 3.77 (m, 1H), 7.00 (t, 2H, *J* = 8.8 Hz), 7.28–7.31 (m, 2Hz); ^13^C-NMR (125 MHz, CDCl_3_) δ (ppm): 12.8 (CH_3_), 18.9 (CH_2_), 19.5 (CH_2_), 22.1 (CH_2_), 24.3 (CH_3_), 25.1 (CH_3_), 28.8 (CH_3_), 33.0 (CH_2_), 34.9 (CH_2_), 38.0 (CH_2_), 38.0 (C_q_), 39.6 (CH_2_), 40.7 (C_q_), 42.2 (C_q_), 43.7 (C_q_), 48.8 (CH), 50.0 (CH_2_), 51.0 (CH_3_), 54.3 (CH_2_), 56.7 (CH), 57.7 (CH), 58.1 (CH), 88.5 (CH), 115.1 (CH), 115.3 (CH), 127.9 (CH), 128.0 (CH), 141.5 (C_q_), 160.8 (C_q-F_), 162.8 (C_q-F_), 177.9 (C=O); ^19^F-NMR (470 MHz, CDCl_3_) δ (ppm): −116.3 (C_q-F_). C_30_H_44_FNO_3_: 485.6737. HRMS (ESI+): *m*/*z* calcd. for C_30_H_45_FNO_3_ [M + H]^+^ 485.3305; found 485.3321.

##### (4*R*,4a*S*,6a*S*,7*R*,8*R*,9*S*,11b*S*)-Methyl 8-hydroxy-4,9,11b-trimethyl-7-((((*R*)-1-phenylpropyl)amino)methyl)tetradecahydro-6a,9-methanocyclohepta[a]naphthalene-4-carboxylate (**24**)

The reaction was performed starting from **22** with (*R*)-(+)-*α*-ethylbenzylamine (0.28 mmol, 36 µL) according to the general procedure. Yield: 120 mg (88%); white crystals; m.p. 144–145 °C; [*α*]_D_^20^ = −47 (*c* 0.26 MeOH); ^1^H-NMR (500 MHz, CDCl_3_) δ (ppm): 0.58 (s, 3H), 0.81 (t, 3H, *J* = 7.4 Hz), 0.81–0.91 (m, 3H), 0.93 (s, 3H), 0.95–1.02 (m, 3H), 1.10 (s, 3H), 1.14–1.20 (m, 1H), 1.32–1.79 (m, 14H), 2.12 (d, 1H, *J* = 13.3 Hz), 2.30 (t, 1H, *J* = 11.7 Hz), 2.82 (dd, 1H, *J* = 4.2 Hz, *J* = 11.0 Hz), 3.47–3.50 (m, 2H), 3.54 (s, 3H), 7.21–7.25 (m, 1H), 7.26–7.33 (m, 4H); ^13^C-NMR (125 MHz, CDCl_3_) δ (ppm): 10.7 (CH_3_), 12.8 (CH_3_), 18.8 (CH_2_), 19.5 (CH_2_), 22.1 (CH_2_), 25.1 (CH_3_), 28.8 (CH_3_), 30.7 (CH_2_), 33.0 (CH_2_), 34.9 (CH_2_), 38.0 (C_q_), 38.1 (CH_2_), 39.6 (CH_2_), 40.6 (C_q_), 42.2 (C_q_), 43.7 (C_q_), 48.9 (CH), 50.2 (CH_2_), 51.0 (CH_3_), 54.4 (CH_2_), 57.0 (CH), 57.8 (CH), 65.7 (CH), 88.8 (CH), 126.9 (2 × CH), 127.1 (2 × CH), 128.3 (CH), 144.6 (C_q_), 178.0 (C=O); C_31_H_47_NO_3_: 481.7098. HRMS (ESI+): *m*/*z* calcd. for C_31_H_48_NO_3_ [M + H]^+^ 482.3634; found 482.3644.

##### (4*R*,4a*S*,6a*S*,7*R*,8*R*,9*S*,11b*S*)-Methyl 8-hydroxy-4,9,11b-trimethyl-7-((((*S*)-1-phenylpropyl)amino)methyl)tetradecahydro-6a,9-methanocyclohepta[a]naphthalene-4-carboxylate (**25**)

The reaction was performed starting from **22** with (*S*)-(+)-*α*-ethylbenzylamine (0.28 mmol, 36 µL) according to the general procedure. Yield: 60 mg (48%); white crystals; m.p. 115–116 °C; [*α*]_D_^20^ = −23 (*c* 0.27 MeOH); ^1^H-NMR (500 MHz, CDCl_3_) δ (ppm): 0.71 (s, 3H), 0.78–0.83 (m, 4H), 0.85–0.88 (m, 4H), 0.91–1.02 (m, 4H), 1.13 (s, 3H), 1.15–1.21 (m, 2H), 1.39–1.83 (m, 14H), 2.08–2.17 (m, 2H), 2.85 (dd, 1H, *J* = 3.9 Hz, *J* = 11.1 Hz), 3.34 (d, 1H, *J* = 4.9 Hz), 3.53 (t, 1H, *J* = 7.1 Hz), 3.62 (s, 3H), 7.23–7.24 (m, 1H), 7.28–7.34 (m, 4H); ^13^C-NMR (125 MHz, CDCl_3_) δ (ppm): 10.7 (CH_3_), 13.1 (CH_3_), 19.0 (CH_2_), 19.6 (CH_2_), 22.1 (CH_2_), 25.1 (CH_3_), 29.0 (CH_3_), 31.6 (CH_2_), 33.0 (CH_2_), 34.9 (CH_2_), 37.9 (CH), 38.0 (C_q_), 39.6 (CH_2_), 40.4 (C_q_), 42.1 (C_q_), 43.7 (C_q_), 48.5 (CH), 49.6 (CH_2_), 51.3 (CH_3_), 54.3 (CH_2_), 57.1 (CH), 57.9 (CH), 64.9 (CH), 88.5 (CH), 126.9 (CH), 127.5 (2 × CH), 128.3 (2 × CH), 144.0 (C_q_), 177.9 (C=O); C_31_H_47_NO_3_: 481.7098. HRMS (ESI+): *m*/*z* calcd. for C_31_H_48_NO_3_ [M + H]^+^ 482.3634; found 482.3631.

##### (4*R*,4a*S*,6a*S*,7*R*,8*R*,9*S*,11b*S*)-Methyl 8-hydroxy-4,9,11b-trimethyl-7-((((*S*)-1-(naphthalen-2-yl)ethyl)amino)methyl)tetradecahydro-6a,9-methanocyclohepta[a]naphthalene-4-carboxylate (**26**)

The reaction was performed starting from **22** with (*S*)-(+)-1-(1-naphthyl)ethylamine (0.28 mmol, 45 µL) according to the general procedure. Yield: 60 mg (42%); white crystals; m.p. 194–195 °C; [*α*]_D_^20^ = −65 (*c* 0.47 MeOH); ^1^H-NMR (500 MHz, CDCl_3_) δ (ppm): 0.66 (s, 3H), 0.81–0.87 (m, 2H), 0.89 (s, 3H), 0.91–1.01 (m, 4H), 1.10 (s, 3H), 1.14–1.23 (m, 2H), 1.37–1.40 (m, 1H), 1.43–1.46 (m, 1H), 1.48 (d, 3H, *J* = 6.6 Hz), 1.59–1.82 (m, 6H), 1.87–1.89 (m, 1H), 2.12 (d, 1H, *J* = 13.4 Hz), 2.23 (t, 1H, *J* = 12.0 Hz), 2.31 (s, 1H), 2.95 (dd, 1H, *J* = 3.9 Hz, *J* = 11.0 Hz), 3.43 (s, 3H), 3.50 (d, 1H, *J* = 4.8 Hz), 4.67–4.71 (m, 1H), 7.45–7.52 (m, 3H), 7.69 (d, 1H, *J* = 7.0 Hz), 7.74 (d, 1H, *J* = 8.2 Hz), 7.86 (d, 1H, *J* = 7.8 Hz), 8.20 (d, 1H, *J* = 8.4 Hz); ^13^C-NMR (125 MHz, CDCl_3_) δ (ppm): 13.1 (CH_3_), 18.9 (CH_2_), 19.6 (CH_2_), 22.1 (CH_2_), 24.5 (CH_3_), 25.1 (CH_3_), 28.9 (CH_3_), 33.0 (CH_2_), 34.9 (CH_2_), 37.9 (CH), 38.1 (C_q_), 39.6 (CH_2_), 40.5 (C_q_), 42.2 (C_q_), 43.7 (C_q_), 48.6 (CH_2_), 50.2 (CH_2_), 51.0 (CH_3_), 53.7 (CH), 54.3 (CH_2_), 57.1 (CH), 57.8 (CH), 88.6 (CH), 122.6 (CH), 122.9 (CH), 125.3 (CH), 125.7 (2 × CH), 127.1 (CH), 128.9 (CH), 131.5 (C_q_), 134.0 (C_q_), 141.2 (C_q_), 177.9 (C=O); C_34_H_47_NO_3_: 517.7419. HRMS (ESI+): *m*/*z* calcd. for C_34_H_48_NO_3_ [M + H]^+^ 518.3634; found 518.3629.

##### (4*R*,4a*S*,6a*S*,7*R*,8*R*,9*S*,11b*S*)-Methyl 8-hydroxy-4,9,11b-trimethyl-7-((((*R*)-1-(naphthalen-2-yl)ethyl)amino)methyl)tetradecahydro-6a,9-methanocyclohepta[a]naphthalene-4-carboxylate (**27**)

The reaction was performed starting from **22** with (*R*)-(+)-1-(1-naphthyl)ethylamine (0.28 mmol, 45 µL) according to the general procedure. Yield: 60 mg (38%); white crystals; m.p. 153–154 °C; [*α*]_D_^20^ = −40 (*c* 0.75 MeOH); ^1^H-NMR (500 MHz, CDCl_3_) δ (ppm): 0.66 (s, 3H), 0.84–0.92 (m, 2H), 0.93 (s, 3H), 0.95–1.02 (m, 3H), 1.09 (s, 3H), 1.15–1.22 (m, 1H), 1.37–1.47 (m, 3H), 1.51 (d, 3H, *J* = 6.7 Hz), 1.57–1.86 (m, 9H), 2.12 (d, 1H, *J* = 13.3 Hz), 2.42 (t, 1H, *J* = 11.3 Hz), 2.96 (dd, 1H, *J* = 3.9 Hz, *J* = 10.9 Hz), 3.50 (s, 3H), 3.52 (d, 1H, *J* = 5.0 Hz), 4.63 (m, 1H), 7.44–7.54 (m, 3H), 7.64 (d, 1H, *J* = 7.2 Hz), 7.74 (d, 1H, *J* = 8.3 Hz), 7.87 (d, 1H, *J* = 7.6 Hz), 8.19 (d, 1H, *J* = 8.5 Hz); ^13^C-NMR (125 MHz, CDCl_3_) δ (ppm): 12.8 (CH_3_), 18.9 (CH_2_), 19.5 (CH_2_), 22.2 (CH_2_), 23.5 (CH_3_), 25.1 (CH_3_), 28.8 (CH_3_), 33.0 (CH_2_), 34.9 (CH_2_), 38.1 (CH), 38.1 (C_q_), 39.6 (CH_2_), 40.7 (C_q_), 42.3 (C_q_), 43.7 (C_q_), 49.1 (CH_2_), 50.4 (CH_2_), 51.0 (CH_3_), 54.3 (CH_2_), 54.4 (C_q_), 57.0 (CH), 57.8 (CH), 88.8 (CH), 122.4 (CH), 123.0 (CH), 125.4 (CH), 125.7 (CH), 125.8 (CH), 127.2 (CH), 129.0 (CH), 131.2 (C_q_), 134.1 (C_q_), 141.6 (C_q_), 178.0 (C=O); C_34_H_47_NO_3_: 517.7419. HRMS (ESI+): *m*/*z* calcd. for C_34_H_48_NO_3_ [M + H]^+^ 518.3634; found 518.3632.

##### (4*R*,4a*S*,6a*S*,7*R*,8*R*,9*S*,11b*S*)-Methyl 7-(((3-(1H-imidazol-1-yl)propyl)amino)methyl)-8-hydroxy-4,9,11b-trimethyltetradecahydro-6a,9-methanocyclohepta[a]naphthalene-4-carboxylate (**28**)

The reaction was performed starting from **22** with 1-(3-aminopropyl)imidazole (0.28 mmol, 33 µL) according to the general procedure. Yield: 60 mg (42%); white crystals; m.p. 107–108 °C; [*α*]_D_^20^ = −39 (*c* 0.20 MeOH); ^1^H-NMR (500 MHz, CDCl_3_) δ (ppm): 0.71 (s, 3H), 0.84–0.90 (m, 1H), 0.92 (s, 3H), 0.95–1.08 (m, 5H), 1.16 (s, 3H), 1.18–1.22 (m, 1H), 1.34–1.42 (m, 2H), 1.64–1.81 (m, 9H), 1.94–1.99 (m, 2H), 2.16 (d, 1H, *J* = 13.3 Hz), 2.31 (t, 1H, *J* = 11.8 Hz), 2.55–2.60 (m, 1H), 2.71–2.76 (m, 1H), 2.95 (dd, 1H, *J* = 4.0 Hz, *J* = 11.0 Hz), 3.43 (d, 1H, *J* = 4.8 Hz), 3.62 (s, 3H), 4.00–4.09 (m, 2H), 6.92 (s, 1H), 7.05 (s, 3H), 7.48 (s, 1H); ^13^C-NMR (125 MHz, CDCl_3_) δ (ppm): 13.2 (CH_3_), 19.0 (CH_2_), 19.6 (CH_2_), 22.2 (CH_2_), 25.1 (CH_3_), 29.0 (CH_3_), 31.4 (CH_2_), 33.1 (CH_2_), 35.1 (CH_2_), 38.0 (CH_2_), 38.2 (Cq), 39.7 (CH_2_), 40.8 (C_q_), 42.4 (C_q_), 43.8 (C_q_), 44.9 (CH_2_), 46.9 (CH_2_), 48.3 (CH), 51.2 (CH_3_), 52.5 (CH_2_), 54.3 (CH_2_), 57.3 (CH), 57.8 (CH), 88.7 (CH), 118.9 (CH), 129.6 (CH), 137.3 (CH), 177.9 (C=O); C_28_H_45_N_3_O_3_: 471.6752. HRMS (ESI+): *m*/*z* calcd. For C_28_H_46_N_3_O_3_ [M + H]^+^ 472.3539; found 472.3551.

### 3.2. Determination of the Antiproliferative Effect

The growth-inhibitory effects of the isosteviol-based 1,3-aminoalcohols were determined by a standard MTT (3-(4,5-dimethylthiazol-2-yl)-2,5-diphenyltetrazolium bromide) assay on a panel containing five cell lines, including Hela (cervical cancer), MDA-MB-231 and MCF-7 (breast cancers), and A2780 ovarian cancer cells [34]. All cell lines were purchased from the European Collection of Cell Cultures (Salisbury, UK). The cells were maintained in a minimal essential medium supplemented with 10% foetal bovine serum, 1% non-essential amino acids, and 1% penicillin–streptomycin at 37 °C in a humidified atmosphere containing 5% CO_2_. All media and supplements were obtained from Lonza Group Ltd. (Basel, Switzerland). Cancer cells were seeded into 96-well plates (5000 cells/well); after overnight incubation, the test compound was added in two different concentrations (10 µM and 30 µM) and incubated for another 72 h under cell-culturing conditions. In the next step, 20 μL of 5 mg/mL of an MTT solution was added to each well and incubated for a further 4 h. The medium was removed and the precipitated formazan crystals were dissolved in DMSO after 60 min of shaking at 37 °C. As a final step, the absorbance was measured at 545 nm by using a microplate reader. Untreated cells were included as controls. In the case of the most effective compounds (i.e., compounds eliciting higher than 50 or 85% at 10 or 30 μM, respectively), the assays were repeated with a set of dilutions (1.0–30 μM) in order to determine the IC_50_ values. Two independent experiments were performed with five wells for each condition. Calculations were performed using the GraphPad Prism 10.0 software (GraphPad Software Inc., San Diego, CA, USA).

## 4. Conclusions

Starting from isosteviol, a series of diterpenoid 1,3-aminoalcohol derivatives were prepared via stereoselective transformations. To study the effect of the carboxylate ester function at position 4, the free carboxylic acid as well as the benzyl, propargyl, and acryloyl ester analogues were prepared as a new development of our former results in this field. The antiproliferative activity of compounds against human tumour cell lines (A2780, HeLa, MCF-7, and MDA-MB-231) was also investigated. The exchange of the methyl ester to the propargyl group increased the antiproliferative activity, which could be explained by their π–π bonding properties and the increased lipophilicity, while the exchange to the benzyl ester presented no remarkable advantage. Surprisingly, compound **20** with the *N*-(1*H*-imidazol-1-yl)propyl substituent was proven to be the most active derivative, despite our previous observation that *N*-alkyl substitution reduced the antiproliferative activity [26,28]. Since this latter agent seems to be superior to the clinically utilised cisplatin, it can be regarded as a potential hit. It can be useful to investigate additional different aminoalcohol- or aminodiol-type diterpenes with a potential antiproliferative activity in the future. Additionally, Boc-protected aminoalcohol derivative **12** exerted substantial and selective cell growth-inhibiting action against A2780 cells (IC_50_: 4.78 µM), indicating that the structure could be utilised for the design and synthesis of candidates against ovarian cancer.

## Figures and Tables

**Table 1:** Synthesis of 1,3-aminoalcohols **14**–**19** via Schiff intermediates.

Entry	RNH_2_	Product	Yield (%)
1	(*R*)-4-fluoro-*α*-methylbenzylamine	**15**	21
2	(*R*)-(+)-*α*-ethylbenzylamine	**16**	52
3	(*S*)-(+)-*α*-ethylbenzylamine	**17**	68
4	(*S*)-(+)-1-(1-naphthyl)ethylamine	**18**	40
5	(*R*)-(+)-1-(1-naphthyl)ethylamine	**19**	46
6	1-(3-aminopropyl)imidazole	**20**	33

**Table 2:** Synthesis of 1,3-aminoalcohols **23**–**28** via Schiff products.

Entry	RNH_2_	Product	Yield (%)
1	(*R*)-4-fluoro-*α*-methylbenzylamine	**23**	50
2	(*R*)-(+)-*α*-ethylbenzylamine	**24**	88
3	(*S*)-(+)-*α*-ethylbenzylamine	**25**	48
4	(*S*)-(+)-1-(1-naphthyl)ethylamine	**26**	42
5	(*R*)-(+)-1-(1-naphthyl)ethylamine	**27**	38
6	1-(3-aminopropyl)imidazole	**28**	58

## Data Availability

Data are contained within the article and Supplementary Materials.

## Supplementary Materials

The following supporting information can be downloaded at: https://www.mdpi.com/article/10.3390/ph17020262/s1, Figures S1–S139: NMR spectra of the prepared new compounds; Table S1: calculated IC_50_ values and 95% confidence intervals (Cis) of the investigated molecules against four cancer cell lines and non-malignant fibroblasts (NIH/3T3).
