# Supplementary material for: Synthesis and Study of the Structure–Activity Relationship of Antiproliferative N-Substituted Isosteviol-Based 1,3-Aminoalcohols
Source: Pharmaceuticals (Basel). 2024 Feb 19;17(2):262. doi: 10.3390/ph17020262 (PMC10893060; doi:10.3390/ph17020262)

Supporting informations
for

Synthesis Study of the Structure–Activity Relationship of Antiproliferative *N*-Substituted Isosteviol-Based 1,3-Aminoalcohols

Dániel Ozsvár [1], Noémi Bózsity [2], István Zupkó [2,3] and Zsolt Szakonyi [1,3,*]

[1] Interdisciplinary Excellence Center, Institute of Pharmaceutical Chemistry, University of Szeged, Eötvös utca 6, H-6720 Szeged, Hungary; ozsmozs88@gmail.com
[2] Institute of Pharmacodynamics and Biopharmacy, University of Szeged, Eötvös utca 6, H-6720 Szeged, Hungary; bozsity-farago.noemi@szte.hu (N.B.); zupko.istvan@szte.hu (I.Z.)
[3] Interdisciplinary Centre of Natural Products, University of Szeged, Eötvös utca 6, H-6720 Szeged, Hungary
* Correspondence: szakonyi.zsolt@szte.hu; Tel.: +36-62-546809

Contents

Antiproliferative effects of the investigated compounds	Table S1
^{1}H, ^{13}C NMR, and 2D NMR spectra of compound **4**	Figure S1 – S6
^{1}H, ^{13}C NMR, and 2D NMR spectra of compound **5**	Figure S7 – S13
^{1}H, ^{13}C NMR, and 2D NMR spectra of compound **6**	Figure S14 – S20
^{1}H, ^{13}C NMR, and 2D NMR spectra of compound **7**	Figure S21 – S26
^{1}H, ^{13}C NMR, and 2D NMR spectra of compound **8**	Figure S27 – S33
^{1}H, ^{13}C NMR, and 2D NMR spectra of compound **10**	Figure S34 – S39
^{1}H, ^{13}C NMR, and 2D NMR spectra of compound **11**	Figure S40 – S46
^{1}H, ^{13}C NMR, and 2D NMR spectra of compound **12**	Figure S47 – S53
^{1}H, ^{13}C NMR, and 2D NMR spectra of compound **13**	Figure S54 – S59
^{1}H, ^{13}C NMR, and 2D NMR spectra of compound **14**	Figure S60 – S66
^{1}H, ^{13}C NMR, and 2D NMR spectra of compound **15**	Figure S67 – S72
^{1}H, ^{13}C NMR, and 2D NMR spectra of compound **16**	Figure S73 – S78
^{1}H, ^{13}C NMR, and 2D NMR spectra of compound **17**	Figure S79 – S84
^{1}H, ^{13}C NMR, and 2D NMR spectra of compound **18**	Figure S85 – S90
^{1}H, ^{13}C NMR, and 2D NMR spectra of compound **19**	Figure S91 – S96
^{1}H, ^{13}C NMR, and 2D NMR spectra of compound **20**	Figure S97 – S102
^{1}H, ^{13}C NMR, and 2D NMR spectra of compound **23**	Figure S103 – S109
^{1}H, ^{13}C NMR, and 2D NMR spectra of compound **24**	Figure S110 – S115
^{1}H, ^{13}C NMR, and 2D NMR spectra of compound **25**	Figure S116 – S121
^{1}H, ^{13}C NMR, and 2D NMR spectra of compound **26**	Figure S122 – S127
^{1}H, ^{13}C NMR, and 2D NMR spectra of compound **27**	Figure S128 – S133
^{1}H, ^{13}C NMR, and 2D NMR spectra of compound **28**	Figure S134 – S139

Table S1. Calculated IC_{50} values and 95% confidence intervals (CI) of the investigated molecules against four cancer cell lines and non-malignant fibroblasts (NIH/3T3)

Compound	HeLa		A2780		MDA-MB-231		MCF-7		NIH/3T3	
	IC_{50}	CI (95%)	IC_{50}	CI (95%)	IC_{50}	CI (95%)	IC_{50}	CI (95%)	IC_{50}	CI (95%)
**4**	> 10	*	> 10		> 10		> 10		> 10	
**5**	4.17	3.79–4.60	4.57	3.59–5.81	5.98	4.84–7.37	4.29	3.77–4.89	4.27	3.67–4.95
**6**	> 10		> 10		> 10		> 10		> 10	
**7**	> 10		> 10		> 10		> 10		> 10	
**8**	> 10		> 10		> 10		> 10		> 10	
**11**	> 10		> 10		> 10		> 10		> 10	
**12**	> 10		4.78	3.68–6.21	> 10		> 10		> 10	
**14**	2.70	2.48–2.94	1.36	1.19–1.57	2.23	1.32–3.76	1.54	1.33–1.78	5.92	4.03–8.68
**15**	4.18	2.65–6.59	3.91	2.38–6.44	4.28	3.43–5.35	4.20	3.38–5.23	4.80	3.76–6.12
**16**	3.86	3.37–4.42	4.00	3.67–4.36	4.79	4.19–5.48	3.48	3.09–3.90	1.37	1.27–1.48
**17**	4.45	3.38–5.85	3.78	2.16–6.61	4.04	2.19–7.42	4.74	2.38–9.41	1.95	1.78–2.14
**18**	3.05	2.46–3.79	2.89	2.67–3.18	4.63	3.82–5.62	4.85	3.80–6.21	4.61	3.71–5.72
**19**	6.08	5.72–6.46	4.41	3.65–5.32	7.29	4.92–10.81	5.59	4.80–6.52	5.44	4.69–6.31
**20**	1.99	0.483–2.63	3.74	2.83–4.95	1.78	1.51–2.10	1.37	1.24–1.51	4.78	3.60–6.34
**23**	4.16	3.55–4.87	4.24	3.89–4.62	5.08	4.12–6.27	3.16	2.89–3.45	1.79	1.57–2.04
**24**	3.04	2.55–3.62	4.52	3.59–5.70	4.99	4.07–6.11	2.37	1.63–3.45	4.44	3.95–4.99
**25**	4.43	3.94–4.97	4.83	4.28–5.45	6.53	5.29–8.07	4.17	3.45–5.04	3.98	3.59–4.41
**26**	4.33	3.61–5.19	4.11	3.62–4.67	6.07	4.93–7.48	3.67	3.25–4.13	3.12	2.66–3.66
**27**	3.40	1.74–6.66	3.26	2.93–3.61	4.11	3.51–4.81	2.43	2.26–2.62	5.03	4.07–6.23
**28**	3.72	2.77–5.01	4.42	3.03–6.43	4.34	3.01–6.27	3.82	2.50–5.84	1.55	1.47–1.64

* All values are given in μM. No IC_{50} values were calculated when less than 50% cell growth inhibition was detected at 10 μM.

Figure S1

^{1}H-NMR of compound (4*R*,4a*S*,6a*S*,7*S*,8*R*,9*S*,11b*S*)-Benzyl 7-formyl-8-hydroxy-4,9,11b-trimethyltetradecahydro-6a,9-methanocyclohepta[a]naphthalene-4-carboxylate **(4)**:

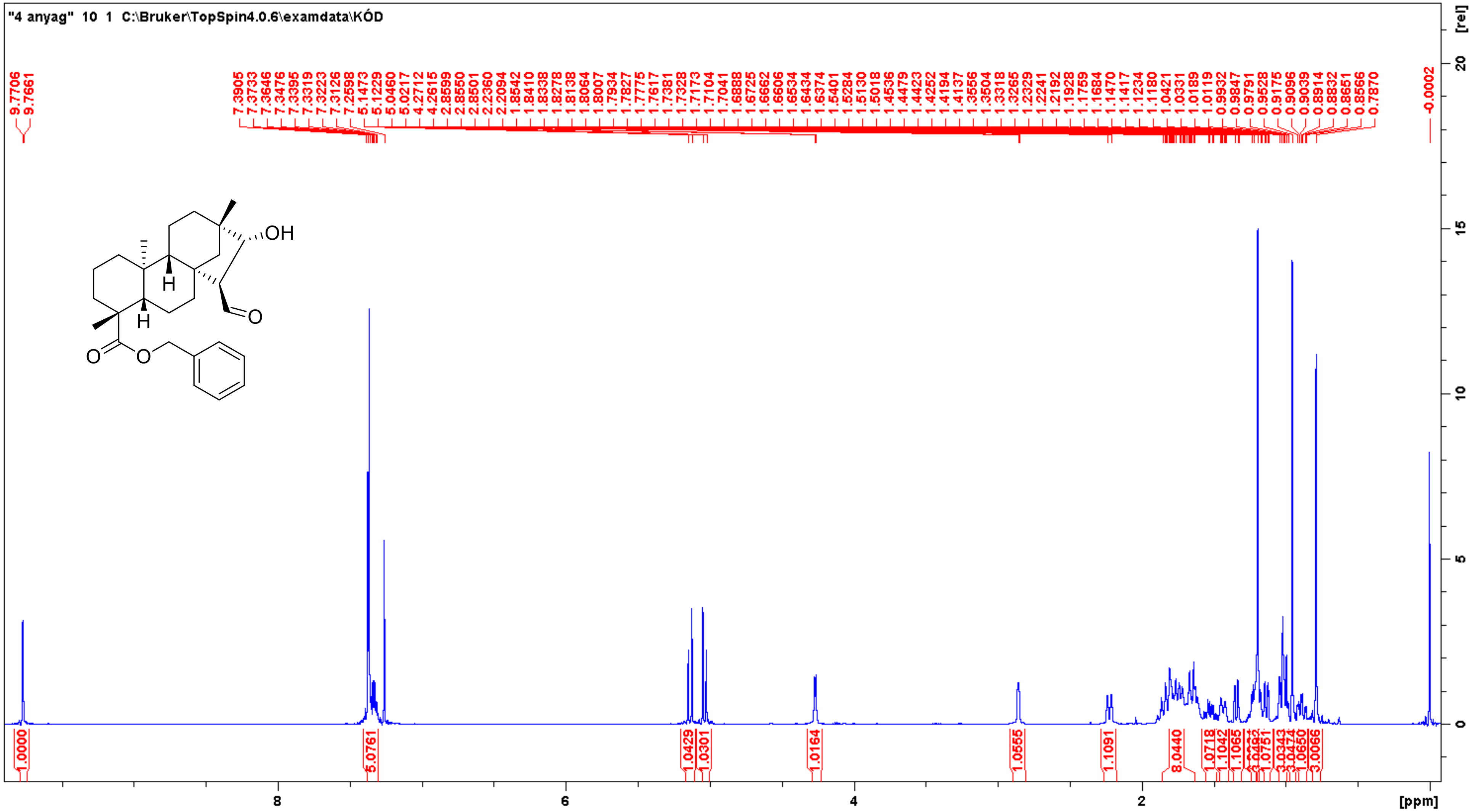

Figure S2

^{13}C-NMR of compound (4*R*,4a*S*,6a*S*,7*S*,8*R*,9*S*,11b*S*)-Benzyl 7-formyl-8-hydroxy-4,9,11b-trimethyltetradecahydro-6a,9-methanocyclohepta[a]naphthalene-4-carboxylate **(4)**:

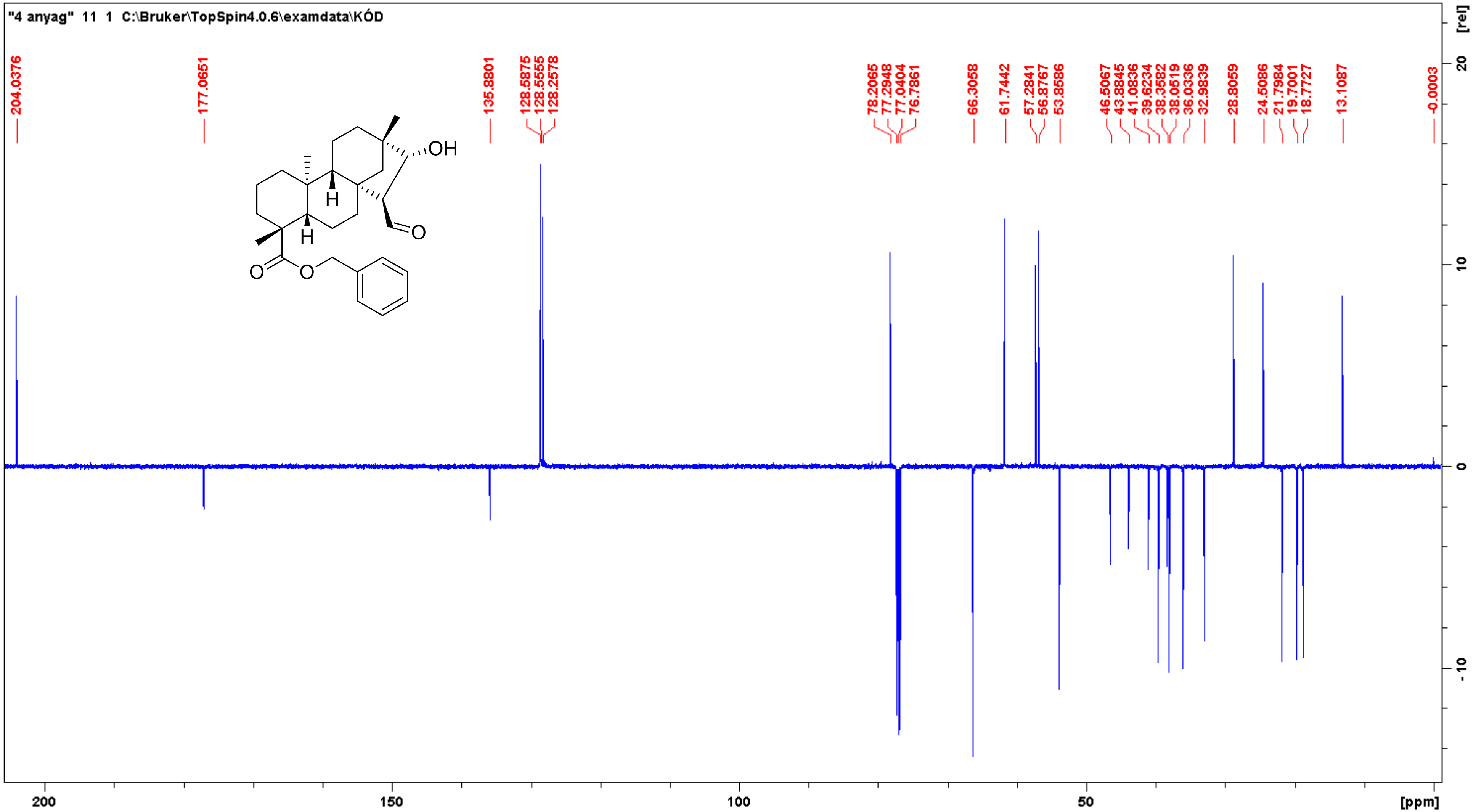

Figure S3

COSY NMR of compound (4*R*,4a*S*,6a*S*,7*S*,8*R*,9*S*,11b*S*)-Benzyl 7-formyl-8-hydroxy-4,9,11b-trimethyltetradecahydro-6a,9-methanocyclohepta[a]naphthalene-4-carboxylate **(4)**:

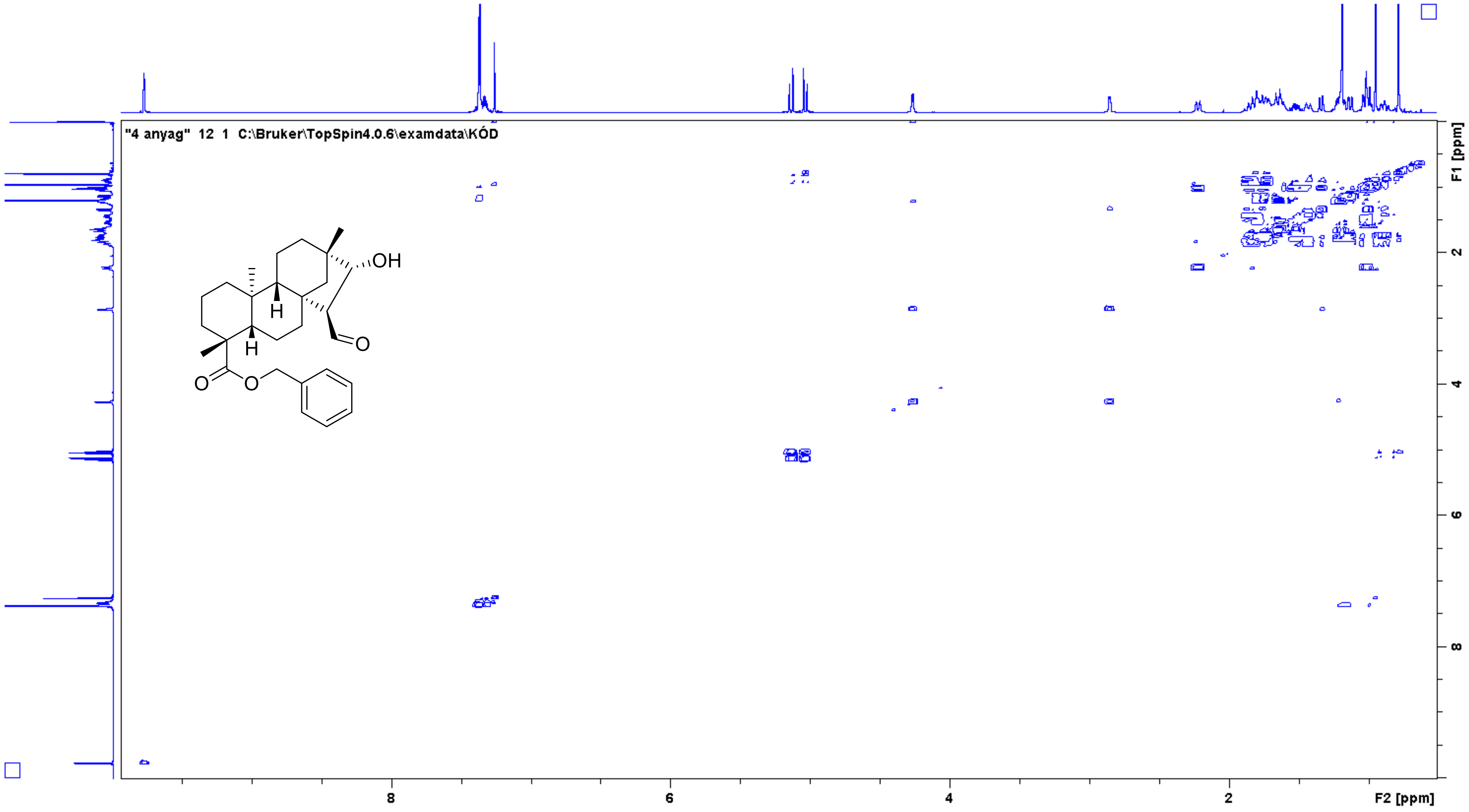

Figure S4

NOESY NMR of compound (4*R*,4a*S*,6a*S*,7*S*,8*R*,9*S*,11b*S*)-Benzyl 7-formyl-8-hydroxy-4,9,11b-trimethyltetradecahydro-6a,9-methanocyclohepta[a]naphthalene-4-carboxylate **(4)**:

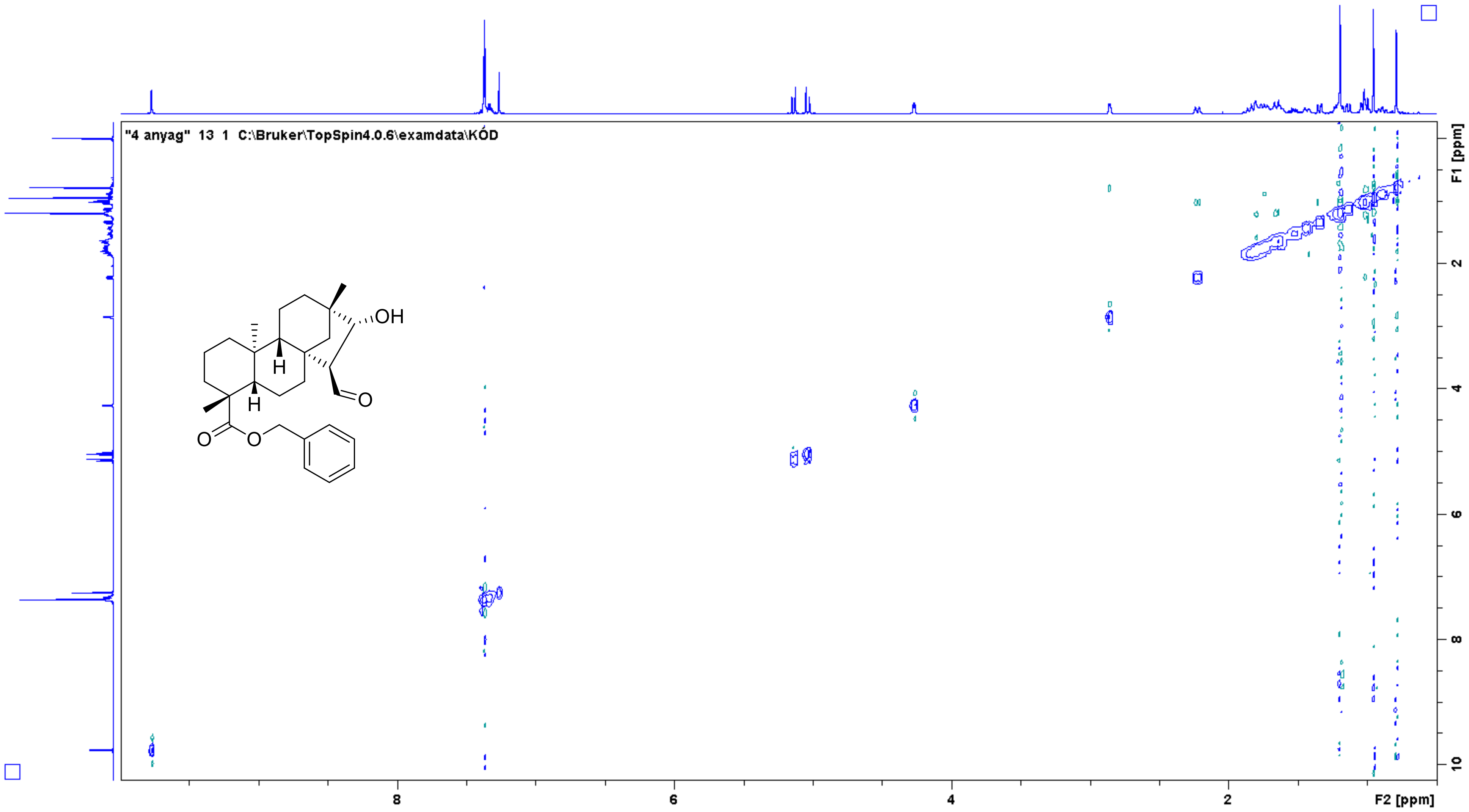

Figure S5

HSQC of compound (4*R*,4a*S*,6a*S*,7*S*,8*R*,9*S*,11b*S*)-Benzyl 7-formyl-8-hydroxy-4,9,11b-trimethyltetradecahydro-6a,9-methanocyclohepta[a]naphthalene-4-carboxylate **(4)**:

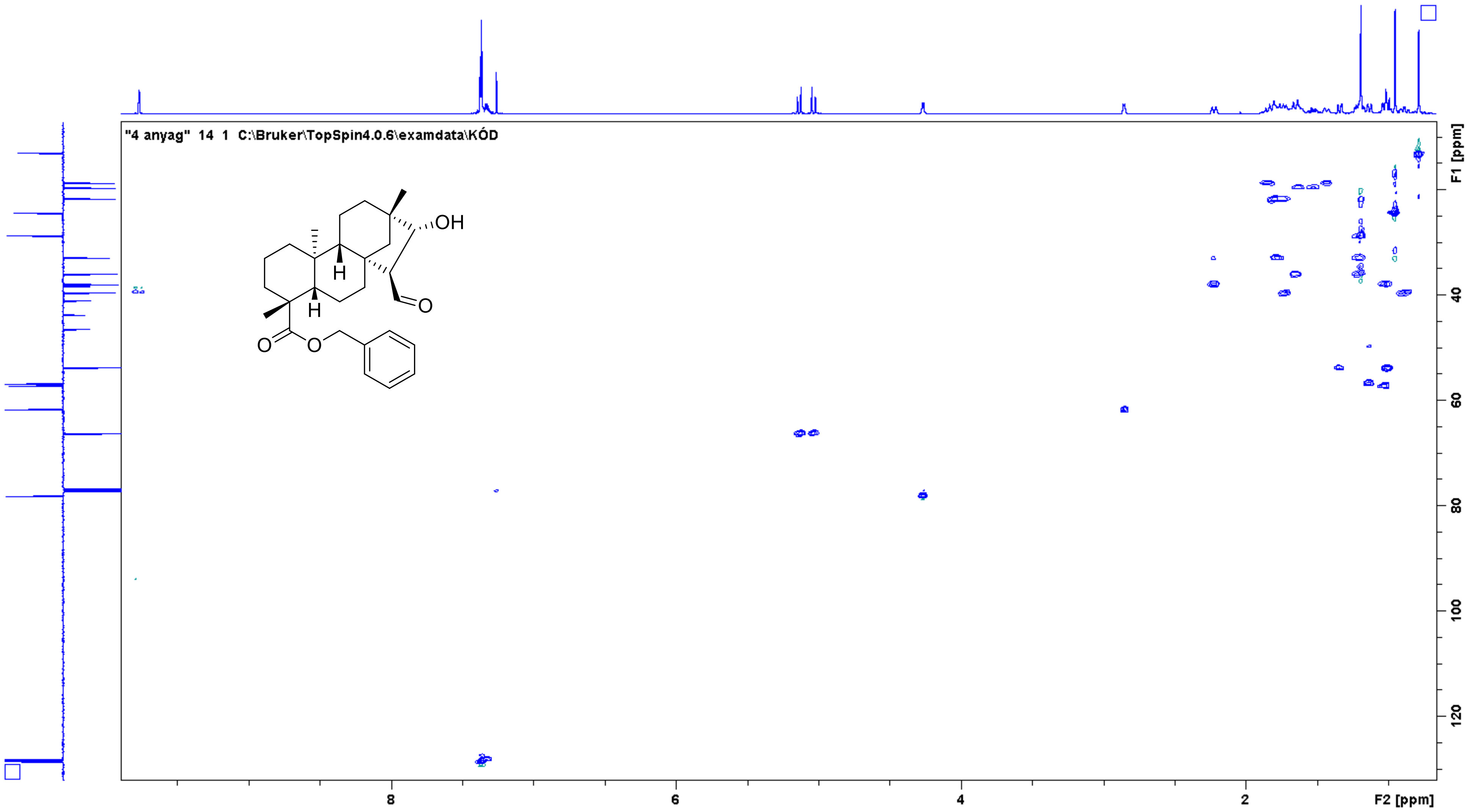

Figure S6

HMBC of compound (4*R*,4a*S*,6a*S*,7*S*,8*R*,9*S*,11b*S*)-Benzyl 7-formyl-8-hydroxy-4,9,11b-trimethyltetradecahydro-6a,9-methanocyclohepta[a]naphthalene-4-carboxylate **(4)**:

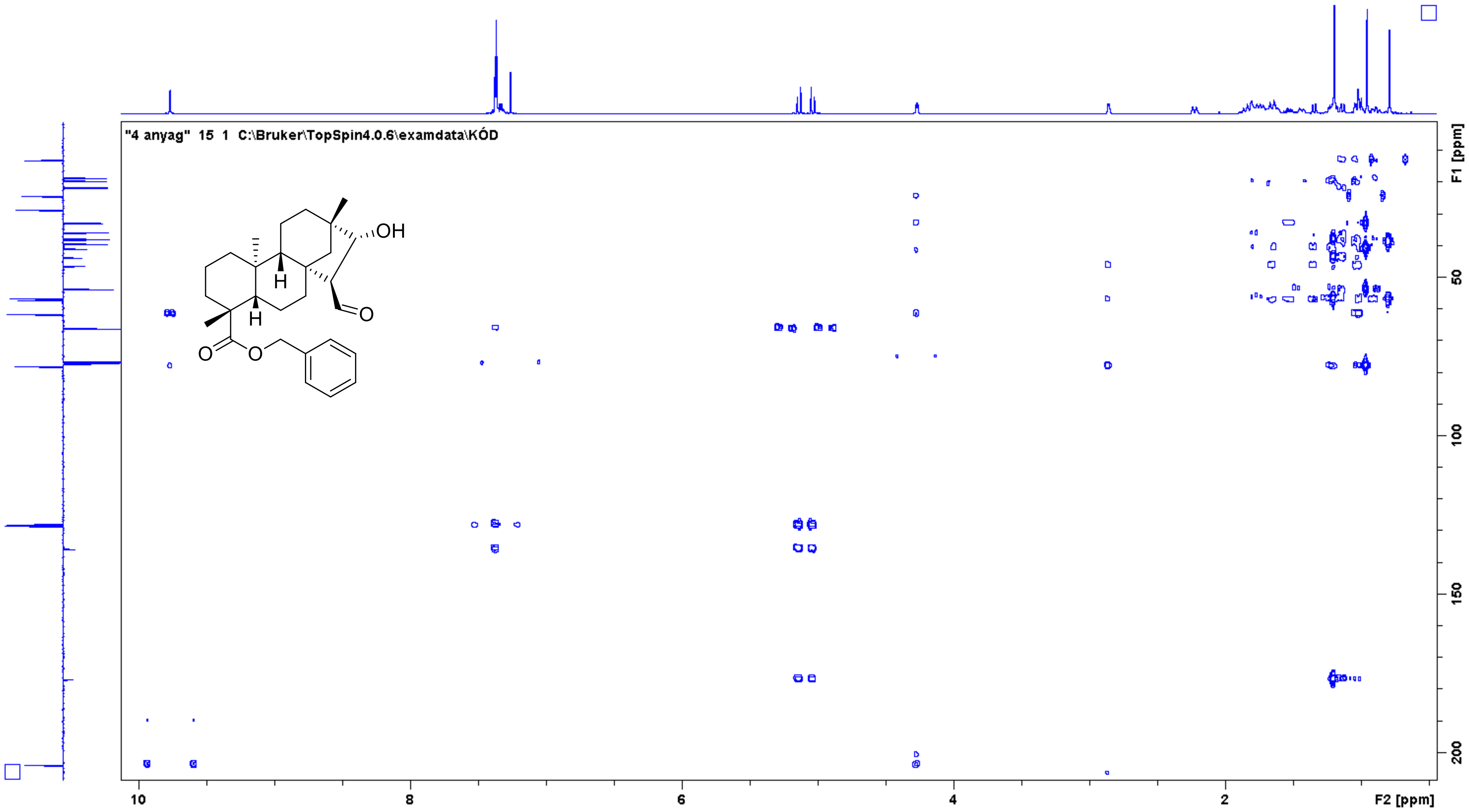

Figure S7

^{1}H-NMR of compound (4*R*,4a*S*,6a*S*,7*R*,8*R*,9*S*,11b*S*)-Benzyl 7-(((4-fluorobenzyl)amino)methyl)-8-hydroxy-4,9,11b-trimethyltetradecahydro-6a,9-methanocyclohepta[a]naphthalene-4-carboxylate **(5)**:

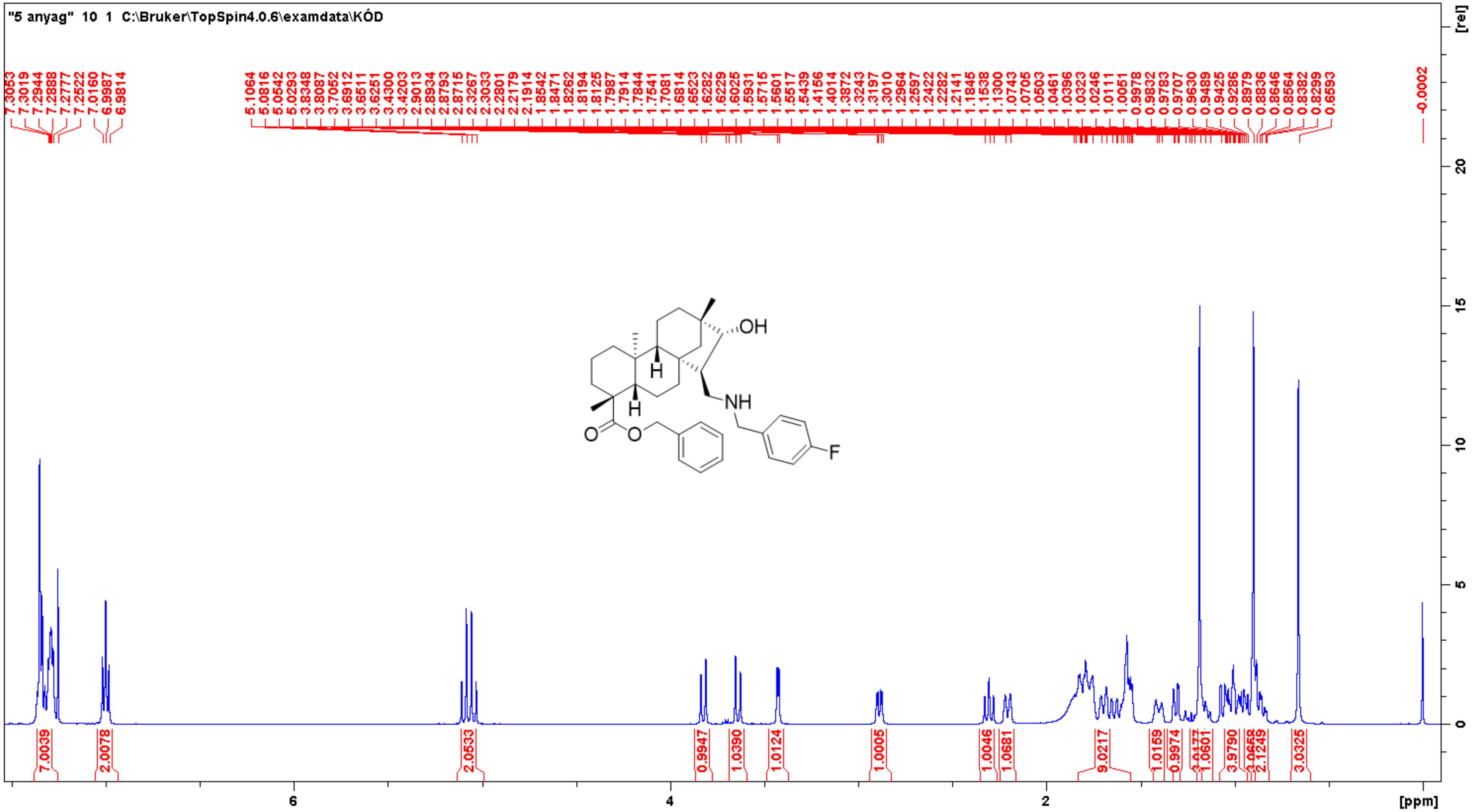

Figure S8

^{13}C-NMR of compound (4*R*,4a*S*,6a*S*,7*R*,8*R*,9*S*,11b*S*)-Benzyl 7-(((4-fluorobenzyl)amino)methyl)-8-hydroxy-4,9,11b-trimethyltetradecahydro-6a,9-methanocyclohepta[a]naphthalene-4-carboxylate **(5)**:

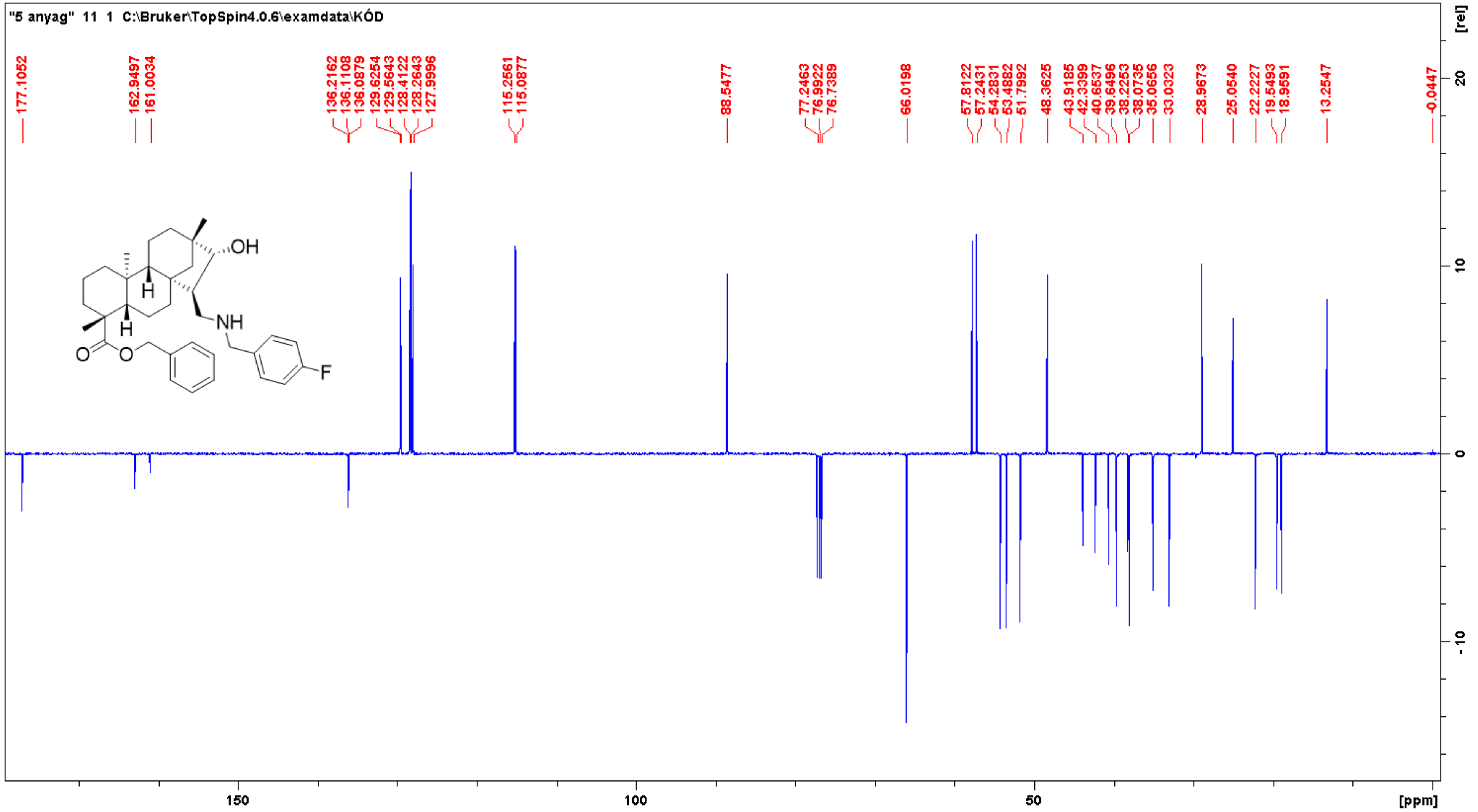

Figure S9

COSY of compound (4*R*,4a*S*,6a*S*,7*R*,8*R*,9*S*,11b*S*)-Benzyl 7-(((4-fluorobenzyl)amino)methyl)-8-hydroxy-4,9,11b-trimethyltetradecahydro-6a,9-methanocyclohepta[a]naphthalene-4-carboxylate **(5)**:

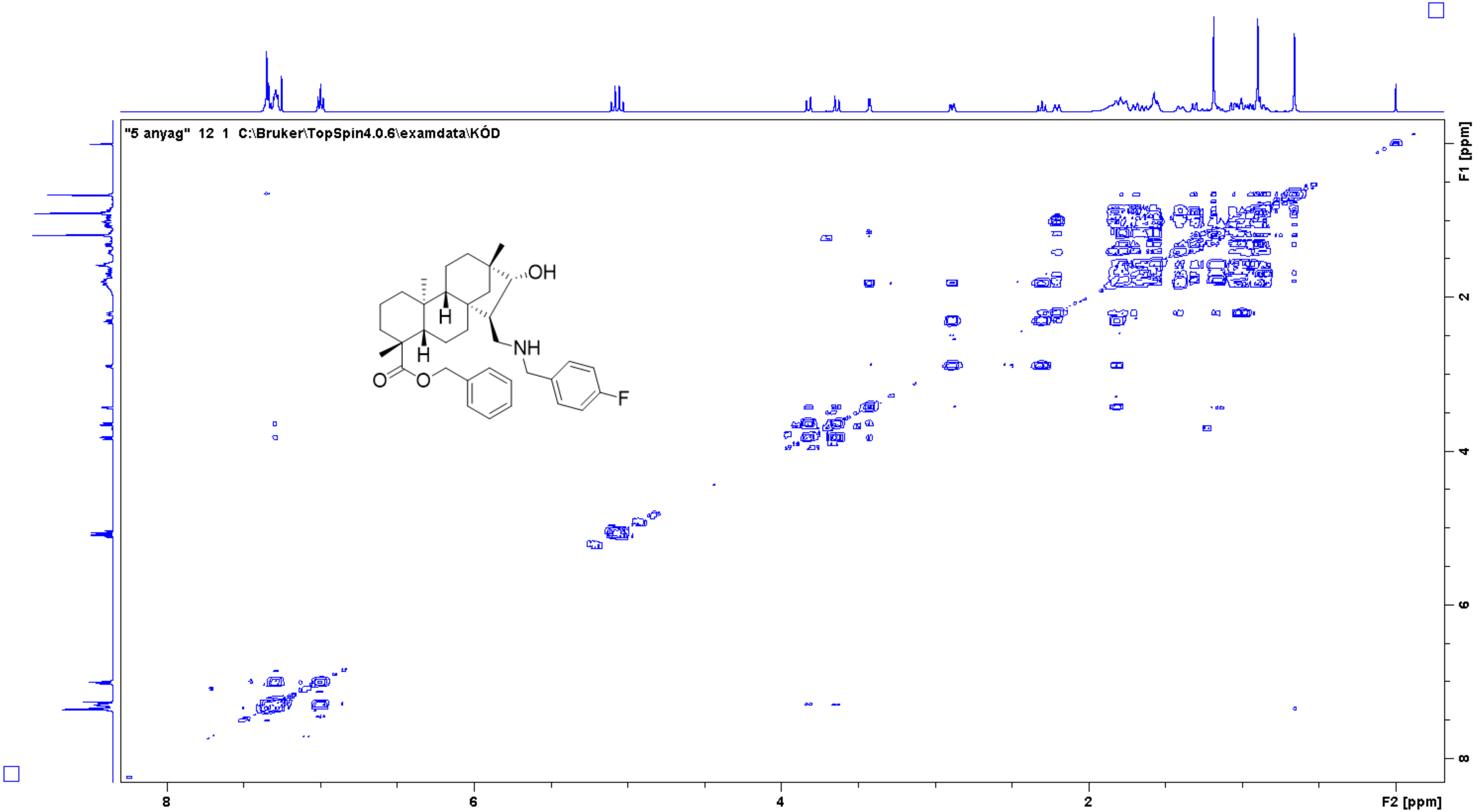

Figure S10

NOESY of compound (4*R*,4a*S*,6a*S*,7*R*,8*R*,9*S*,11b*S*)-Benzyl 7-(((4-fluorobenzyl)amino)methyl)-8-hydroxy-4,9,11b-trimethyltetradecahydro-6a,9-methanocyclohepta[a]naphthalene-4-carboxylate **(5)**:

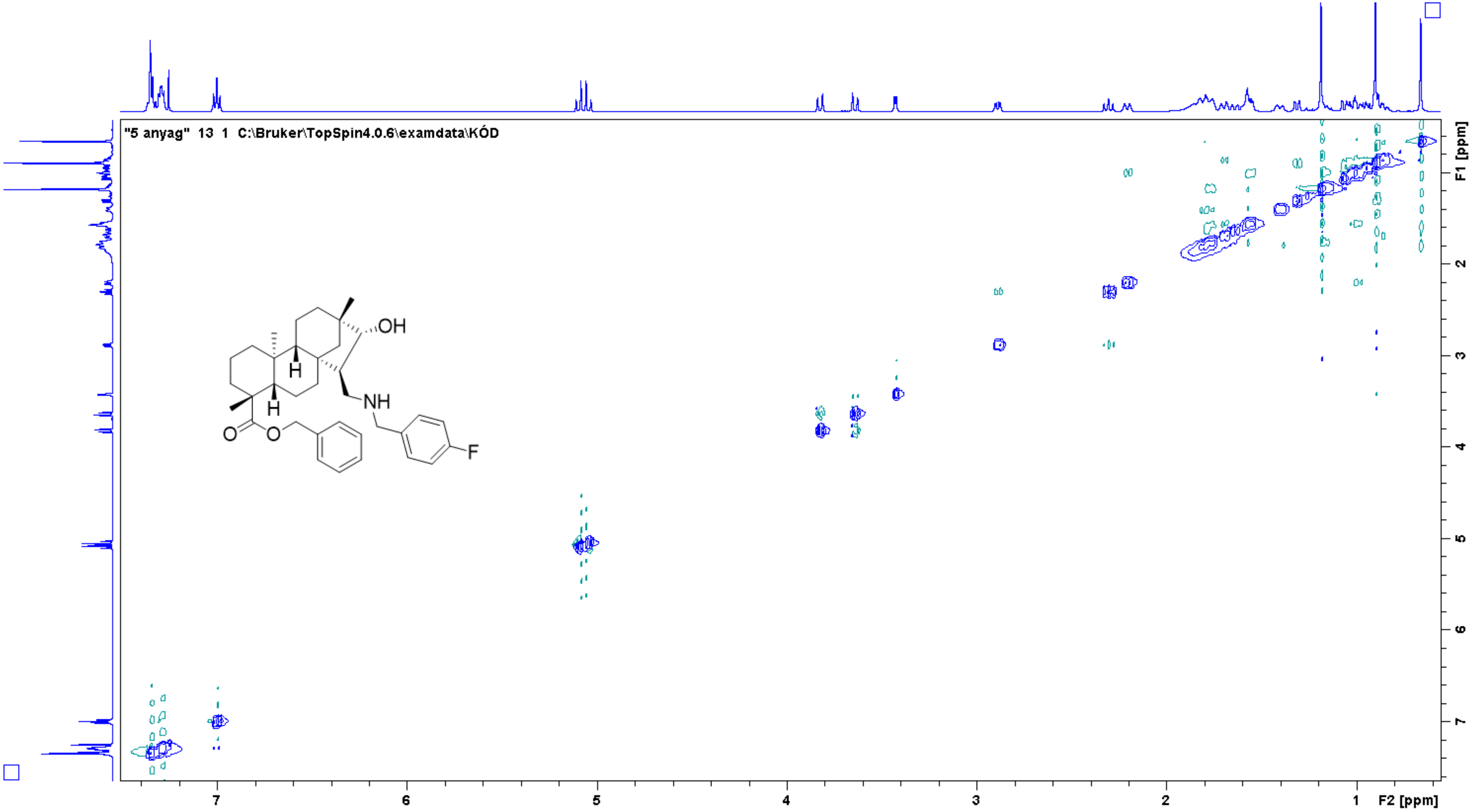

Figure S11

HSQC of compound (4*R*,4a*S*,6a*S*,7*R*,8*R*,9*S*,11b*S*)-Benzyl 7-(((4-fluorobenzyl)amino)methyl)-8-hydroxy-4,9,11b-trimethyltetradecahydro-6a,9-methanocyclohepta[a]naphthalene-4-carboxylate **(5)**:

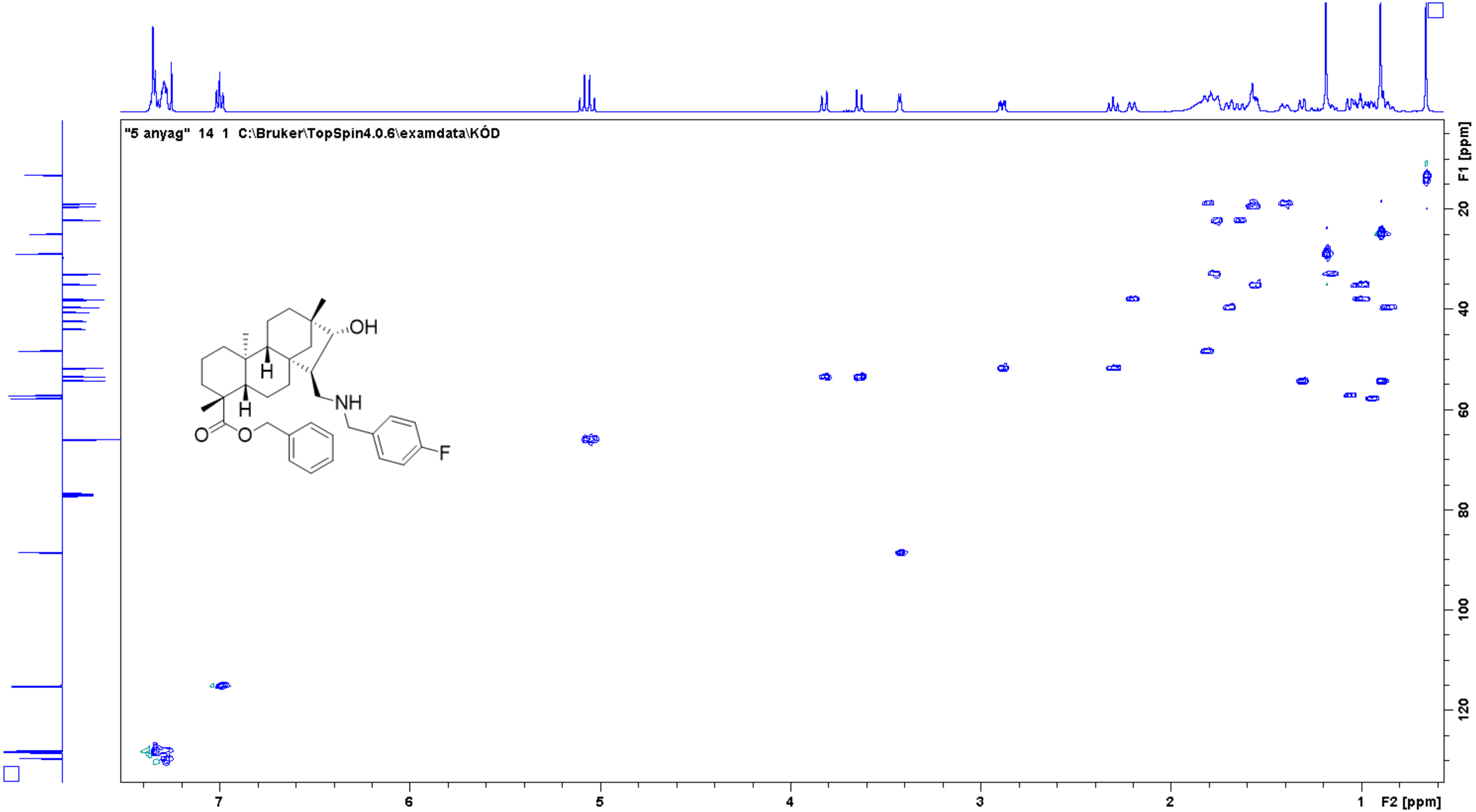

Figure S12

HMBC of compound (4*R*,4a*S*,6a*S*,7*R*,8*R*,9*S*,11b*S*)-Benzyl 7-(((4-fluorobenzyl)amino)methyl)-8-hydroxy-4,9,11b-trimethyltetradecahydro-6a,9-methanocyclohepta[a]naphthalene-4-carboxylate **(5)**:

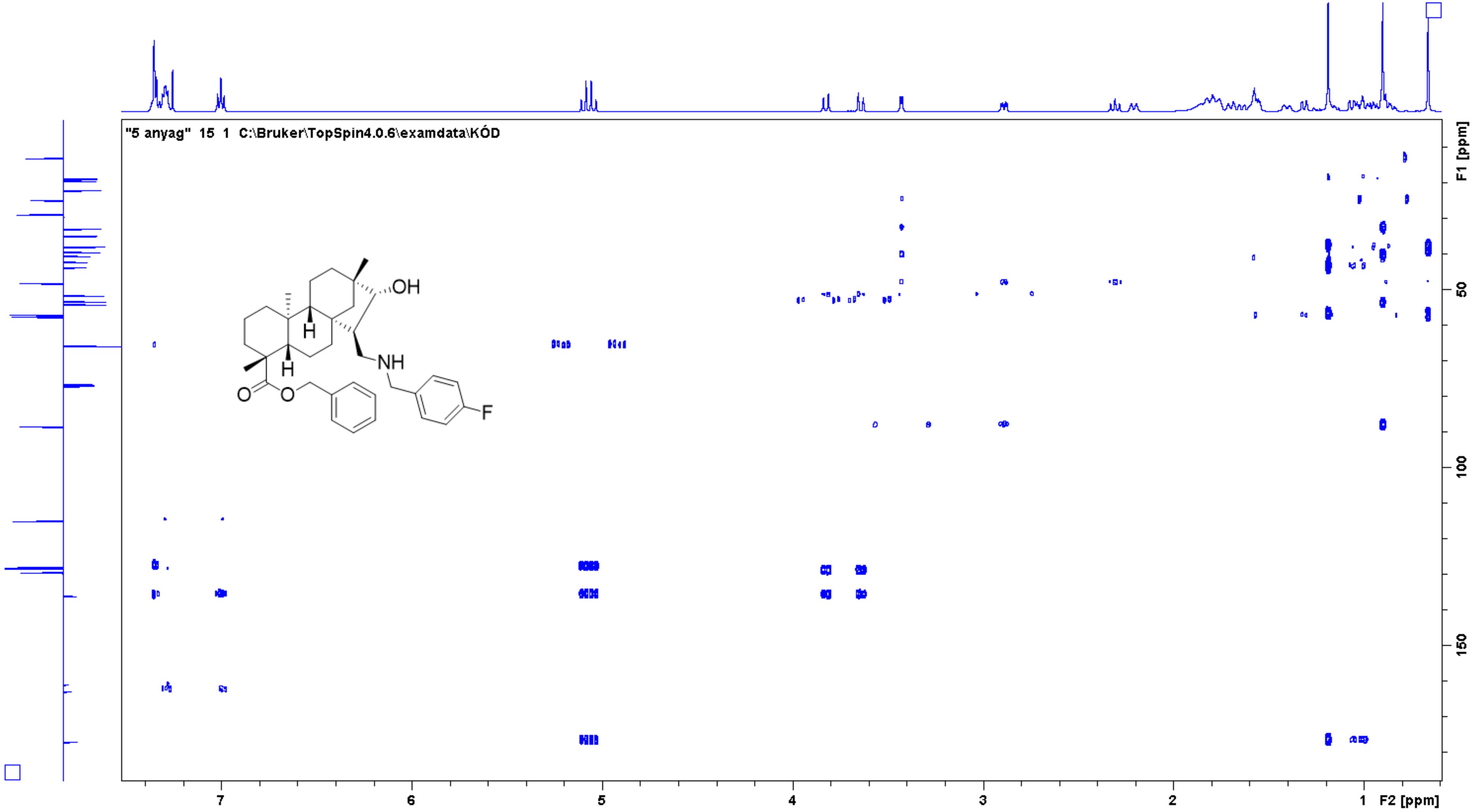

Figure S13

^{19}F of compound (4*R*,4a*S*,6a*S*,7*R*,8*R*,9*S*,11b*S*)-Benzyl 7-(((4-fluorobenzyl)amino)methyl)-8-hydroxy-4,9,11b-trimethyltetradecahydro-6a,9-methanocyclohepta[a]naphthalene-4-carboxylate **(5)**:

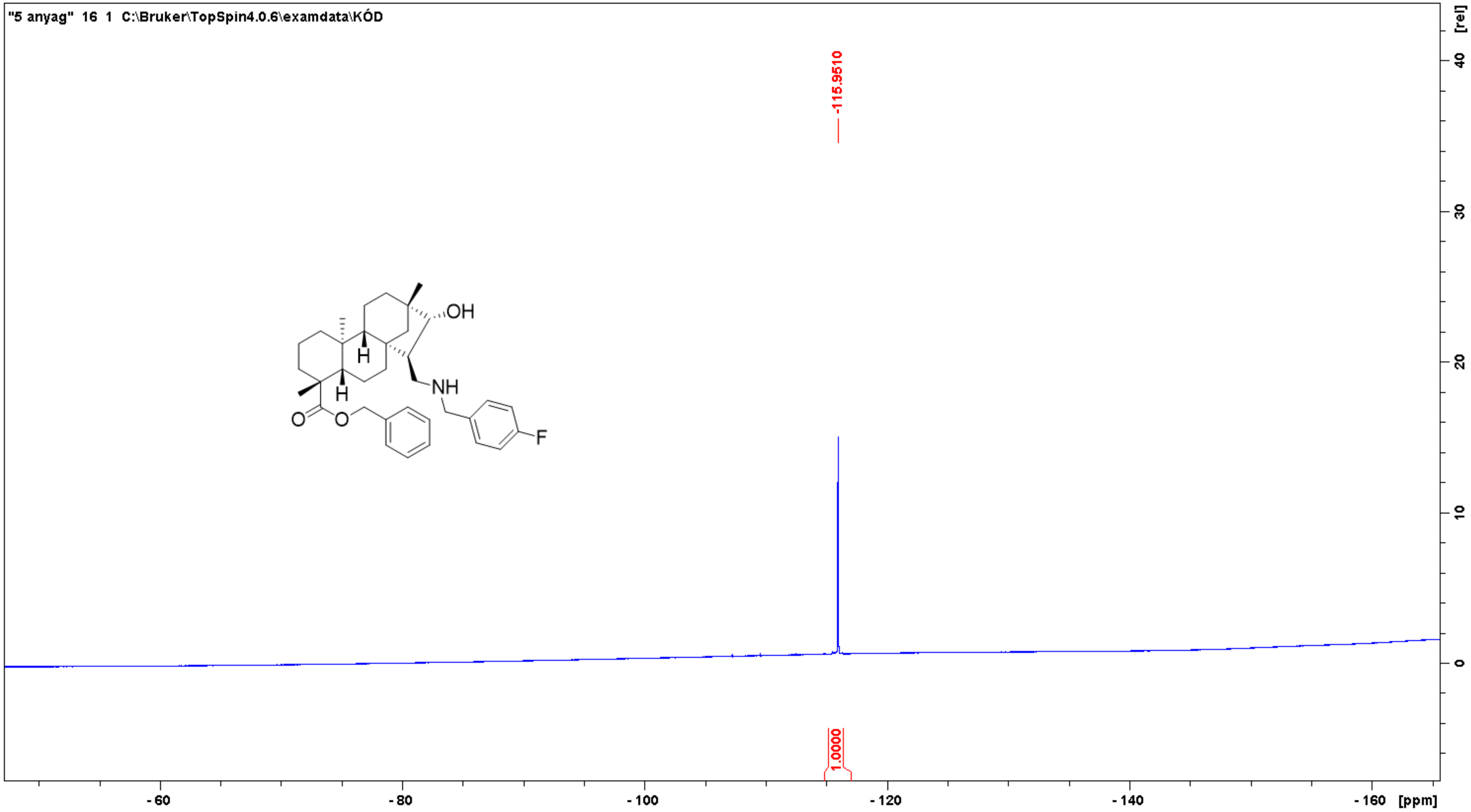

Figure S14

^{1}H-NMR of compound (4*R*,4a*S*,6aS,7*R*,8*R*,9*S*,11b*S*)-Benzyl 7-(((*tert*-butoxycarbonyl)(4-fluorobenzyl)amino)methyl)-8-hydroxy-4,9,11b-trimethyltetradecahydro-6a,9-methanocyclohepta[a]naphthalene-4-carboxylate **(6)**:

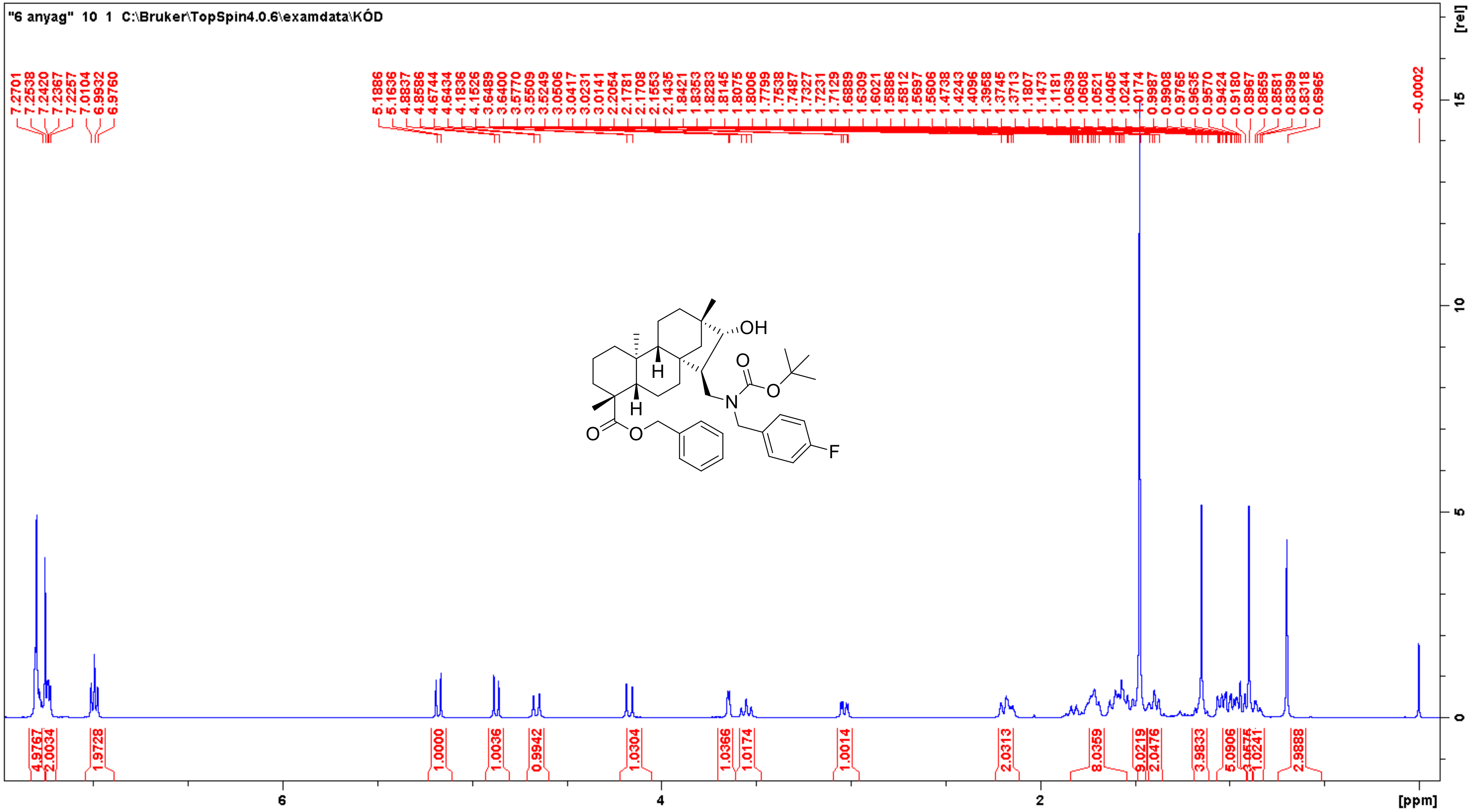

Figure S15

^{13}C-NMR of compound (4*R*,4a*S*,6aS,7*R*,8*R*,9*S*,11b*S*)-Benzyl 7-(((*tert*-butoxycarbonyl)(4-fluorobenzyl)amino)methyl)-8-hydroxy-4,9,11b-trimethyltetradecahydro-6a,9-methanocyclohepta[a]naphthalene-4-carboxylate **(6)**:

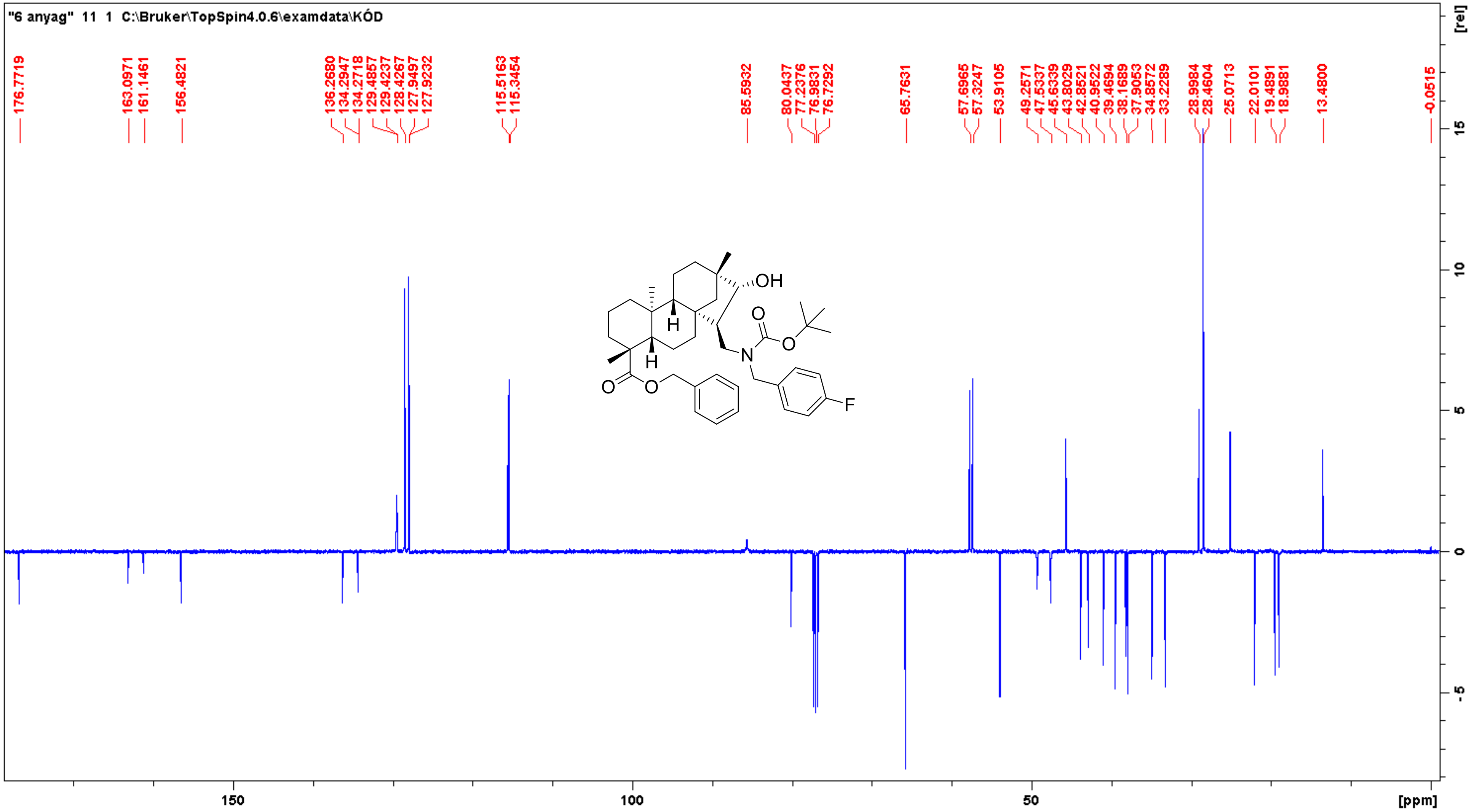

Figure S16

COSY of compound (4*R*,4a*S*,6aS,7*R*,8*R*,9*S*,11b*S*)-Benzyl 7-(((*tert*-butoxycarbonyl)(4-fluorobenzyl)amino)methyl)-8-hydroxy-4,9,11b-trimethyltetradecahydro-6a,9-methanocyclohepta[a]naphthalene-4-carboxylate **(6)**:

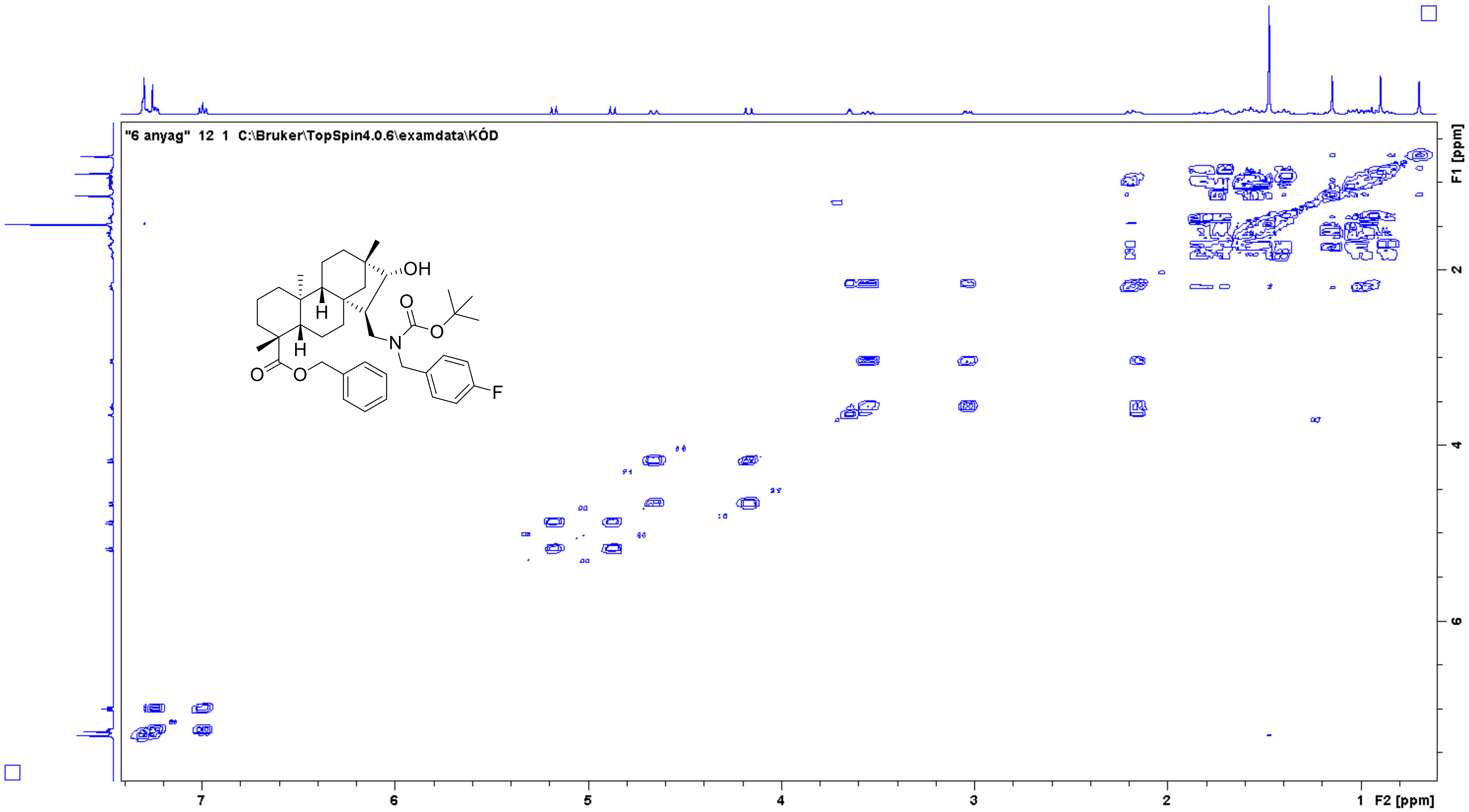

Figure S17

NOESY of compound (4*R*,4a*S*,6aS,7*R*,8*R*,9*S*,11b*S*)-Benzyl 7-(((*tert*-butoxycarbonyl)(4-fluorobenzyl)amino)methyl)-8-hydroxy-4,9,11b-trimethyltetradecahydro-6a,9-methanocyclohepta[a]naphthalene-4-carboxylate **(6)**:

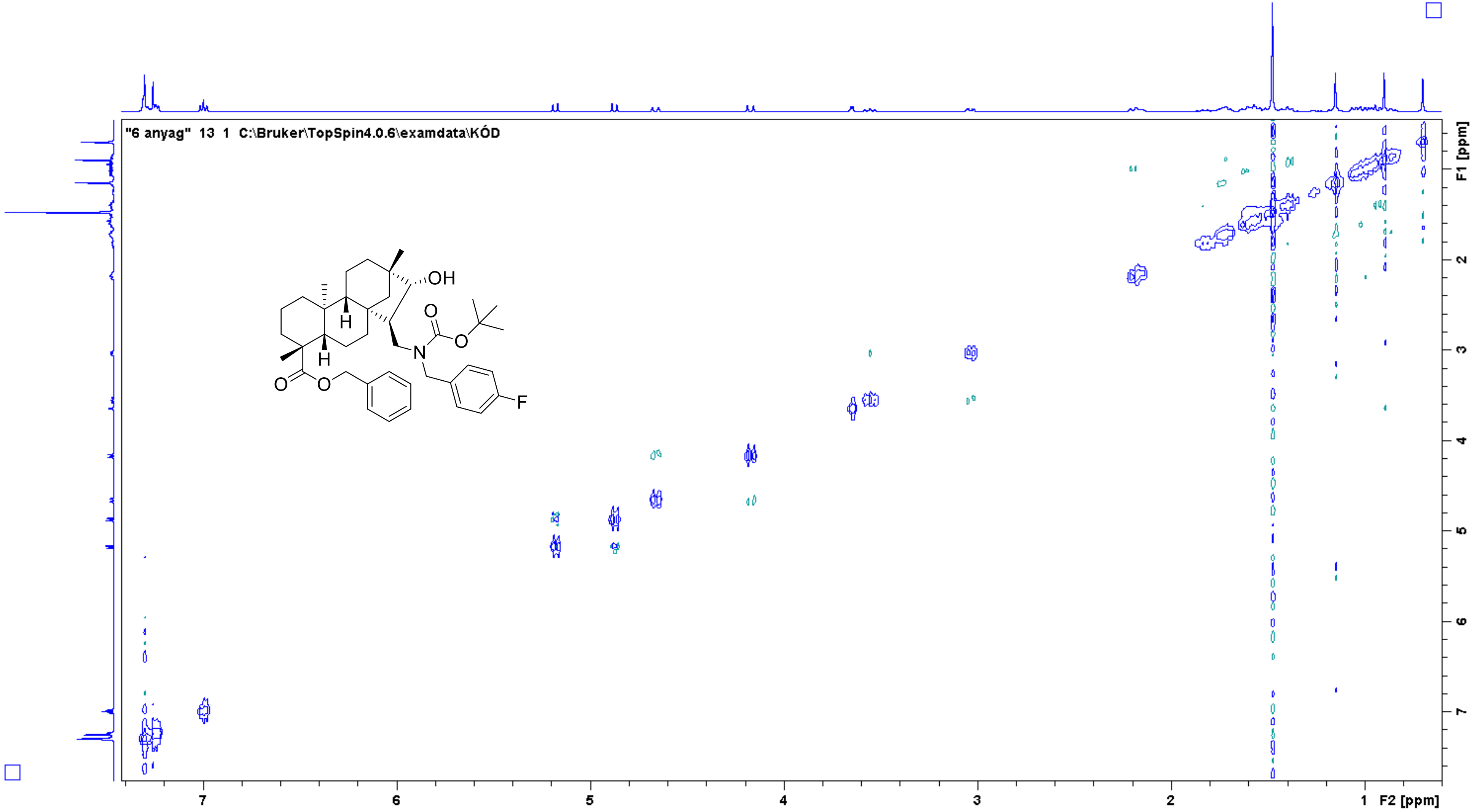

Figure S18

HSQC of compound (4*R*,4a*S*,6aS,7*R*,8*R*,9*S*,11b*S*)-Benzyl 7-(((*tert*-butoxycarbonyl)(4-fluorobenzyl)amino)methyl)-8-hydroxy-4,9,11b-trimethyltetradecahydro-6a,9-methanocyclohepta[a]naphthalene-4-carboxylate **(6)**:

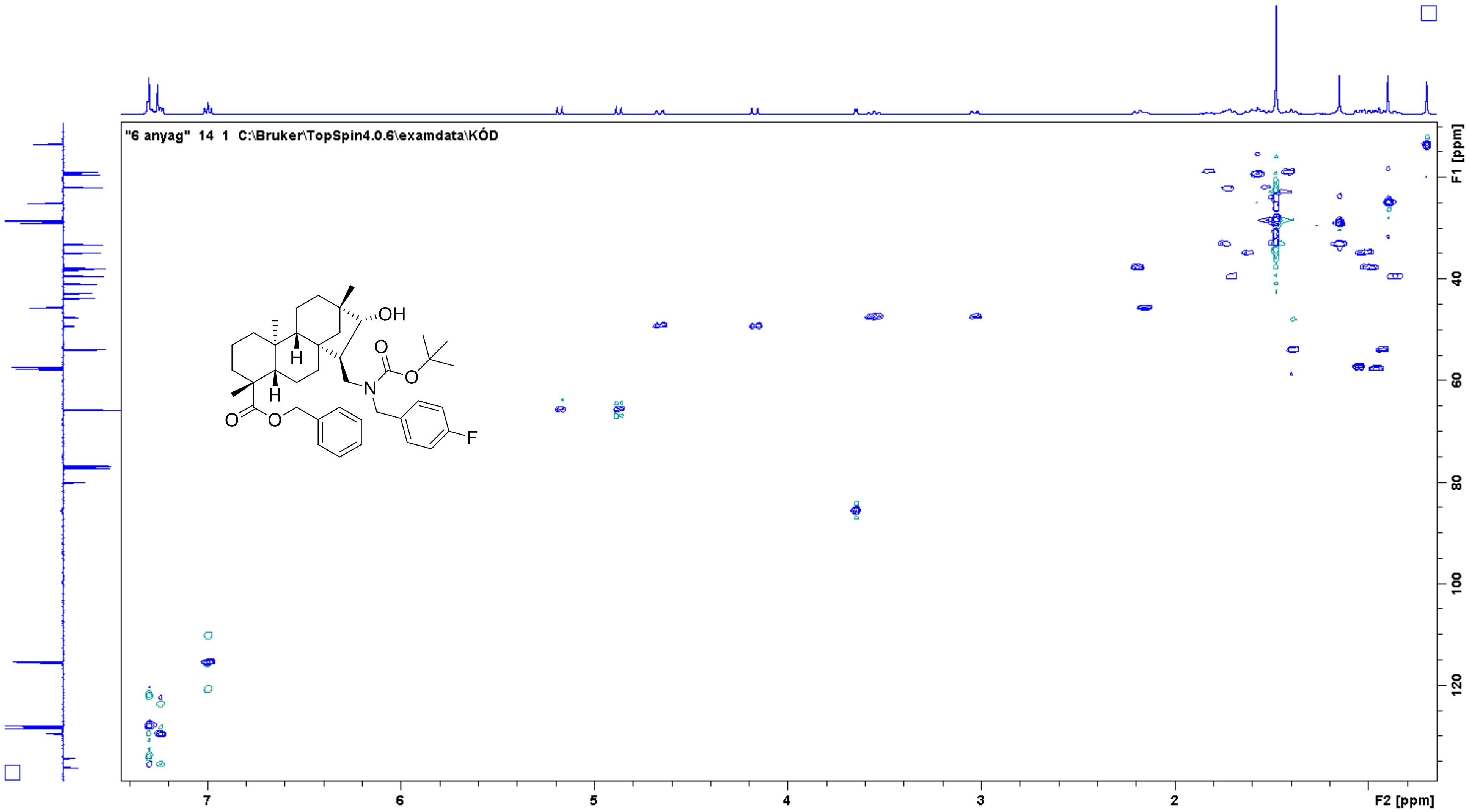

Figure S19

HMBC of compound (4*R*,4a*S*,6aS,7*R*,8*R*,9*S*,11b*S*)-Benzyl 7-(((*tert*-butoxycarbonyl)(4-fluorobenzyl)amino)methyl)-8-hydroxy-4,9,11b-trimethyltetradecahydro-6a,9-methanocyclohepta[a]naphthalene-4-carboxylate **(6)**:

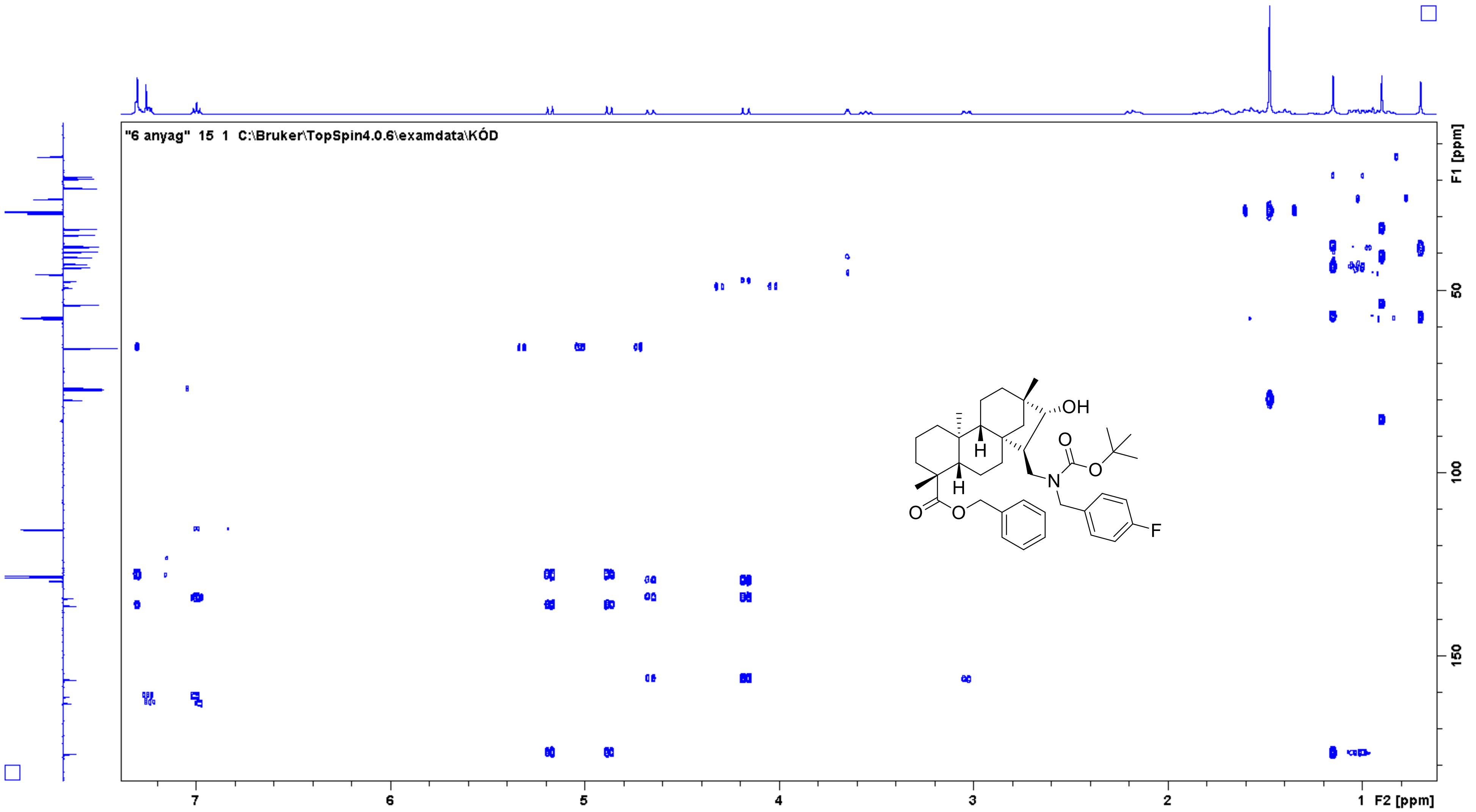

Figure S20

^{19}F-NMR of compound (4*R*,4a*S*,6aS,7*R*,8*R*,9*S*,11b*S*)-Benzyl 7-(((*tert*-butoxycarbonyl)(4-fluorobenzyl)amino)methyl)-8-hydroxy-4,9,11b-trimethyltetradecahydro-6a,9-methanocyclohepta[a]naphthalene-4-carboxylate **(6)**:

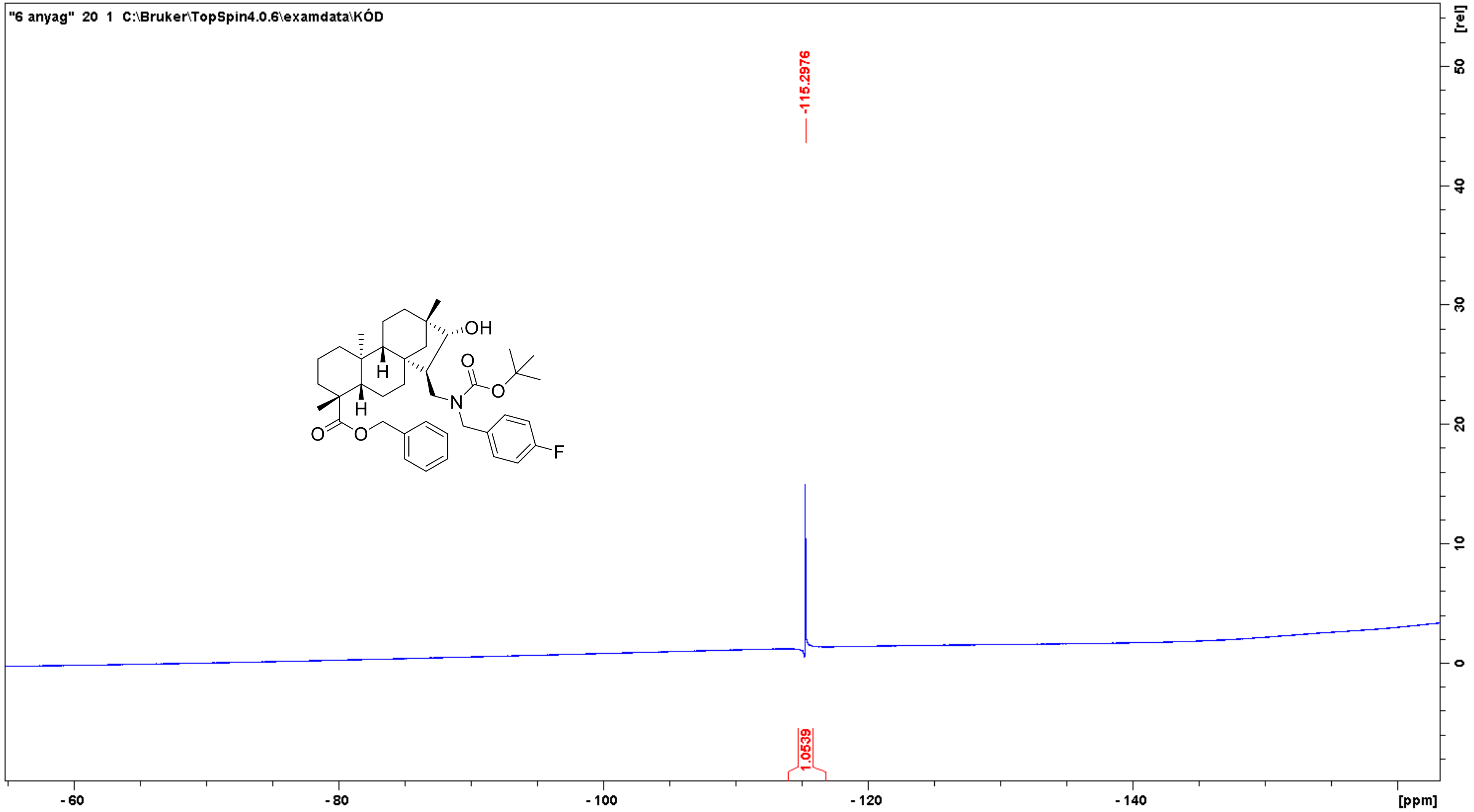

Figure S21

^{1}H-NMR of compound (4*R*,4a*S*,6a*S*,7*R*,8*R*,9*S*,11b*S*)-7-(((*Tert*-butoxycarbonyl)(4-fluorobenzyl)amino)methyl)-8-hydroxy-4,9,11b-trimethyltetradecahydro-6a,9-methanocyclohepta[a]naphthalene-4-carboxylic acid **(7)**:

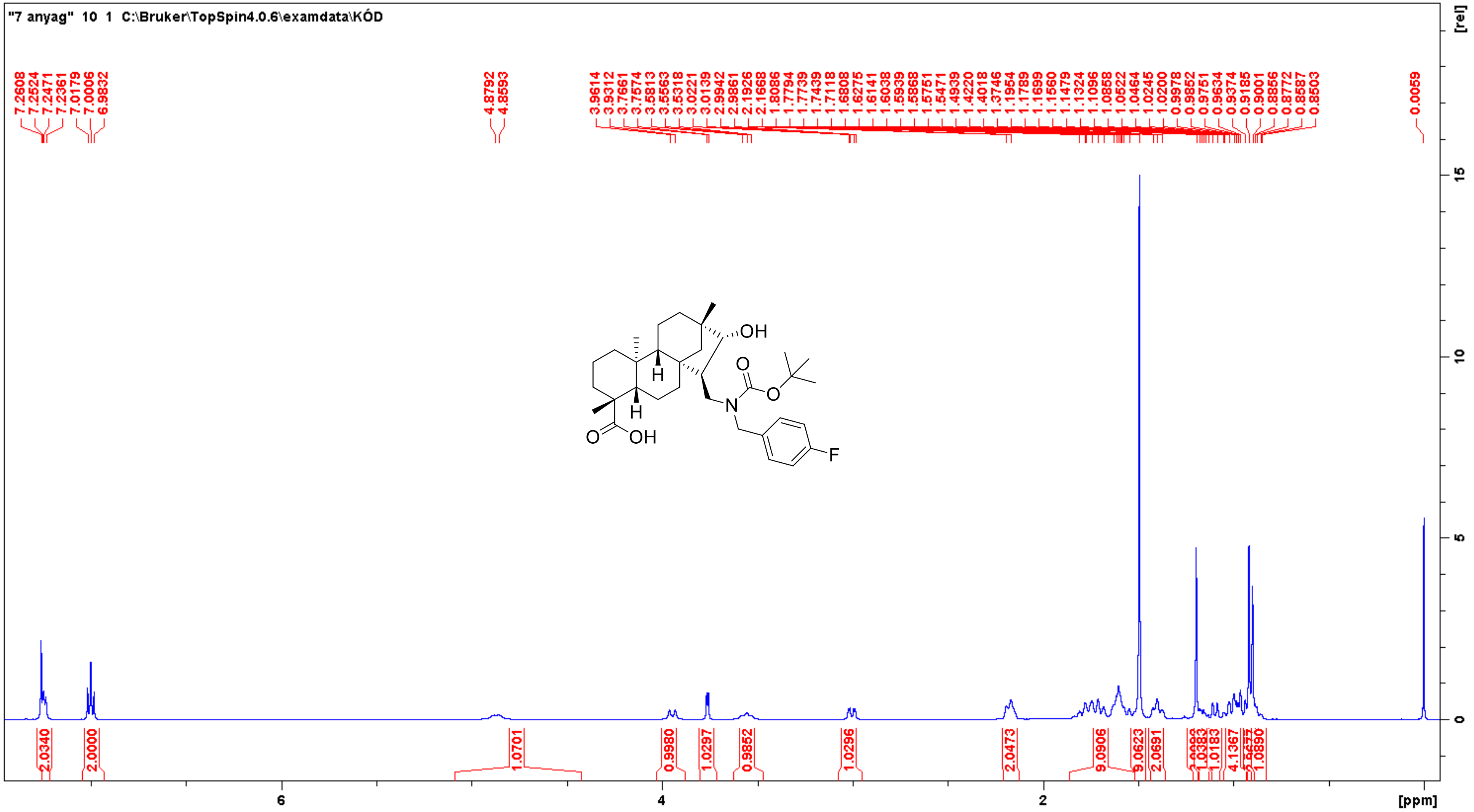

Figure S22

[2]13C-NMR of compound (4*R*,4a*S*,6a*S*,7*R*,8*R*,9*S*,11b*S*)-7-(((*Tert*-butoxycarbonyl)(4-fluorobenzyl)amino)methyl)-8-hydroxy-4,9,11b-trimethyltetradecahydro-6a,9-methanocyclohepta[a]naphthalene-4-carboxylic acid **(7)**:

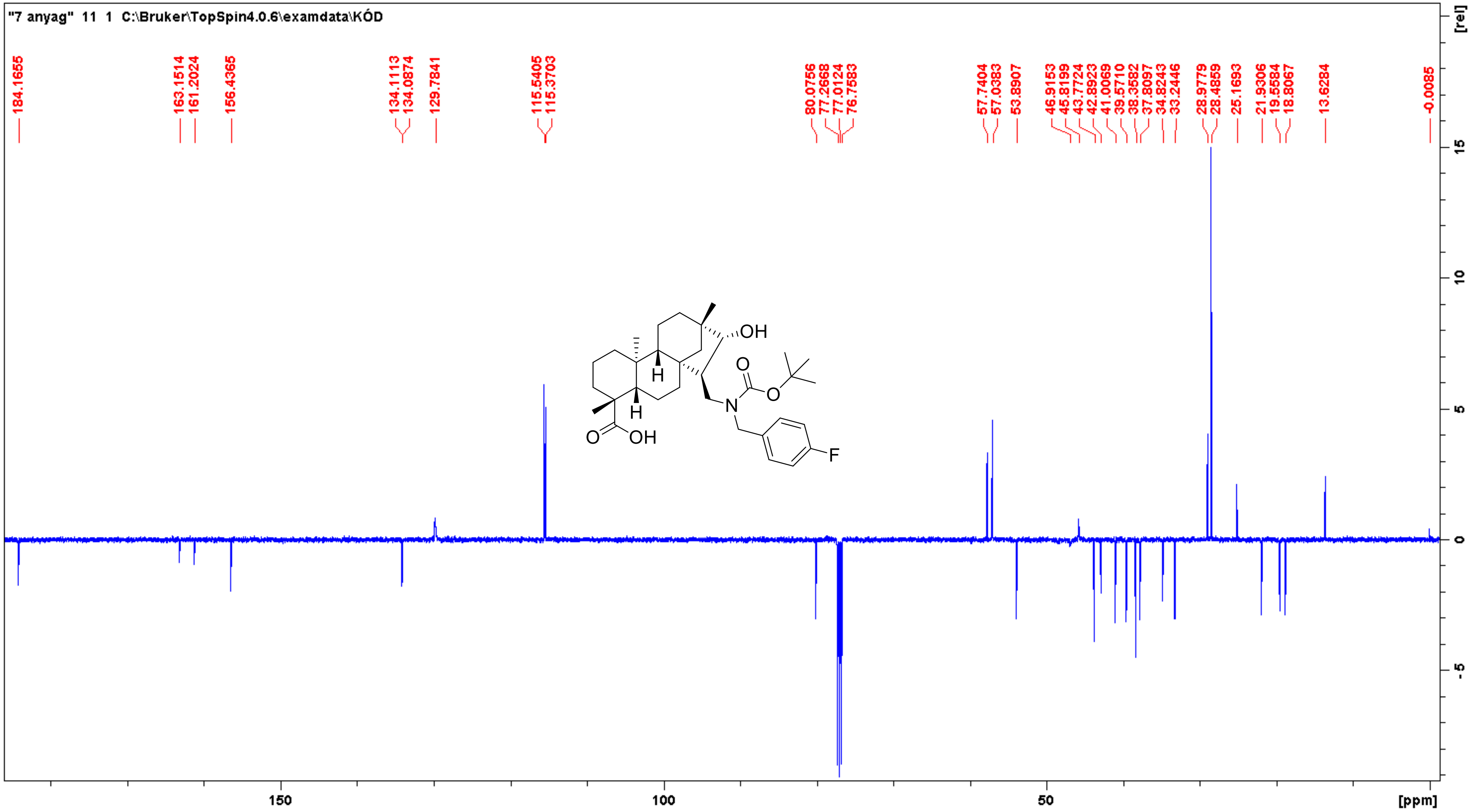

Figure S23

COSY of compound (4*R*,4a*S*,6a*S*,7*R*,8*R*,9*S*,11b*S*)-7-(((*Tert*-butoxycarbonyl)(4-fluorobenzyl)amino)methyl)-8-hydroxy-4,9,11b-trimethyltetradecahydro-6a,9-methanocyclohepta[a]naphthalene-4-carboxylic acid **(7)**:

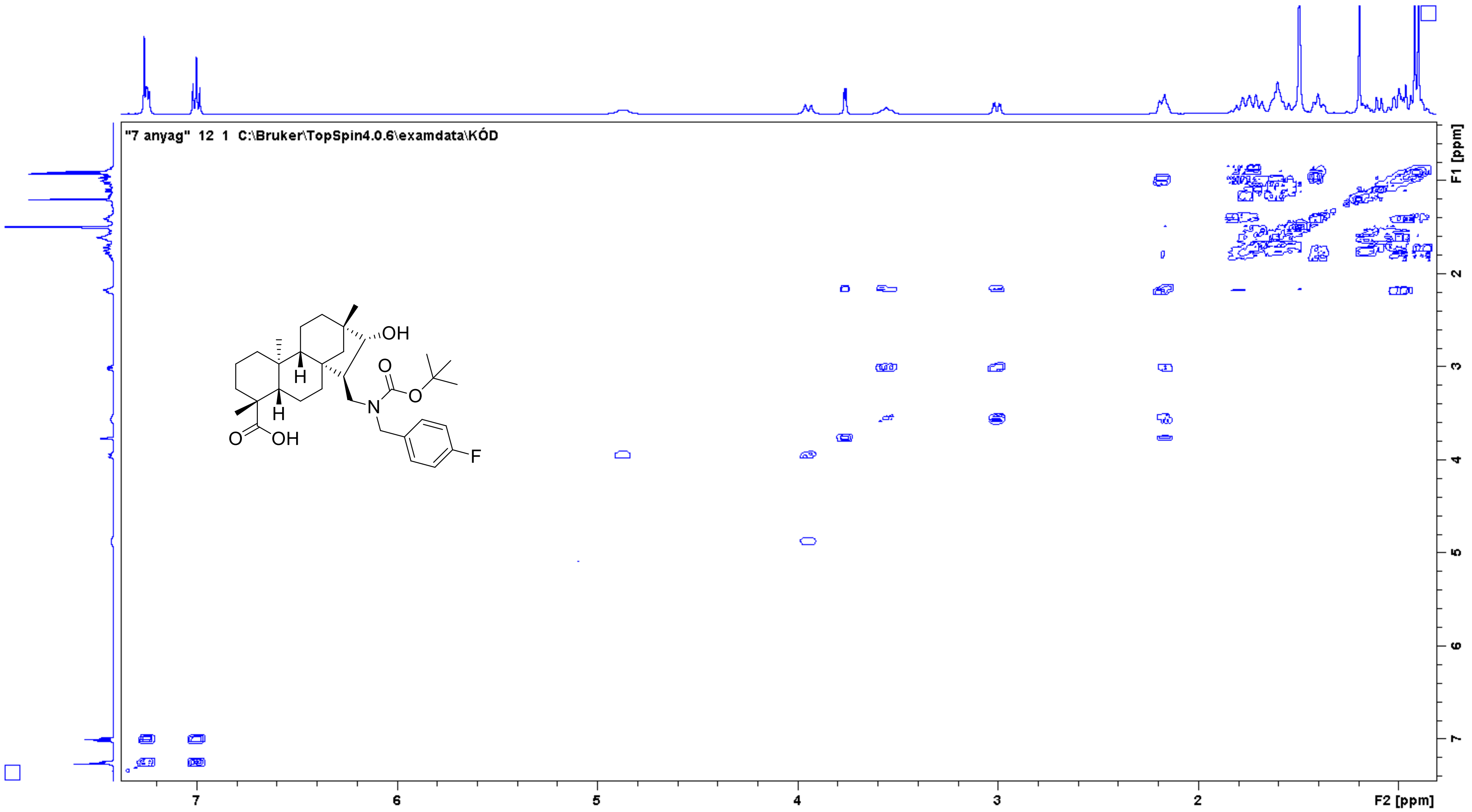

Figure S24

HSQC of compound (4*R*,4a*S*,6a*S*,7*R*,8*R*,9*S*,11b*S*)-7-(((*Tert*-butoxycarbonyl)(4-fluorobenzyl)amino)methyl)-8-hydroxy-4,9,11b-trimethyltetradecahydro-6a,9-methanocyclohepta[a]naphthalene-4-carboxylic acid **(7)**:

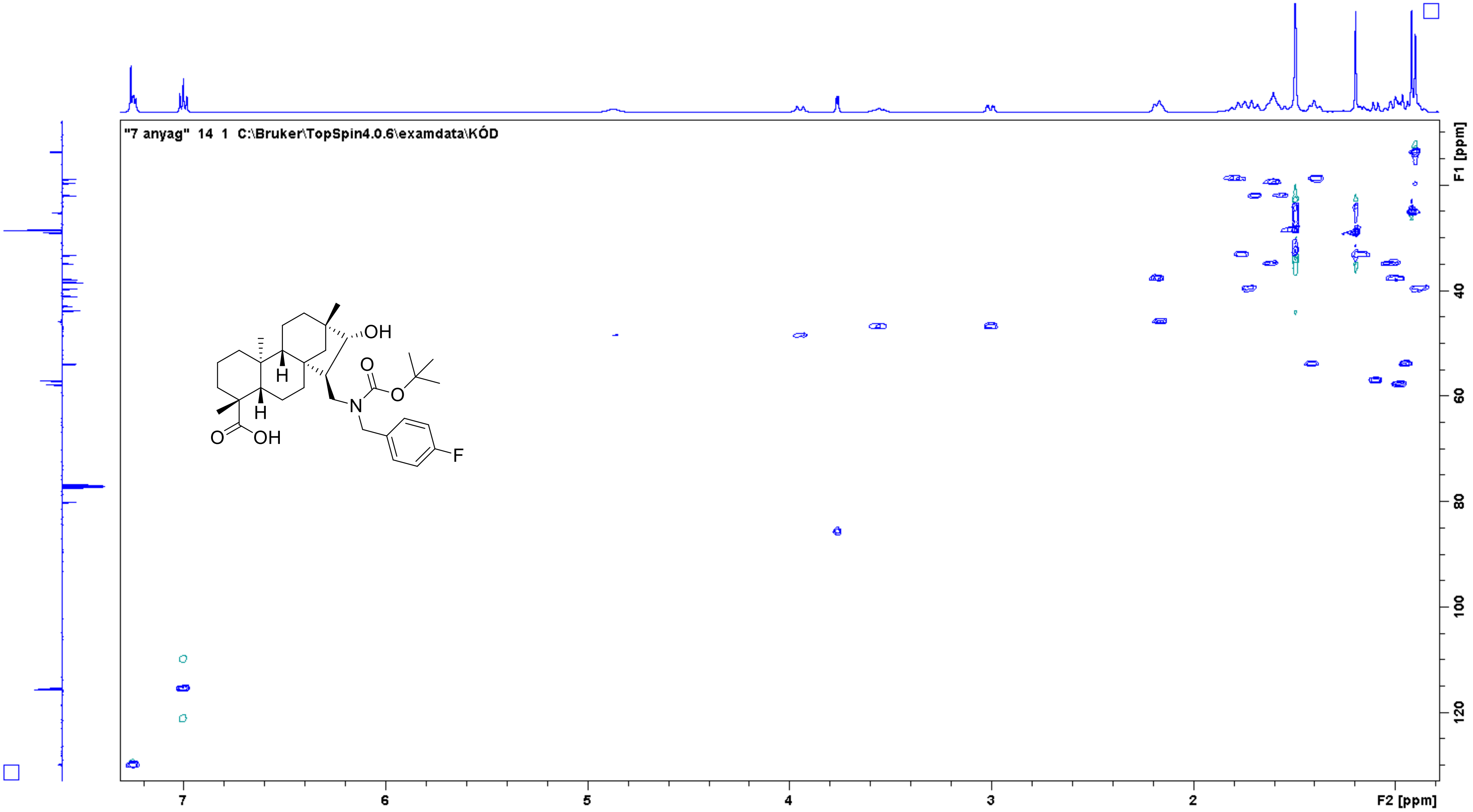

Figure S25

HMBC of compound (4*R*,4a*S*,6a*S*,7*R*,8*R*,9*S*,11b*S*)-7-(((*Tert*-butoxycarbonyl)(4-fluorobenzyl)amino)methyl)-8-hydroxy-4,9,11b-trimethyltetradecahydro-6a,9-methanocyclohepta[a]naphthalene-4-carboxylic acid **(7)**:

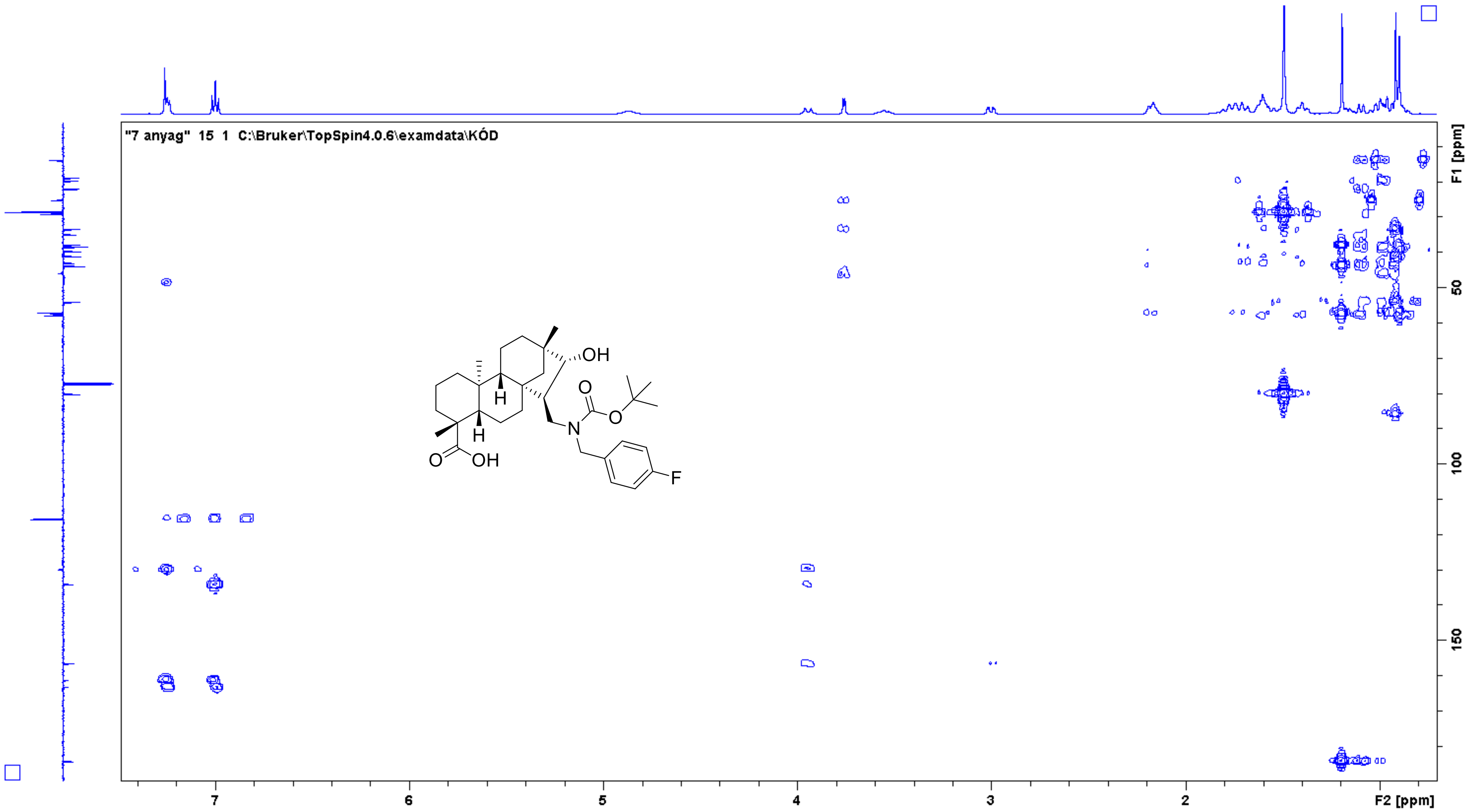

Figure S26

^{19}F-NMR of compound (4*R*,4a*S*,6a*S*,7*R*,8*R*,9*S*,11b*S*)-7-(((*Tert*-butoxycarbonyl)(4-fluorobenzyl)amino)methyl)-8-hydroxy-4,9,11b-trimethyltetradecahydro-6a,9-methanocyclohepta[a]naphthalene-4-carboxylic acid **(7)**:

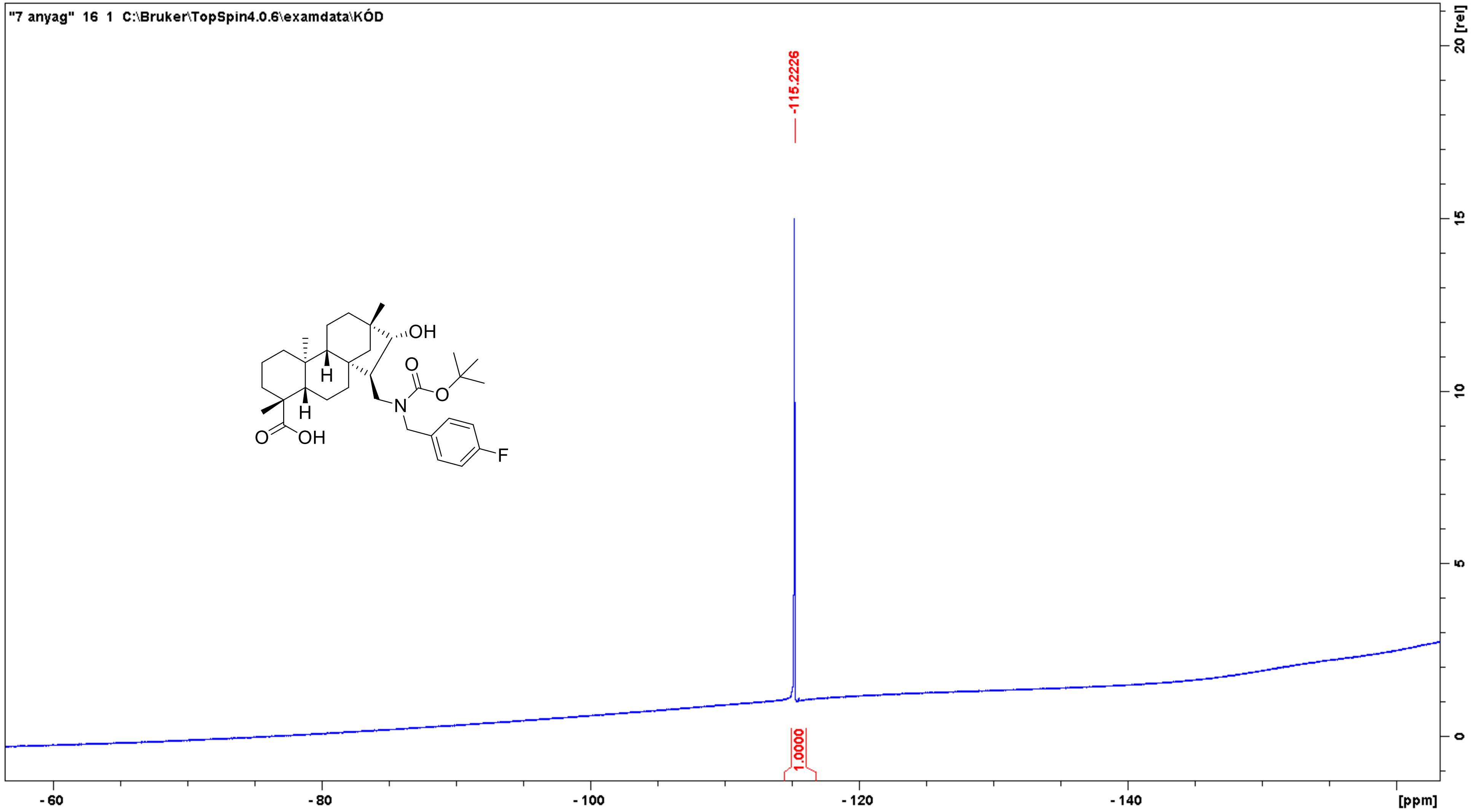

Figure S27

^{1}H-NMR of compound (4*R*,4a*S*,6a*S*,7*R*,8*R*,9*S*,11b*S*)-7-(((4-Fluorobenzyl)amino)methyl)-8-hydroxy-4,9,11b-trimethyltetradecahydro-6a,9-methanocyclohepta[a]naphthalene-4-carboxylic acid **(8)**:

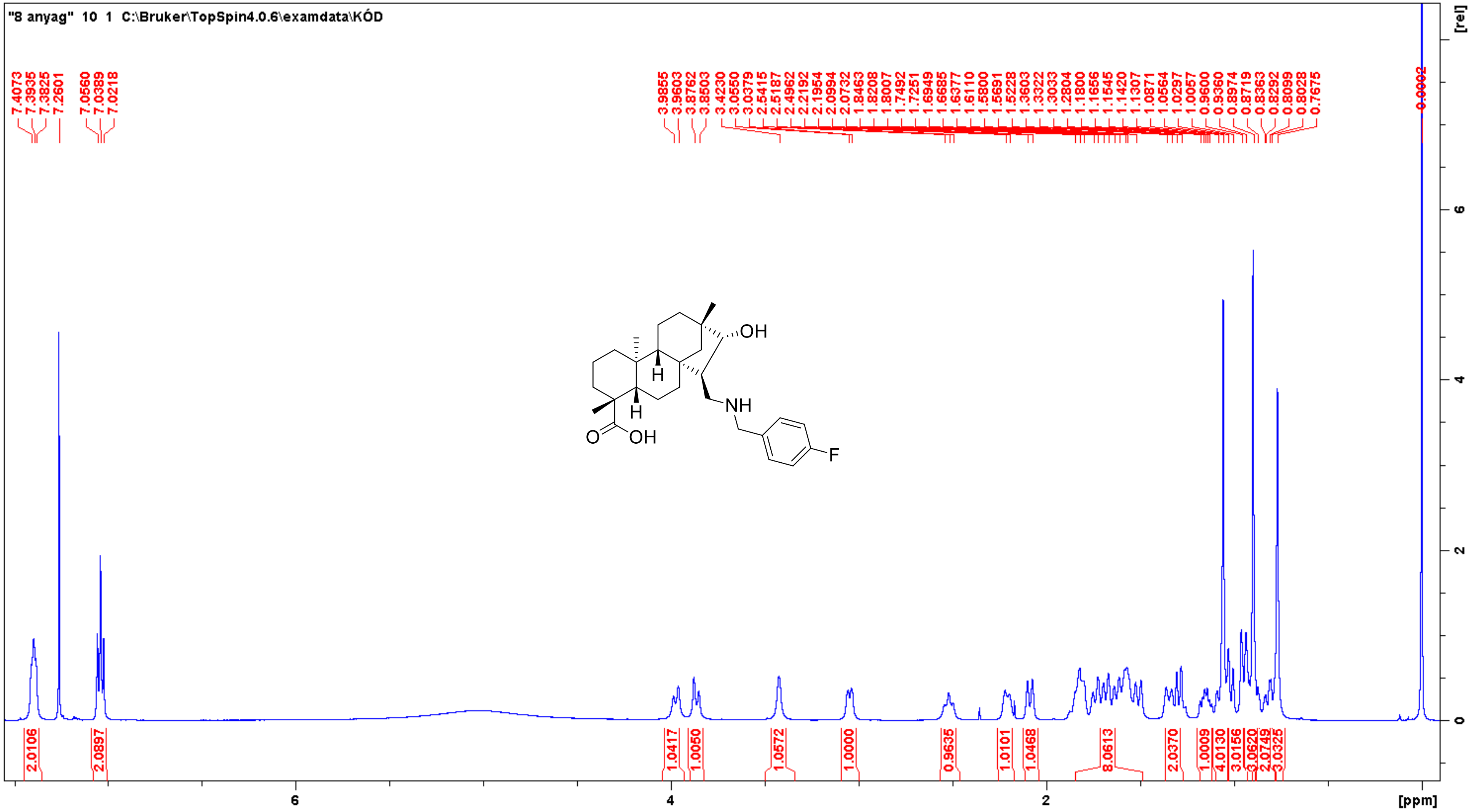

Figure S28

^{13}C-NMR of compound (4*R*,4a*S*,6a*S*,7*R*,8*R*,9*S*,11b*S*)-7-(((4-Fluorobenzyl)amino)methyl)-8-hydroxy-4,9,11b-trimethyltetradecahydro-6a,9-methanocyclohepta[a]naphthalene-4-carboxylic acid **(8)**:

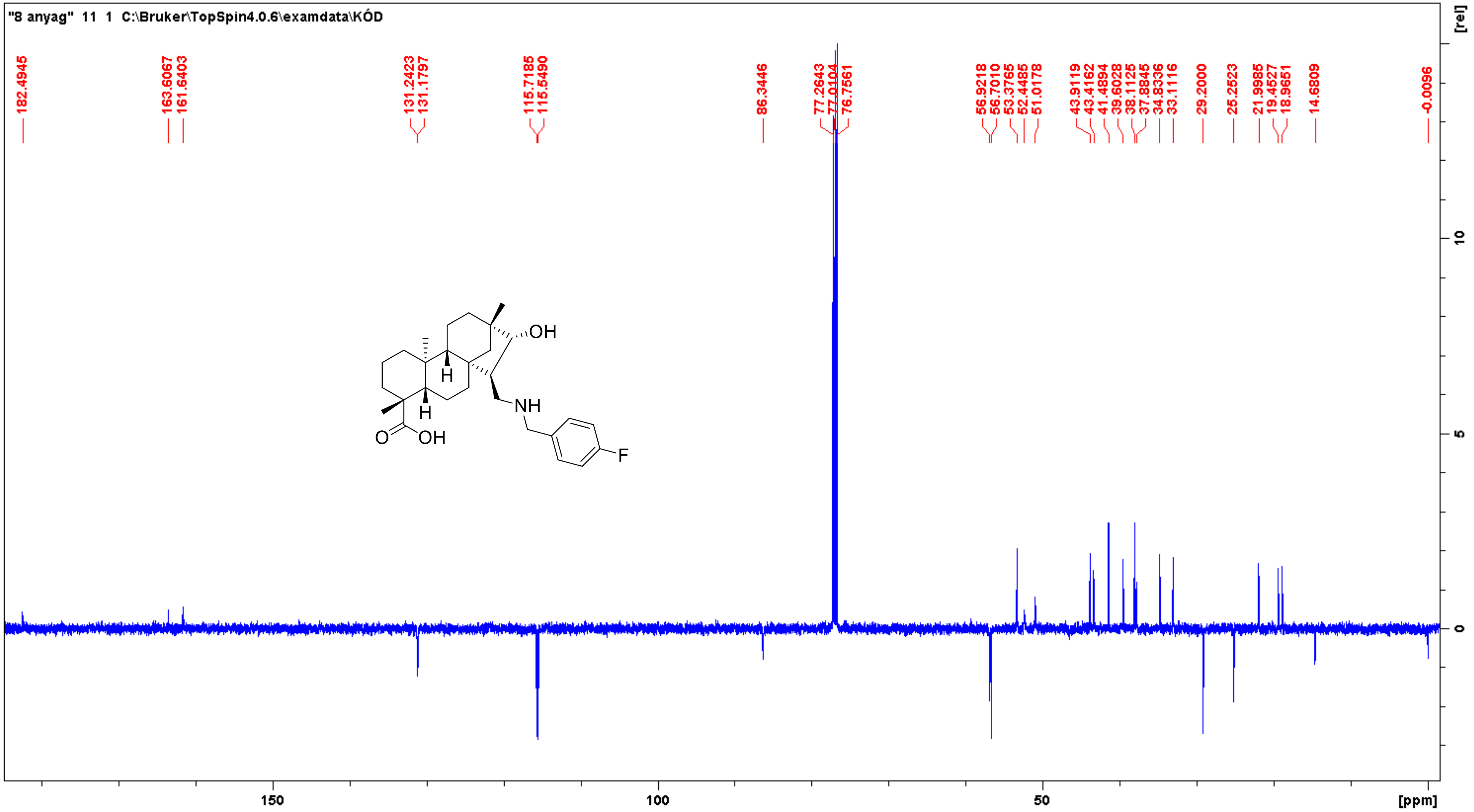

Figure S29

COSY of compound (4*R*,4a*S*,6a*S*,7*R*,8*R*,9*S*,11b*S*)-7-(((4-Fluorobenzyl)amino)methyl)-8-hydroxy-4,9,11b-trimethyltetradecahydro-6a,9-methanocyclohepta[a]naphthalene-4-carboxylic acid **(8)**:

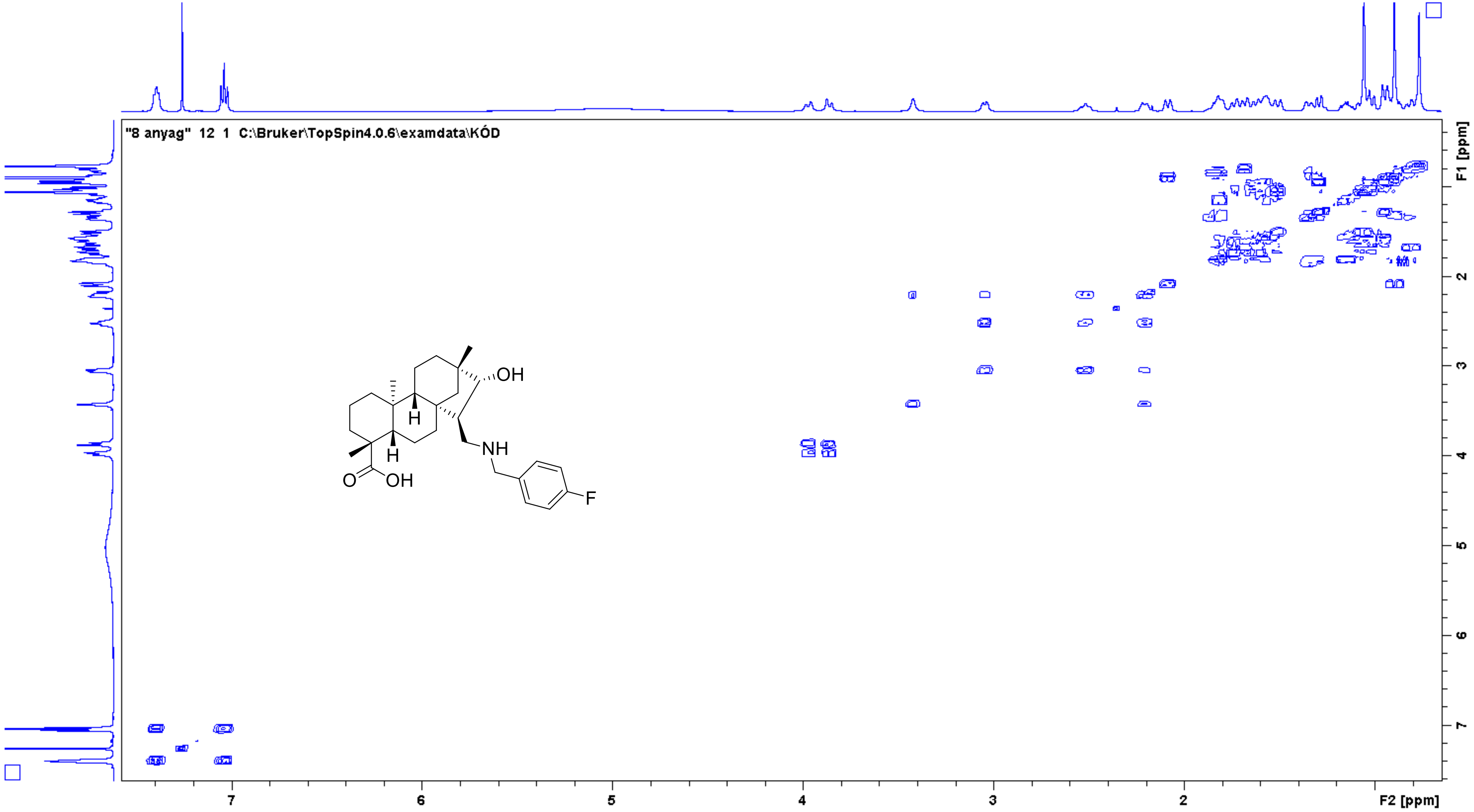

Figure S30

NOESY of compound (4*R*,4a*S*,6a*S*,7*R*,8*R*,9*S*,11b*S*)-7-(((4-Fluorobenzyl)amino)methyl)-8-hydroxy-4,9,11b-trimethyltetradecahydro-6a,9-methanocyclohepta[a]naphthalene-4-carboxylic acid **(8)**:

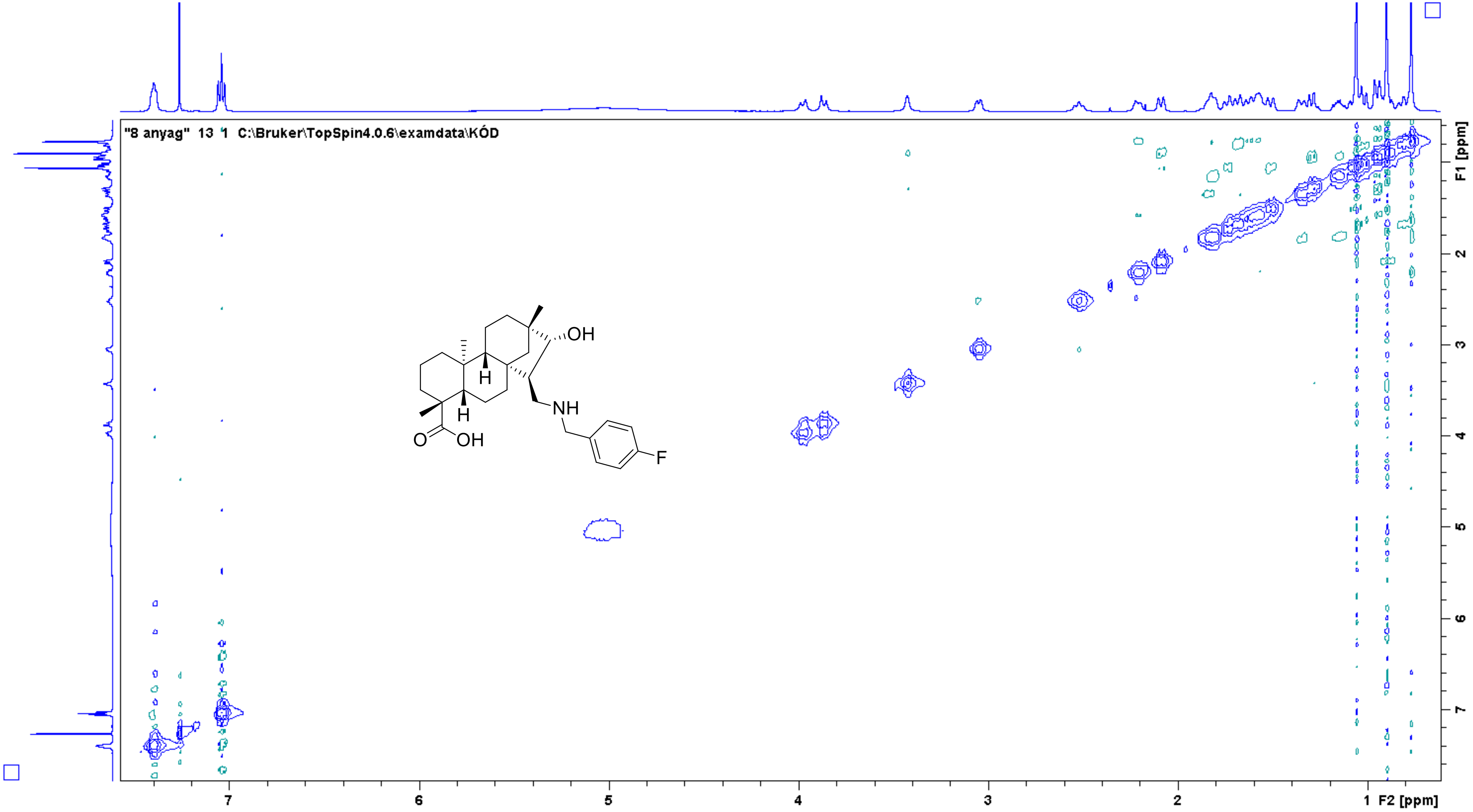

Figure S31

HSQC of compound (4*R*,4a*S*,6a*S*,7*R*,8*R*,9*S*,11b*S*)-7-(((4-Fluorobenzyl)amino)methyl)-8-hydroxy-4,9,11b-trimethyltetradecahydro-6a,9-methanocyclohepta[a]naphthalene-4-carboxylic acid **(8)**:

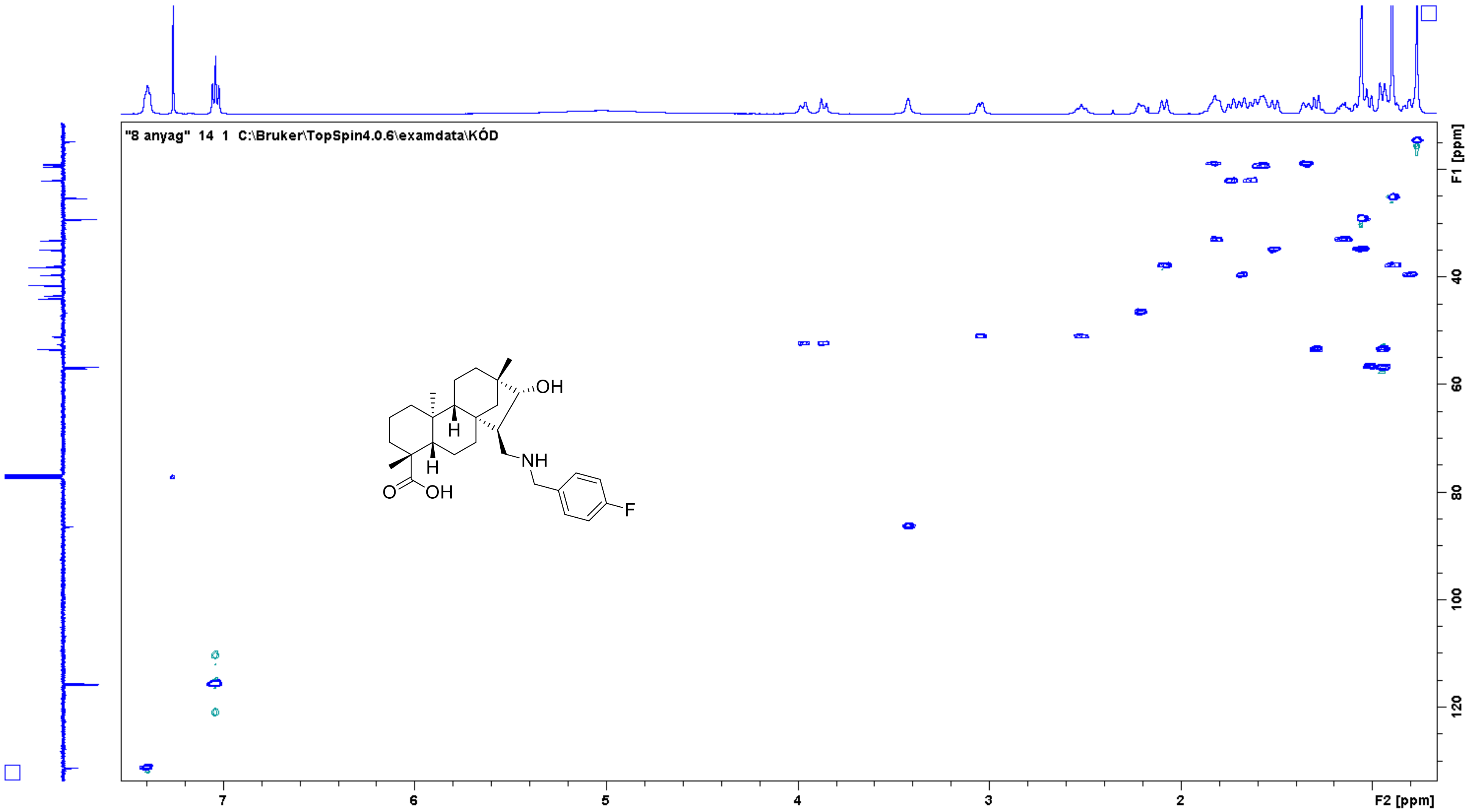

Figure S32

HMBC of compound (4*R*,4a*S*,6a*S*,7*R*,8*R*,9*S*,11b*S*)-7-(((4-Fluorobenzyl)amino)methyl)-8-hydroxy-4,9,11b-trimethyltetradecahydro-6a,9-methanocyclohepta[a]naphthalene-4-carboxylic acid **(8)**:

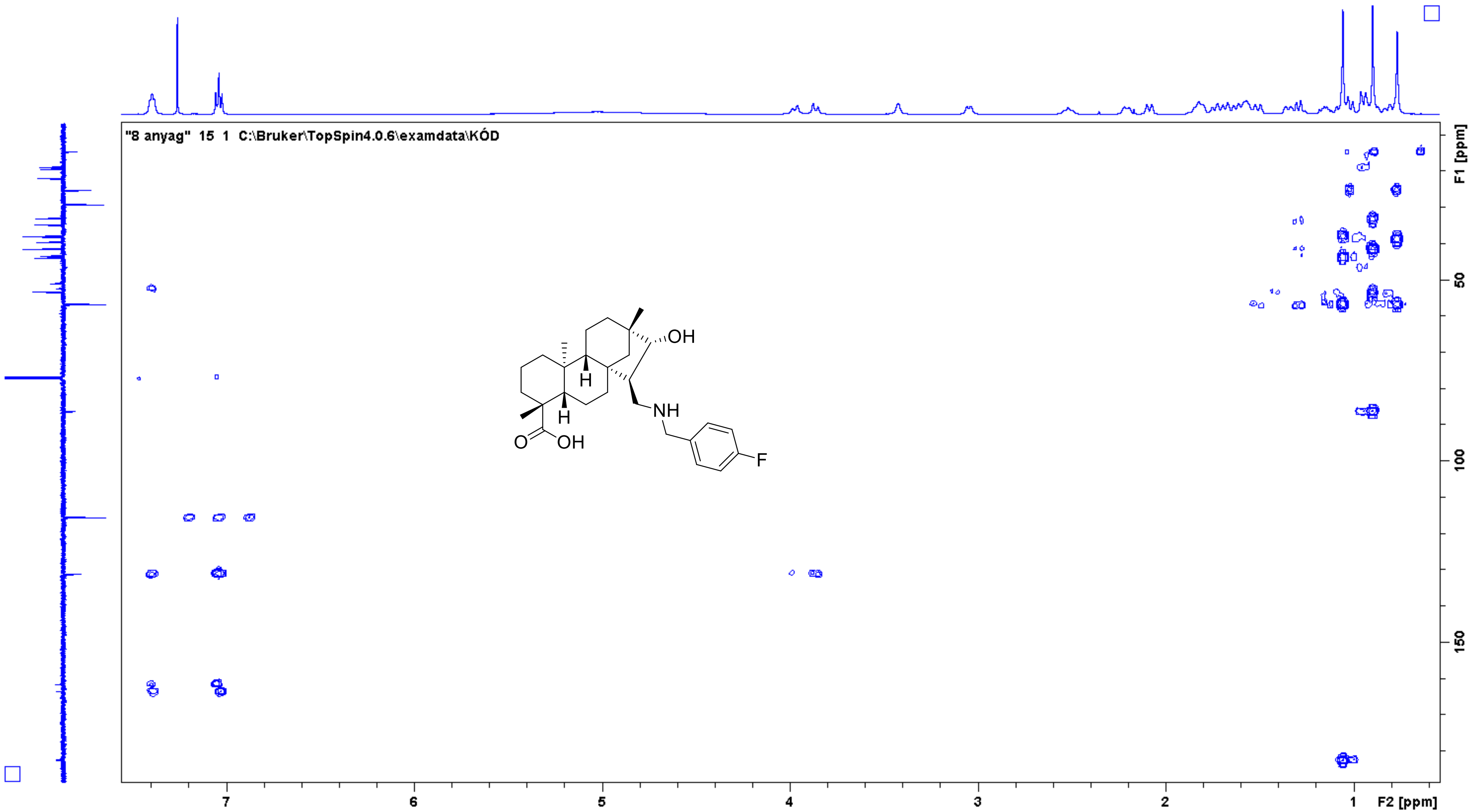

Figure S33

^{19}F-NMR of compound (4*R*,4a*S*,6a*S*,7*R*,8*R*,9*S*,11b*S*)-7-(((4-Fluorobenzyl)amino)methyl)-8-hydroxy-4,9,11b-trimethyltetradecahydro-6a,9-methanocyclohepta[a]naphthalene-4-carboxylic acid **(8)**:

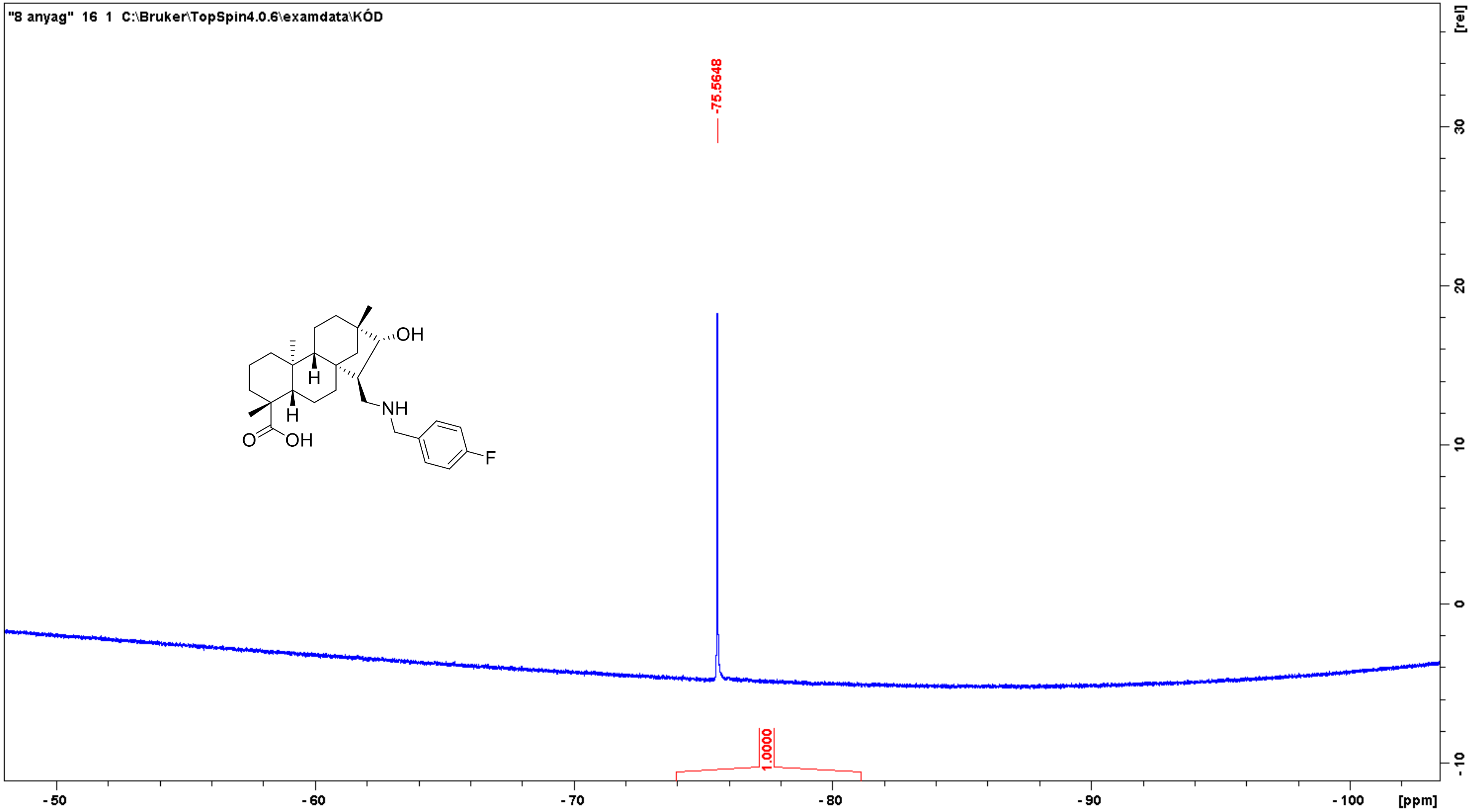

Figure S34

^{1}H-NMR of compound 3-(4-Bromobutoxy)-3-oxopropyl acrylate **(10)**:

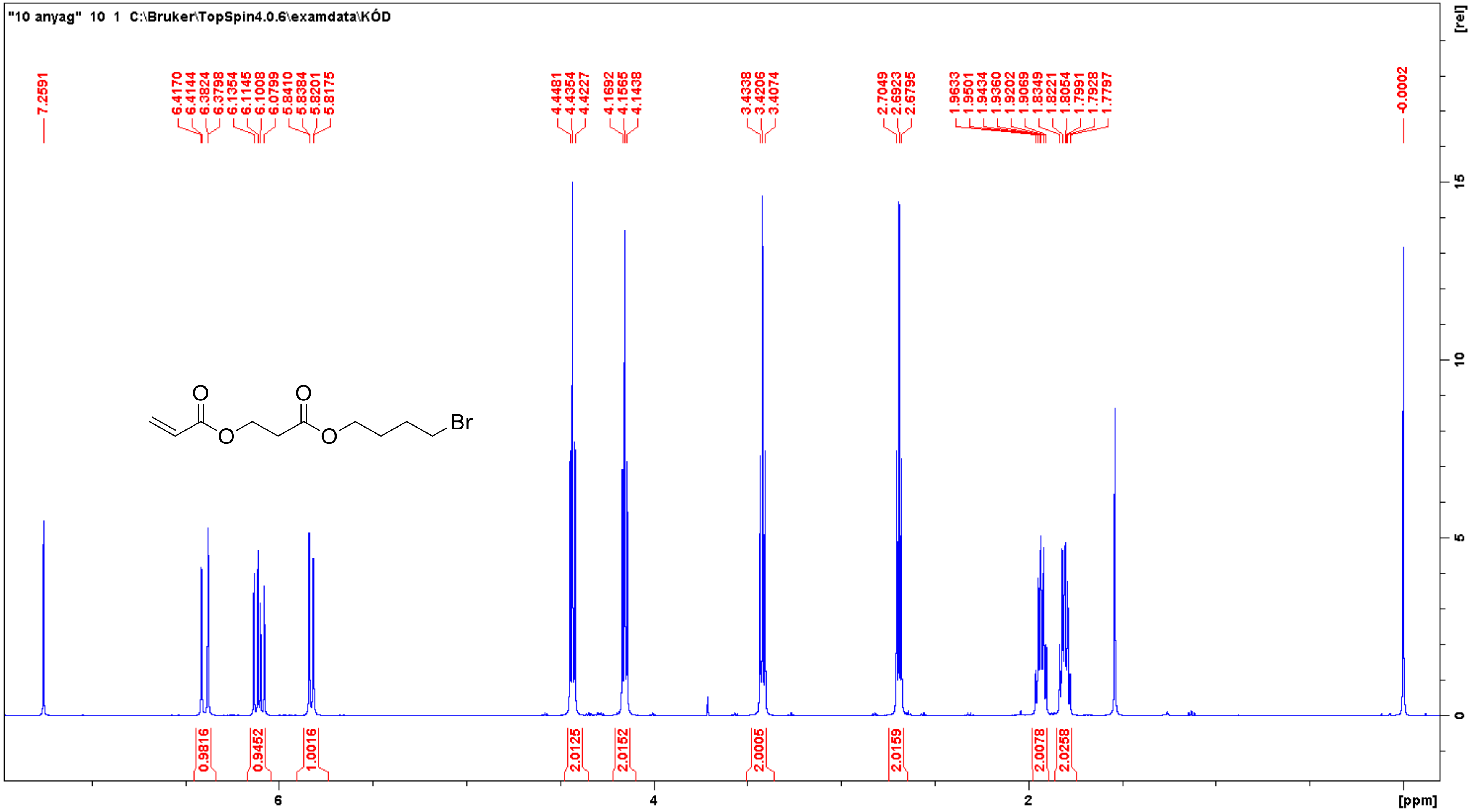

Figure S35

^{13}C-NMR of compound 3-(4-Bromobutoxy)-3-oxopropyl acrylate **(10)**:

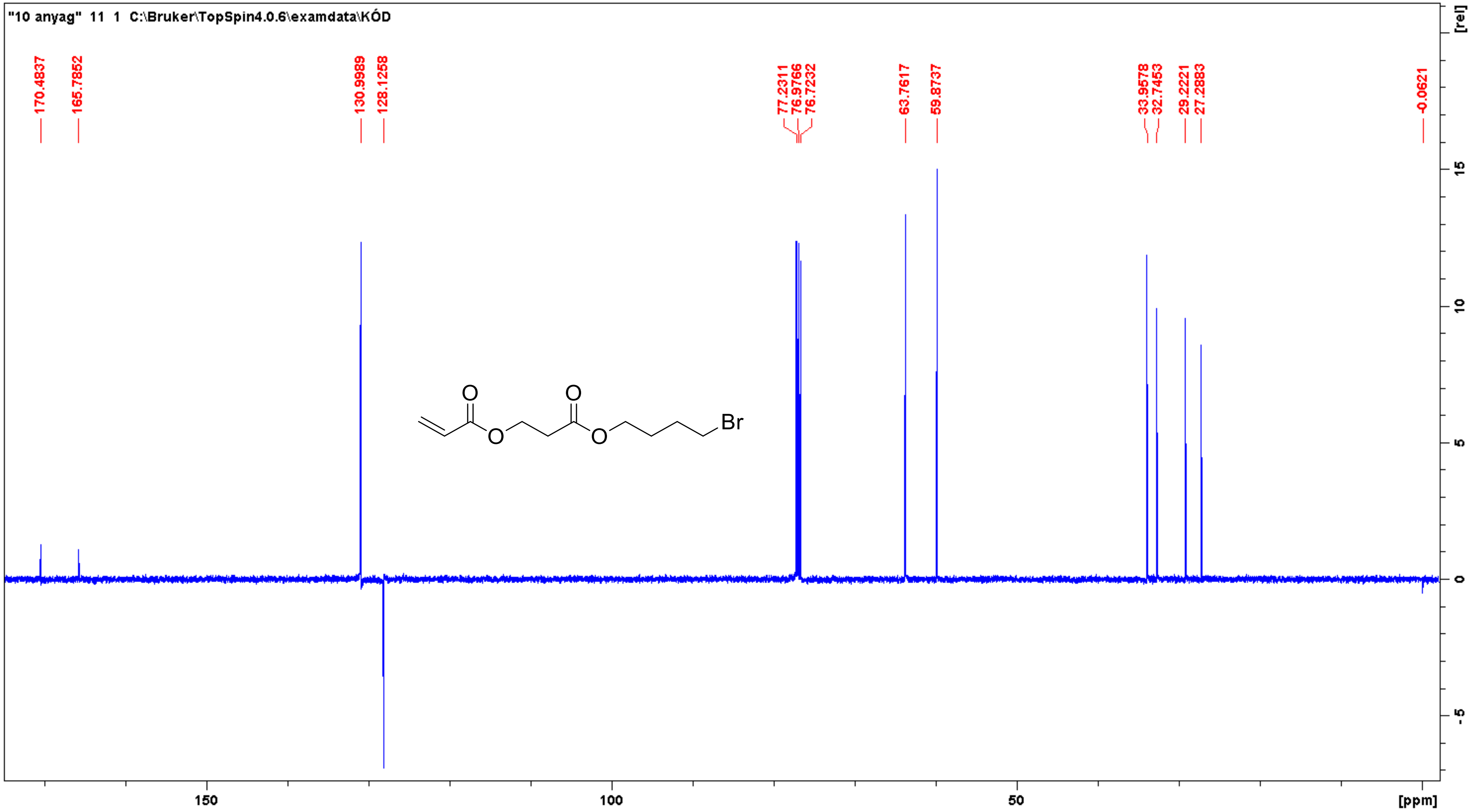

Figure S36

COSY of compound 3-(4-Bromobutoxy)-3-oxopropyl acrylate **(10)**:

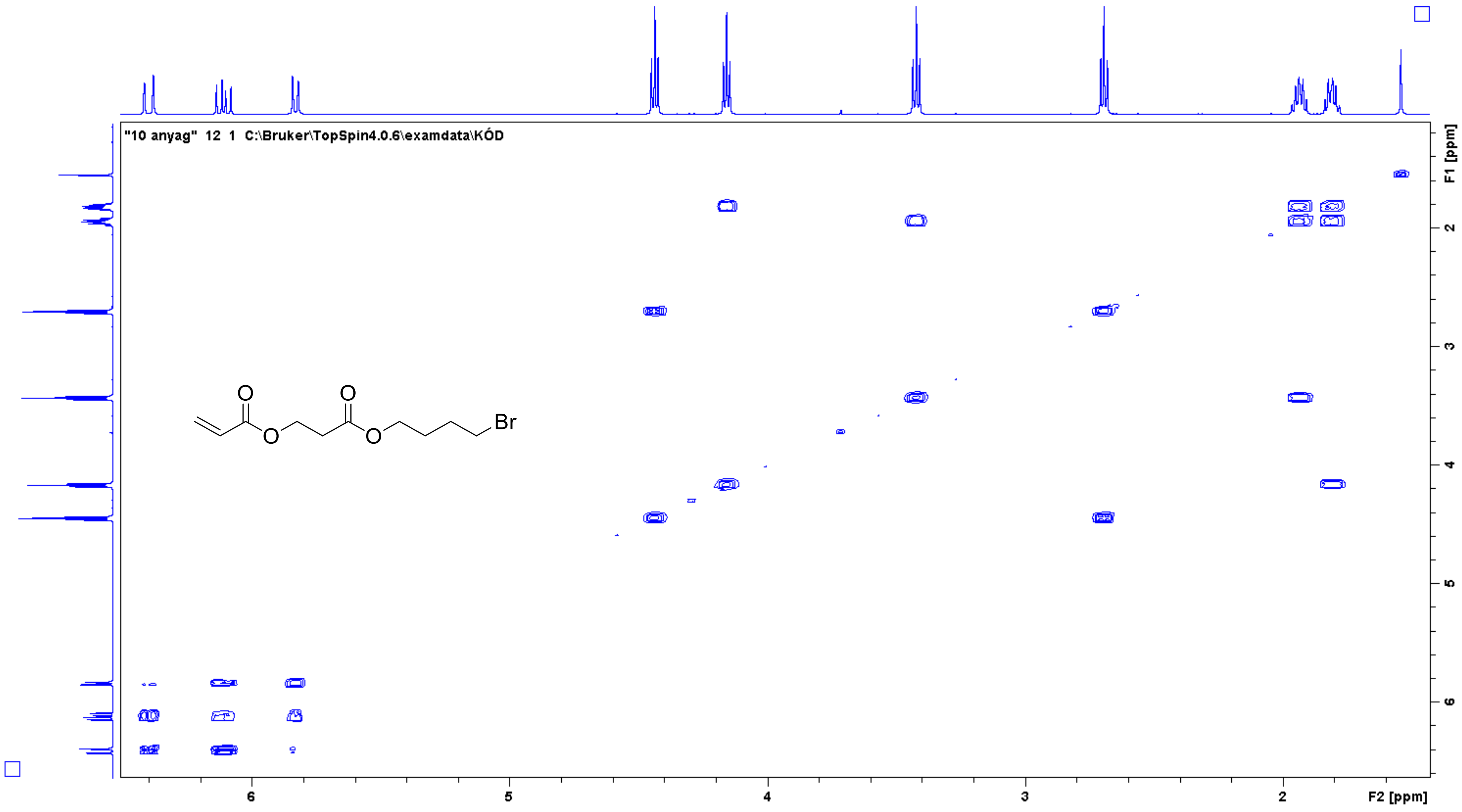

Figure S37

NOESY of compound 3-(4-Bromobutoxy)-3-oxopropyl acrylate **(10)**:

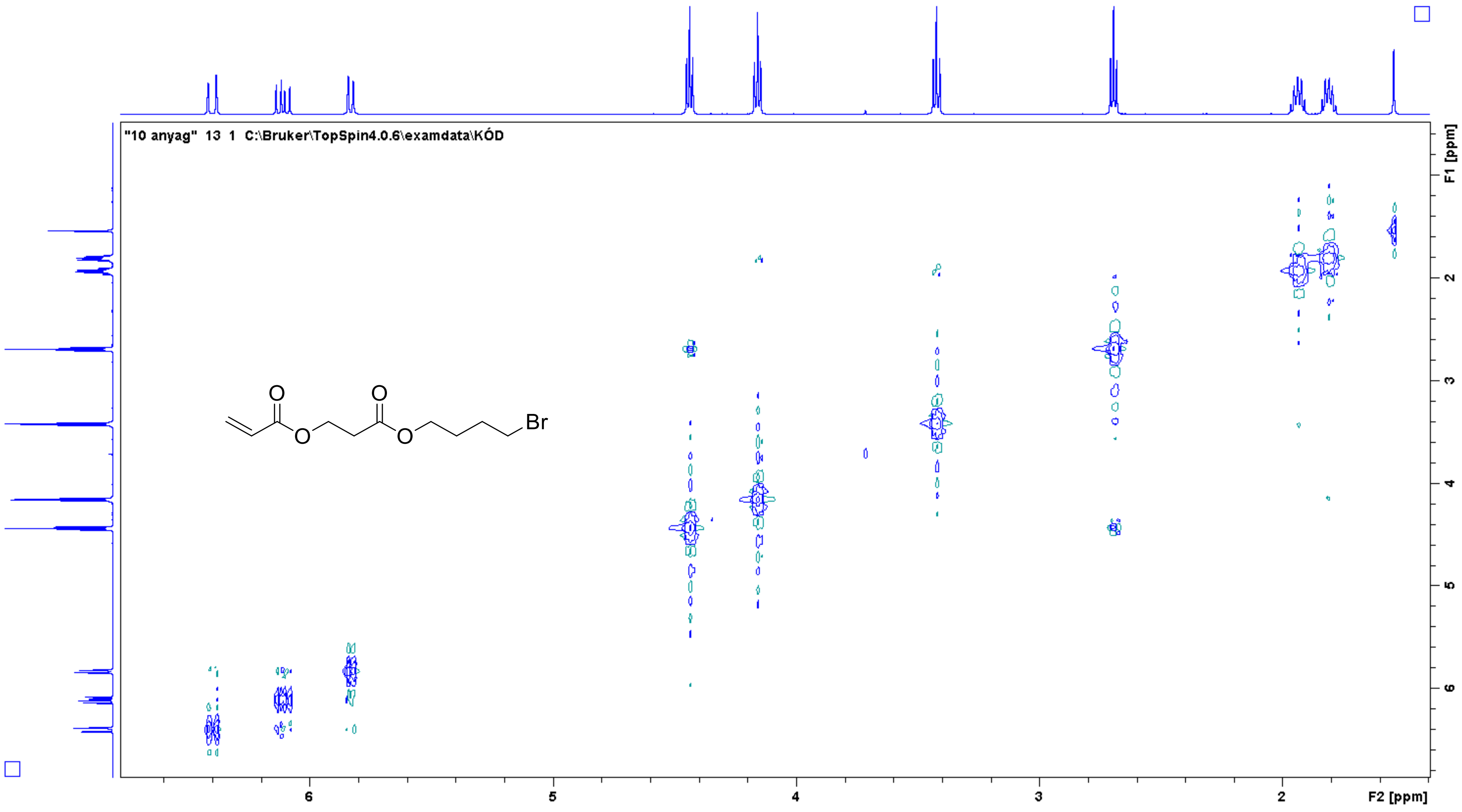

Figure S38

HSQC of compound 3-(4-Bromobutoxy)-3-oxopropyl acrylate **(10)**:

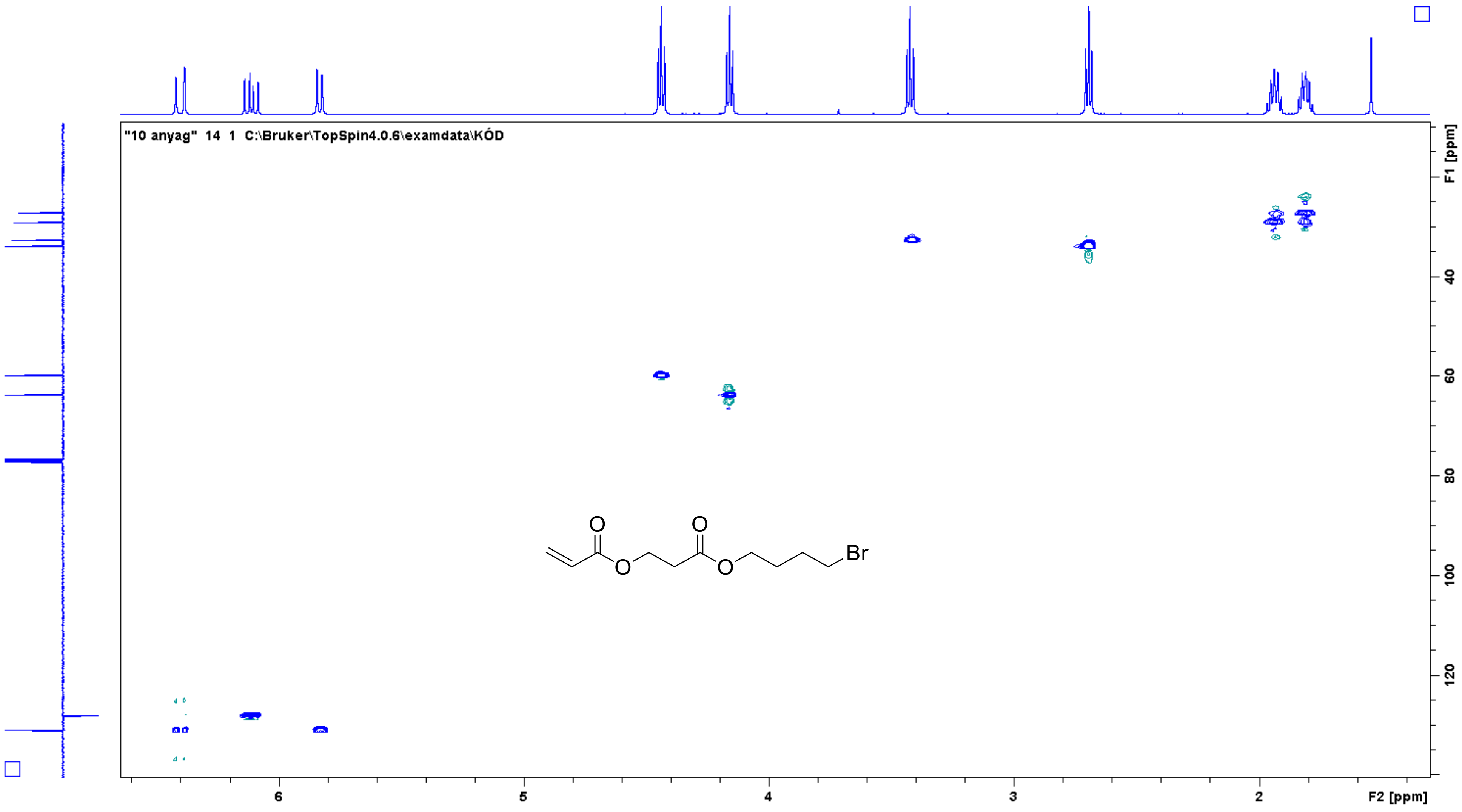

Figure S39

HMBC of compound 3-(4-Bromobutoxy)-3-oxopropyl acrylate **(10)**:

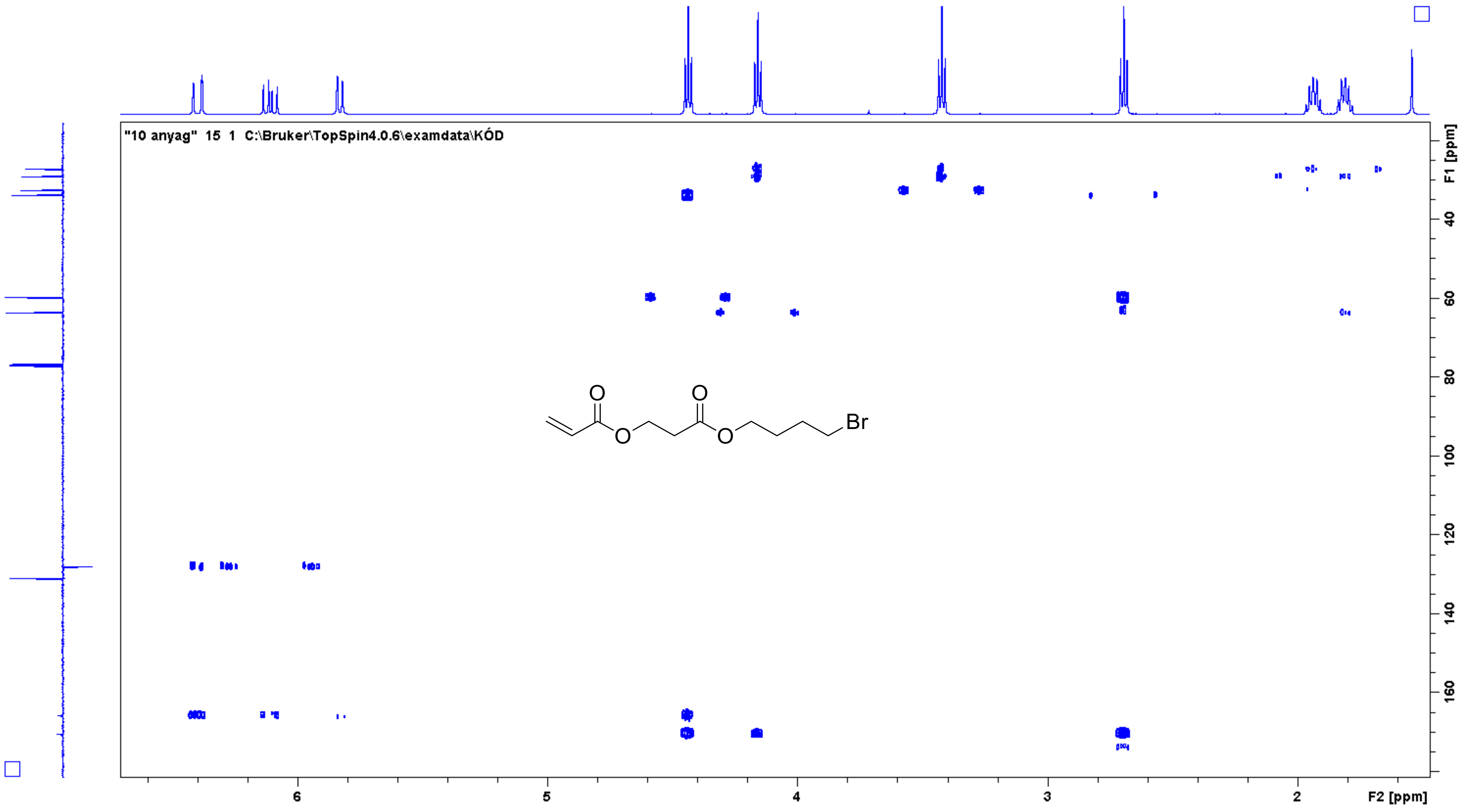

Figure S40

^{1}H-NMR of compound (4*R*,4*aS*,6a*S*,7*R*,8*R*,9*S*,11b*S*)-4-(Acryloyloxy)butyl 7-(((*tert*-butoxycarbonyl)(4-fluorobenzyl)amino)methyl)-8-hydroxy-4,9,11b-trimethyltetradecahydro-6a,9-methanocyclohepta[a]naphthalene-4-carboxylate **(11)**:

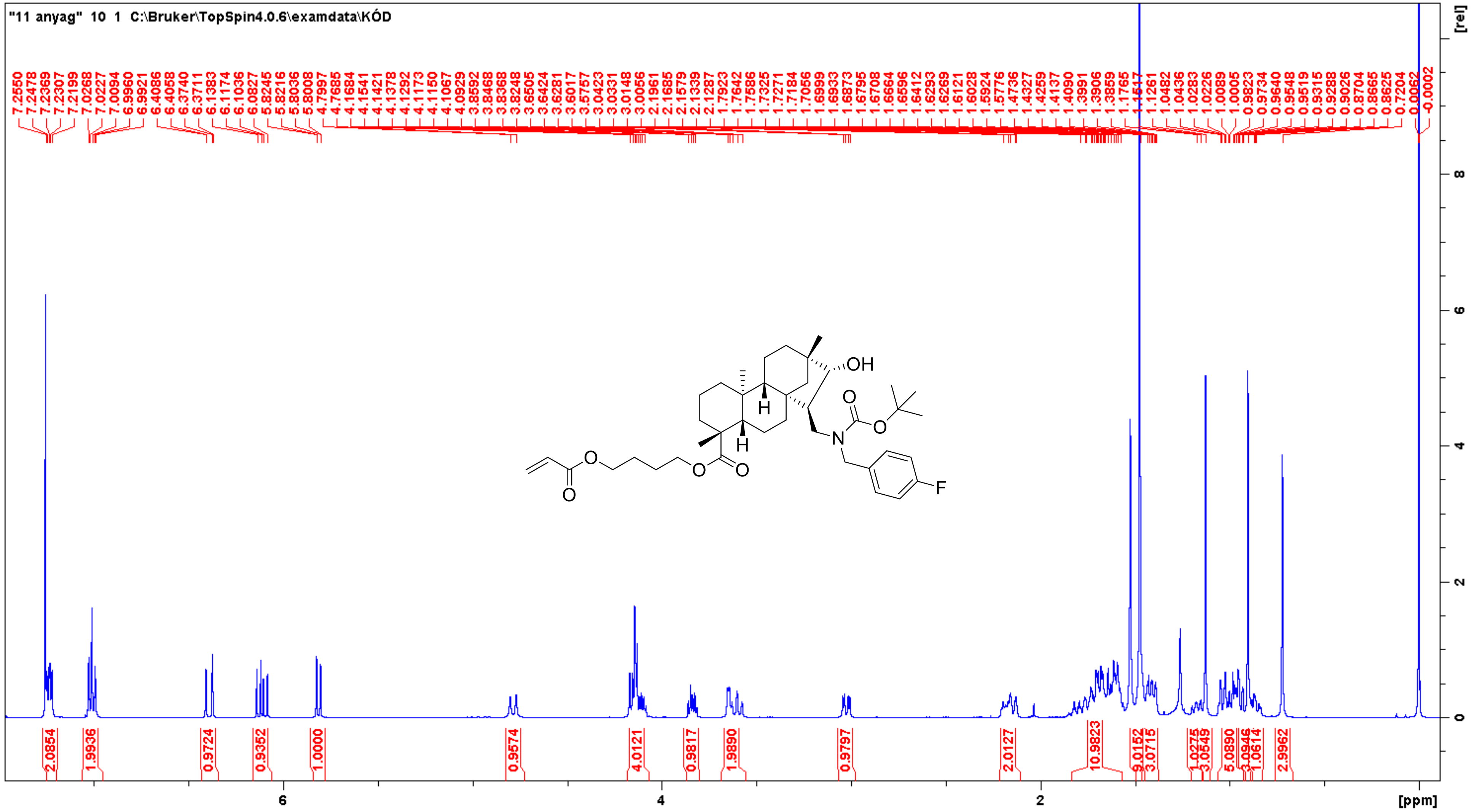

Figure S41

^{13}C-NMR of compound (4*R*,*4aS*,6a*S*,7*R*,8*R*,9*S*,11b*S*)-4-(Acryloyloxy)butyl 7-(((*tert*-butoxycarbonyl)(4-fluorobenzyl)amino)methyl)-8-hydroxy-4,9,11b-trimethyltetradecahydro-6a,9-methanocyclohepta[a]naphthalene-4-carboxylate **(11)**:

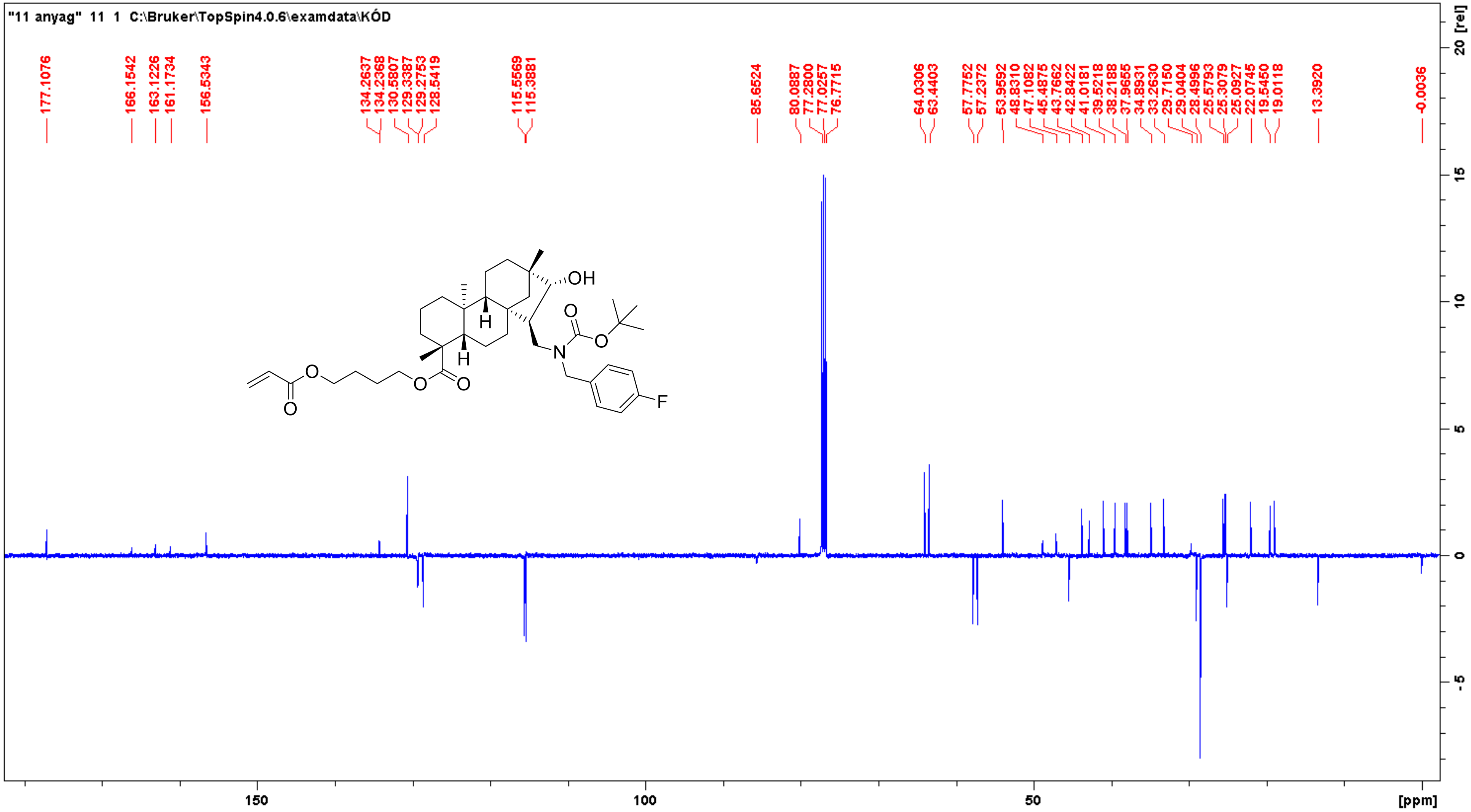

Figure S42

COSY of compound (4*R*,4*aS*,6a*S*,7*R*,8*R*,9*S*,11b*S*)-4-(Acryloyloxy)butyl 7-(((*tert*-butoxycarbonyl)(4-fluorobenzyl)amino)methyl)-8-hydroxy-4,9,11b-trimethyltetradecahydro-6a,9-methanocyclohepta[a]naphthalene-4-carboxylate **(11)**:

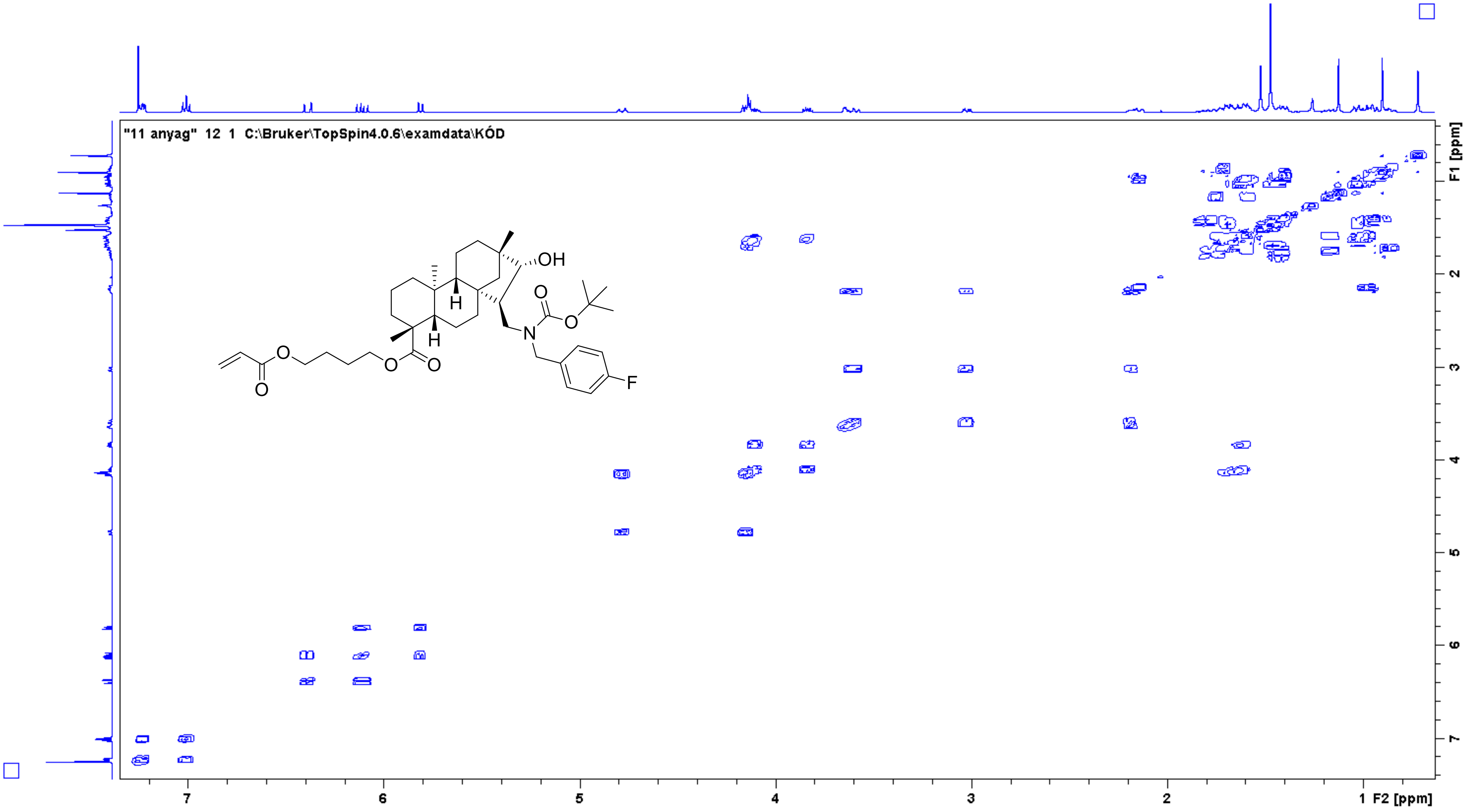

Figure S43

NOESY of compound (4*R*,4*aS*,6a*S*,7*R*,8*R*,9*S*,11b*S*)-4-(Acryloyloxy)butyl 7-(((*tert*-butoxycarbonyl)(4-fluorobenzyl)amino)methyl)-8-hydroxy-4,9,11b-trimethyltetradecahydro-6a,9-methanocyclohepta[a]naphthalene-4-carboxylate **(11)**:

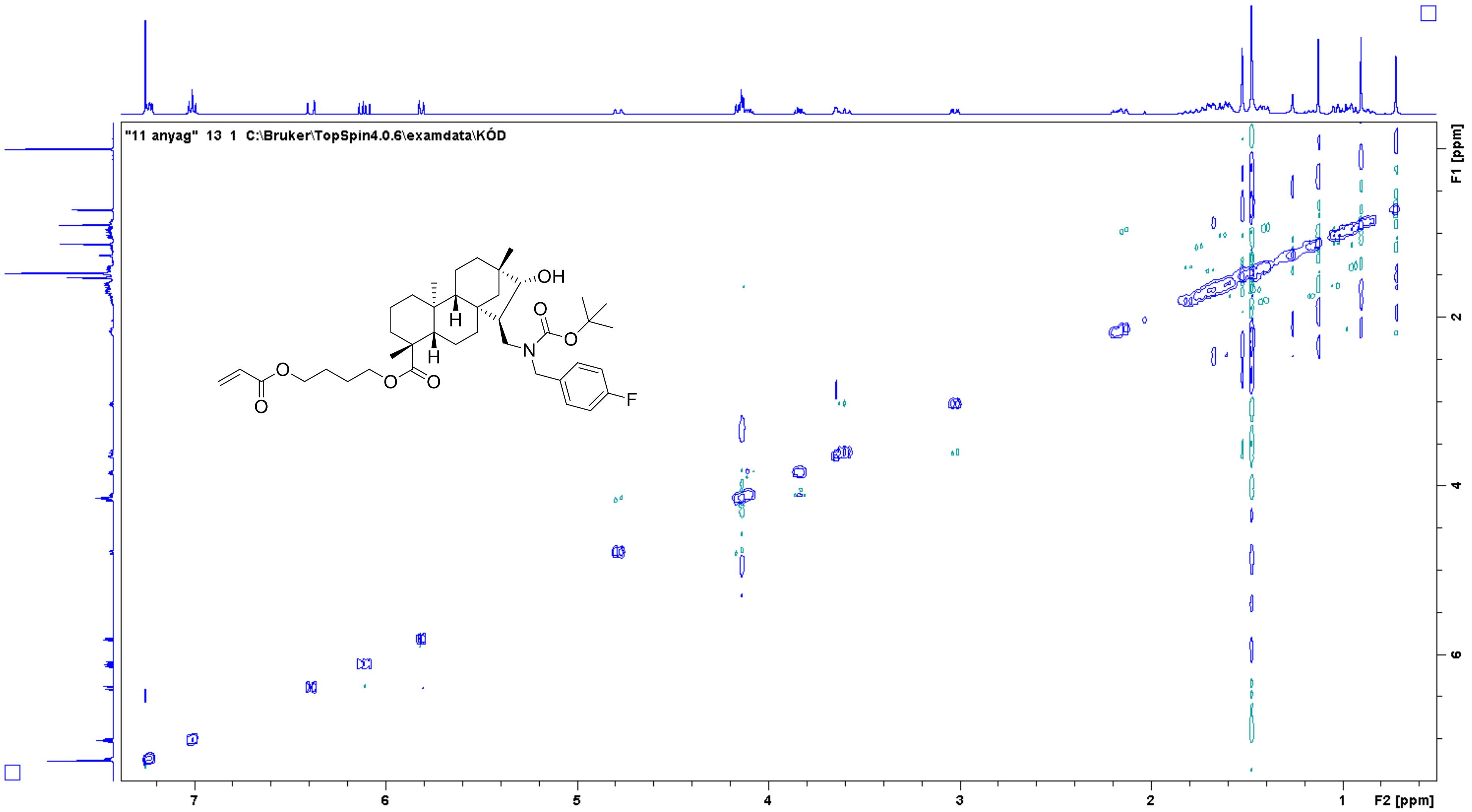

Figure S44

HSQC of compound (4*R*,4*aS*,6a*S*,7*R*,8*R*,9*S*,11b*S*)-4-(Acryloyloxy)butyl 7-(((*tert*-butoxycarbonyl)(4-fluorobenzyl)amino)methyl)-8-hydroxy-4,9,11b-trimethyltetradecahydro-6a,9-methanocyclohepta[a]naphthalene-4-carboxylate **(11)**:

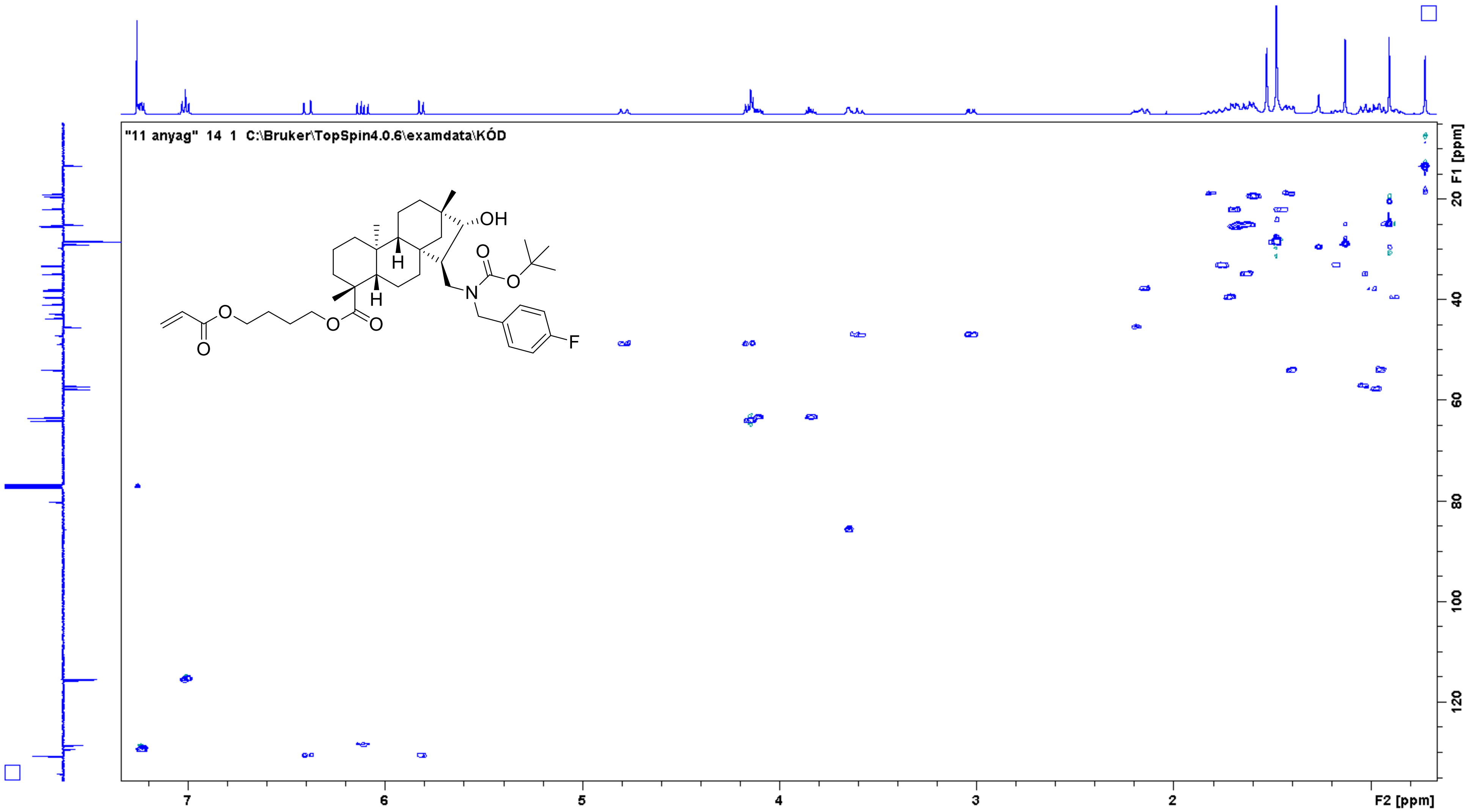

Figure S45

HMBC of compound (4*R*,4*aS*,6a*S*,7*R*,8*R*,9*S*,11b*S*)-4-(Acryloyloxy)butyl 7-(((*tert*-butoxycarbonyl)(4-fluorobenzyl)amino)methyl)-8-hydroxy-4,9,11b-trimethyltetradecahydro-6a,9-methanocyclohepta[a]naphthalene-4-carboxylate **(11)**:

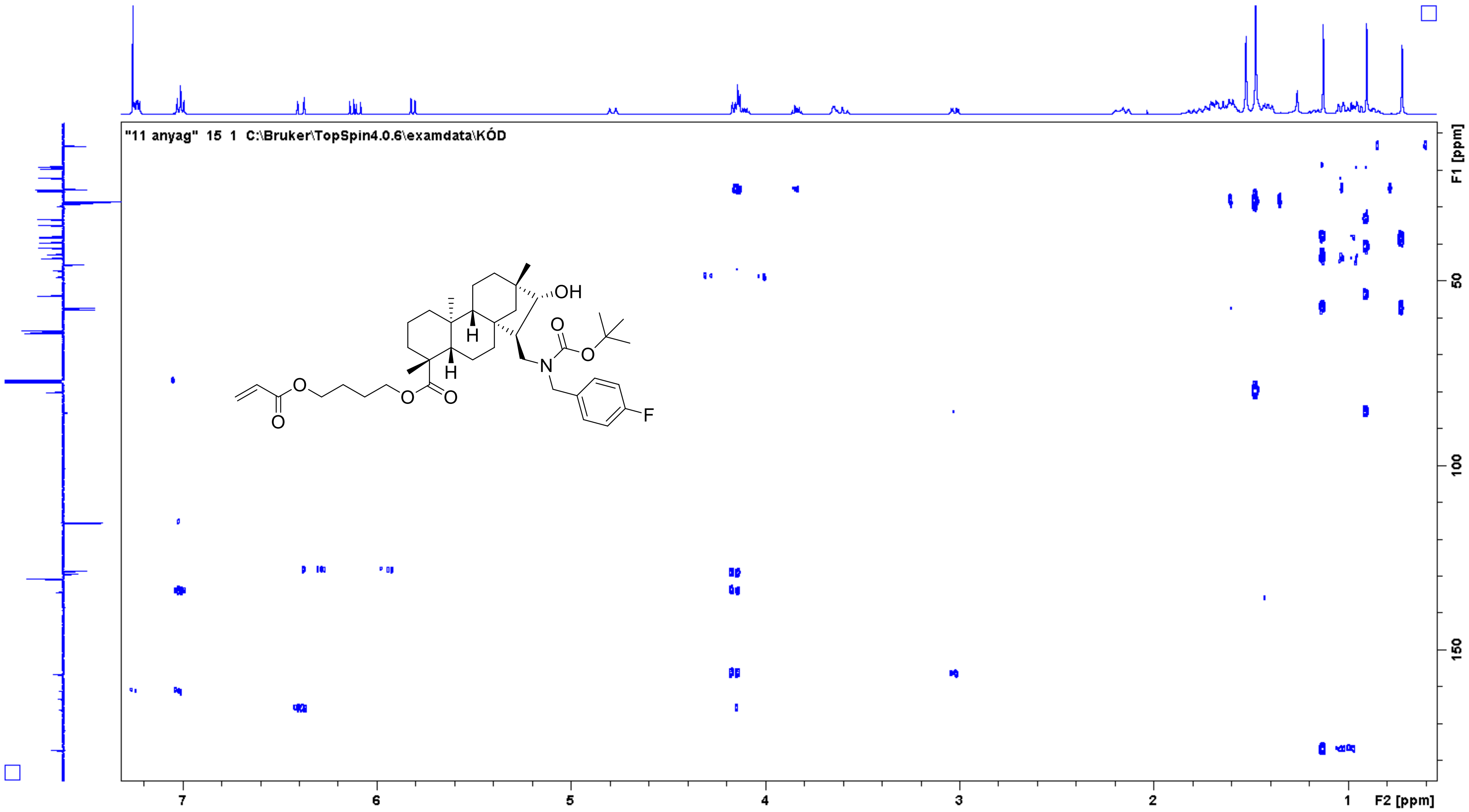

Figure S46

^{19}F-NMR of compound (4*R*,4*aS*,6a*S*,7*R*,8*R*,9*S*,11b*S*)-4-(Acryloyloxy)butyl 7-(((*tert*-butoxycarbonyl)(4-fluorobenzyl)amino)methyl)-8-hydroxy-4,9,11b-trimethyltetradecahydro-6a,9-methanocyclohepta[a]naphthalene-4-carboxylate **(11)**:

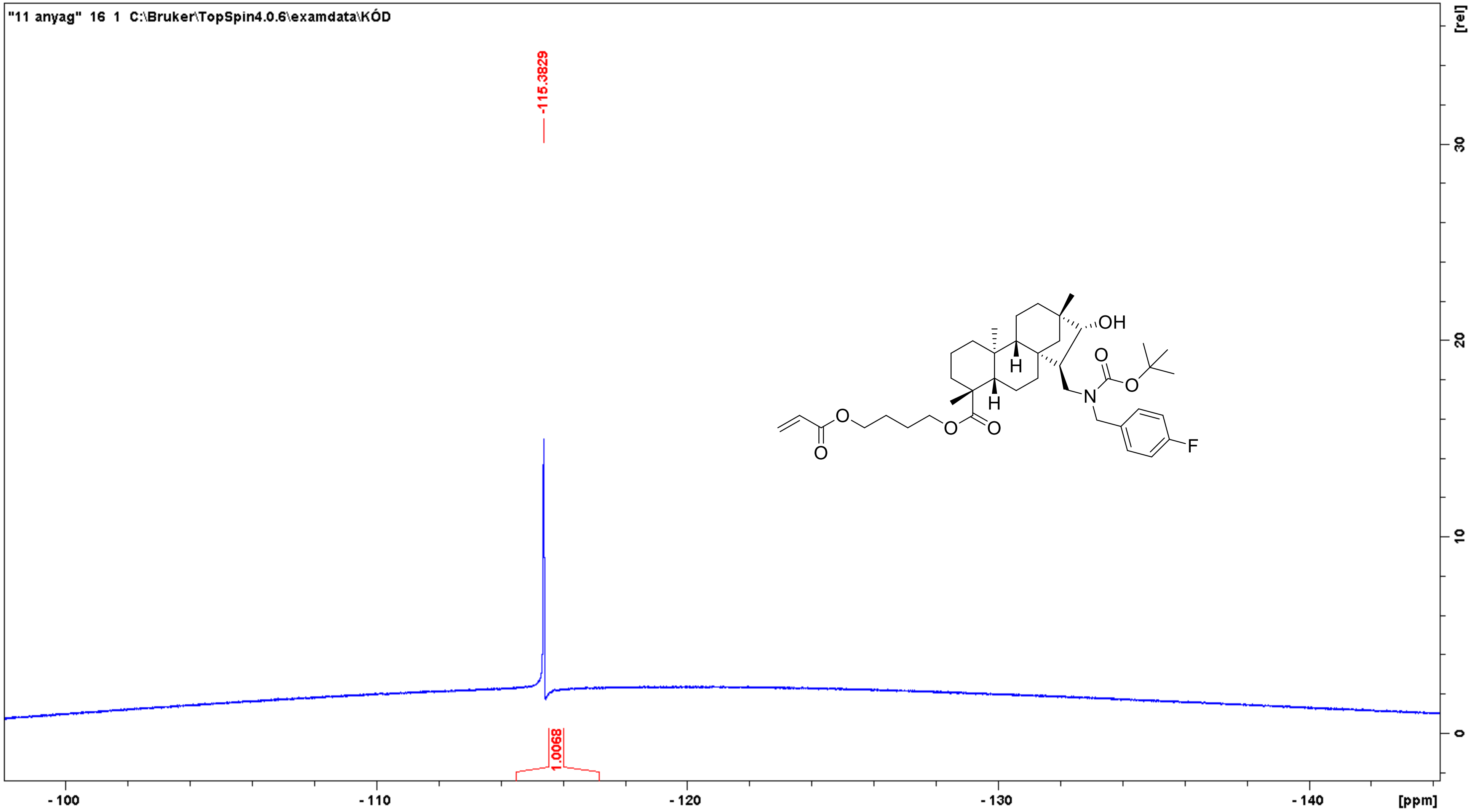

Figure S47

^{1}H-NMR of compound (4*R*,4a*S*,6a*S*,7*R*,8*R*,9*S*,11b*S*)-4-((3-(Acryloyloxy)propanoyl)oxy)butyl 7-(((*tert*-butoxycarbonyl)(4-fluorobenzyl)amino)methyl)-8-hydroxy-4,9,11b-trimethyltetradecahydro-6a,9-methanocyclohepta[a]naphthalene-4-carboxylate **(12)**:

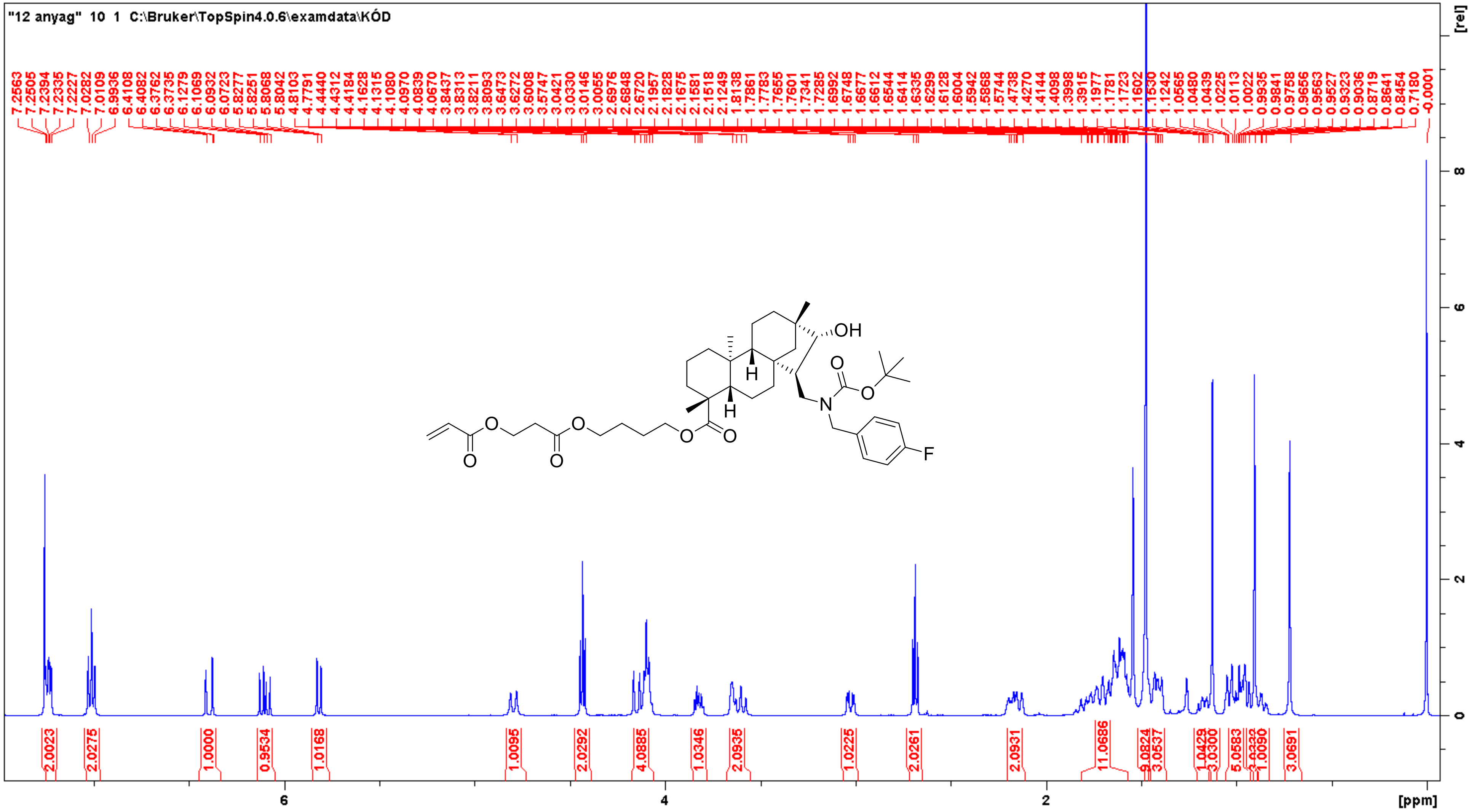

Figure S48

^{13}C-NMR of compound (4*R*,4a*S*,6a*S*,7*R*,8*R*,9*S*,11b*S*)-4-((3-(Acryloyloxy)propanoyl)oxy)butyl 7-(((*tert*-butoxycarbonyl)(4-fluorobenzyl)amino)methyl)-8-hydroxy-4,9,11b-trimethyltetradecahydro-6a,9-methanocyclohepta[a]naphthalene-4-carboxylate **(12)**:

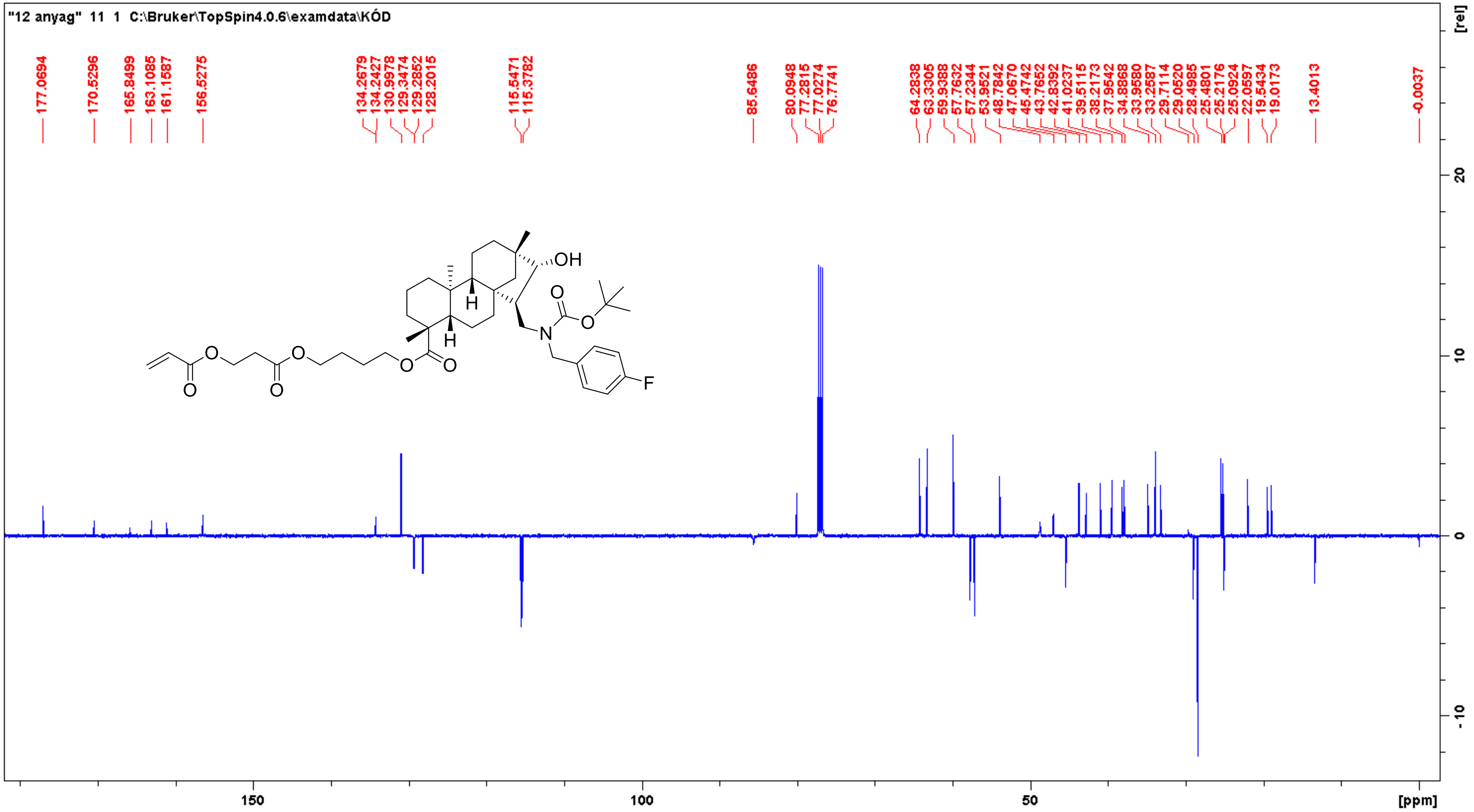

Figure S49

COSY of compound (4*R*,4a*S*,6a*S*,7*R*,8*R*,9*S*,11b*S*)-4-((3-(Acryloyloxy)propanoyl)oxy)butyl 7-(((*tert*-butoxycarbonyl)(4-fluorobenzyl)amino)methyl)-8-hydroxy-4,9,11b-trimethyltetradecahydro-6a,9-methanocyclohepta[a]naphthalene-4-carboxylate **(12)**:

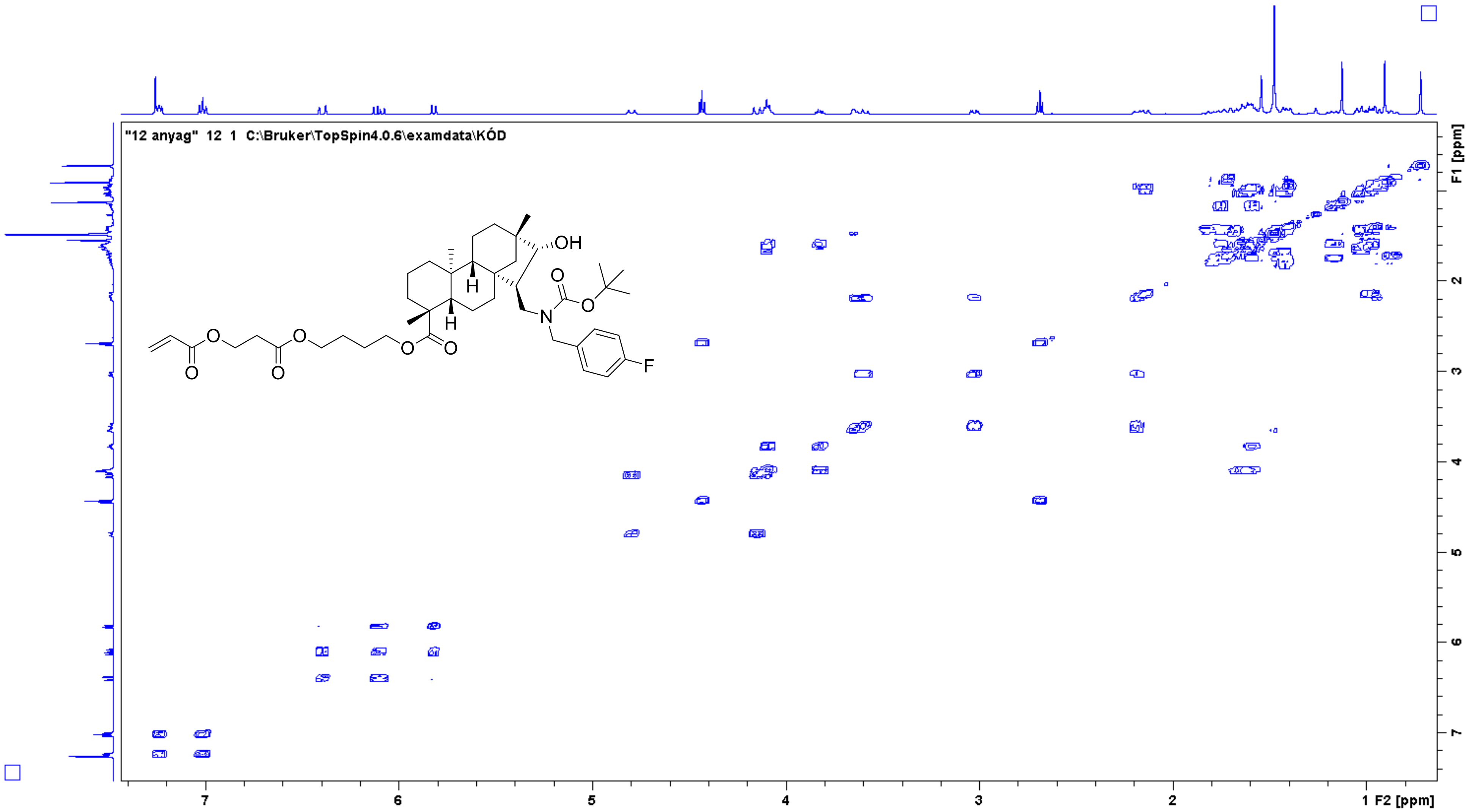

Figure S50

NOESY of compound (4*R*,4a*S*,6a*S*,7*R*,8*R*,9*S*,11b*S*)-4-((3-(Acryloyloxy)propanoyl)oxy)butyl 7-(((*tert*-butoxycarbonyl)(4-fluorobenzyl)amino)methyl)-8-hydroxy-4,9,11b-trimethyltetradecahydro-6a,9-methanocyclohepta[a]naphthalene-4-carboxylate **(12)**:

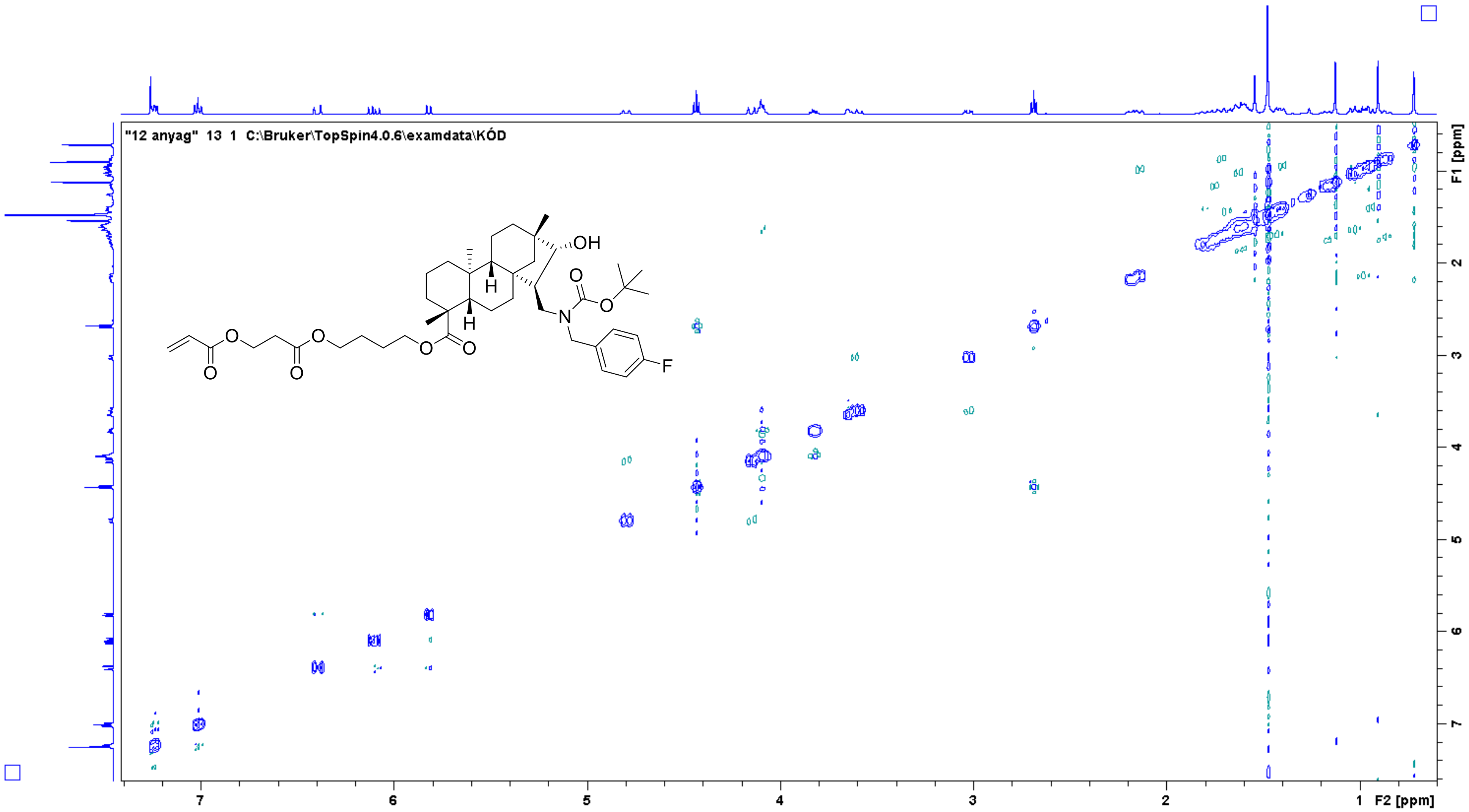

Figure S51

HSQC of compound (4*R*,4a*S*,6a*S*,7*R*,8*R*,9*S*,11b*S*)-4-((3-(Acryloyloxy)propanoyl)oxy)butyl 7-(((*tert*-butoxycarbonyl)(4-fluorobenzyl)amino)methyl)-8-hydroxy-4,9,11b-trimethyltetradecahydro-6a,9-methanocyclohepta[a]naphthalene-4-carboxylate **(12)**:

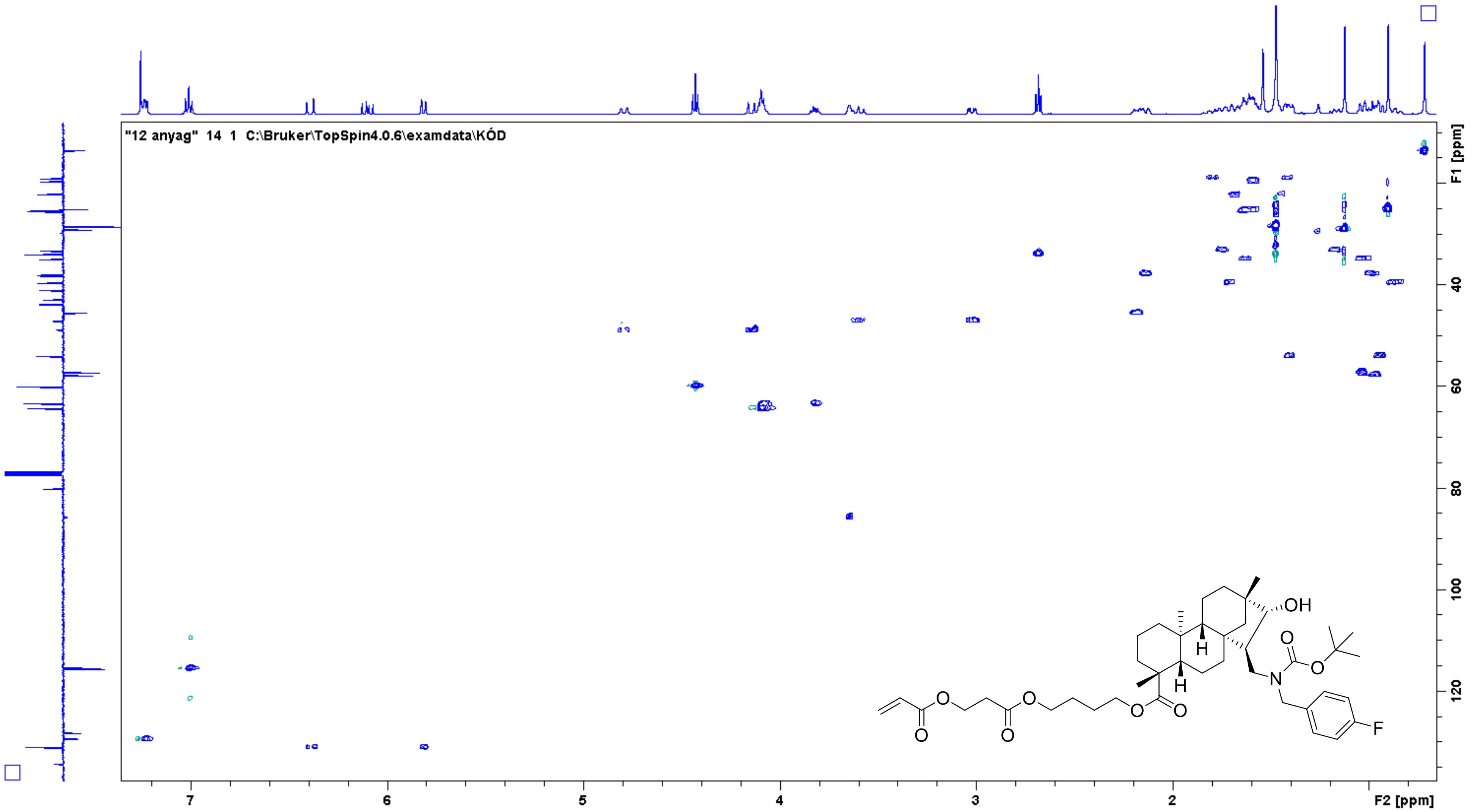

Figure S52

HMBC of compound (4*R*,4a*S*,6a*S*,7*R*,8*R*,9*S*,11b*S*)-4-((3-(Acryloyloxy)propanoyl)oxy)butyl 7-(((*tert*-butoxycarbonyl)(4-fluorobenzyl)amino)methyl)-8-hydroxy-4,9,11b-trimethyltetradecahydro-6a,9-methanocyclohepta[a]naphthalene-4-carboxylate **(12)**:

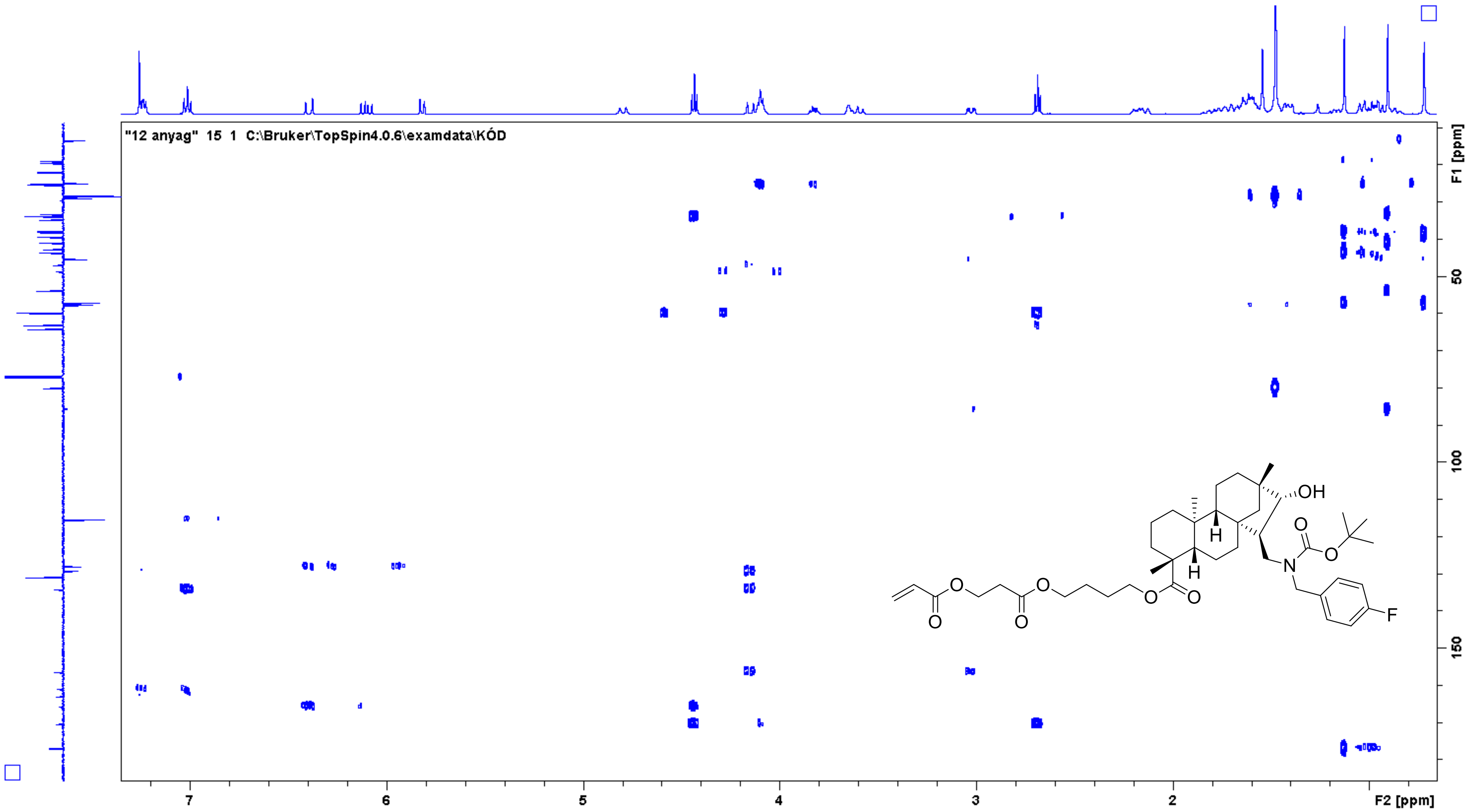

Figure S53

^{19}F-NMR of compound (4*R*,4a*S*,6a*S*,7*R*,8*R*,9*S*,11b*S*)-4-((3-(Acryloyloxy)propanoyl)oxy)butyl 7-(((*tert*-butoxycarbonyl)(4-fluorobenzyl)amino)methyl)-8-hydroxy-4,9,11b-trimethyltetradecahydro-6a,9-methanocyclohepta[a]naphthalene-4-carboxylate **(12)**:

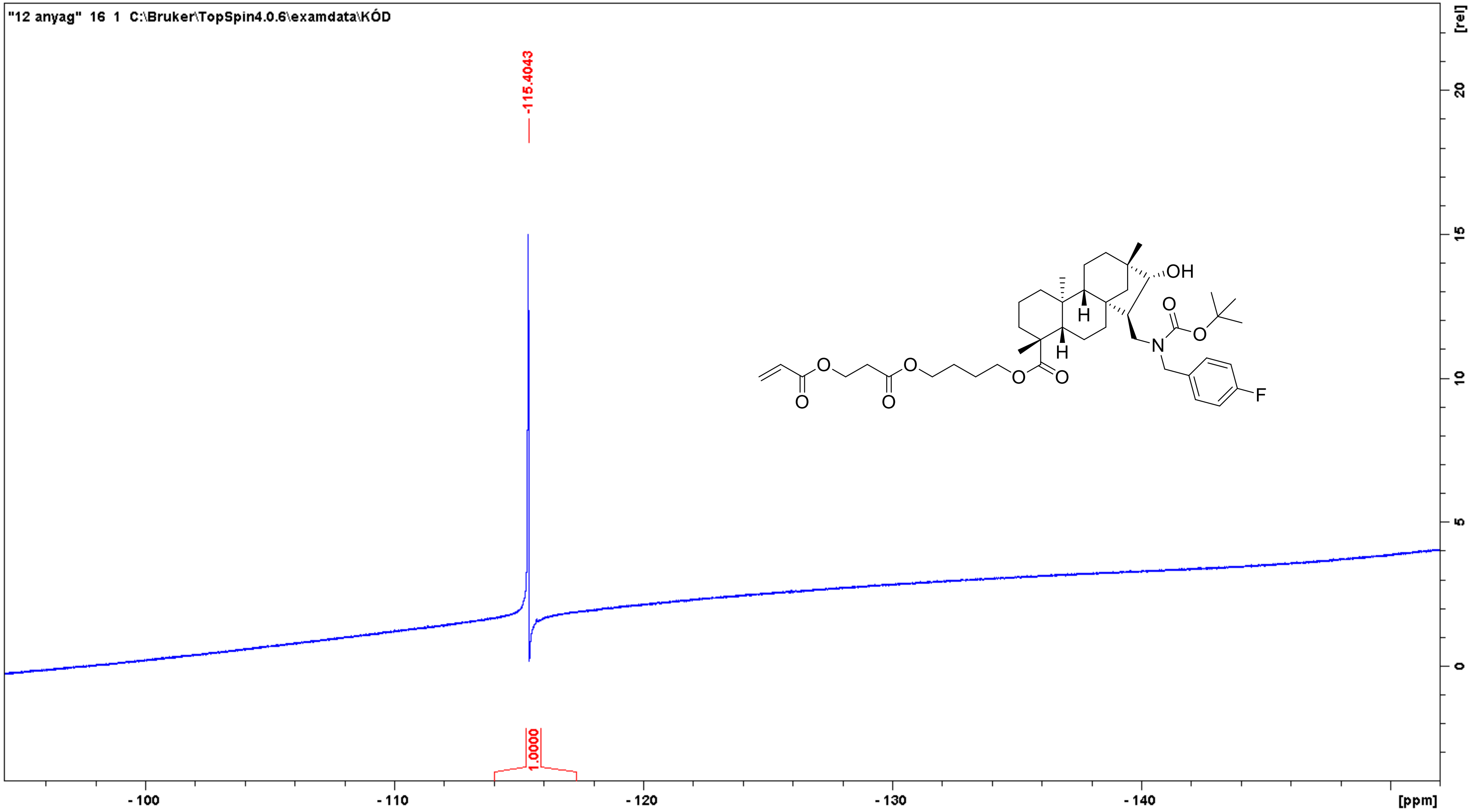

Figure S54

^{1}H-NMR of compound (4*R*,4a*S*,6a*S*,7*R*,8*R*,9*S*,11b*S*)-Prop-2-yn-1-yl 7-(((*tert*-butoxycarbonyl)(4-fluorobenzyl)amino)methyl)-8-hydroxy-4,9,11b-trimethyltetradecahydro-6a,9-methanocyclohepta[a]naphthalene-4-carboxylate **(13)**:

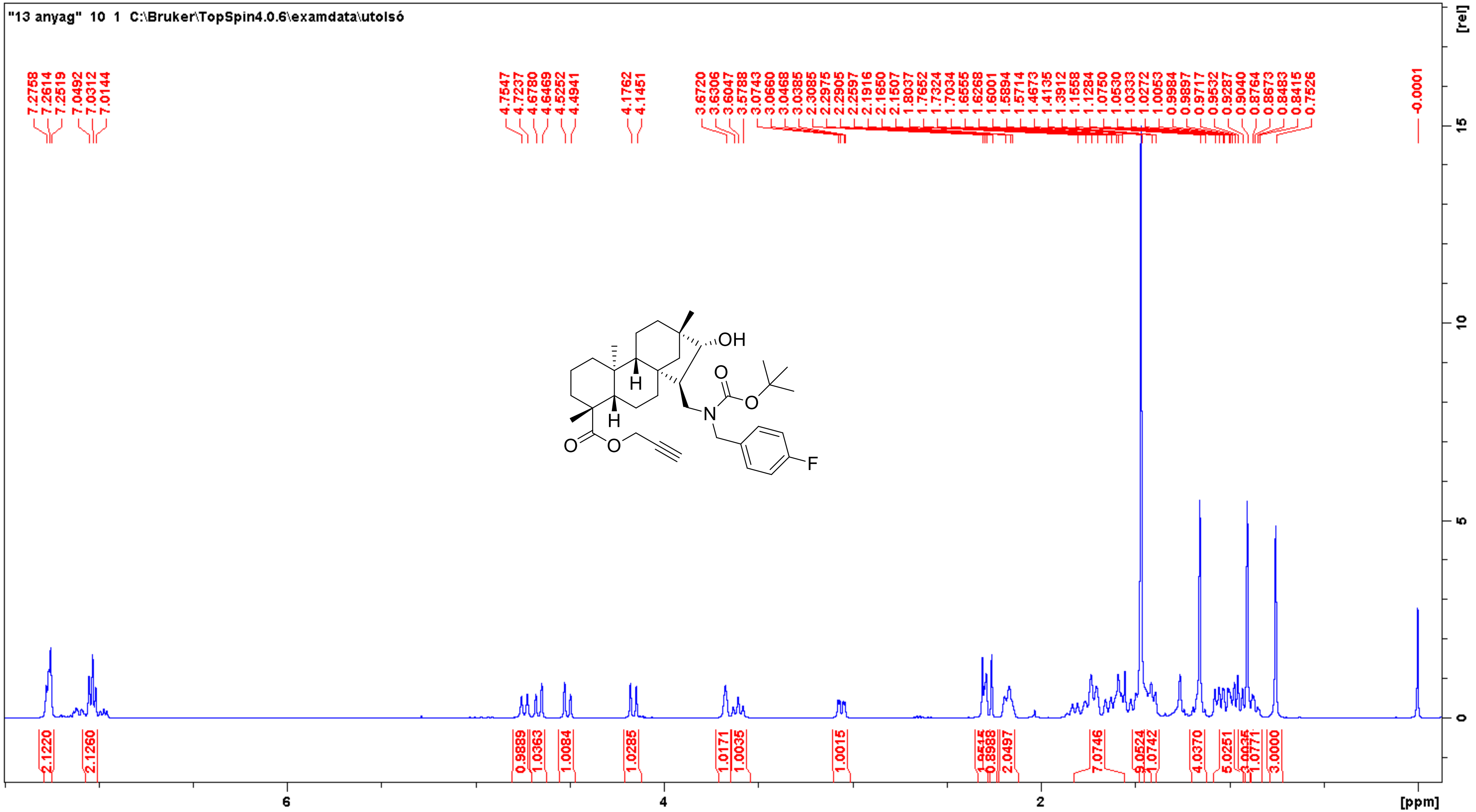

Figure S55

^{13}C-NMR of compound (4*R*,4a*S*,6a*S*,7*R*,8*R*,9*S*,11b*S*)-Prop-2-yn-1-yl 7-(((*tert*-butoxycarbonyl)(4-fluorobenzyl)amino)methyl)-8-hydroxy-4,9,11b-trimethyltetradecahydro-6a,9-methanocyclohepta[a]naphthalene-4-carboxylate **(13)**:

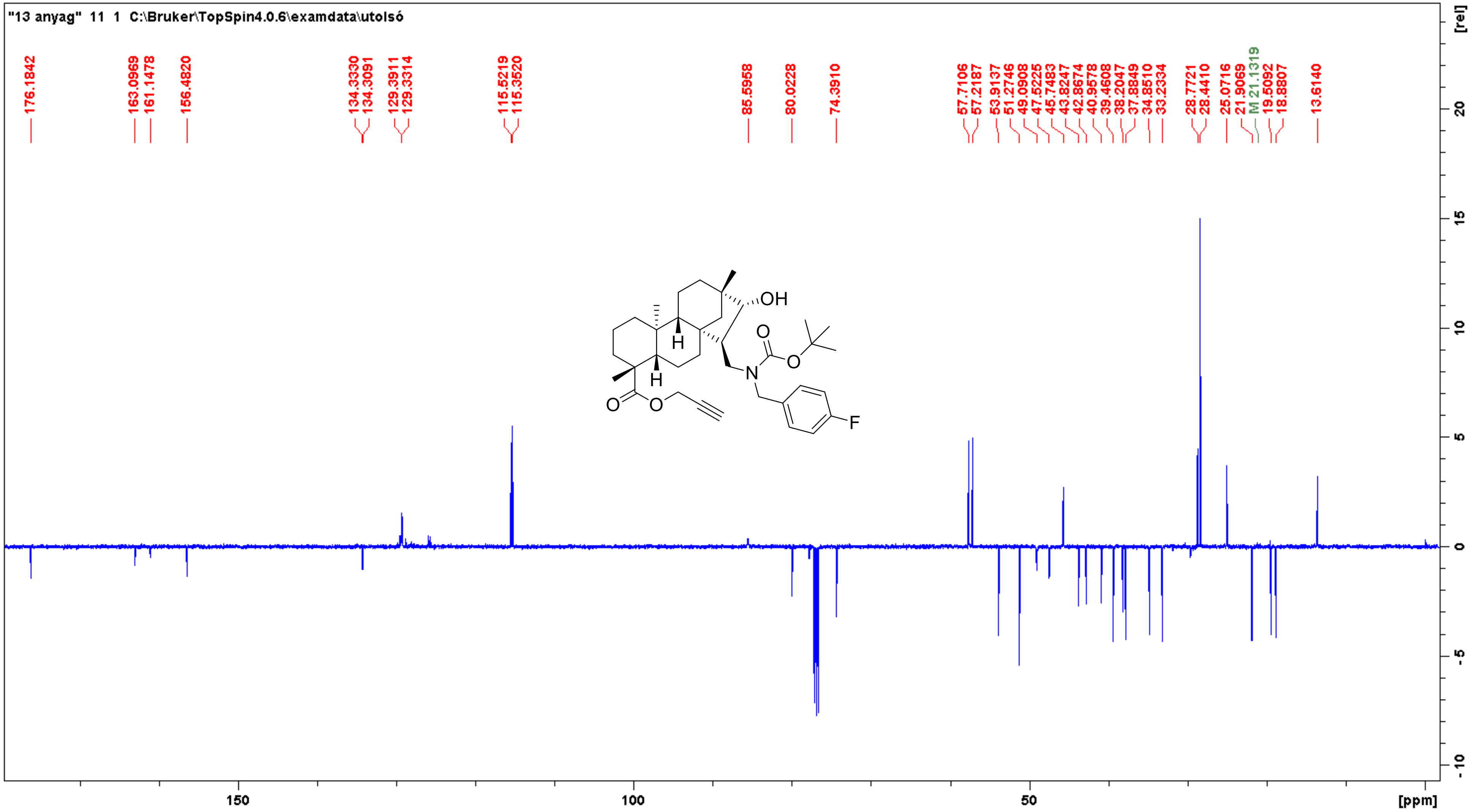

Figure S56

COSY of compound (4*R*,4a*S*,6a*S*,7*R*,8*R*,9*S*,11b*S*)-Prop-2-yn-1-yl 7-(((*tert*-butoxycarbonyl)(4-fluorobenzyl)amino)methyl)-8-hydroxy-4,9,11b-trimethyltetradecahydro-6a,9-methanocyclohepta[a]naphthalene-4-carboxylate **(13)**:

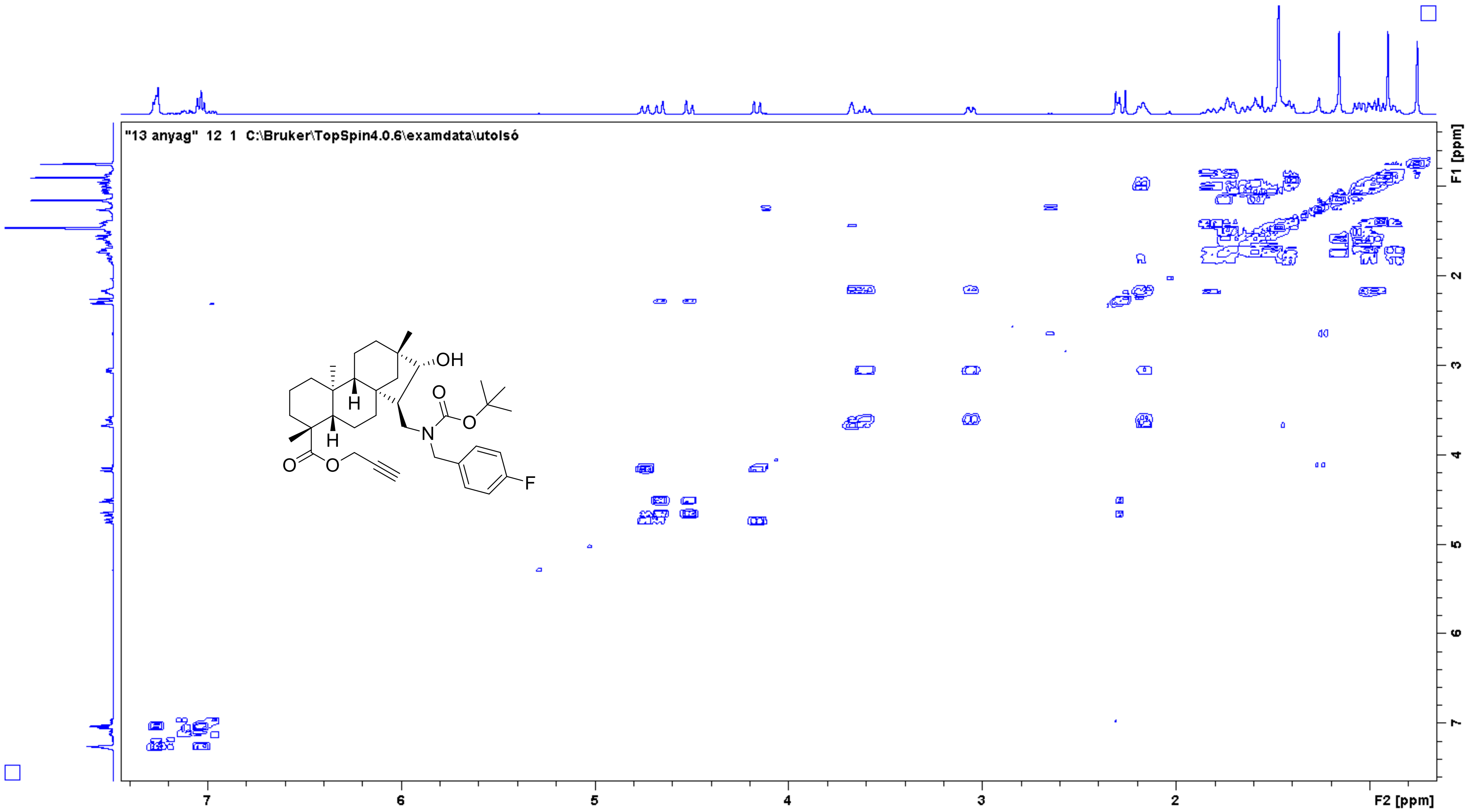

Figure S57

NOESY of compound (4*R*,4a*S*,6a*S*,7*R*,8*R*,9*S*,11b*S*)-Prop-2-yn-1-yl 7-(((*tert*-butoxycarbonyl)(4-fluorobenzyl)amino)methyl)-8-hydroxy-4,9,11b-trimethyltetradecahydro-6a,9-methanocyclohepta[a]naphthalene-4-carboxylate **(13)**:

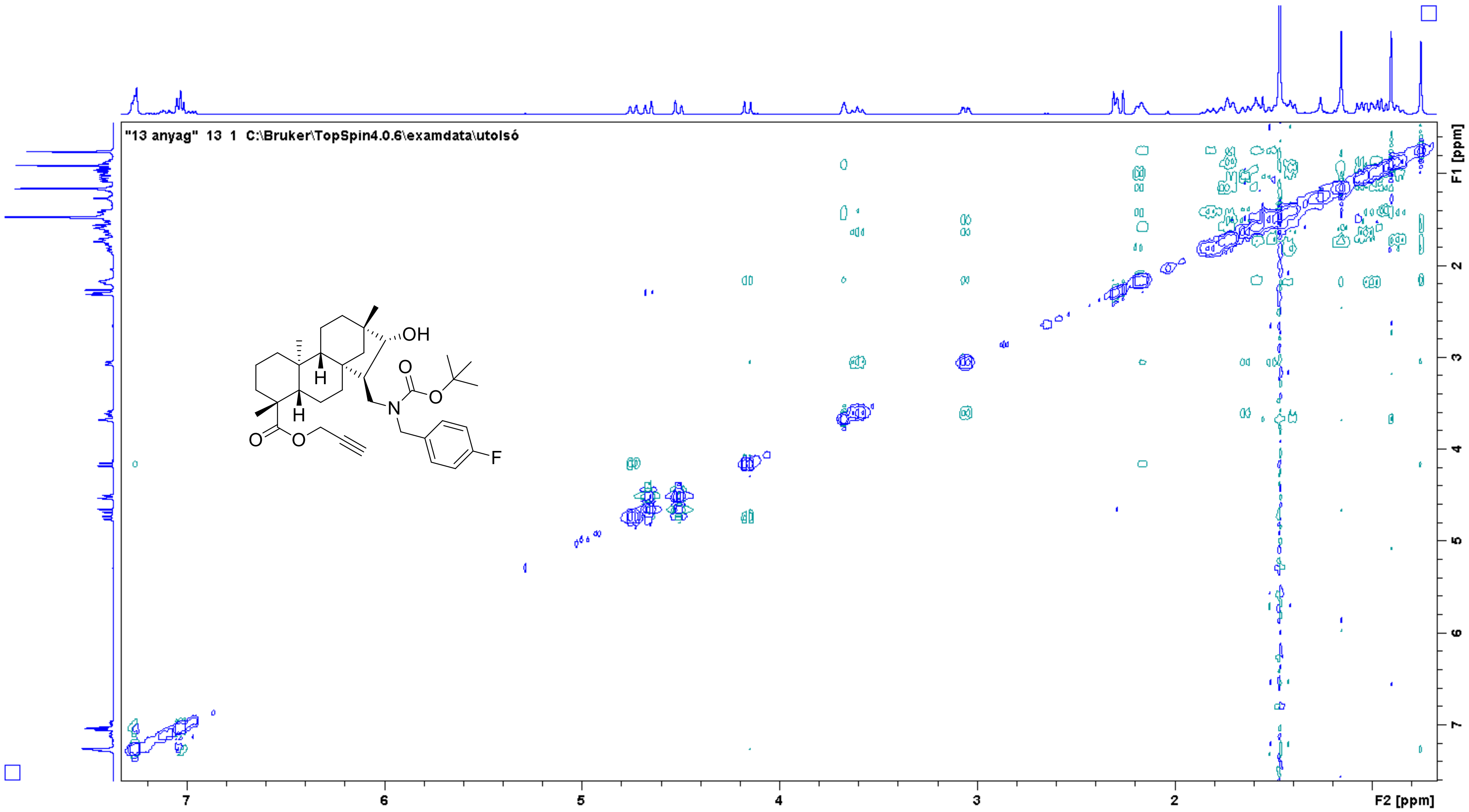

Figure S58

HSQC of compound (4*R*,4a*S*,6a*S*,7*R*,8*R*,9*S*,11b*S*)-Prop-2-yn-1-yl 7-(((*tert*-butoxycarbonyl)(4-fluorobenzyl)amino)methyl)-8-hydroxy-4,9,11b-trimethyltetradecahydro-6a,9-methanocyclohepta[a]naphthalene-4-carboxylate **(13)**:

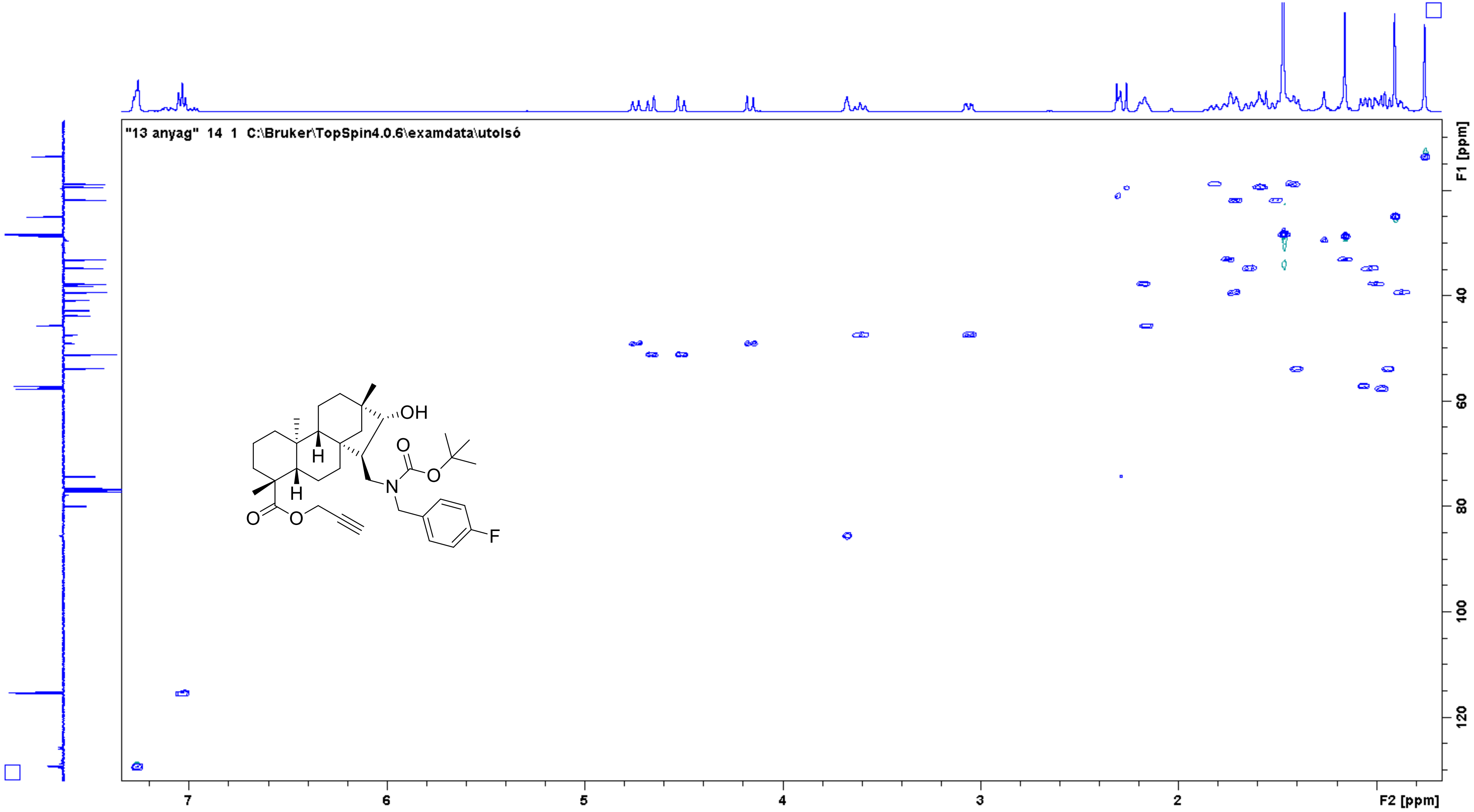

Figure S59

HMBC of compound (4*R*,4a*S*,6a*S*,7*R*,8*R*,9*S*,11b*S*)-Prop-2-yn-1-yl 7-(((*tert*-butoxycarbonyl)(4-fluorobenzyl)amino)methyl)-8-hydroxy-4,9,11b-trimethyltetradecahydro-6a,9-methanocyclohepta[a]naphthalene-4-carboxylate **(13)**:

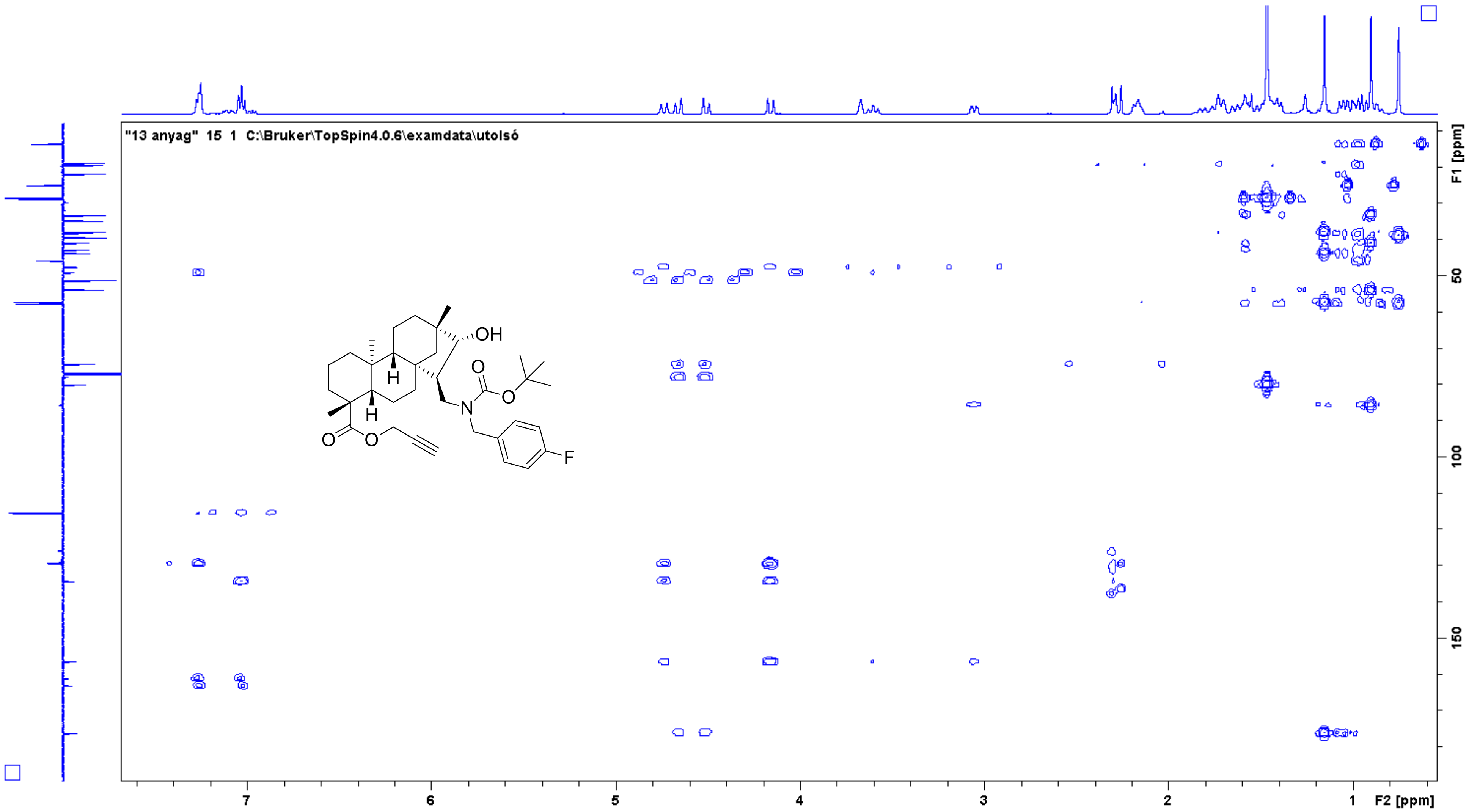

Figure S60

^{1}H-NMR of compound (4*R*,4a*S*,6a*S*,7*R*,8*R*,9*S*,11b*S*)-Prop-2-yn-1-yl 7-(((4-fluorobenzyl)amino)methyl)-8-hydroxy-4,9,11b-trimethyltetradecahydro-6a,9-methanocyclohepta[a]naphthalene-4-carboxylate **(14)**:

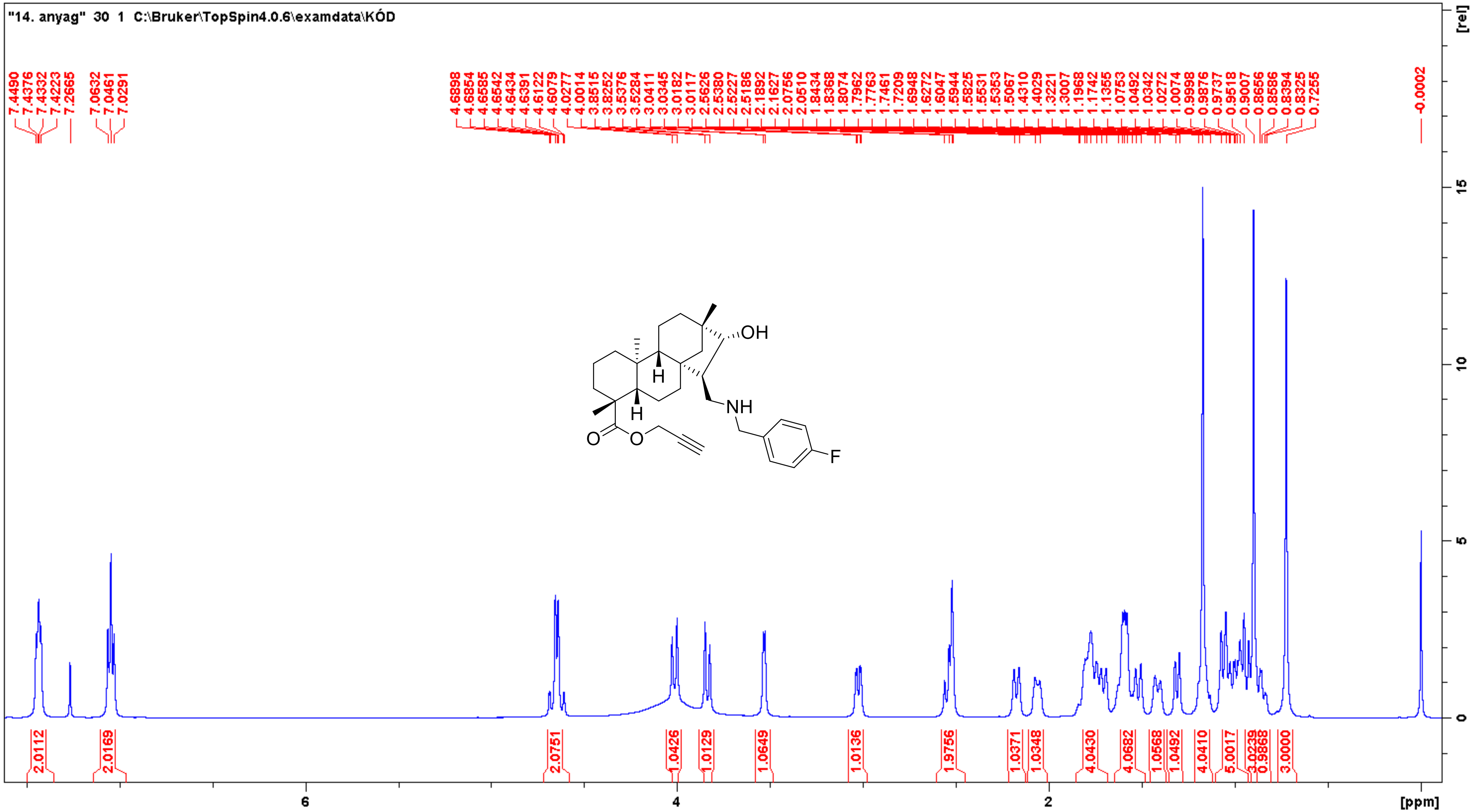

Figure S61

^{13}C-NMR of compound (4*R*,4a*S*,6a*S*,7*R*,8*R*,9*S*,11b*S*)-Prop-2-yn-1-yl 7-(((4-fluorobenzyl)amino)methyl)-8-hydroxy-4,9,11b-trimethyltetradecahydro-6a,9-methanocyclohepta[a]naphthalene-4-carboxylate **(14)**:

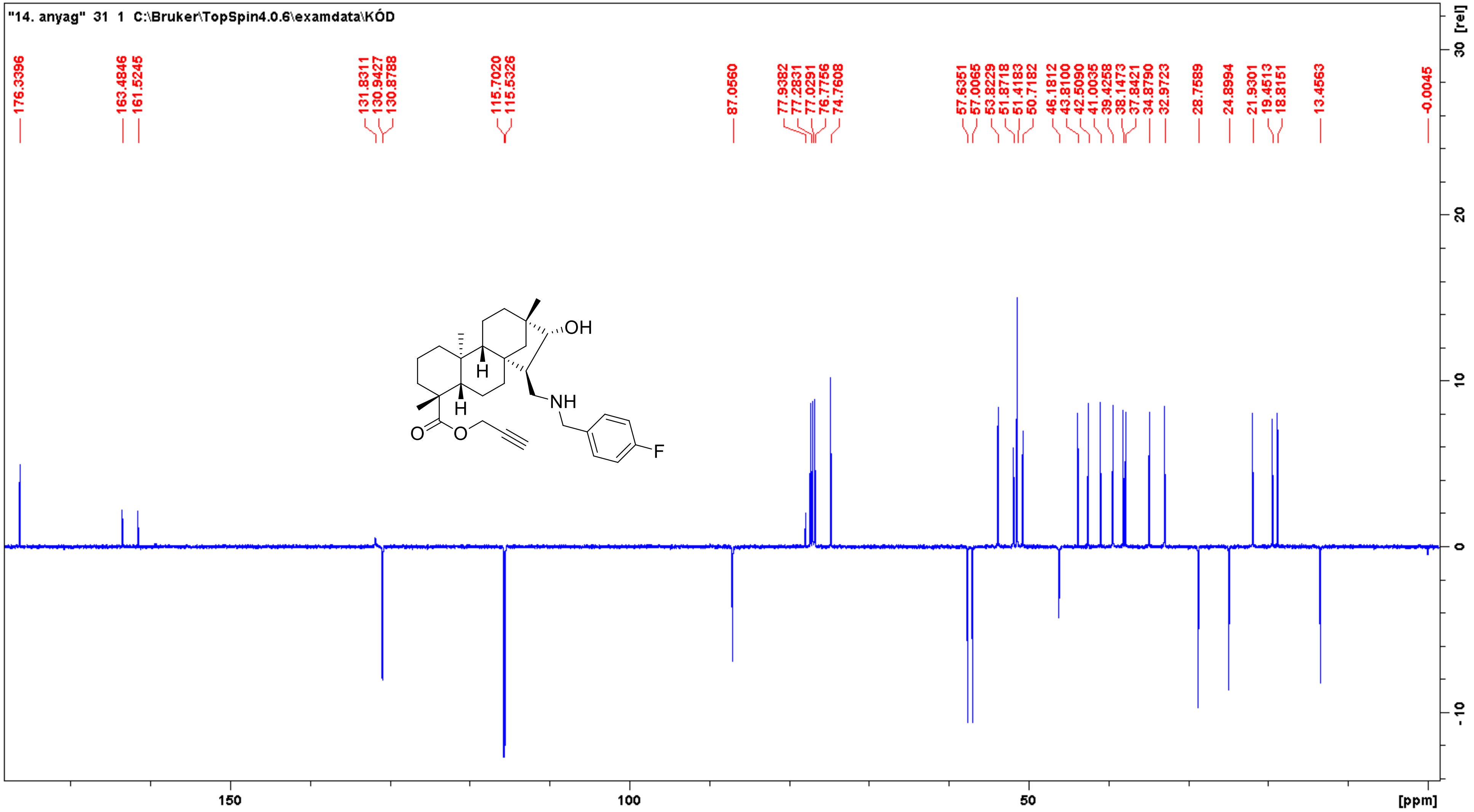

Figure S62

COSY of compound (4*R*,4a*S*,6a*S*,7*R*,8*R*,9*S*,11b*S*)-Prop-2-yn-1-yl 7-(((4-fluorobenzyl)amino)methyl)-8-hydroxy-4,9,11b-trimethyltetradecahydro-6a,9-methanocyclohepta[a]naphthalene-4-carboxylate **(14)**:

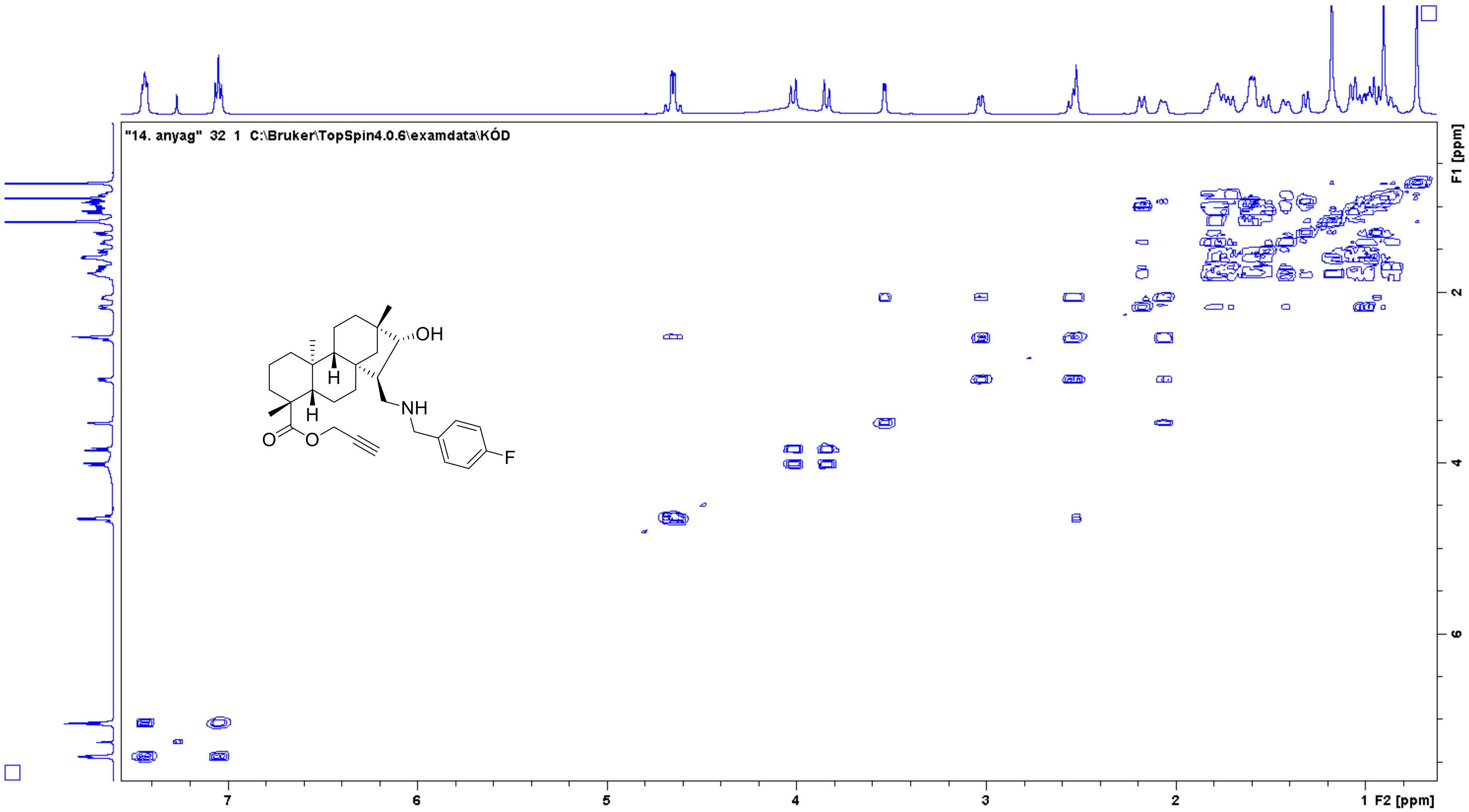

Figure S63

NOESY of compound (4*R*,4a*S*,6a*S*,7*R*,8*R*,9*S*,11b*S*)-Prop-2-yn-1-yl 7-(((4-fluorobenzyl)amino)methyl)-8-hydroxy-4,9,11b-trimethyltetradecahydro-6a,9-methanocyclohepta[a]naphthalene-4-carboxylate **(14)**:

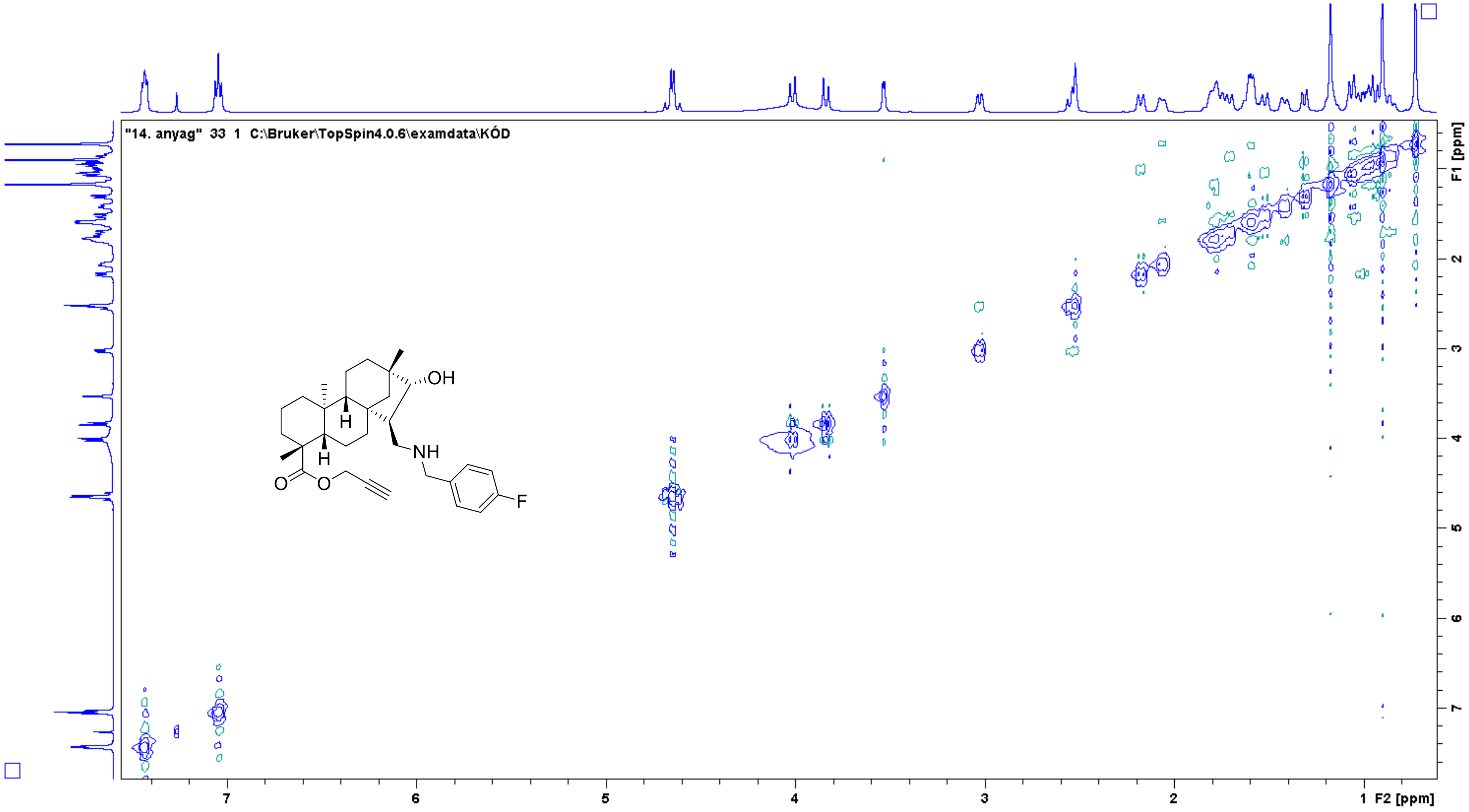

Figure S64

HSQC of compound (4*R*,4a*S*,6a*S*,7*R*,8*R*,9*S*,11b*S*)-Prop-2-yn-1-yl 7-(((4-fluorobenzyl)amino)methyl)-8-hydroxy-4,9,11b-trimethyltetradecahydro-6a,9-methanocyclohepta[a]naphthalene-4-carboxylate **(14)**:

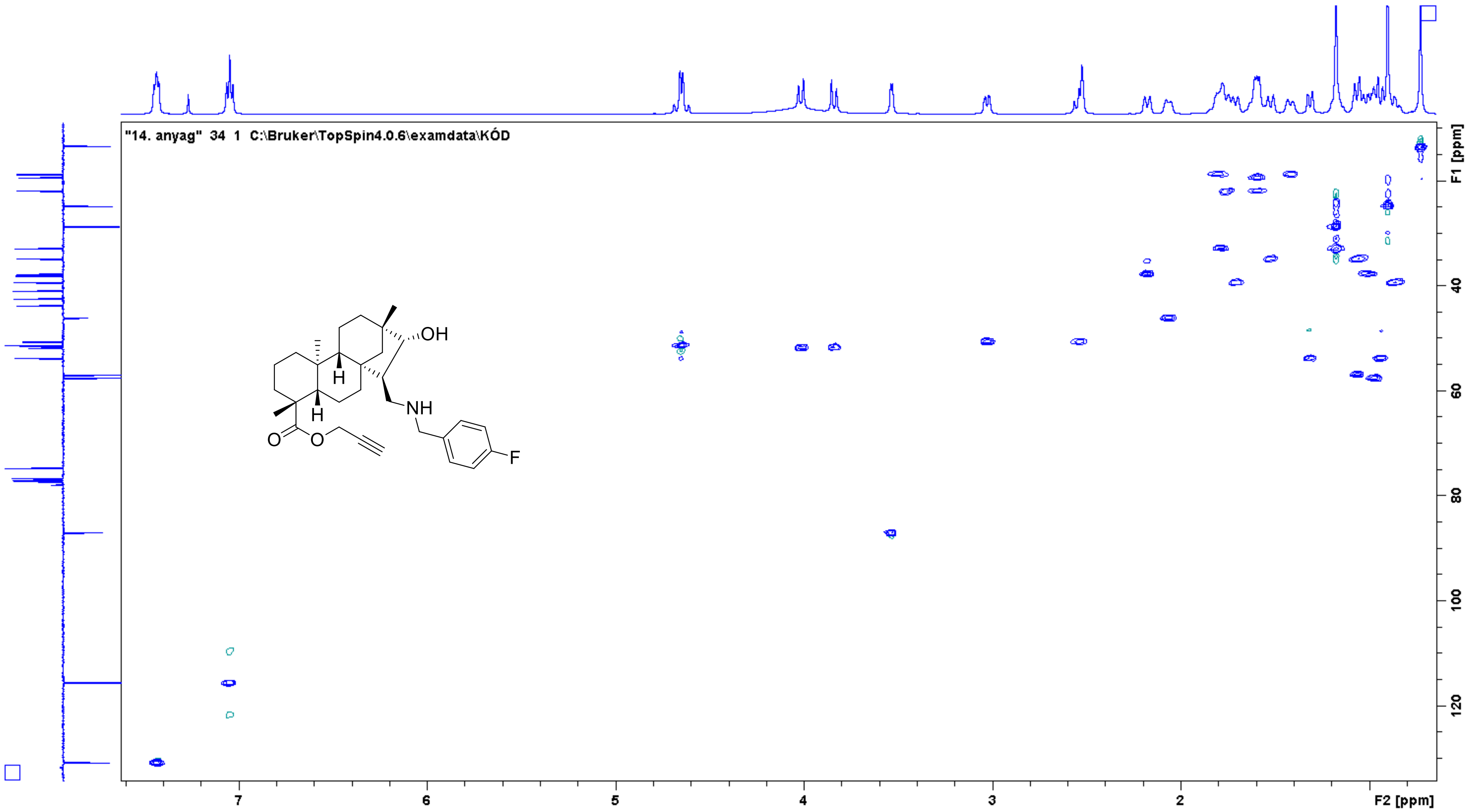

Figure S65

HMBC of compound (4*R*,4a*S*,6a*S*,7*R*,8*R*,9*S*,11b*S*)-Prop-2-yn-1-yl 7-(((4-fluorobenzyl)amino)methyl)-8-hydroxy-4,9,11b-trimethyltetradecahydro-6a,9-methanocyclohepta[a]naphthalene-4-carboxylate **(14)**:

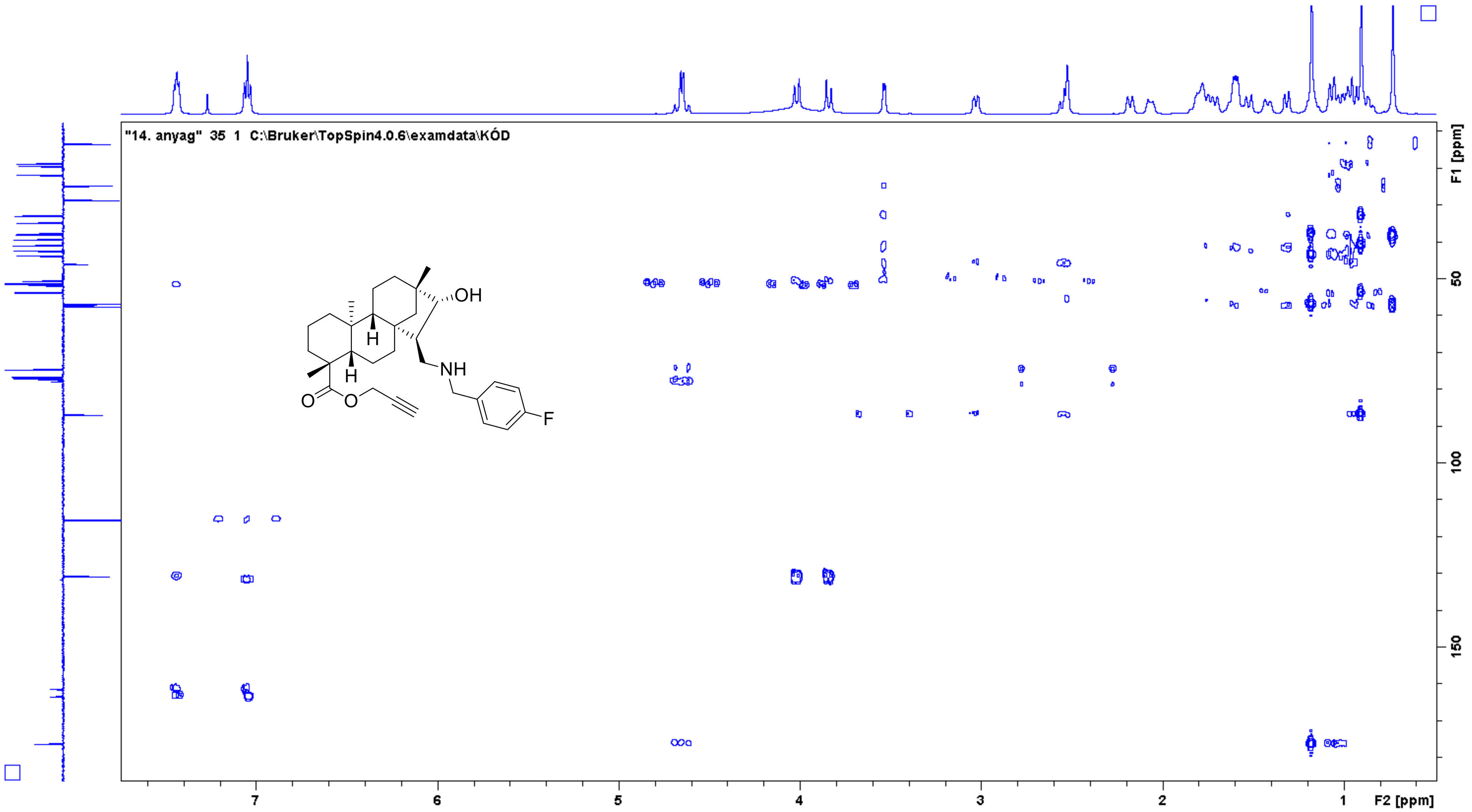

Figure S66

^{19}F-NMR of compound (4*R*,4a*S*,6a*S*,7*R*,8*R*,9*S*,11b*S*)-Prop-2-yn-1-yl 7-(((4-fluorobenzyl)amino)methyl)-8-hydroxy-4,9,11b-trimethyltetradecahydro-6a,9-methanocyclohepta[a]naphthalene-4-carboxylate **(14)**:

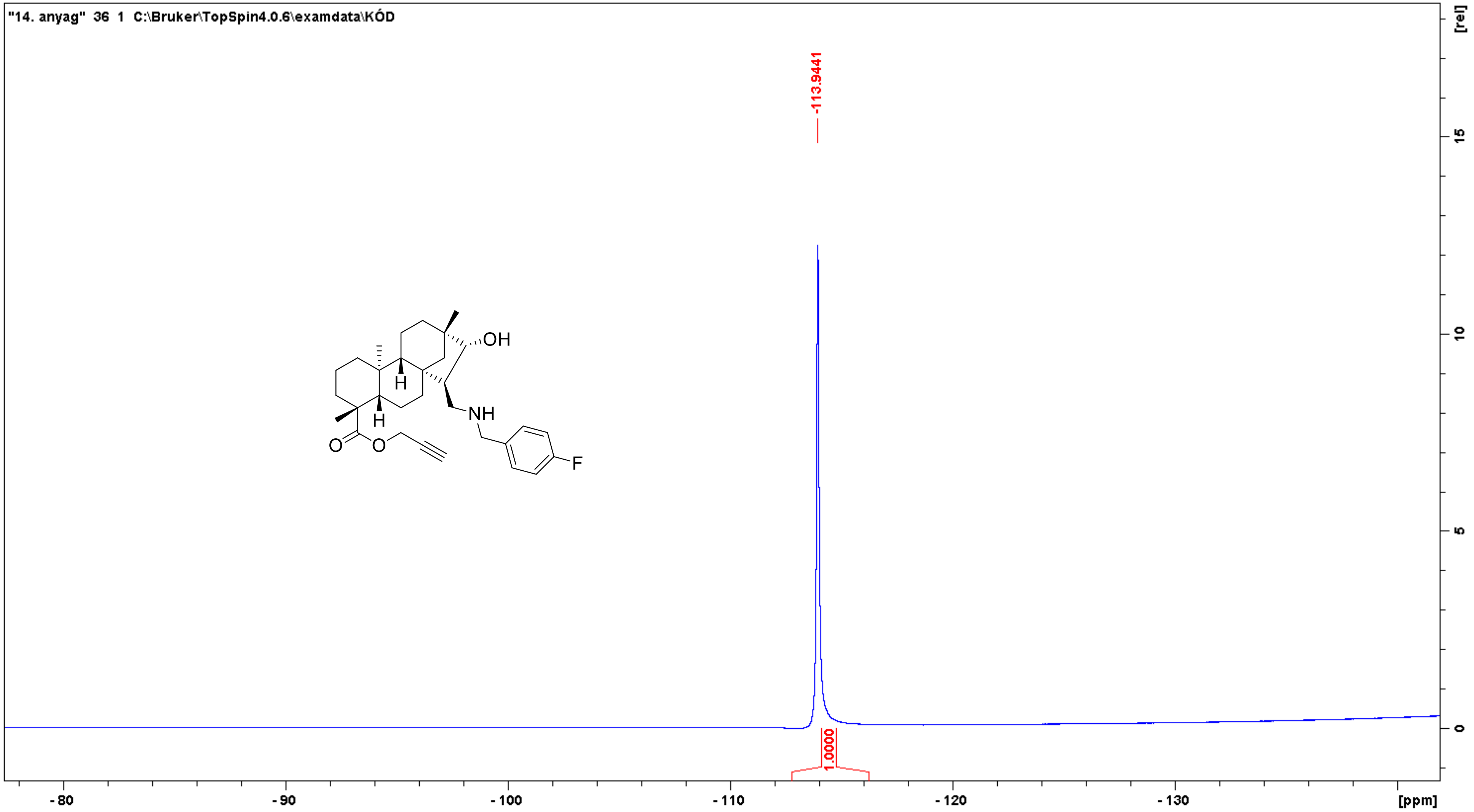

Figure S67

^{1}H-NMR of compound (4*R*,4a*S*,6a*S*,7*R*,8*R*,9*S*,11b*S*)-Benzyl 7-((((*R*)-1-(4-fluorophenyl)ethyl)amino)methyl)-8-hydroxy-4,9,11b-trimethyltetradecahydro-6a,9-methanocyclohepta[a]naphthalene-4-carboxylate **(15)**:

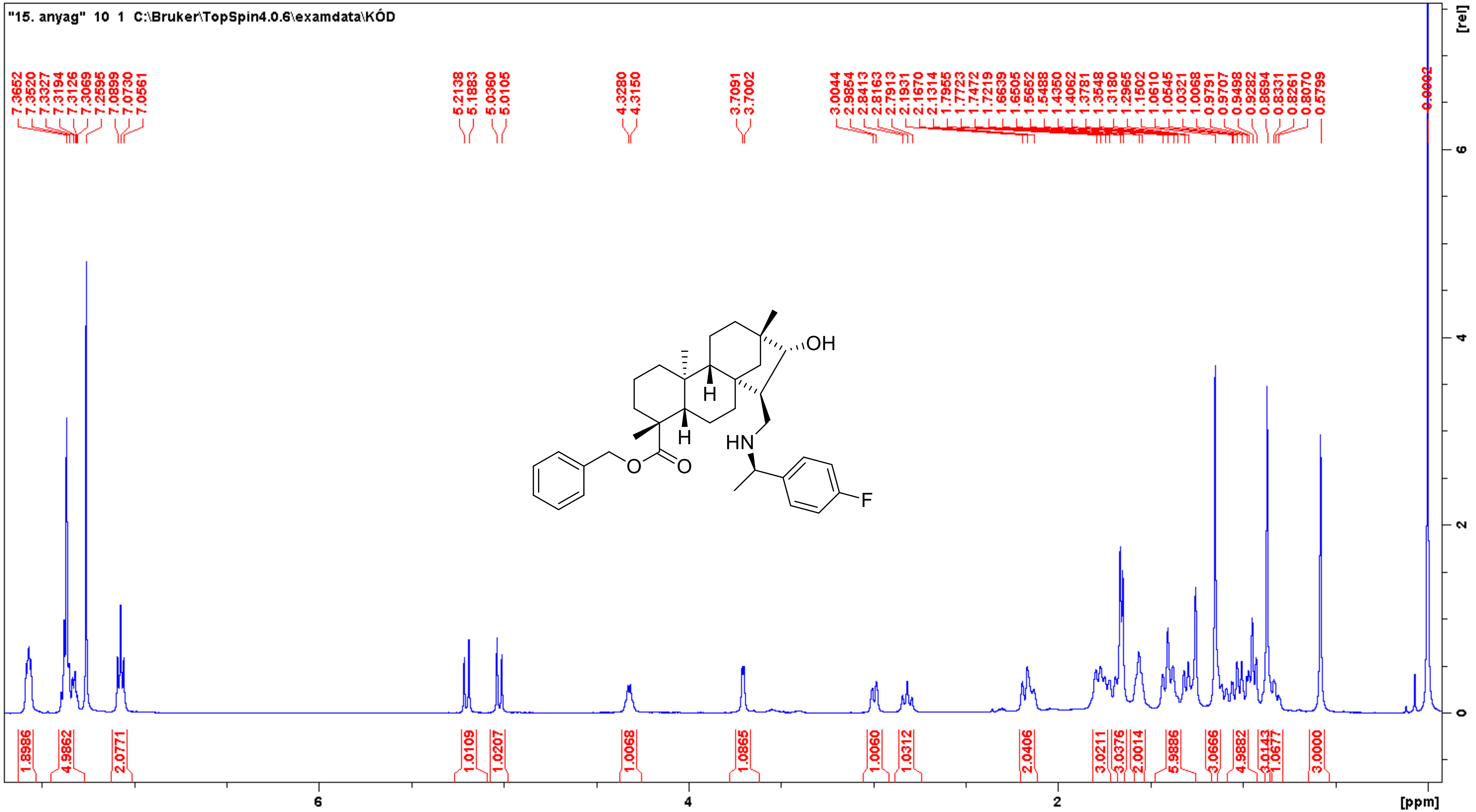

Figure S68

^{13}C-NMR of compound (4*R*,4a*S*,6a*S*,7*R*,8*R*,9*S*,11b*S*)-Benzyl 7-((((*R*)-1-(4-fluorophenyl)ethyl)amino)methyl)-8-hydroxy-4,9,11b-trimethyltetradecahydro-6a,9-methanocyclohepta[a]naphthalene-4-carboxylate **(15)**:

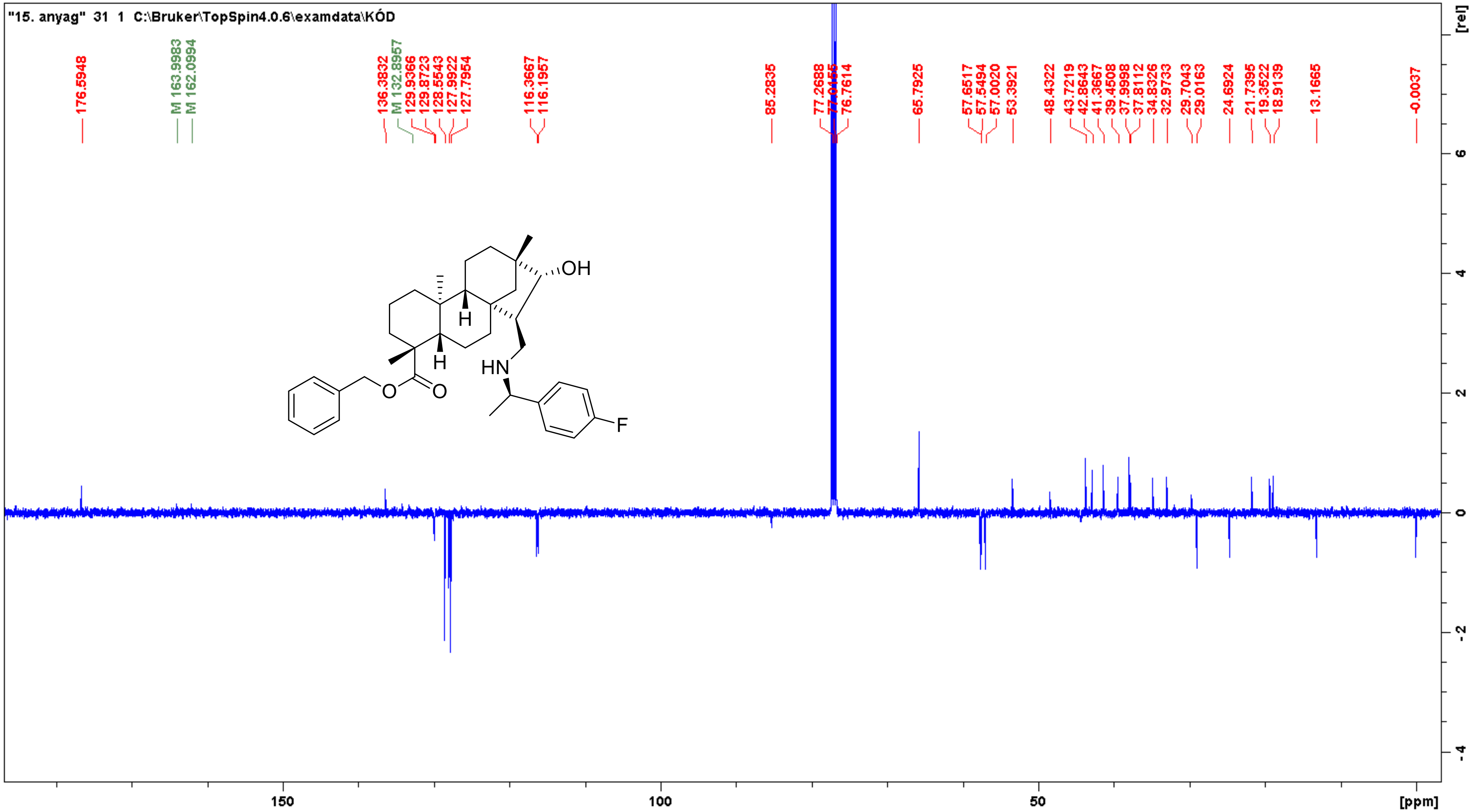

Figure S69

COSY of compound (4*R*,4a*S*,6a*S*,7*R*,8*R*,9*S*,11b*S*)-Benzyl 7-((((*R*)-1-(4-fluorophenyl)ethyl)amino)methyl)-8-hydroxy-4,9,11b-trimethyltetradecahydro-6a,9-methanocyclohepta[a]naphthalene-4-carboxylate **(15)**:

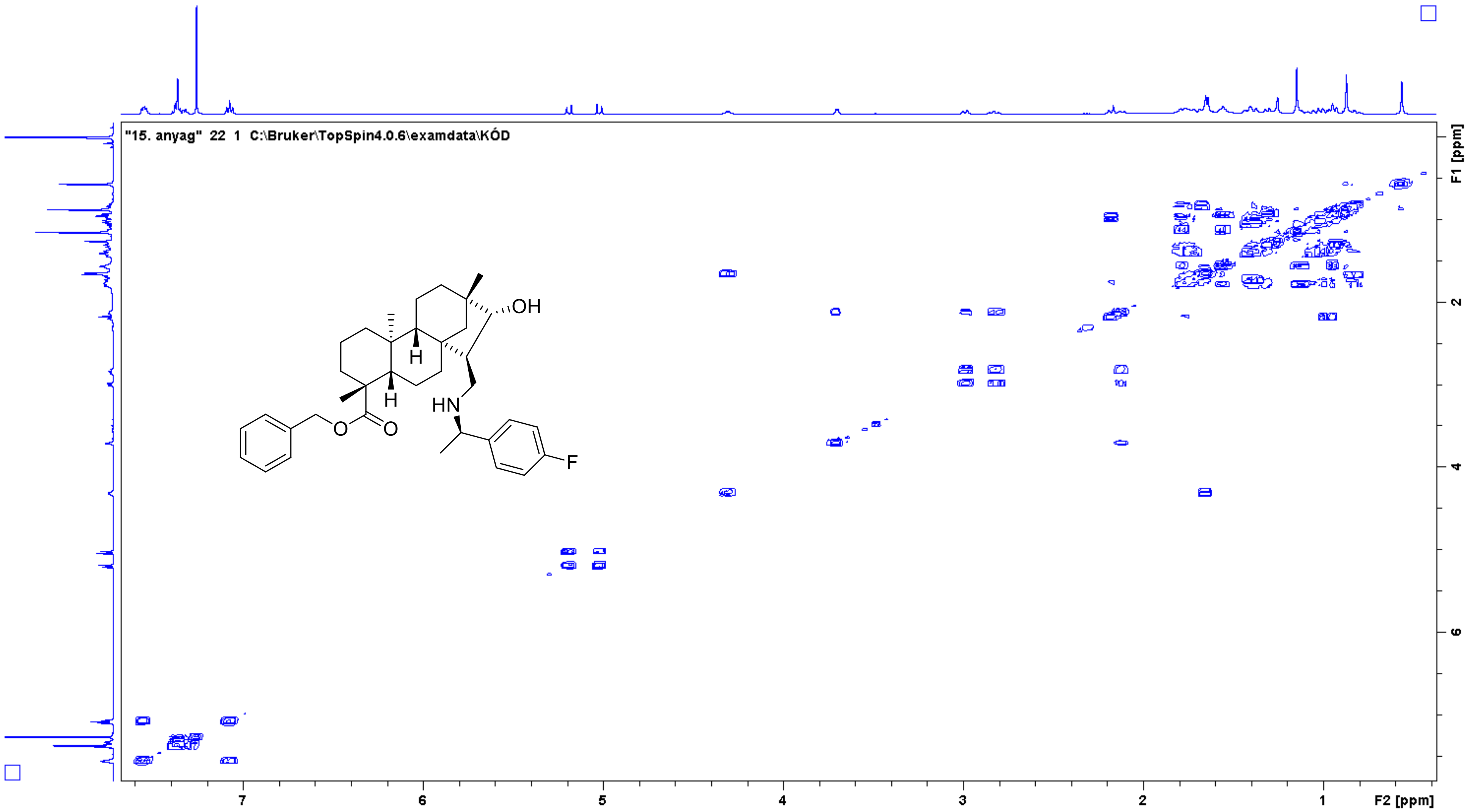

Figure S70

NOESY of compound (4*R*,4a*S*,6a*S*,7*R*,8*R*,9*S*,11b*S*)-Benzyl 7-((((*R*)-1-(4-fluorophenyl)ethyl)amino)methyl)-8-hydroxy-4,9,11b-trimethyltetradecahydro-6a,9-methanocyclohepta[a]naphthalene-4-carboxylate **(15)**:

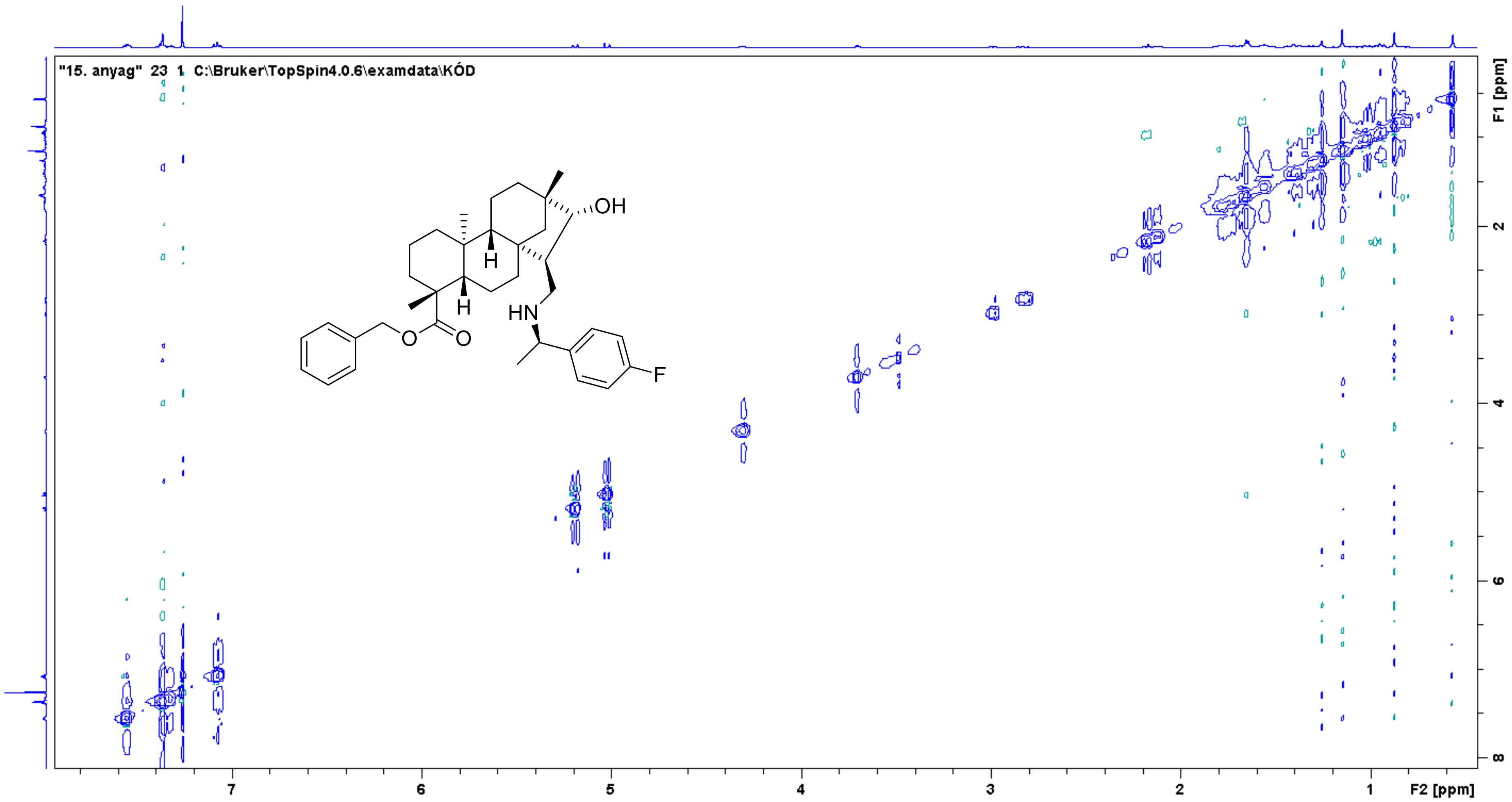

Figure S71

HSQC of compound (4*R*,4a*S*,6a*S*,7*R*,8*R*,9*S*,11b*S*)-Benzyl 7-((((*R*)-1-(4-fluorophenyl)ethyl)amino)methyl)-8-hydroxy-4,9,11b-trimethyltetradecahydro-6a,9-methanocyclohepta[a]naphthalene-4-carboxylate **(15)**:

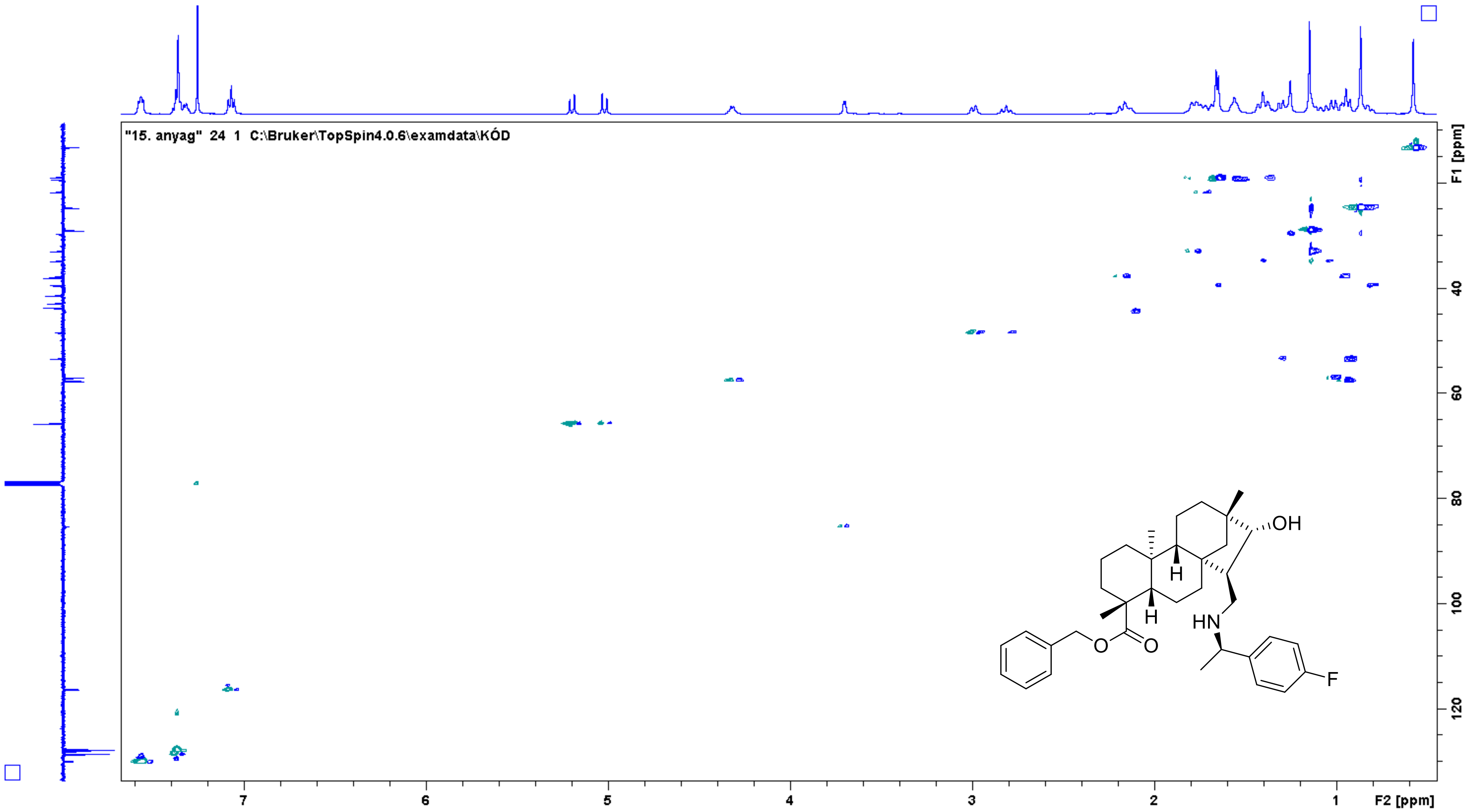

Figure S72

HSQC of compound (4*R*,4a*S*,6a*S*,7*R*,8*R*,9*S*,11b*S*)-Benzyl 7-((((*R*)-1-(4-fluorophenyl)ethyl)amino)methyl)-8-hydroxy-4,9,11b-trimethyltetradecahydro-6a,9-methanocyclohepta[a]naphthalene-4-carboxylate **(15)**:

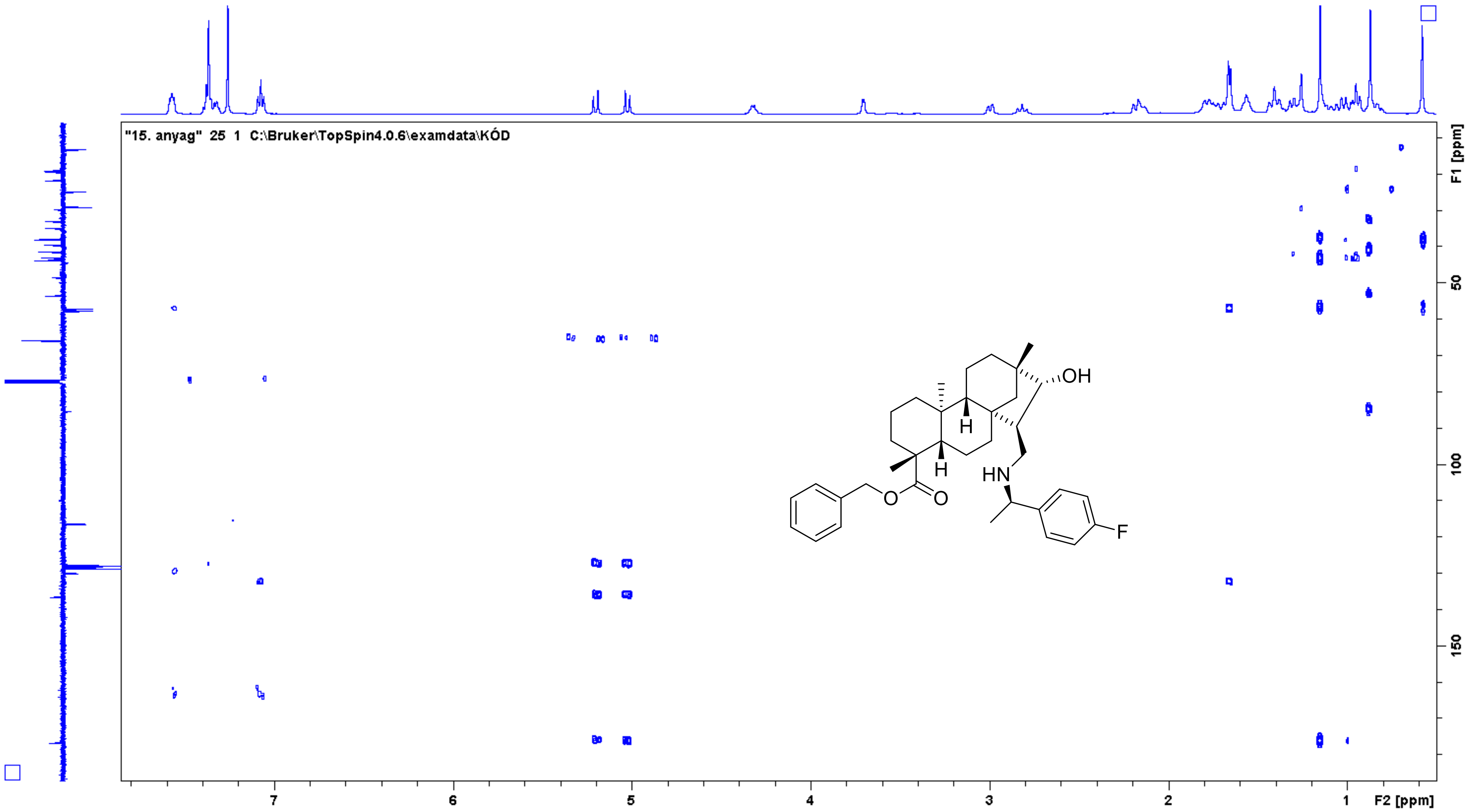

Figure S73

^{1}H-NMR of compound (4*R*,4a*S*,6a*S*,7*R*,8*R*,9*S*,11b*S*)-Benzyl 8-hydroxy-4,9,11b-trimethyl-7-((((*R*)-1-phenylpropyl)amino)methyl)tetradecahydro-6a,9-methanocyclohepta[a]naphthalene-4-carboxylate **(16)**:

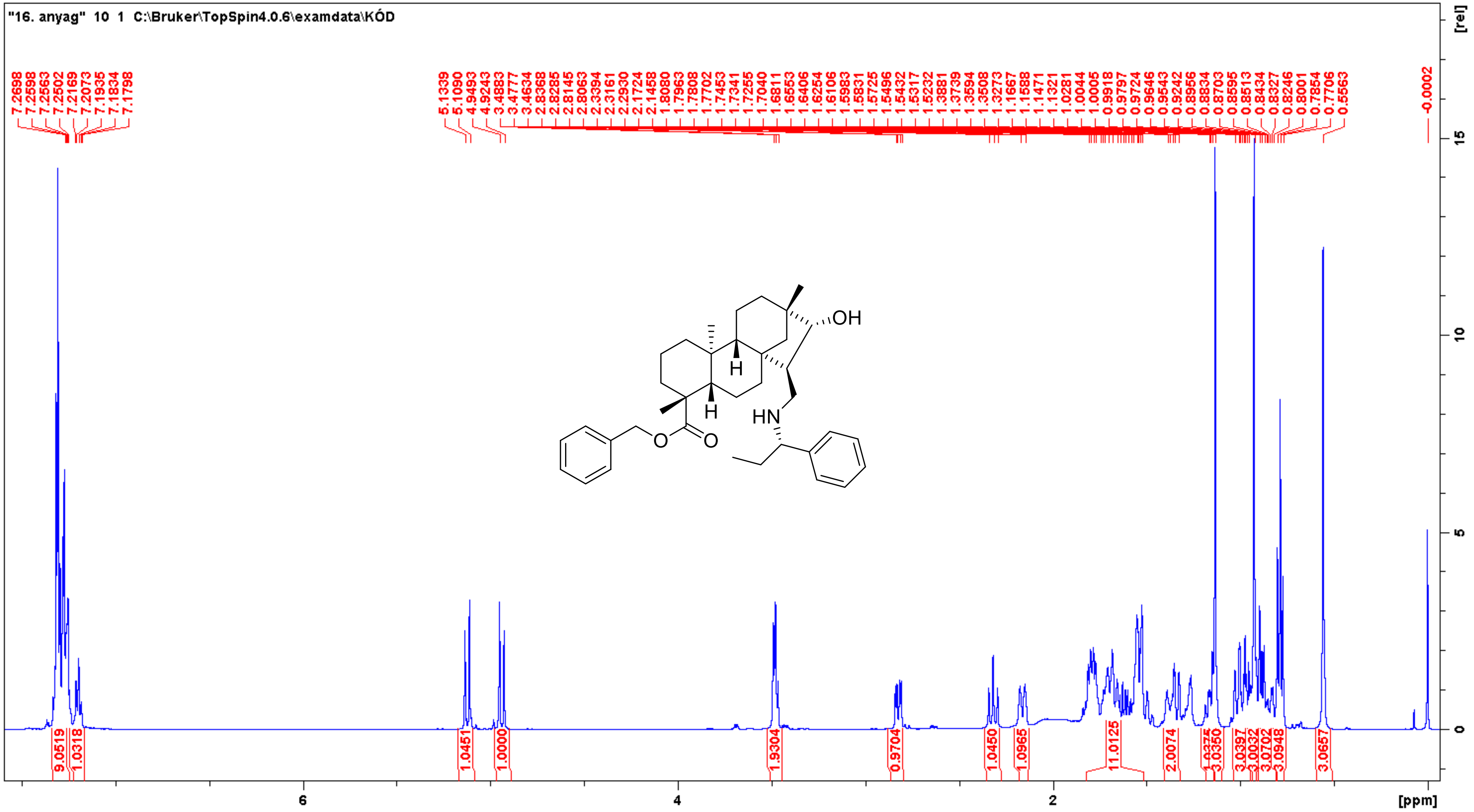

Figure S74

^{13}C-NMR of compound (4*R*,4a*S*,6a*S*,7*R*,8*R*,9*S*,11b*S*)-Benzyl 8-hydroxy-4,9,11b-trimethyl-7-((((*R*)-1-phenylpropyl)amino)methyl)tetradecahydro-6a,9-methanocyclohepta[a]naphthalene-4-carboxylate **(16)**:

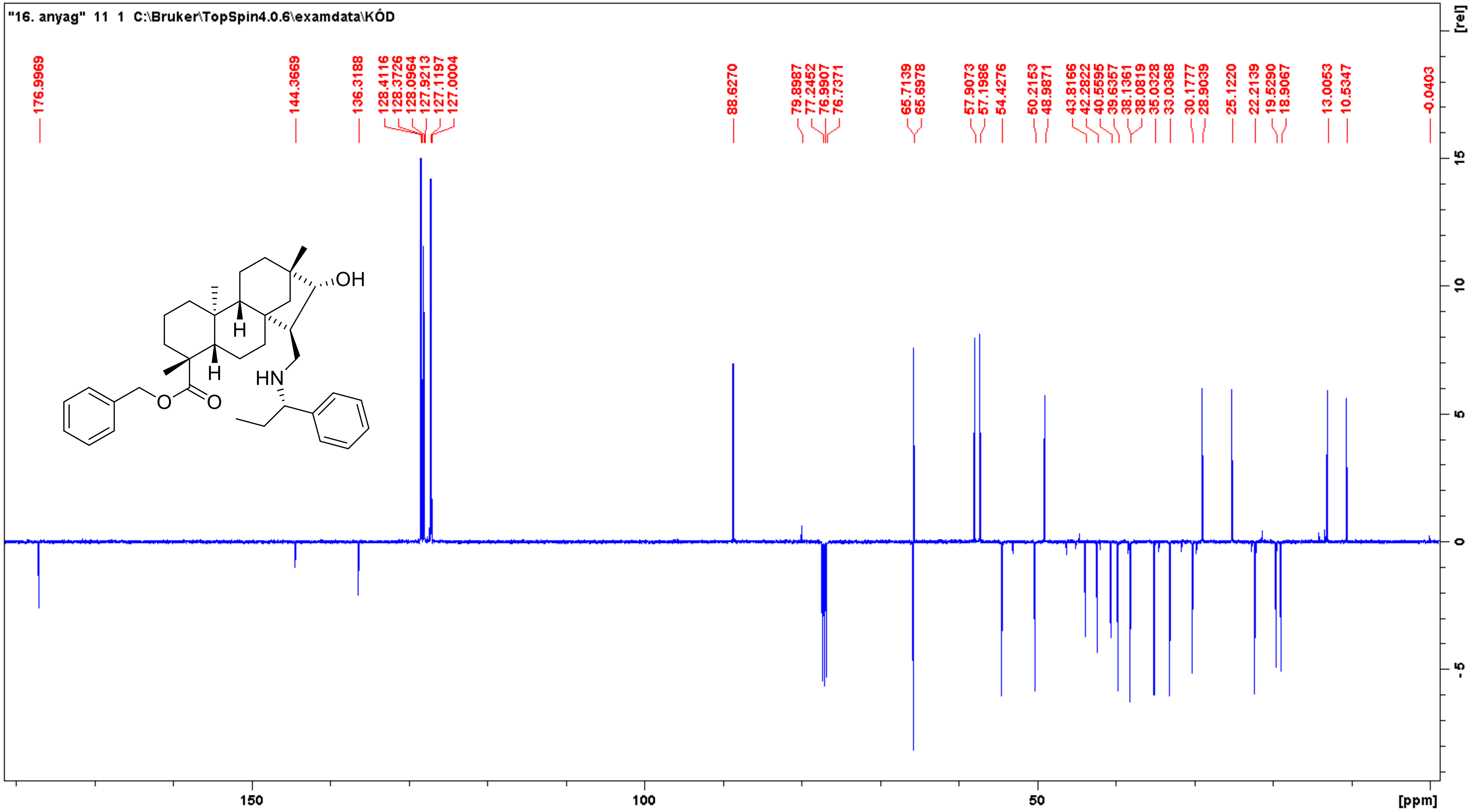

Figure S75

COSY of compound (4*R*,4a*S*,6a*S*,7*R*,8*R*,9*S*,11b*S*)-Benzyl 8-hydroxy-4,9,11b-trimethyl-7-((((*R*)-1-phenylpropyl)amino)methyl)tetradecahydro-6a,9-methanocyclohepta[a]naphthalene-4-carboxylate **(16)**:

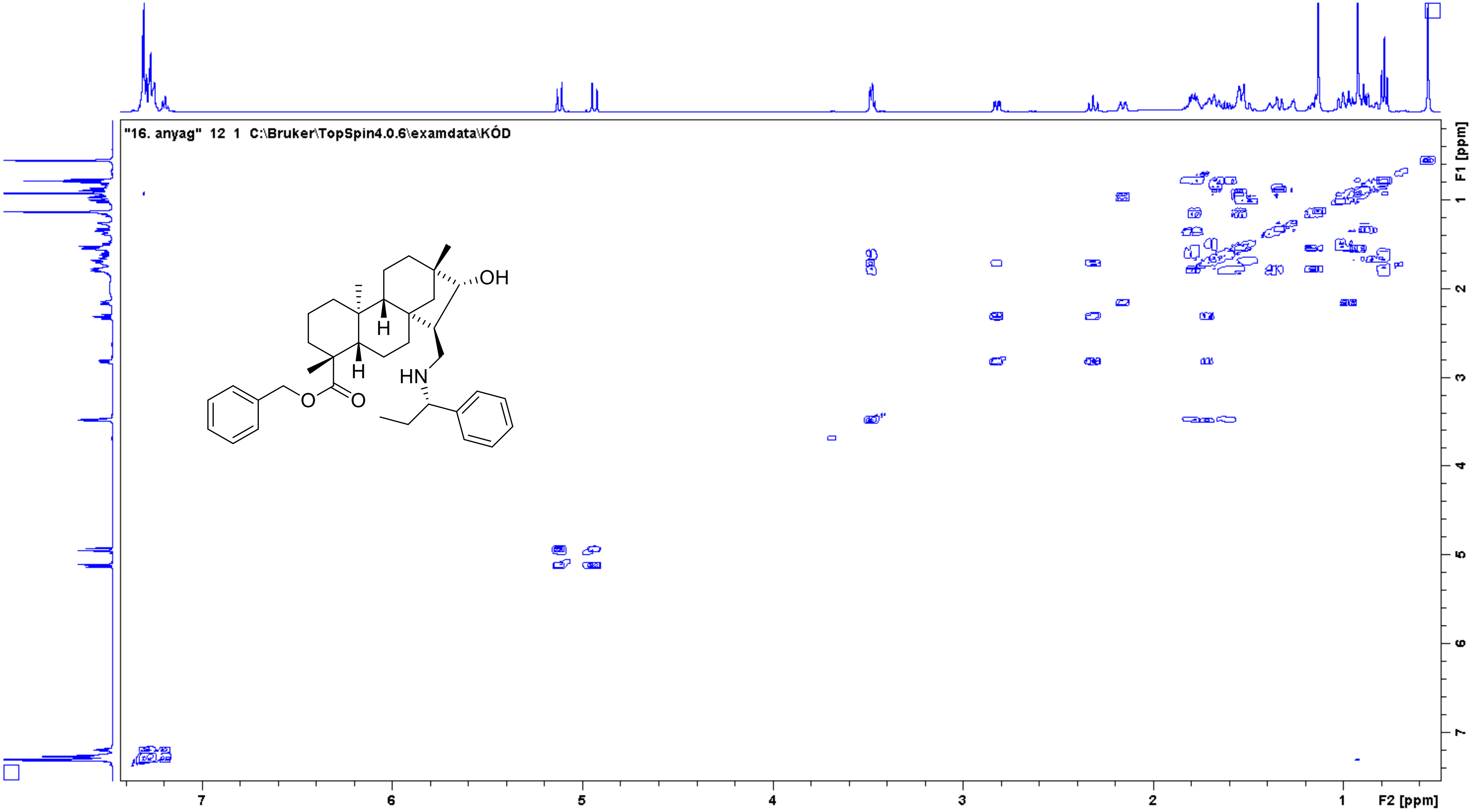

Figure S76

NOESY of compound (4*R*,4a*S*,6a*S*,7*R*,8*R*,9*S*,11b*S*)-Benzyl 8-hydroxy-4,9,11b-trimethyl-7-((((*R*)-1-phenylpropyl)amino)methyl)tetradecahydro-6a,9-methanocyclohepta[a]naphthalene-4-carboxylate **(16)**:

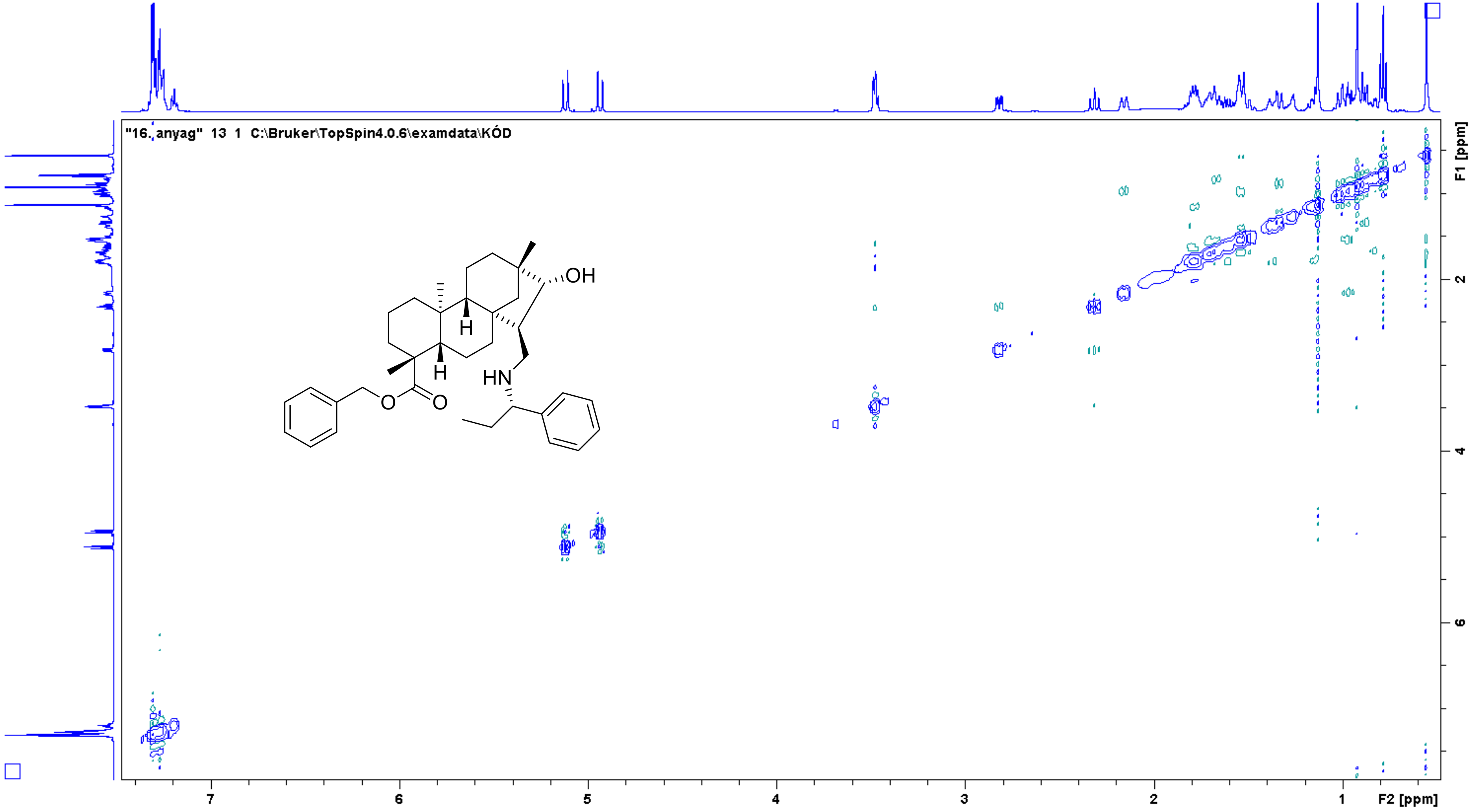

Figure S77

HSQC of compound (4*R*,4a*S*,6a*S*,7*R*,8*R*,9*S*,11b*S*)-Benzyl 8-hydroxy-4,9,11b-trimethyl-7-((((*R*)-1-phenylpropyl)amino)methyl)tetradecahydro-6a,9-methanocyclohepta[a]naphthalene-4-carboxylate **(16)**:

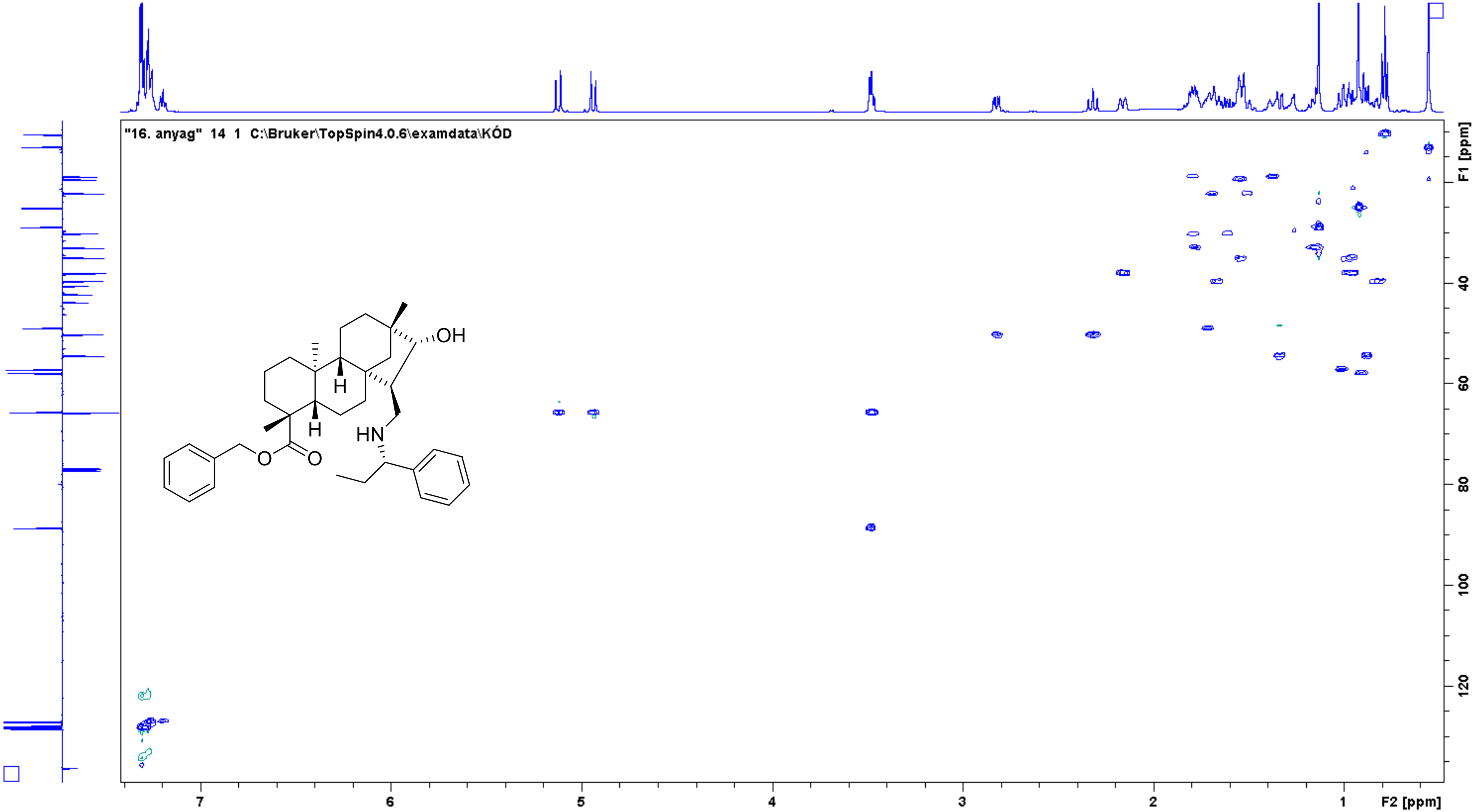

Figure S78

HMBC of compound (4*R*,4a*S*,6a*S*,7*R*,8*R*,9*S*,11b*S*)-Benzyl 8-hydroxy-4,9,11b-trimethyl-7-((((*R*)-1-phenylpropyl)amino)methyl)tetradecahydro-6a,9-methanocyclohepta[a]naphthalene-4-carboxylate **(16)**:

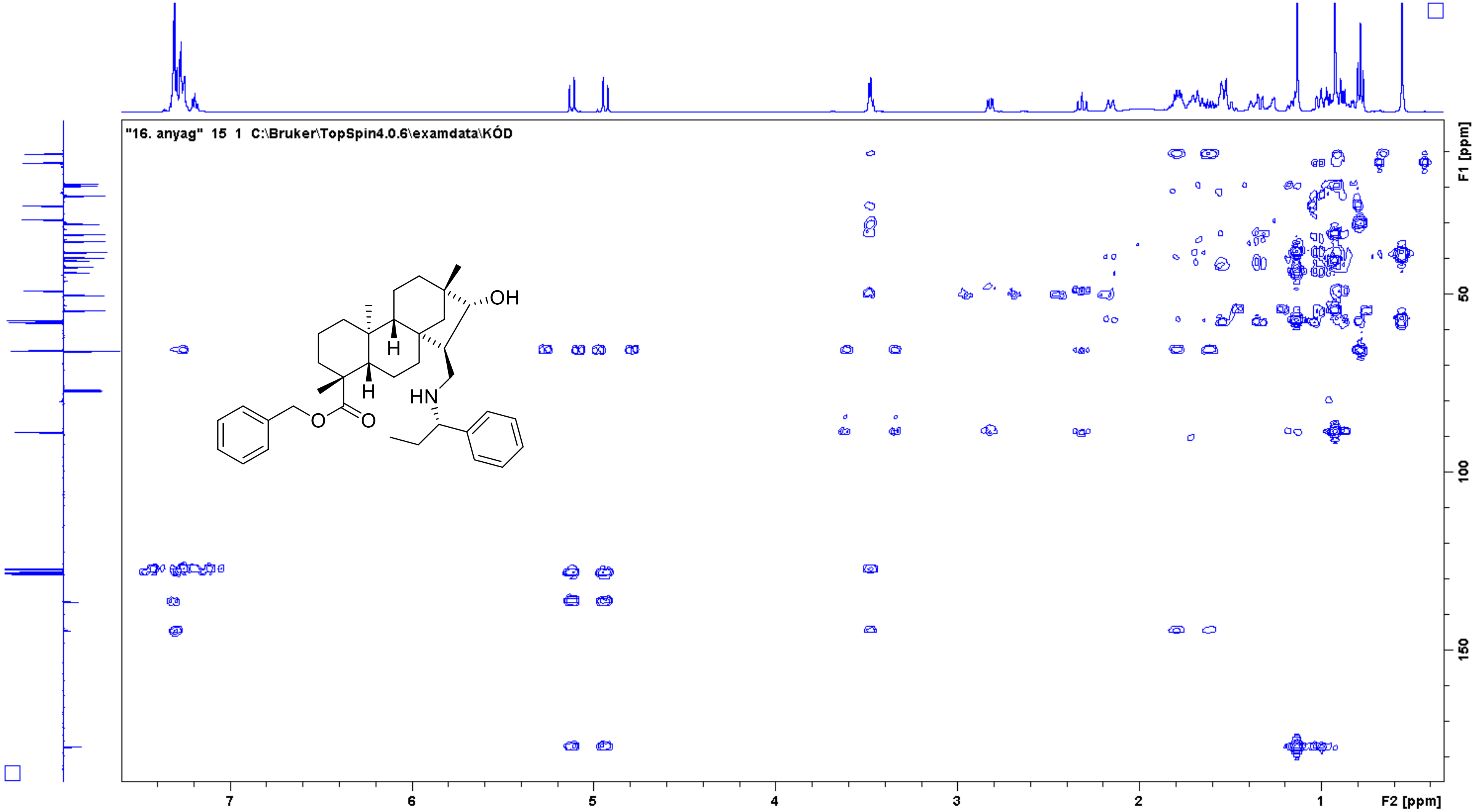

Figure S79

^{1}H-NMR of compound (4*R*,4a*S*,6a*S*,7*R*,8*R*,9*S*,11b*S*)-Benzyl 8-hydroxy-4,9,11b-trimethyl-7-((((*S*)-1-phenylpropyl)amino)methyl)tetradecahydro-6a,9-methanocyclohepta[a]naphthalene-4-carboxylate **(17)**:

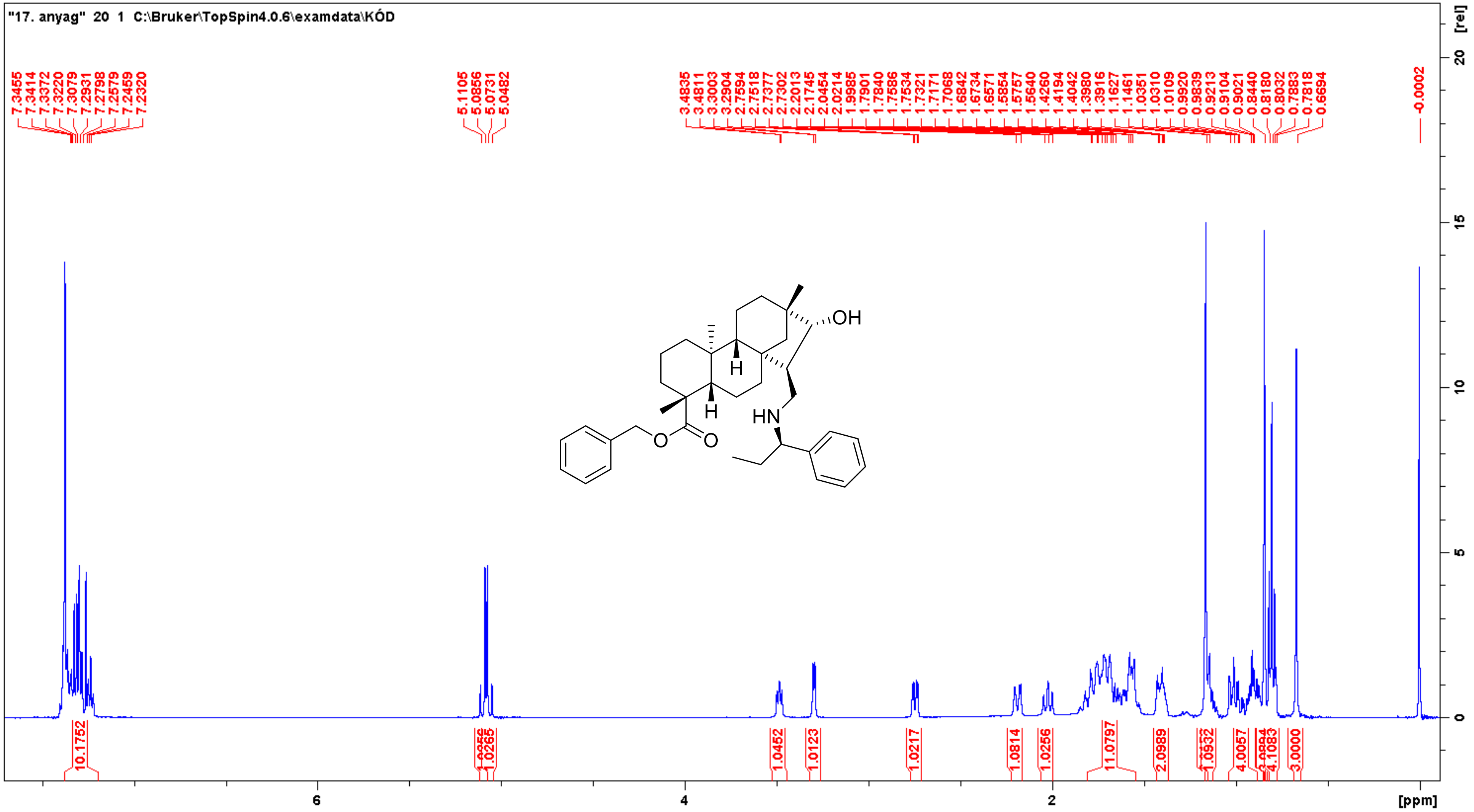

Figure S80

^{13}C-NMR of compound (4*R*,4a*S*,6a*S*,7*R*,8*R*,9*S*,11b*S*)-Benzyl 8-hydroxy-4,9,11b-trimethyl-7-((((*S*)-1-phenylpropyl)amino)methyl)tetradecahydro-6a,9-methanocyclohepta[a]naphthalene-4-carboxylate **(17)**:

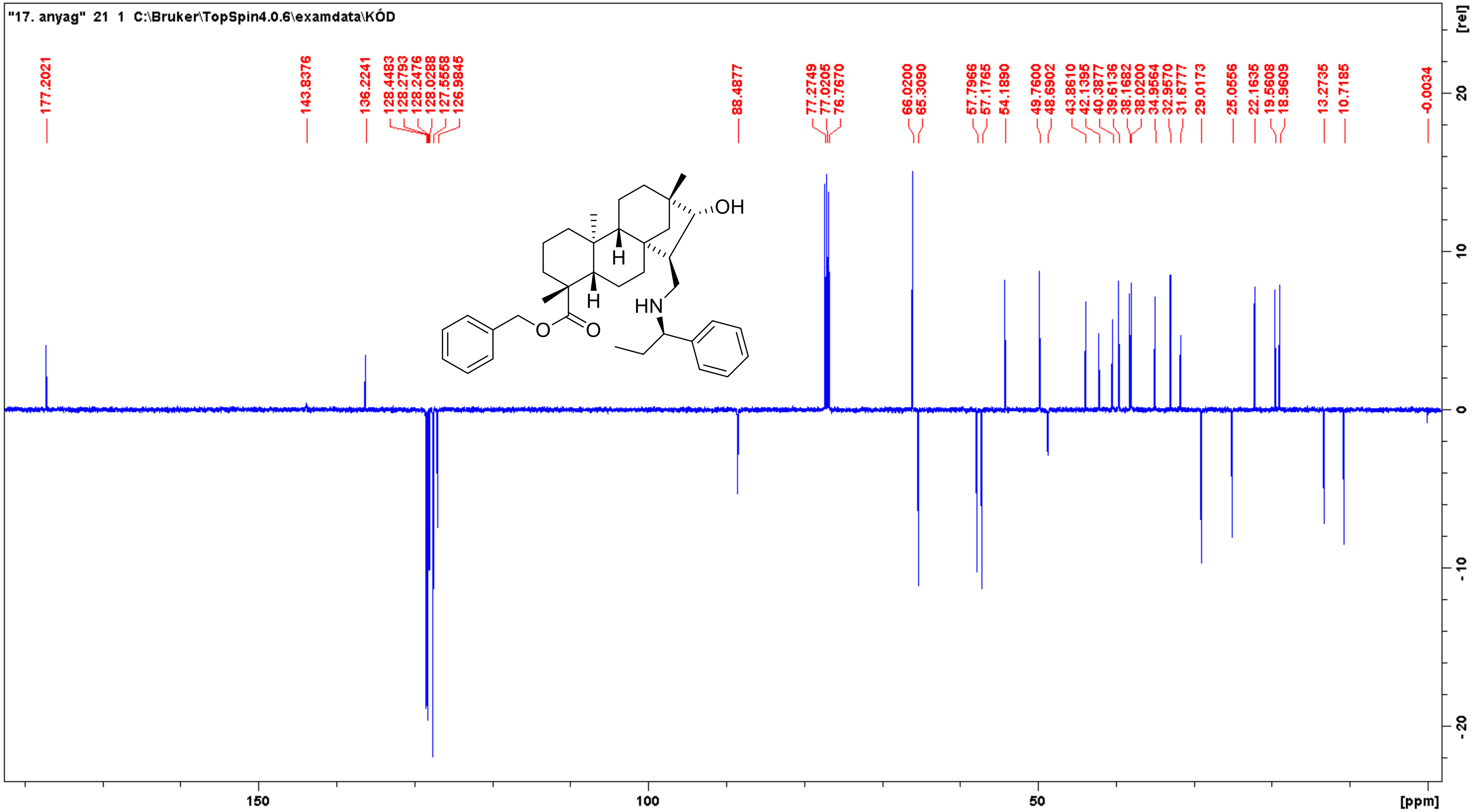

Figure S81

COSY of compound (4*R*,4a*S*,6a*S*,7*R*,8*R*,9*S*,11b*S*)-Benzyl 8-hydroxy-4,9,11b-trimethyl-7-((((*S*)-1-phenylpropyl)amino)methyl)tetradecahydro-6a,9-methanocyclohepta[a]naphthalene-4-carboxylate **(17)**:

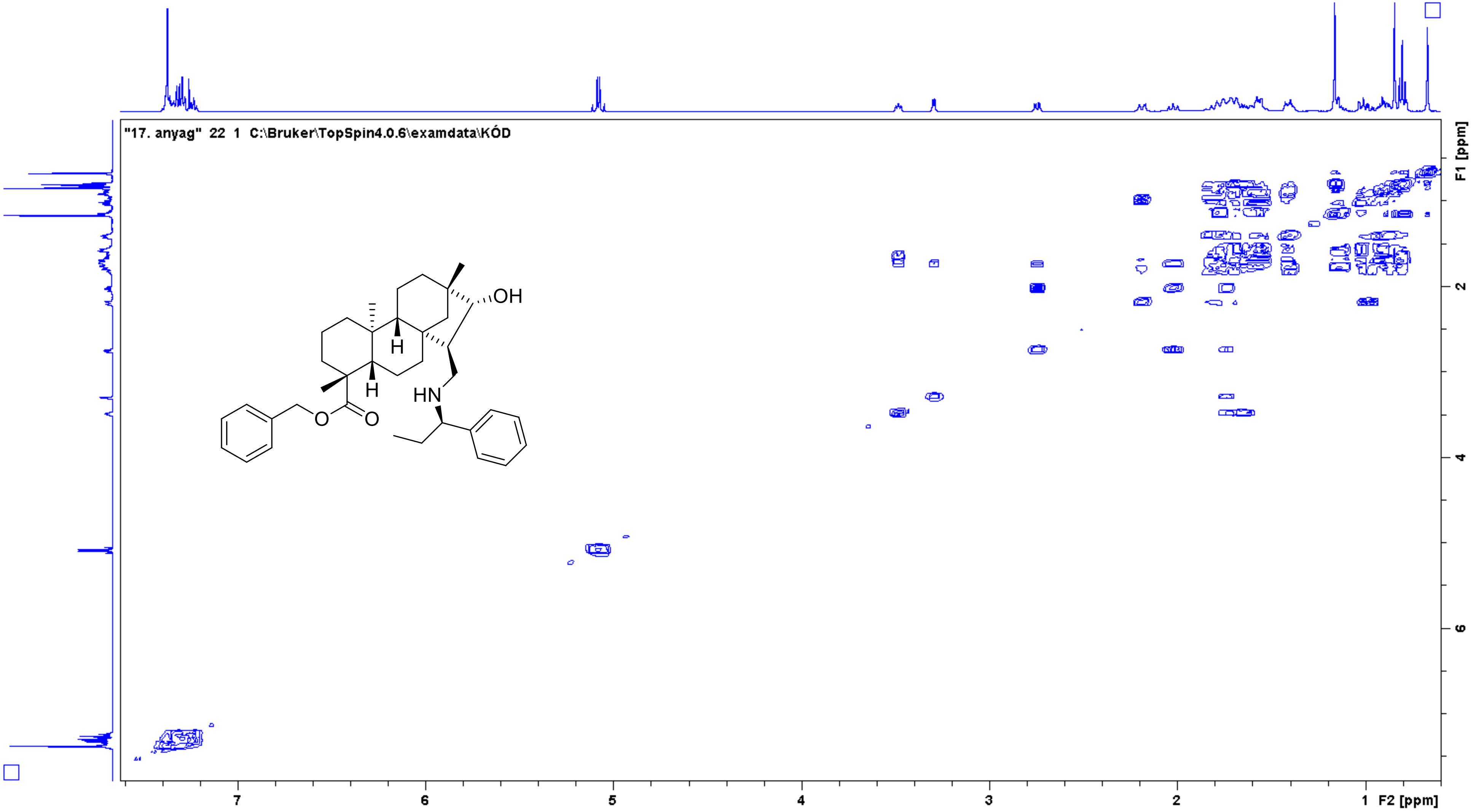

Figure S82

NOESY of compound (4*R*,4a*S*,6a*S*,7*R*,8*R*,9*S*,11b*S*)-Benzyl 8-hydroxy-4,9,11b-trimethyl-7-((((*S*)-1-phenylpropyl)amino)methyl)tetradecahydro-6a,9-methanocyclohepta[a]naphthalene-4-carboxylate **(17)**:

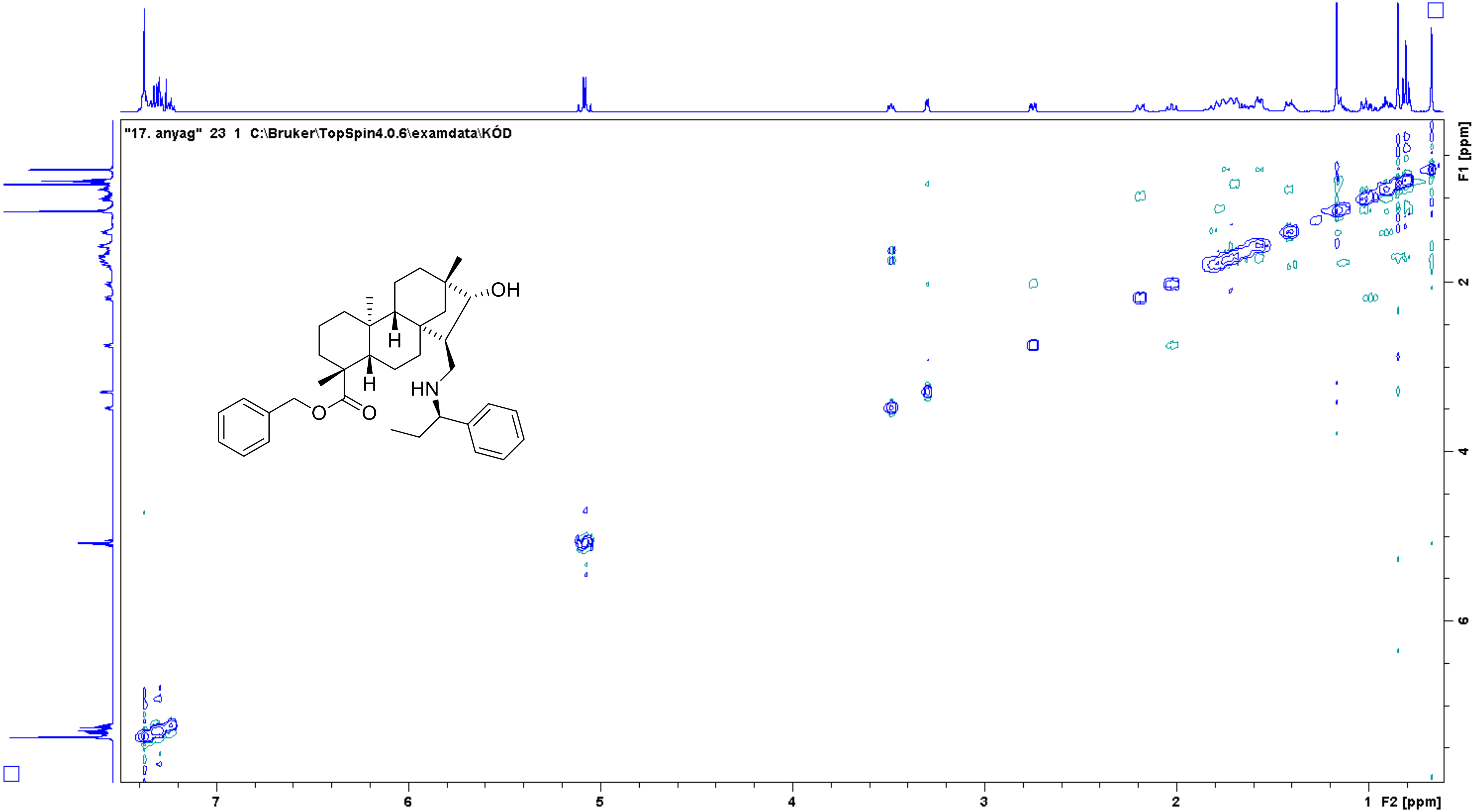

Figure S83

HSQC of compound (4*R*,4a*S*,6a*S*,7*R*,8*R*,9*S*,11b*S*)-Benzyl 8-hydroxy-4,9,11b-trimethyl-7-((((*S*)-1-phenylpropyl)amino)methyl)tetradecahydro-6a,9-methanocyclohepta[a]naphthalene-4-carboxylate **(17)**:

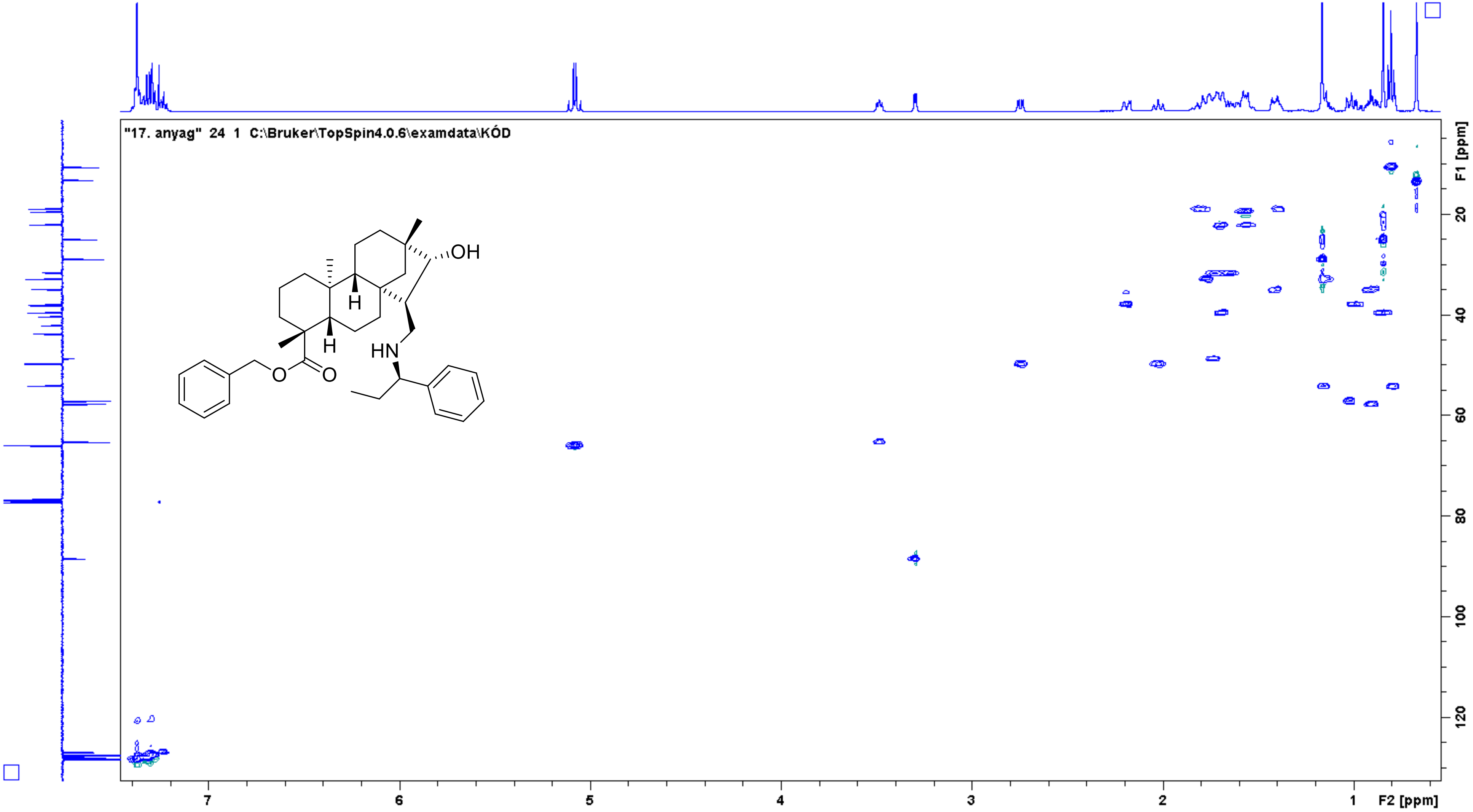

Figure S84

HMBC of compound (4*R*,4a*S*,6a*S*,7*R*,8*R*,9*S*,11b*S*)-Benzyl 8-hydroxy-4,9,11b-trimethyl-7-((((*S*)-1-phenylpropyl)amino)methyl)tetradecahydro-6a,9-methanocyclohepta[a]naphthalene-4-carboxylate **(17)**:

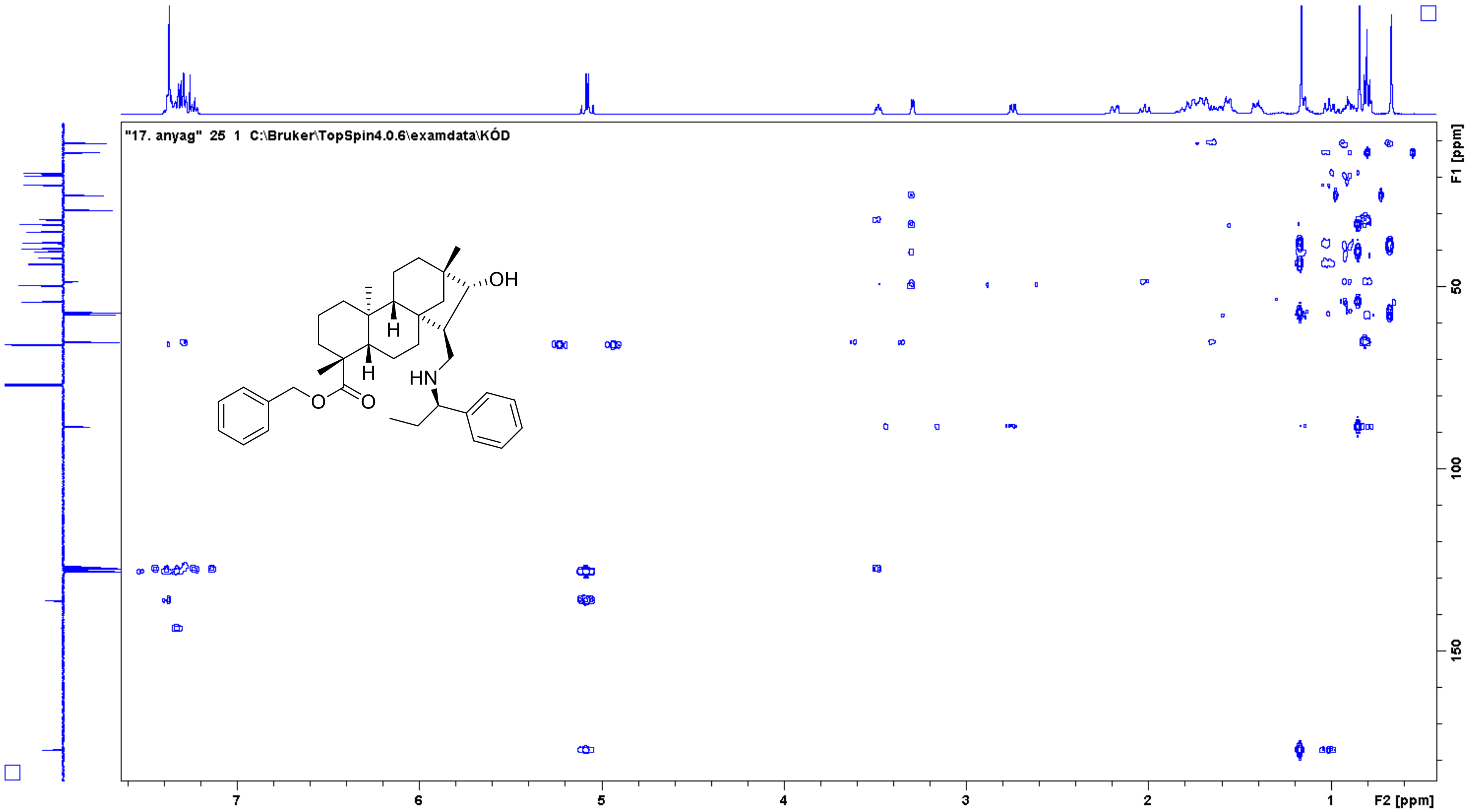

Figure S85

^{1}H-NMR of compound (4*R*,4a*S*,6a*S*,7*R*,8*R*,9*S*,11b*S*)-Benzyl 8-hydroxy-4,9,11b-trimethyl-7-((((*R*)-1-(naphthalen-1-yl)ethyl)amino)methyl)tetradecahydro-6a,9-methanocyclohepta[a]naphthalene-4-carboxylate **(18)**:

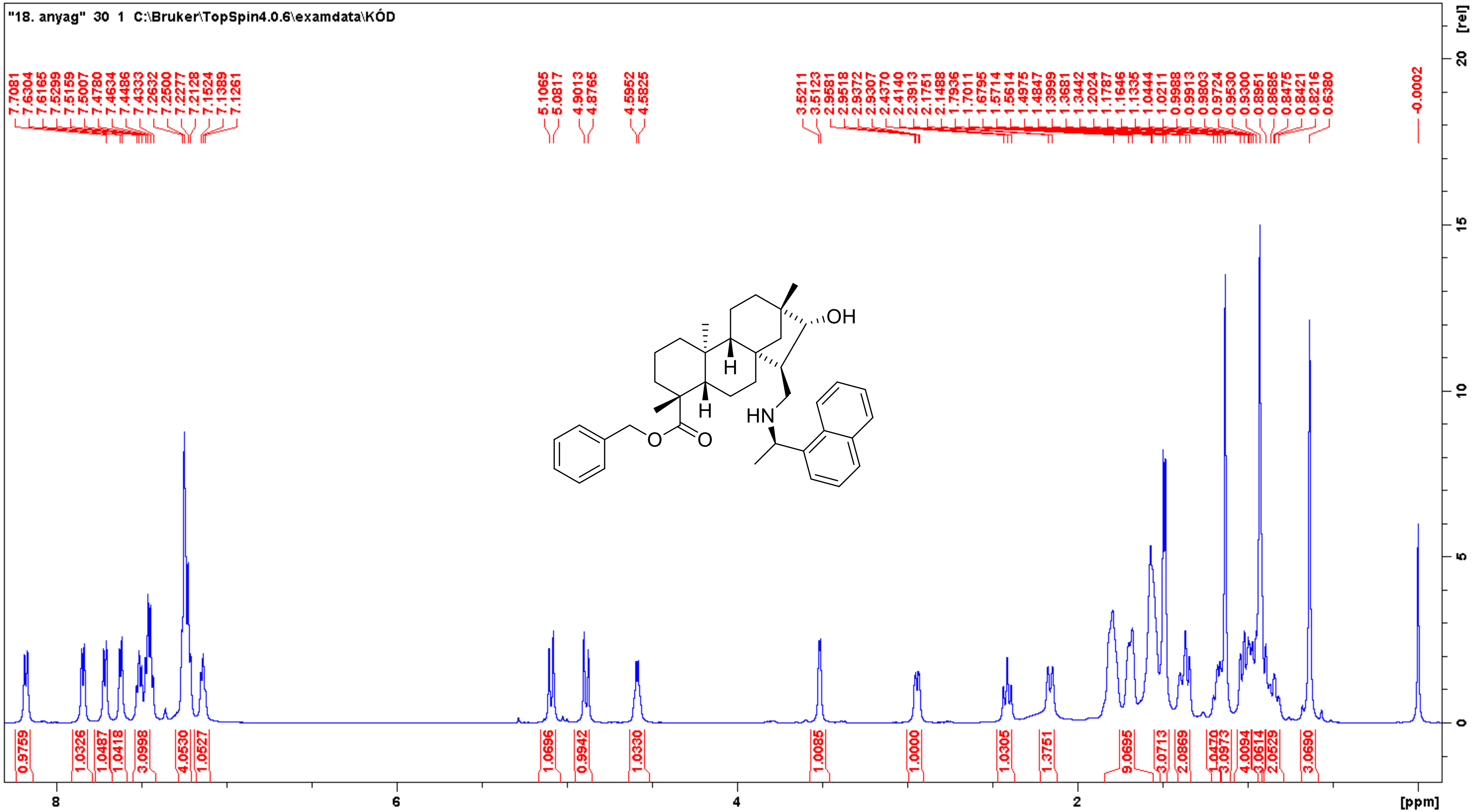

Figure S86

^{13}C-NMR of compound (4*R*,4a*S*,6a*S*,7*R*,8*R*,9*S*,11b*S*)-Benzyl 8-hydroxy-4,9,11b-trimethyl-7-((((*R*)-1-(naphthalen-1-yl)ethyl)amino)methyl)tetradecahydro-6a,9-methanocyclohepta[a]naphthalene-4-carboxylate **(18)**:

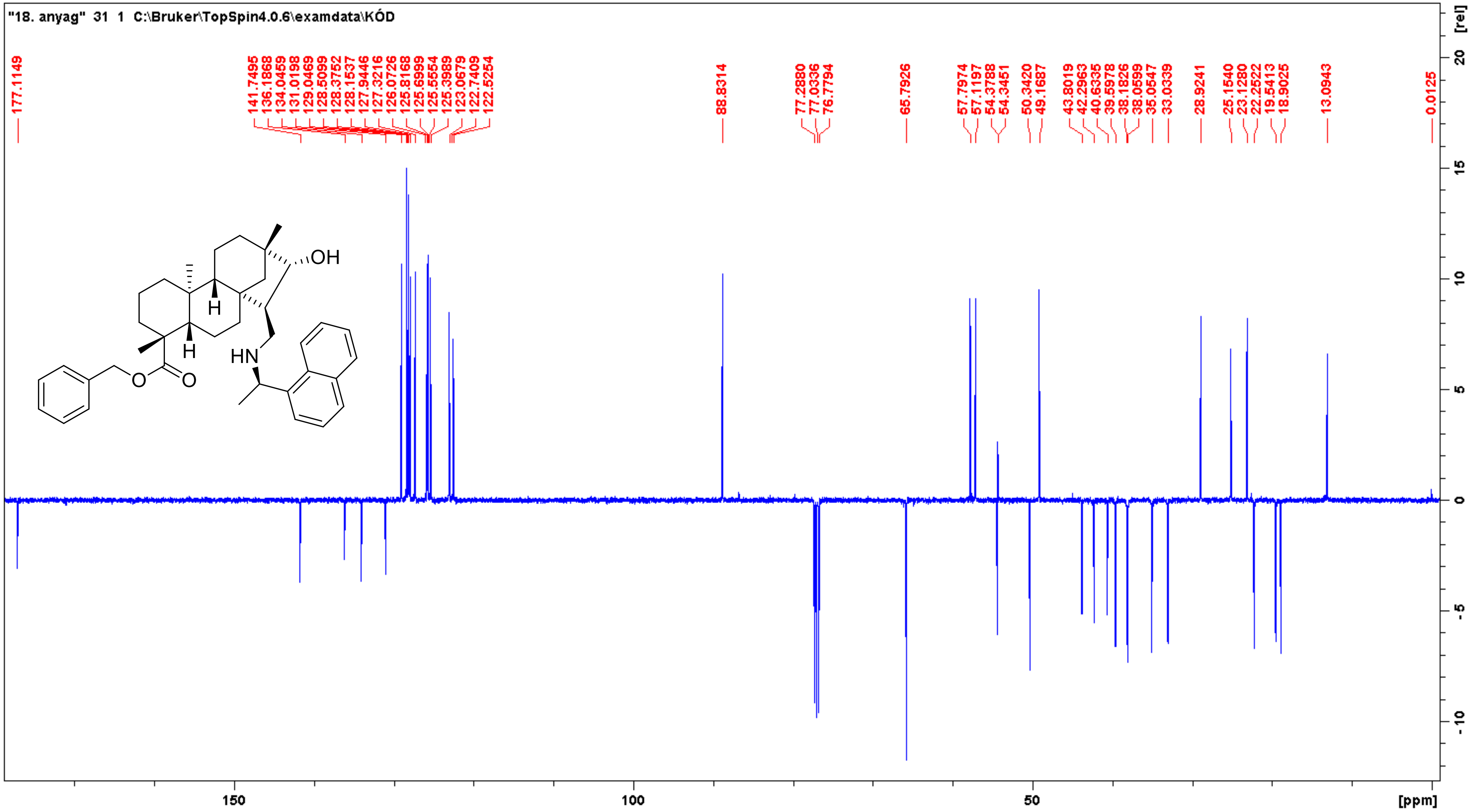

Figure S87

COSY of compound (4*R*,4a*S*,6a*S*,7*R*,8*R*,9*S*,11b*S*)-Benzyl 8-hydroxy-4,9,11b-trimethyl-7-((((*R*)-1-(naphthalen-1-yl)ethyl)amino)methyl)tetradecahydro-6a,9-methanocyclohepta[a]naphthalene-4-carboxylate **(18)**:

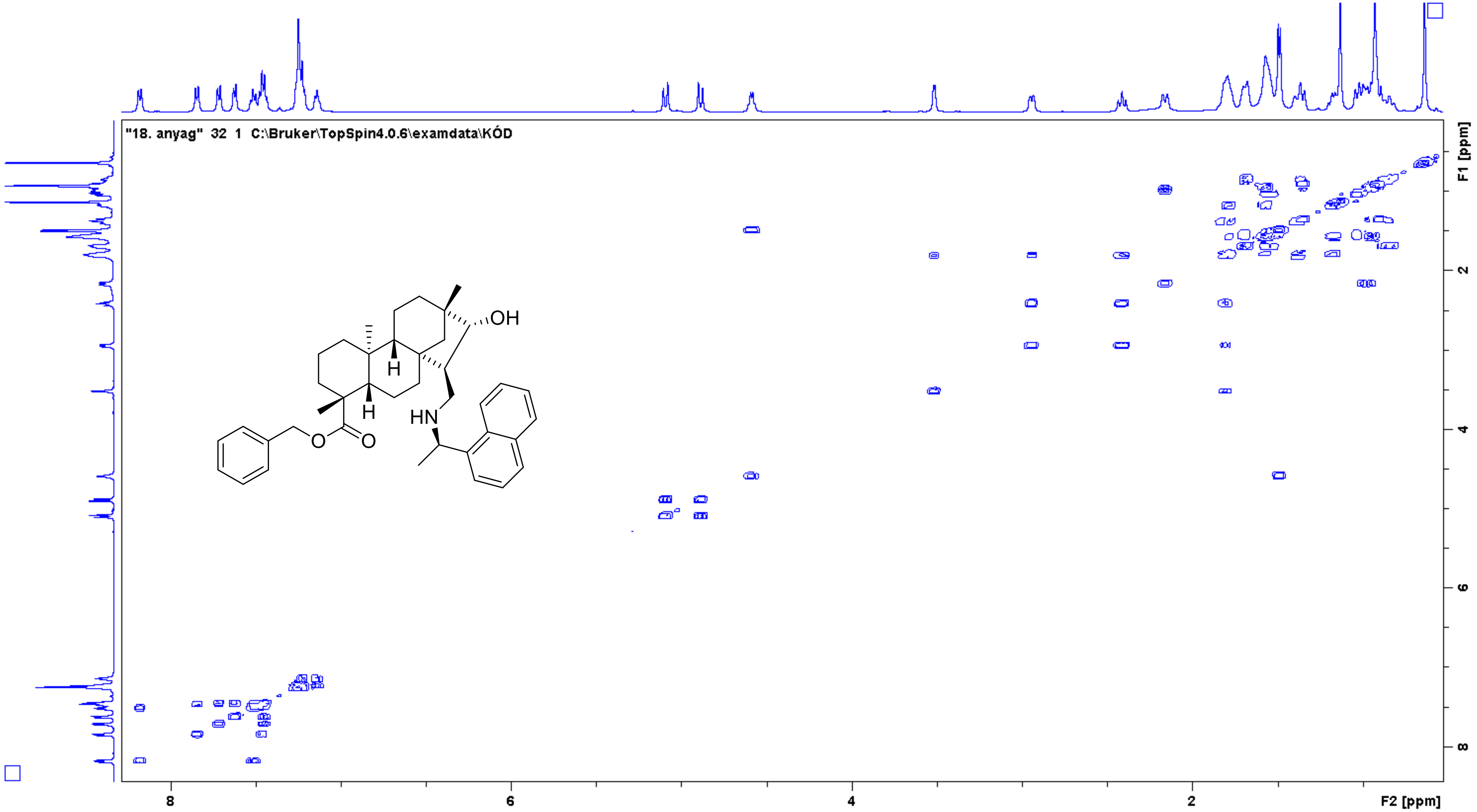

Figure S88

NOESY of compound (4*R*,4a*S*,6a*S*,7*R*,8*R*,9*S*,11b*S*)-Benzyl 8-hydroxy-4,9,11b-trimethyl-7-((((*R*)-1-(naphthalen-1-yl)ethyl)amino)methyl)tetradecahydro-6a,9-methanocyclohepta[a]naphthalene-4-carboxylate **(18)**:

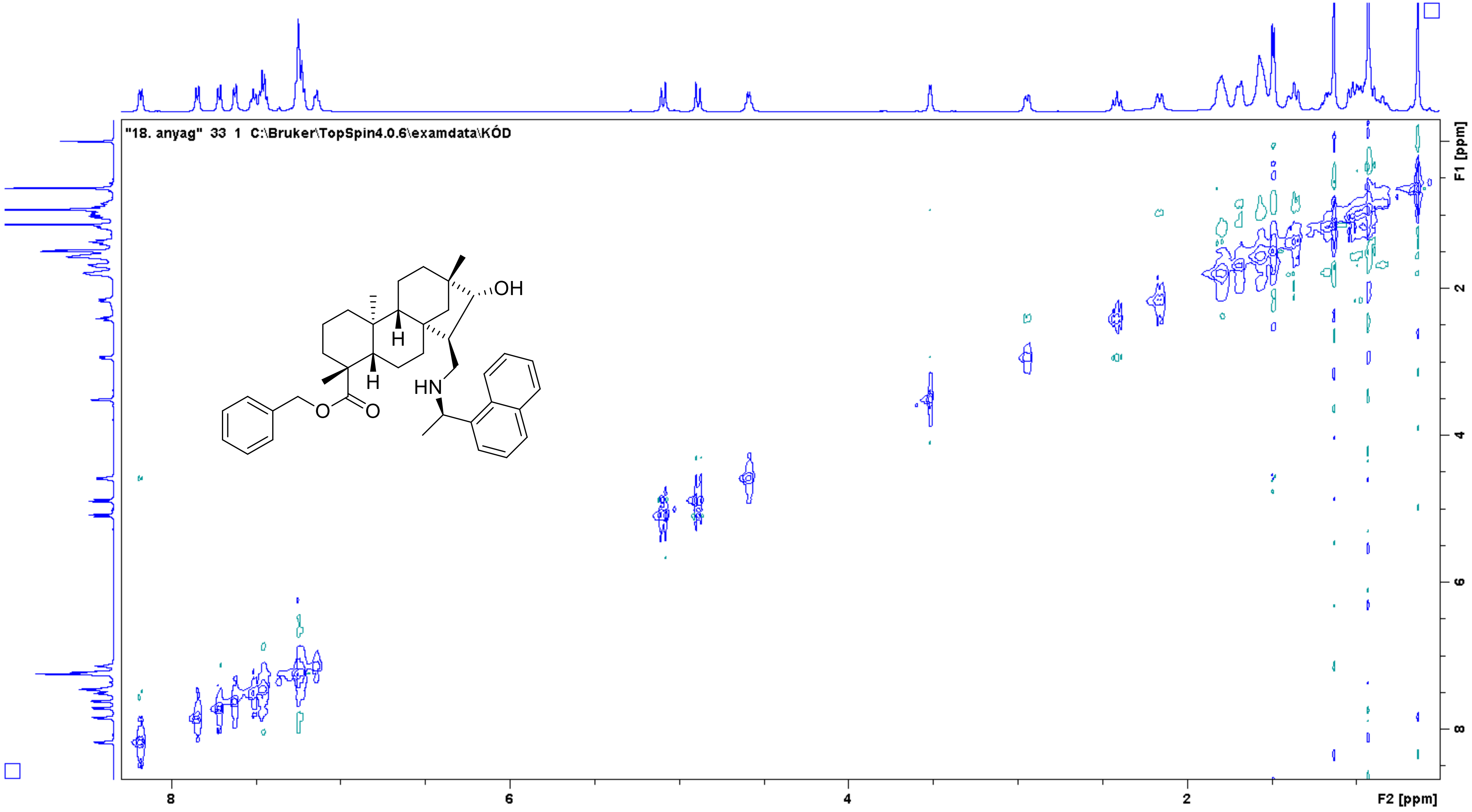

Figure S89

HSQC of compound (4*R*,4a*S*,6a*S*,7*R*,8*R*,9*S*,11b*S*)-Benzyl 8-hydroxy-4,9,11b-trimethyl-7-((((*R*)-1-(naphthalen-1-yl)ethyl)amino)methyl)tetradecahydro-6a,9-methanocyclohepta[a]naphthalene-4-carboxylate **(18)**:

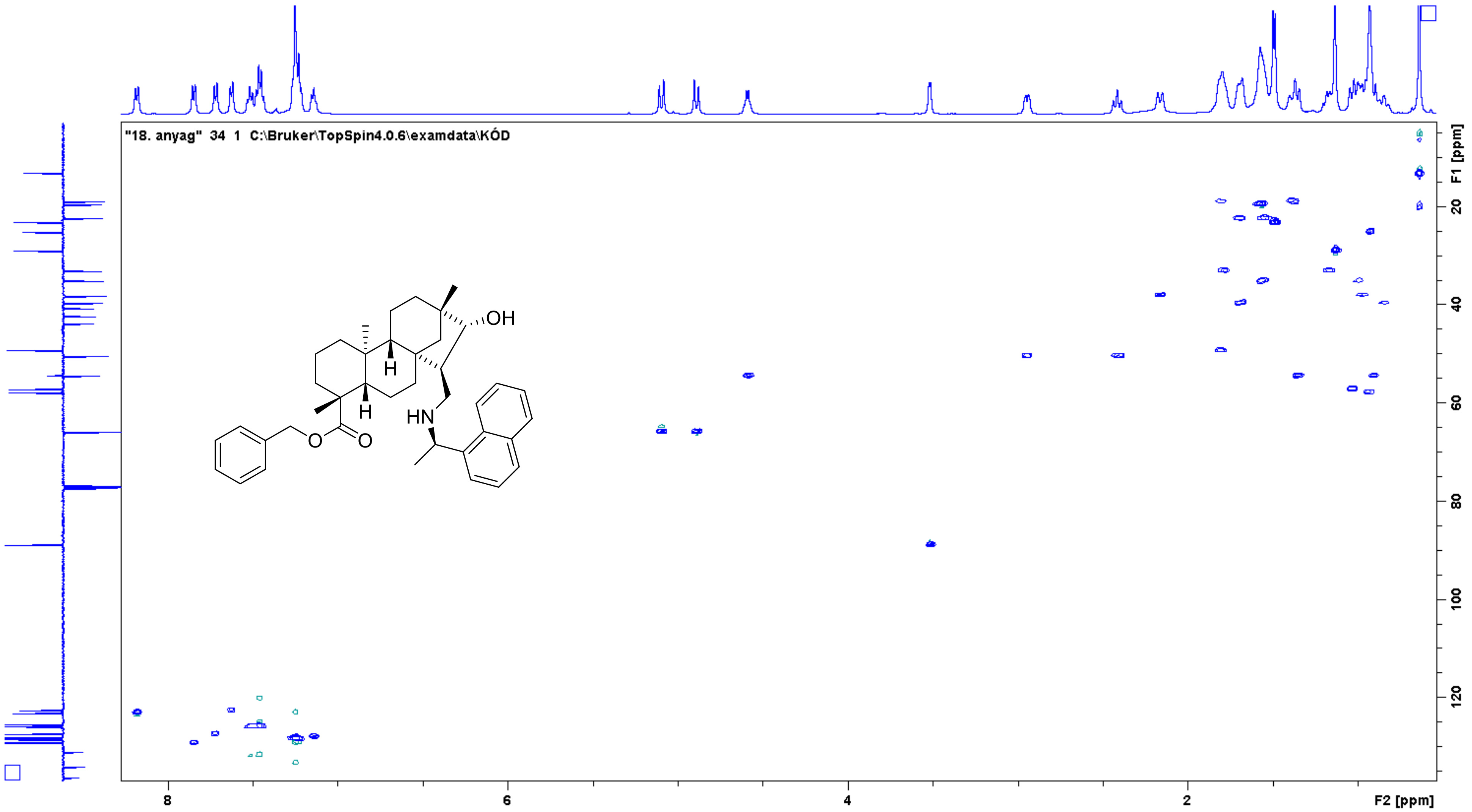

Figure S90

HMBC of compound (4*R*,4a*S*,6a*S*,7*R*,8*R*,9*S*,11b*S*)-Benzyl 8-hydroxy-4,9,11b-trimethyl-7-((((*R*)-1-(naphthalen-1-yl)ethyl)amino)methyl)tetradecahydro-6a,9-methanocyclohepta[a]naphthalene-4-carboxylate **(18)**:

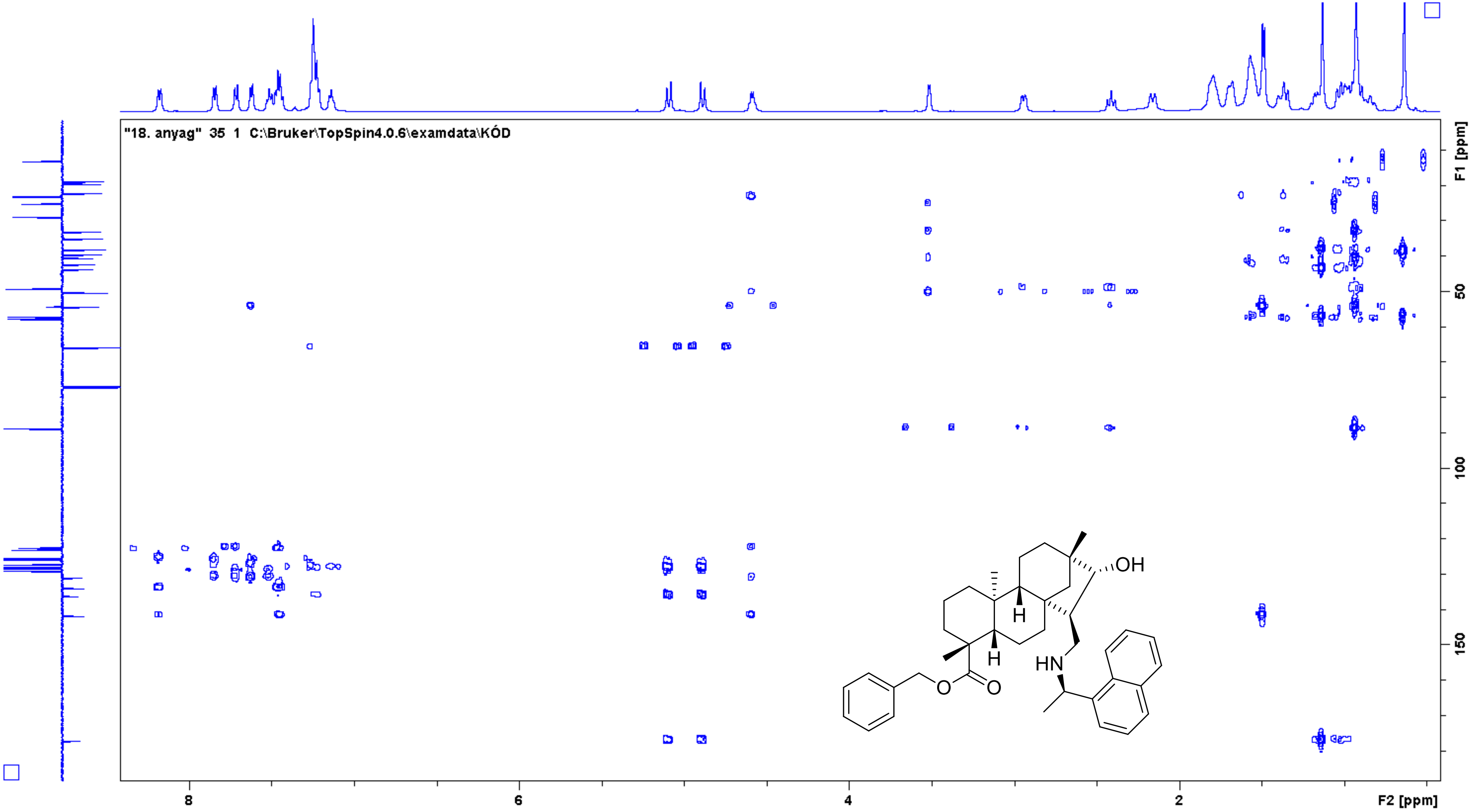

Figure S91

^{1}H-NMR of compound (4*R*,4a*S*,6a*S*,7*R*,8*R*,9*S*,11b*S*)-Benzyl 8-hydroxy-4,9,11b-trimethyl-7-((((*S*)-1-(naphthalen-1-yl)ethyl)amino)methyl)tetradecahydro-6a,9-methanocyclohepta[a]naphthalene-4-carboxylate **(19)**:

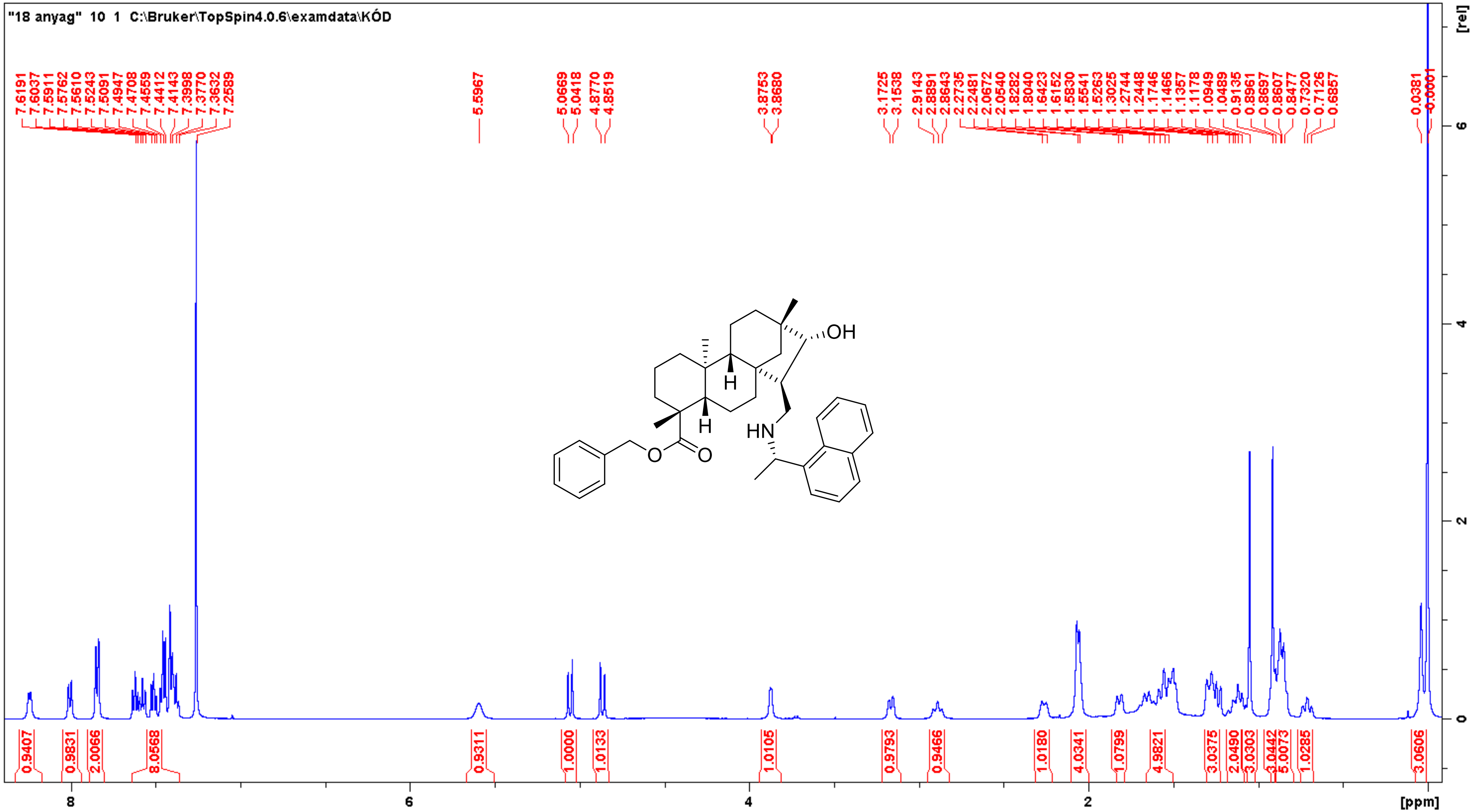

Figure S92

^{13}C-NMR of compound (4*R*,4a*S*,6a*S*,7*R*,8*R*,9*S*,11b*S*)-Benzyl 8-hydroxy-4,9,11b-trimethyl-7-((((*S*)-1-(naphthalen-1-yl)ethyl)amino)methyl)tetradecahydro-6a,9-methanocyclohepta[a]naphthalene-4-carboxylate **(19)**:

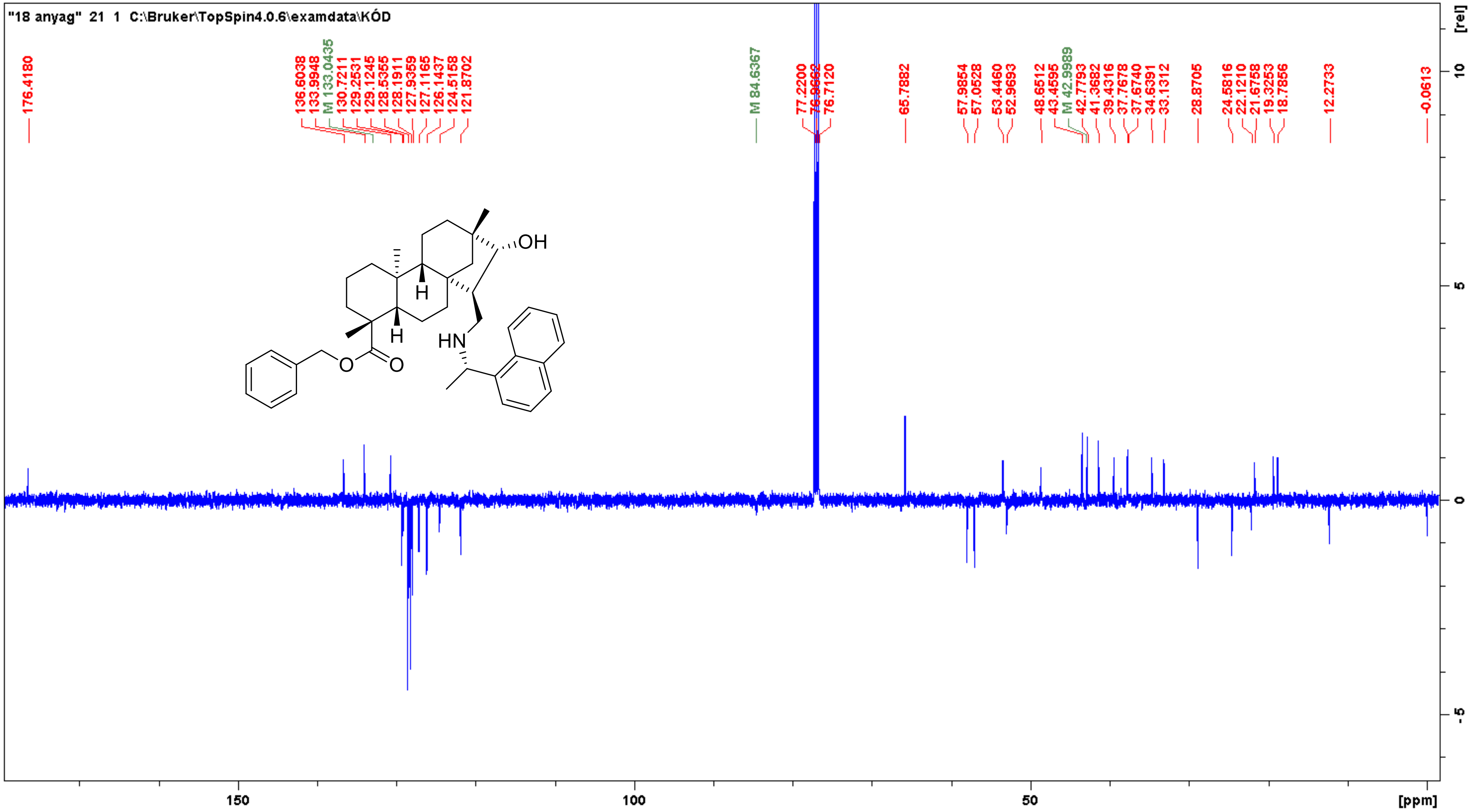

Figure S93

COSY of compound (4*R*,4a*S*,6a*S*,7*R*,8*R*,9*S*,11b*S*)-Benzyl 8-hydroxy-4,9,11b-trimethyl-7-((((*S*)-1-(naphthalen-1-yl)ethyl)amino)methyl)tetradecahydro-6a,9-methanocyclohepta[a]naphthalene-4-carboxylate **(19)**:

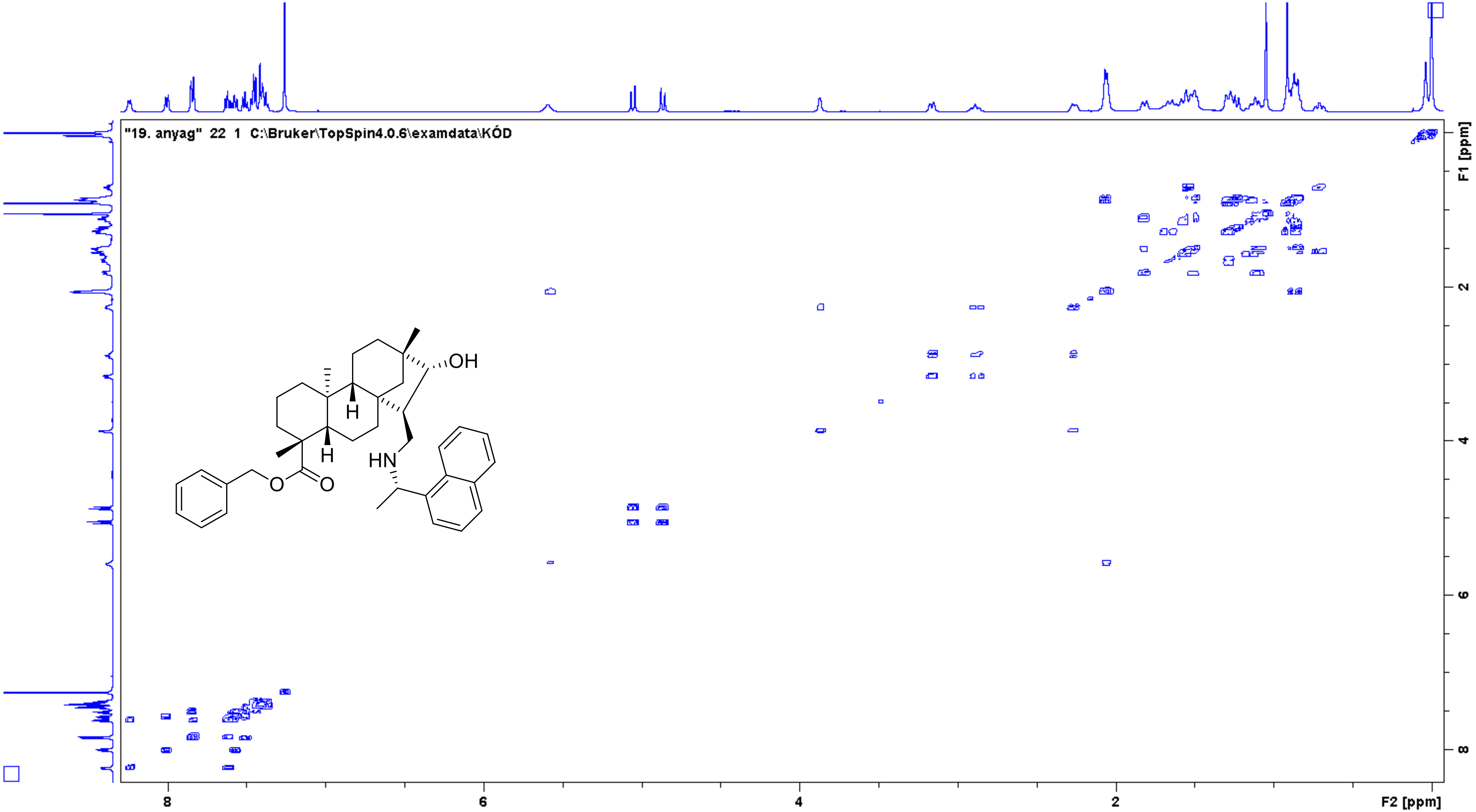

Figure S94

NOESY of compound (4*R*,4a*S*,6a*S*,7*R*,8*R*,9*S*,11b*S*)-Benzyl 8-hydroxy-4,9,11b-trimethyl-7-((((*S*)-1-(naphthalen-1-yl)ethyl)amino)methyl)tetradecahydro-6a,9-methanocyclohepta[a]naphthalene-4-carboxylate **(19)**:

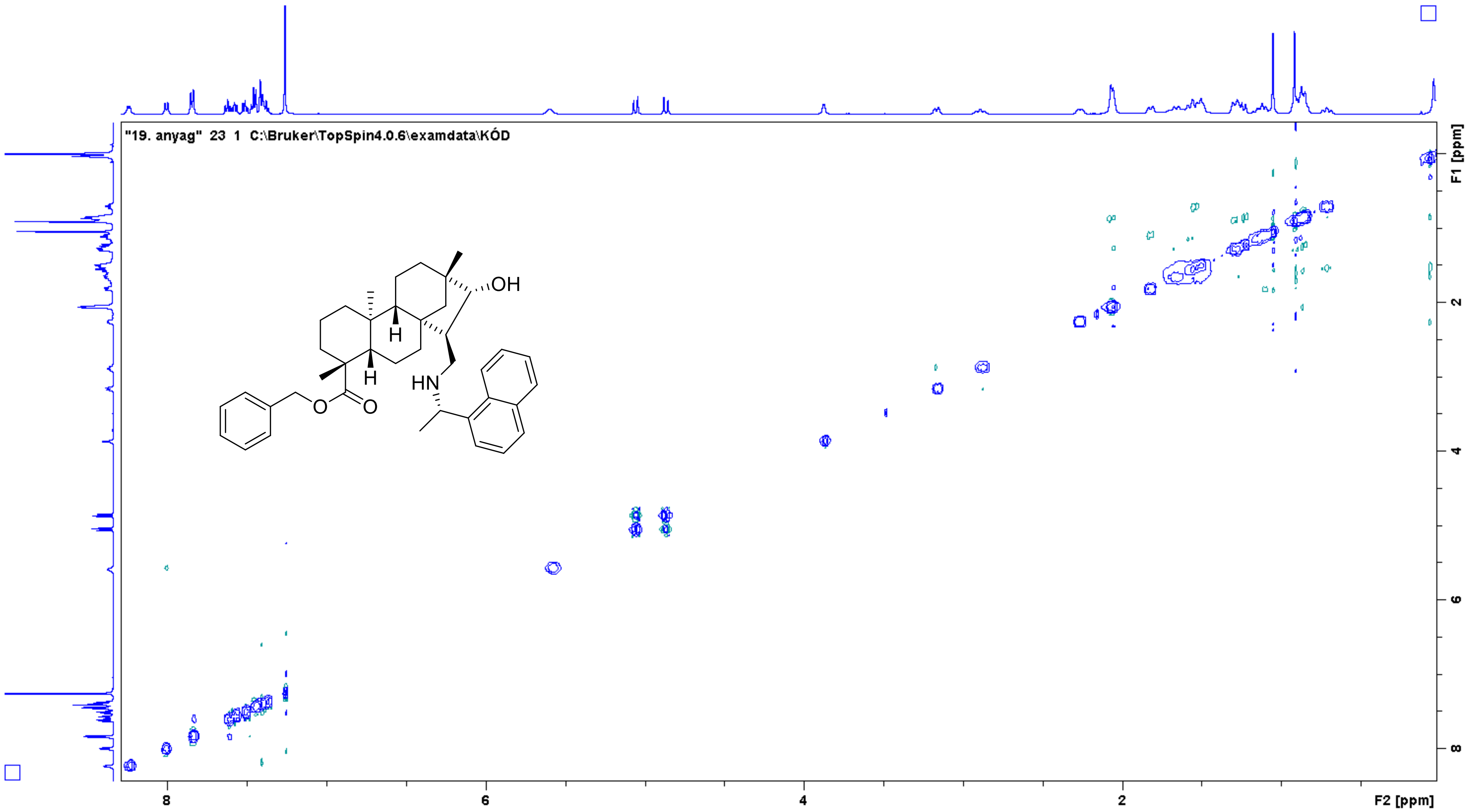

Figure S95

HSQC of compound (4*R*,4a*S*,6a*S*,7*R*,8*R*,9*S*,11b*S*)-Benzyl 8-hydroxy-4,9,11b-trimethyl-7-((((*S*)-1-(naphthalen-1-yl)ethyl)amino)methyl)tetradecahydro-6a,9-methanocyclohepta[a]naphthalene-4-carboxylate **(19)**:

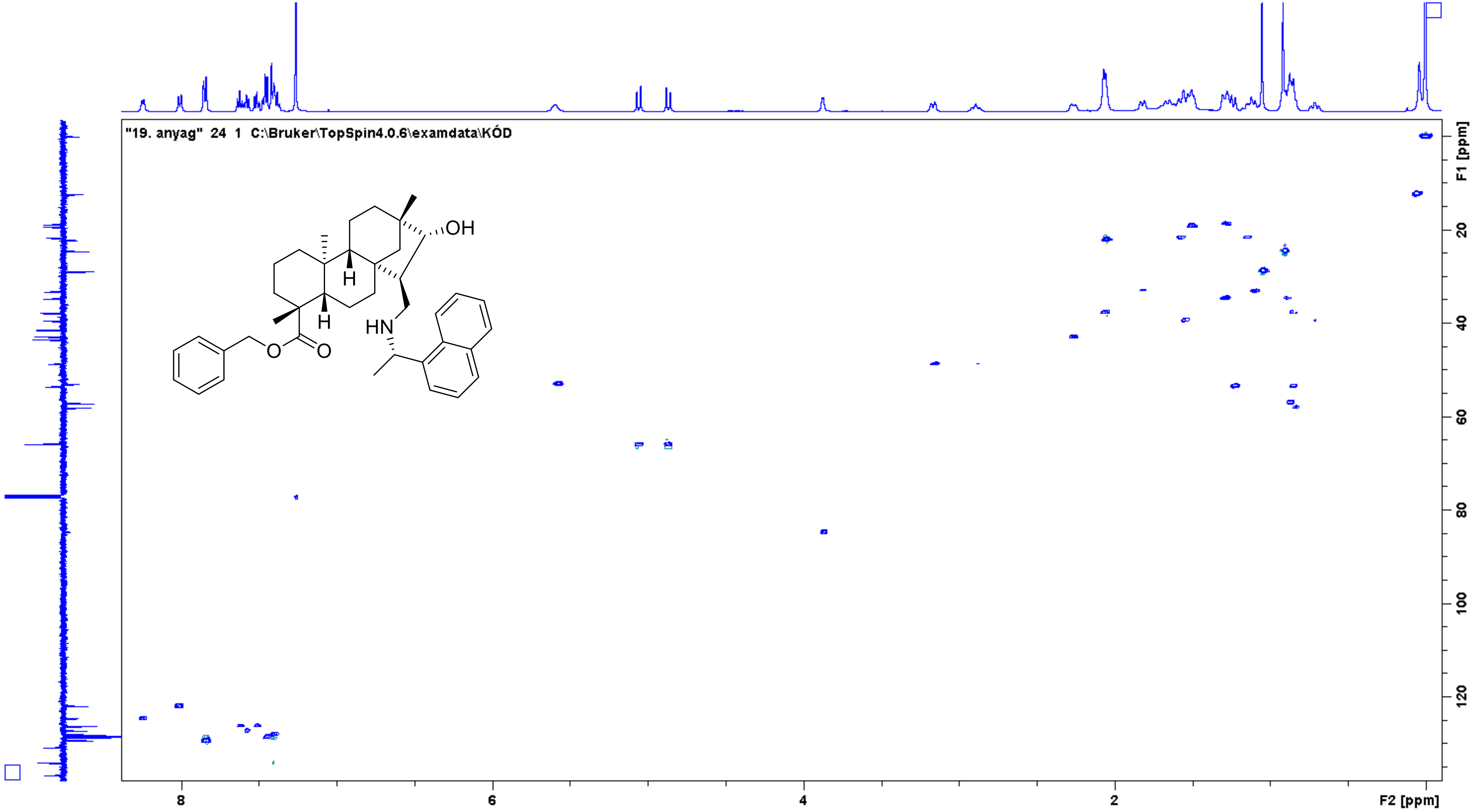

Figure S96

HMBC of compound (4*R*,4a*S*,6a*S*,7*R*,8*R*,9*S*,11b*S*)-Benzyl 8-hydroxy-4,9,11b-trimethyl-7-((((*S*)-1-(naphthalen-1-yl)ethyl)amino)methyl)tetradecahydro-6a,9-methanocyclohepta[a]naphthalene-4-carboxylate **(19)**:

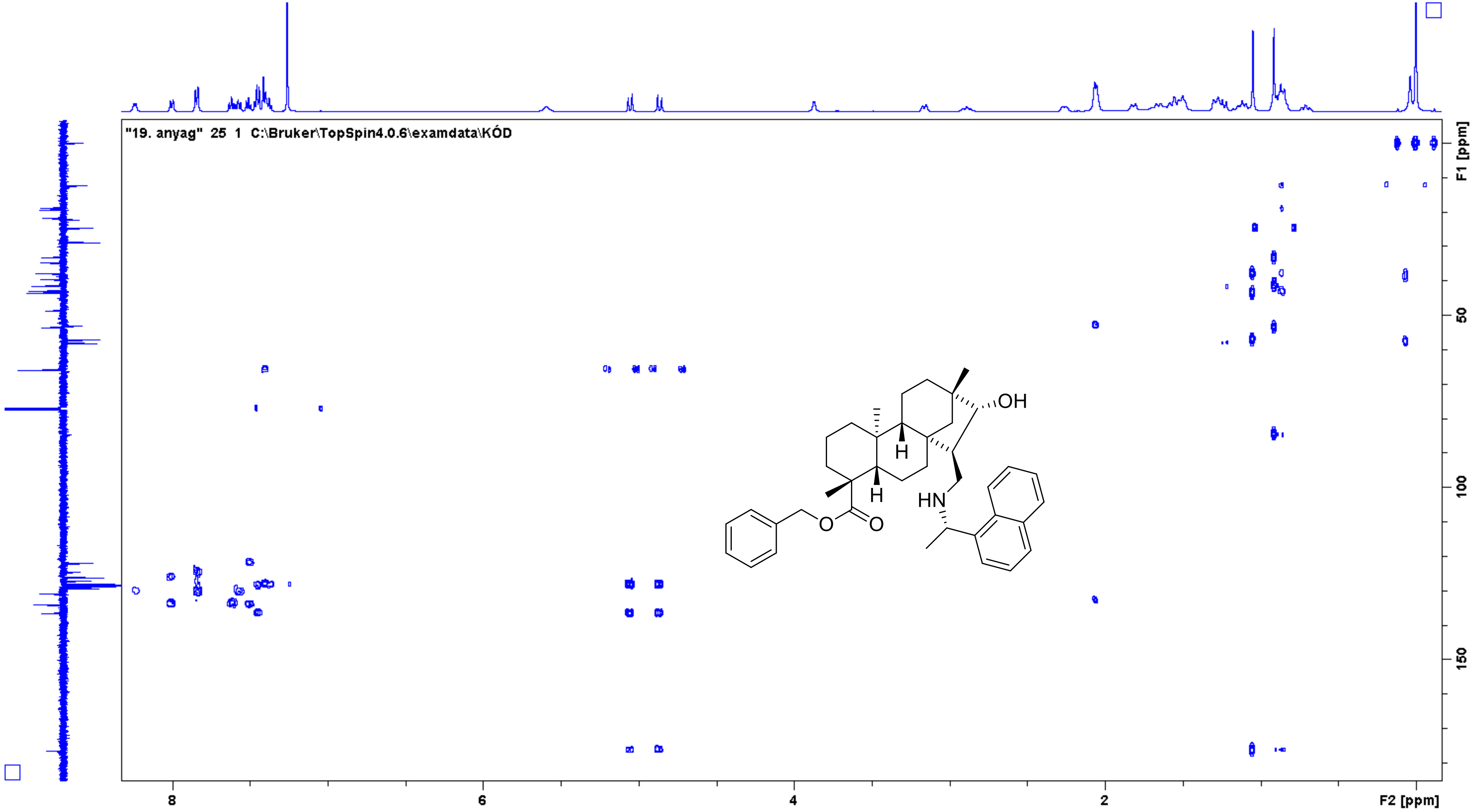

Figure S97

^{1}H-NMR of compound (4*R*,4a*S*,7*R*,8*R*,11b*S*)-Benzyl 7-(((3-(1H-imidazol-1-yl)propyl)amino)methyl)-8-hydroxy-4,9,11b-trimethyltetradecahydro-6a,9-methanocyclohepta[a]naphthalene-4-carboxylate **(20)**:

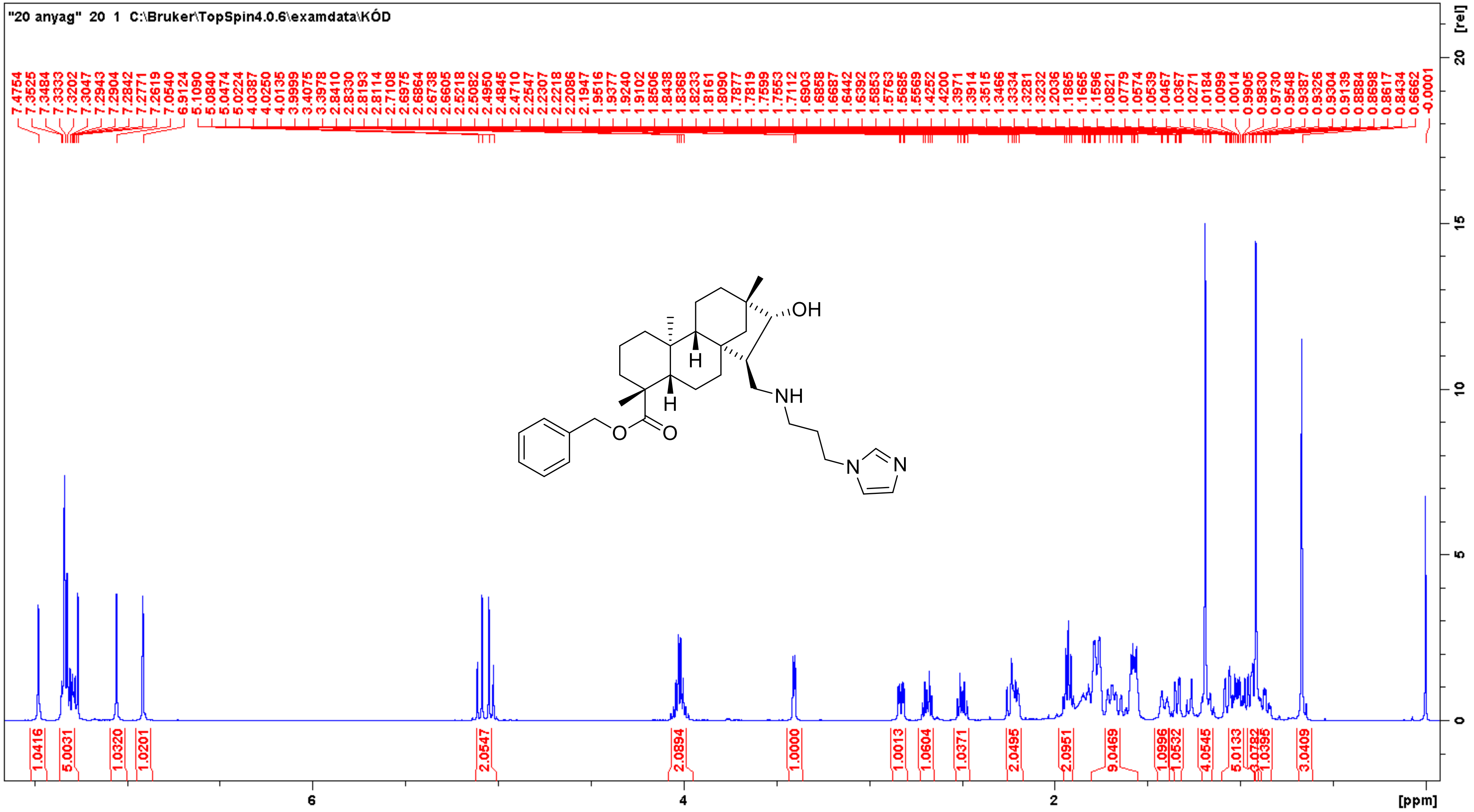

Figure S98

^{13}C-NMR of compound (4*R*,4a*S*,7*R*,8*R*,11b*S*)-Benzyl 7-(((3-(1H-imidazol-1-yl)propyl)amino)methyl)-8-hydroxy-4,9,11b-trimethyltetradecahydro-6a,9-methanocyclohepta[a]naphthalene-4-carboxylate **(20)**:

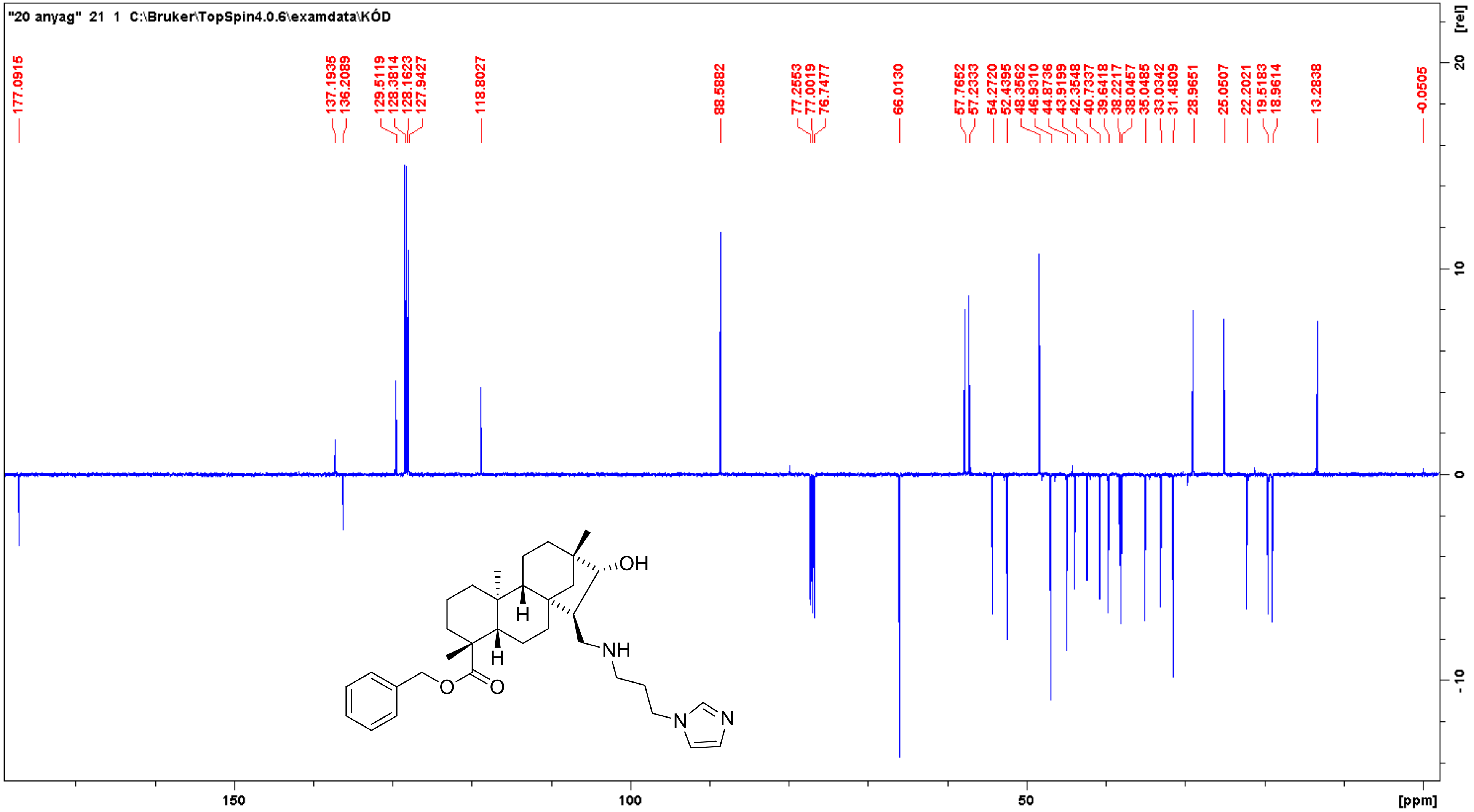

Figure S99

COSY of compound (4*R*,4a*S*,7*R*,8*R*,11b*S*)-Benzyl 7-(((3-(1H-imidazol-1-yl)propyl)amino)methyl)-8-hydroxy-4,9,11b-trimethyltetradecahydro-6a,9-methanocyclohepta[a]naphthalene-4-carboxylate **(20)**:

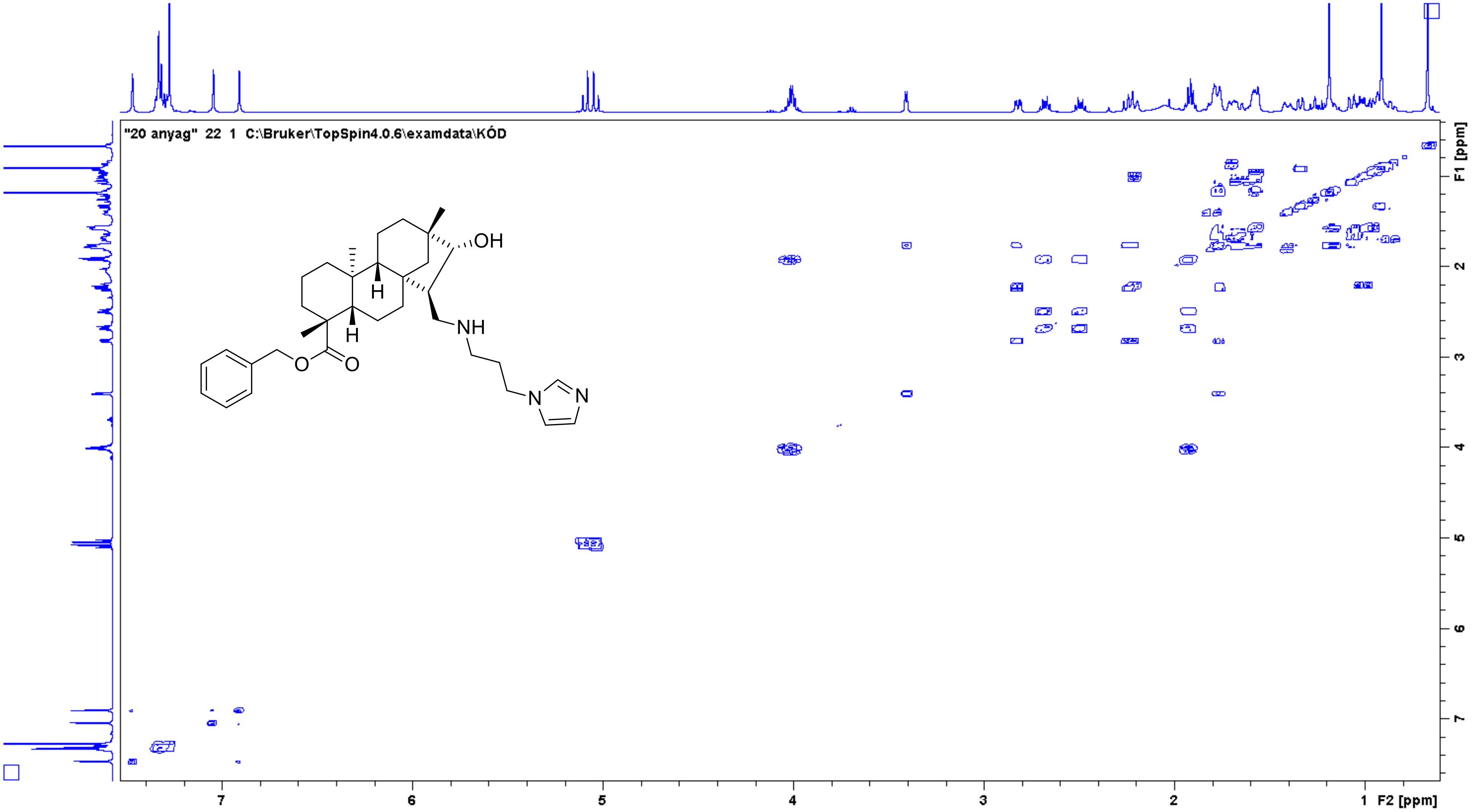

Figure S100

NOESY of compound (4*R*,4a*S*,7*R*,8*R*,11b*S*)-Benzyl 7-(((3-(1H-imidazol-1-yl)propyl)amino)methyl)-8-hydroxy-4,9,11b-trimethyltetradecahydro-6a,9-methanocyclohepta[a]naphthalene-4-carboxylate **(20)**:

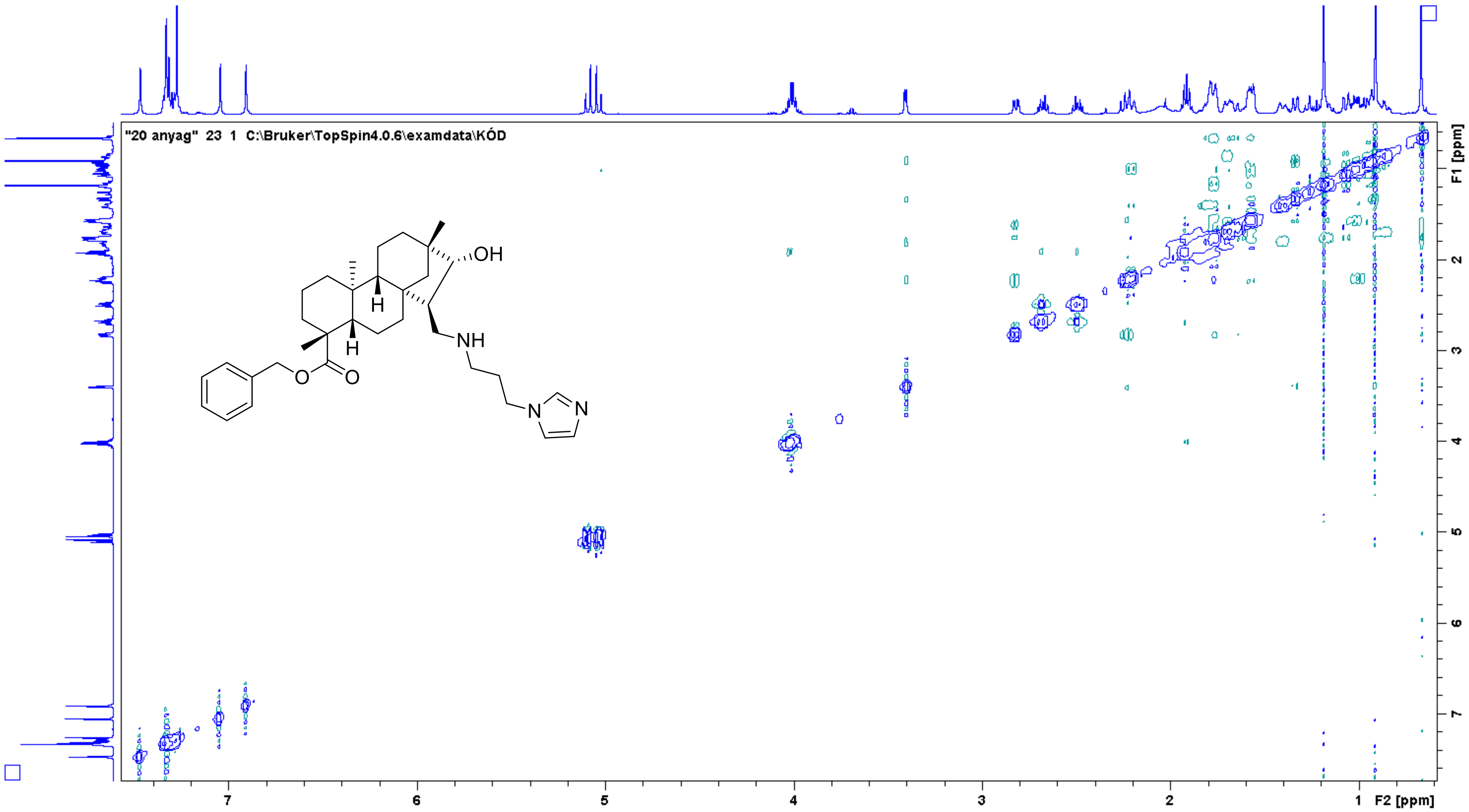

Figure S101

HSQC of compound (4*R*,4a*S*,7*R*,8*R*,11b*S*)-Benzyl 7-(((3-(1H-imidazol-1-yl)propyl)amino)methyl)-8-hydroxy-4,9,11b-trimethyltetradecahydro-6a,9-methanocyclohepta[a]naphthalene-4-carboxylate **(20)**:

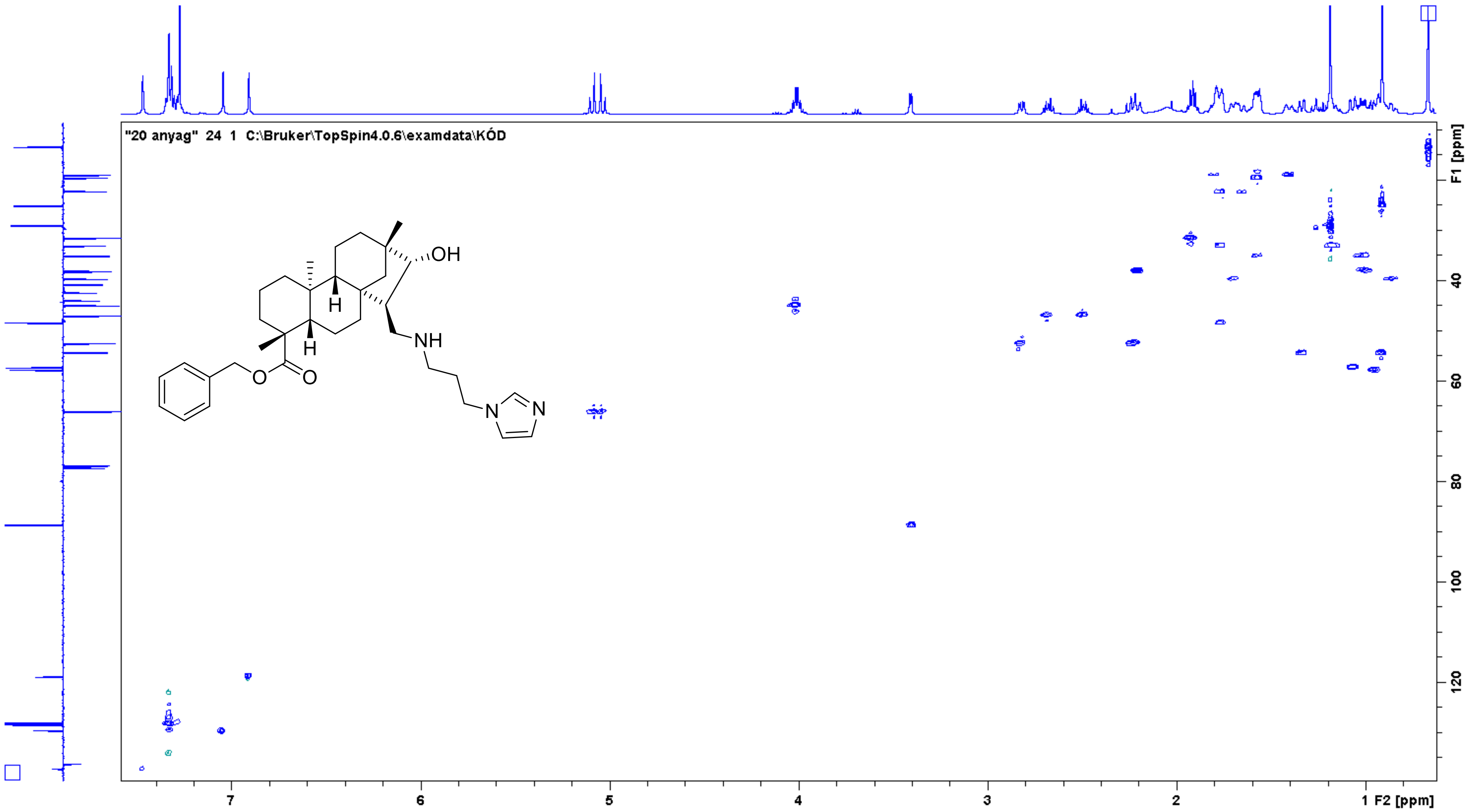

Figure S102

HMBC of compound (4*R*,4a*S*,7*R*,8*R*,11b*S*)-Benzyl 7-(((3-(1H-imidazol-1-yl)propyl)amino)methyl)-8-hydroxy-4,9,11b-trimethyltetradecahydro-6a,9-methanocyclohepta[a]naphthalene-4-carboxylate **(20)**:

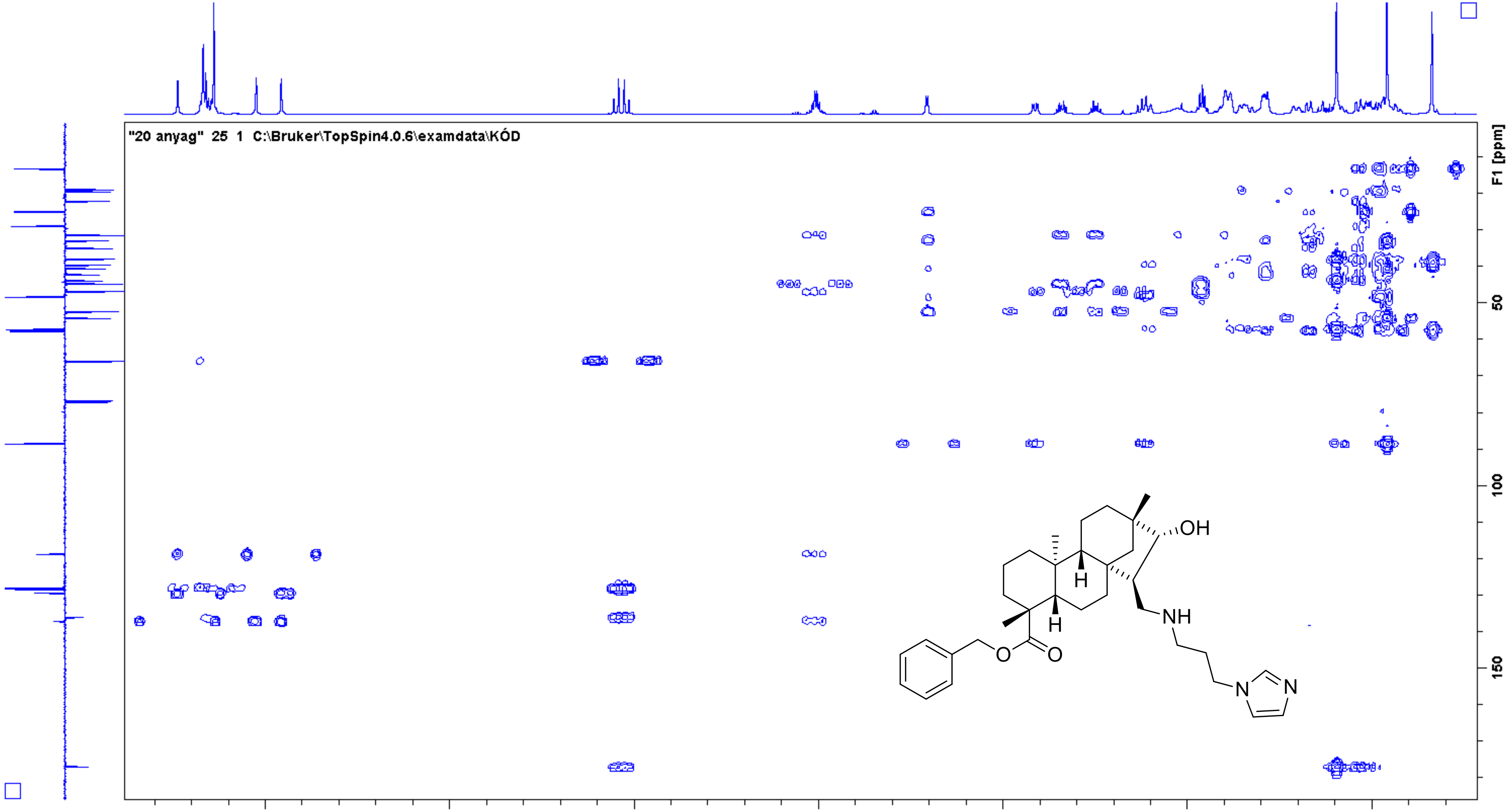

Figure S103

^{1}H-NMR of compound (4*R*,4a*S*,6a*S*,7*R*,8*R*,9*S*,11b*S*)-Methyl 7-((((*R*)-1-(4-fluorophenyl)ethyl)amino)methyl)-8-hydroxy-4,9,11b-trimethyltetradecahydro-6a,9-methanocyclohepta[a]naphthalene-4-carboxylate **(23)**:

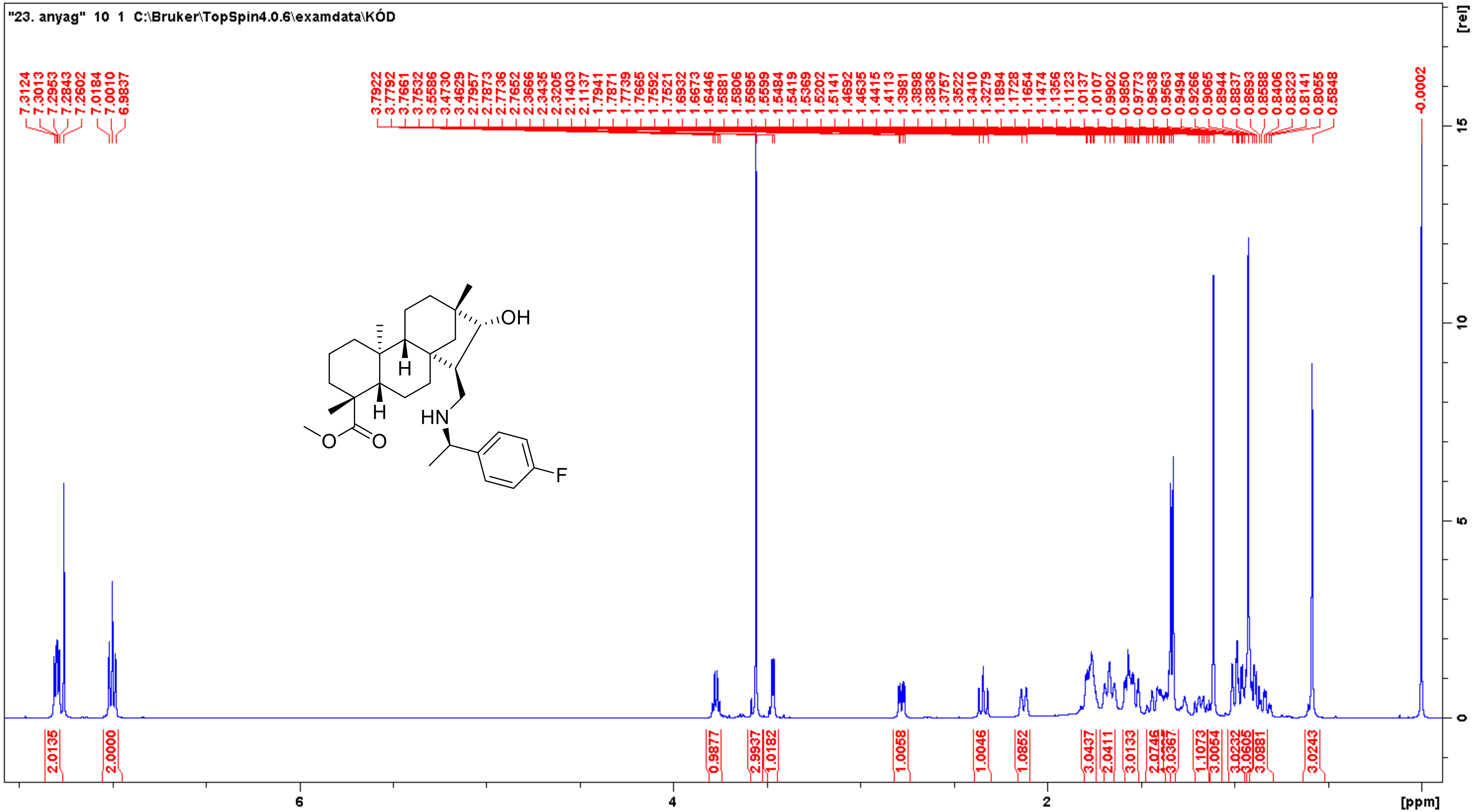

Figure S104

^{13}C-NMR of compound (4*R*,4a*S*,6a*S*,7*R*,8*R*,9*S*,11b*S*)-Methyl 7-((((*R*)-1-(4-fluorophenyl)ethyl)amino)methyl)-8-hydroxy-4,9,11b-trimethyltetradecahydro-6a,9-methanocyclohepta[a]naphthalene-4-carboxylate **(23)**:

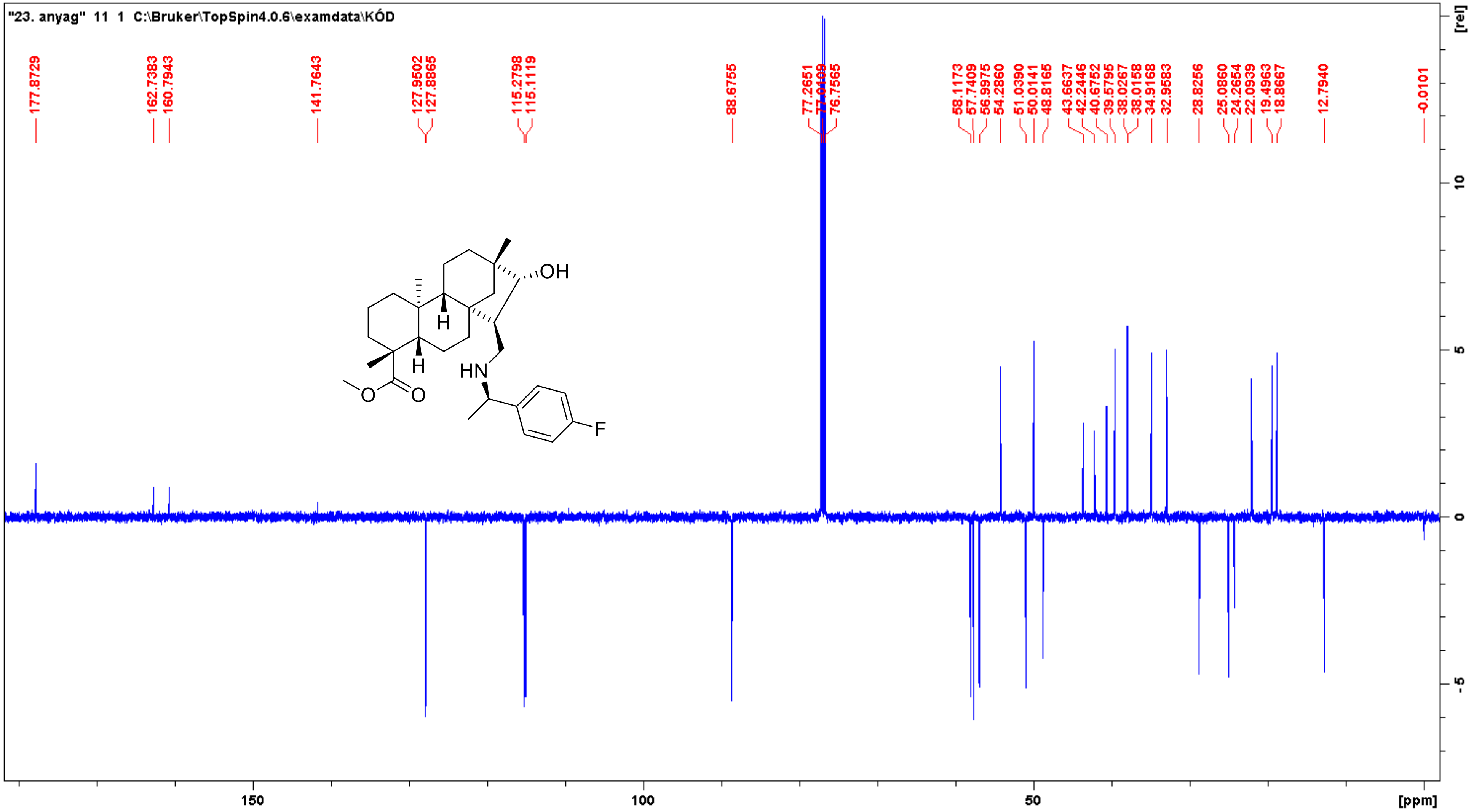

Figure S105

COSY of compound (4*R*,4a*S*,6a*S*,7*R*,8*R*,9*S*,11b*S*)-Methyl 7-((((*R*)-1-(4-fluorophenyl)ethyl)amino)methyl)-8-hydroxy-4,9,11b-trimethyltetradecahydro-6a,9-methanocyclohepta[a]naphthalene-4-carboxylate **(23)**:

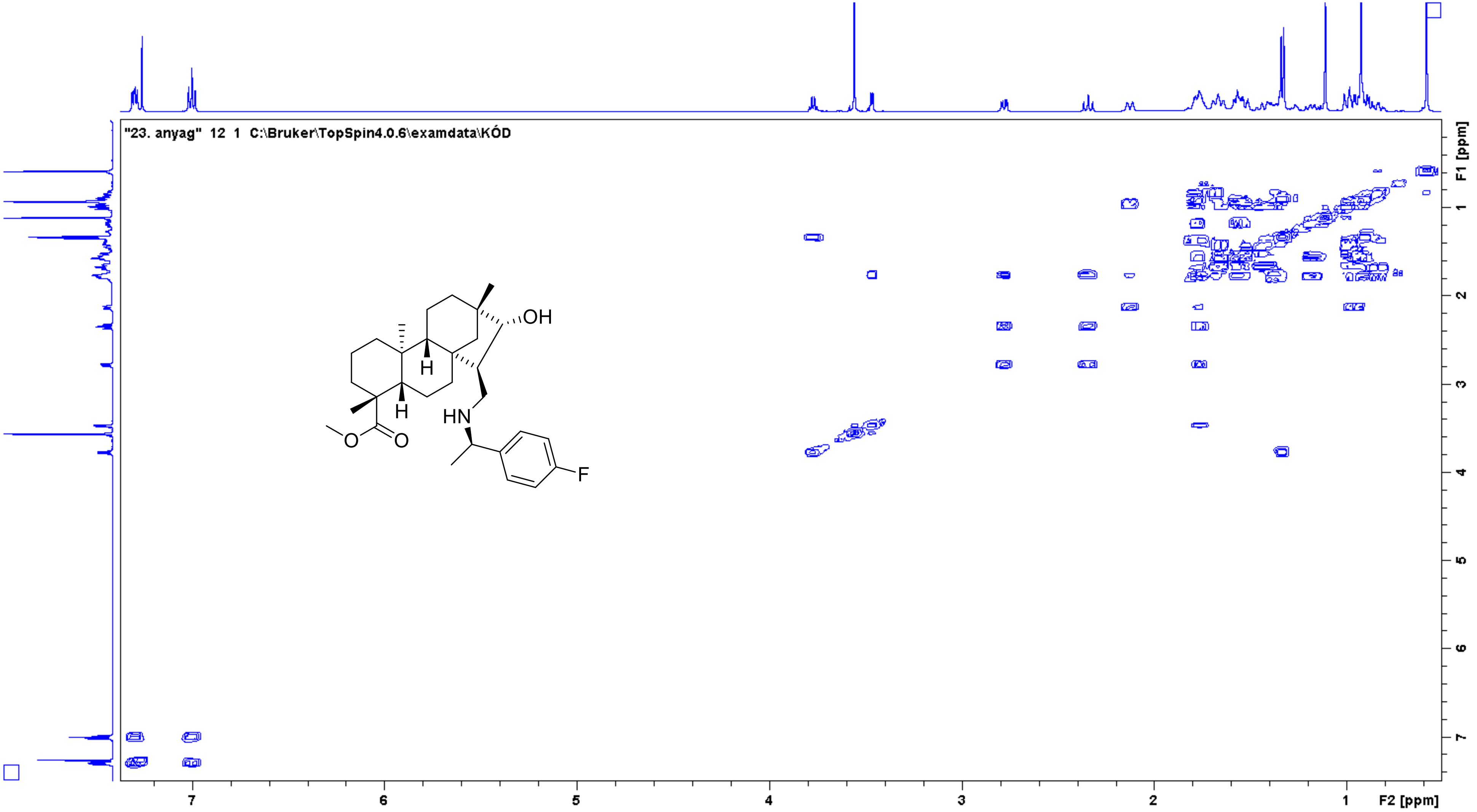

Figure S106

NOESY of compound (4*R*,4a*S*,6a*S*,7*R*,8*R*,9*S*,11b*S*)-Methyl 7-((((*R*)-1-(4-fluorophenyl)ethyl)amino)methyl)-8-hydroxy-4,9,11b-trimethyltetradecahydro-6a,9-methanocyclohepta[a]naphthalene-4-carboxylate **(23)**:

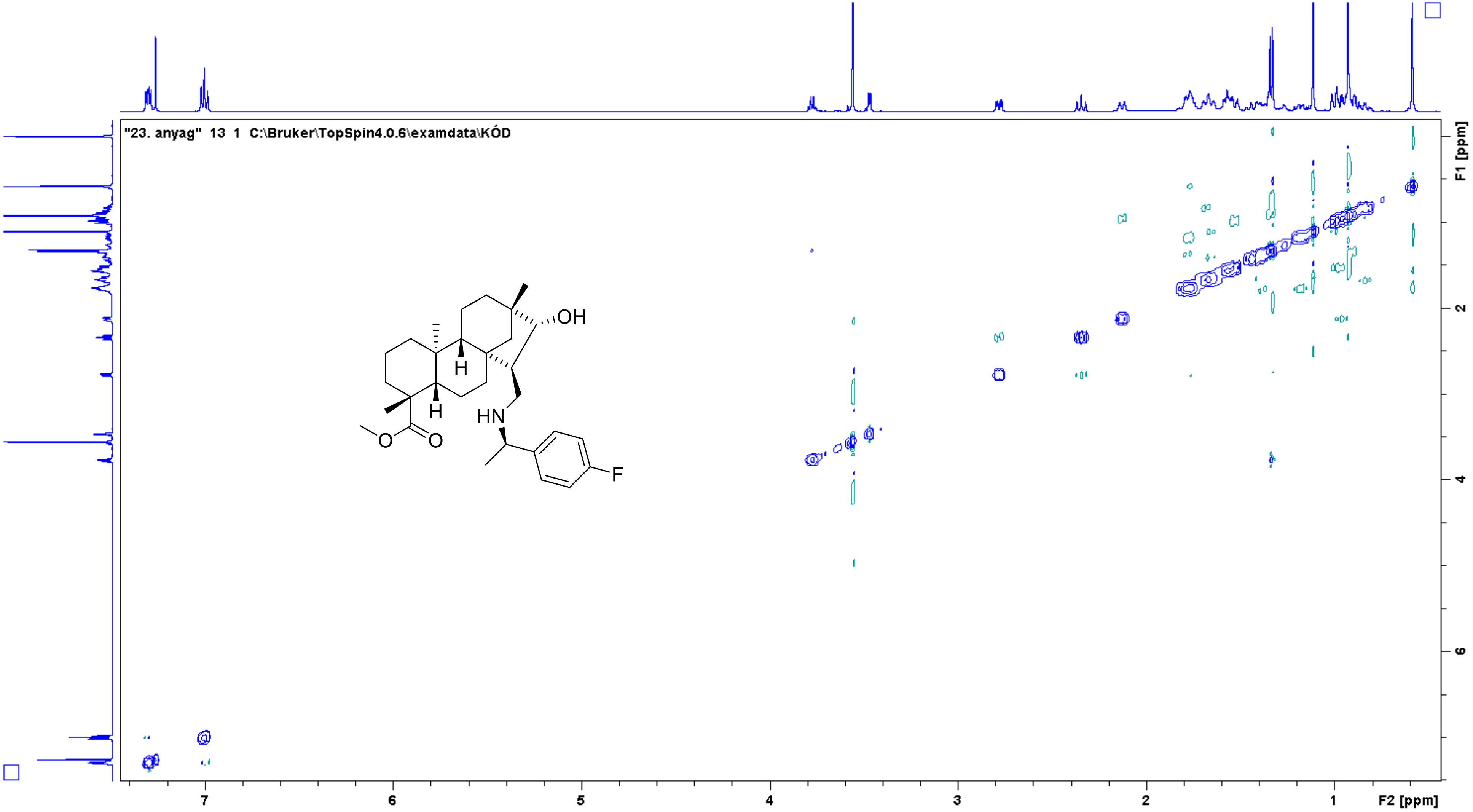

Figure S107

HSQC of compound (4*R*,4a*S*,6a*S*,7*R*,8*R*,9*S*,11b*S*)-Methyl 7-((((*R*)-1-(4-fluorophenyl)ethyl)amino)methyl)-8-hydroxy-4,9,11b-trimethyltetradecahydro-6a,9-methanocyclohepta[a]naphthalene-4-carboxylate **(23)**:

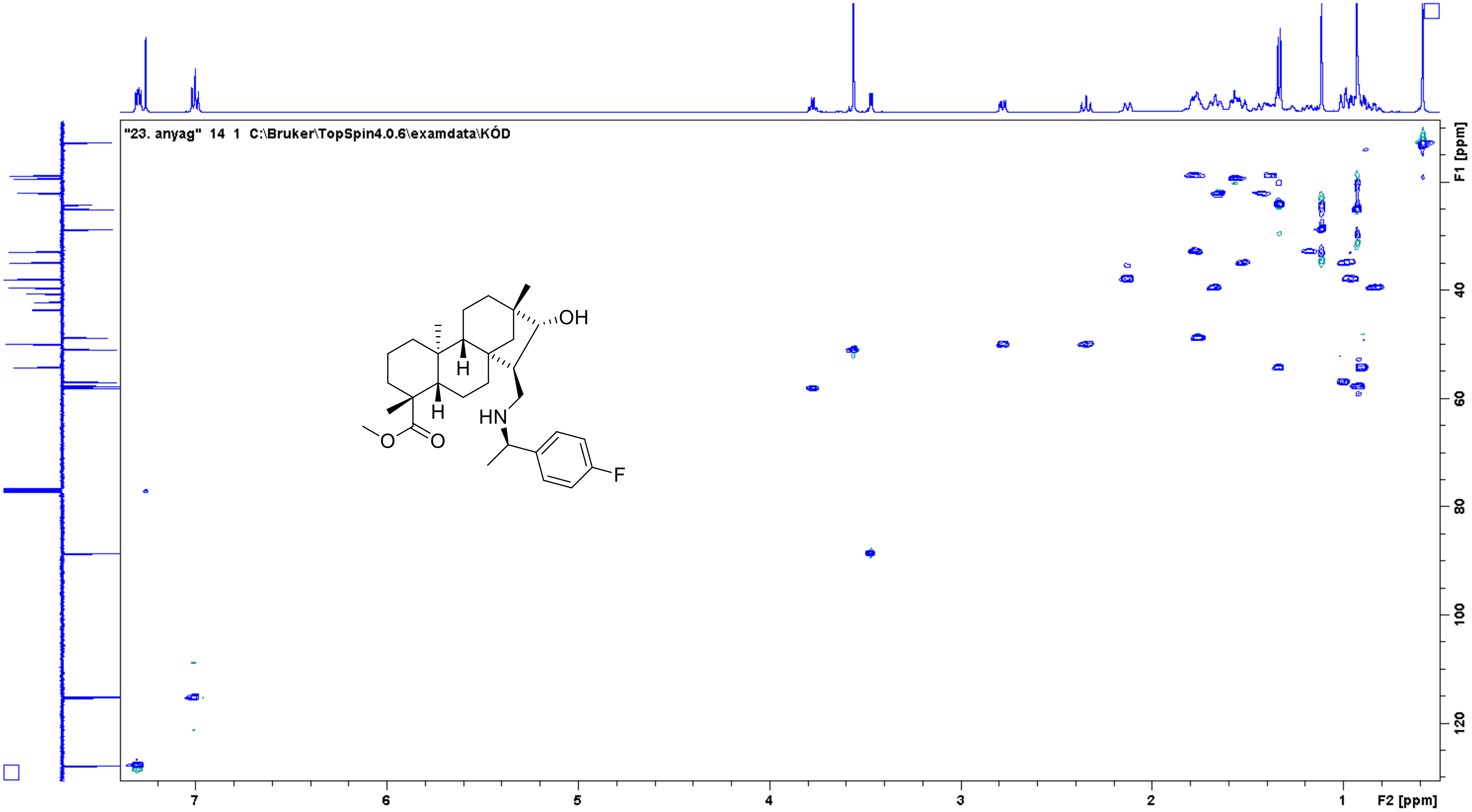

Figure S108

HMBC of compound (4*R*,4a*S*,6a*S*,7*R*,8*R*,9*S*,11b*S*)-Methyl 7-((((*R*)-1-(4-fluorophenyl)ethyl)amino)methyl)-8-hydroxy-4,9,11b-trimethyltetradecahydro-6a,9-methanocyclohepta[a]naphthalene-4-carboxylate **(23)**:

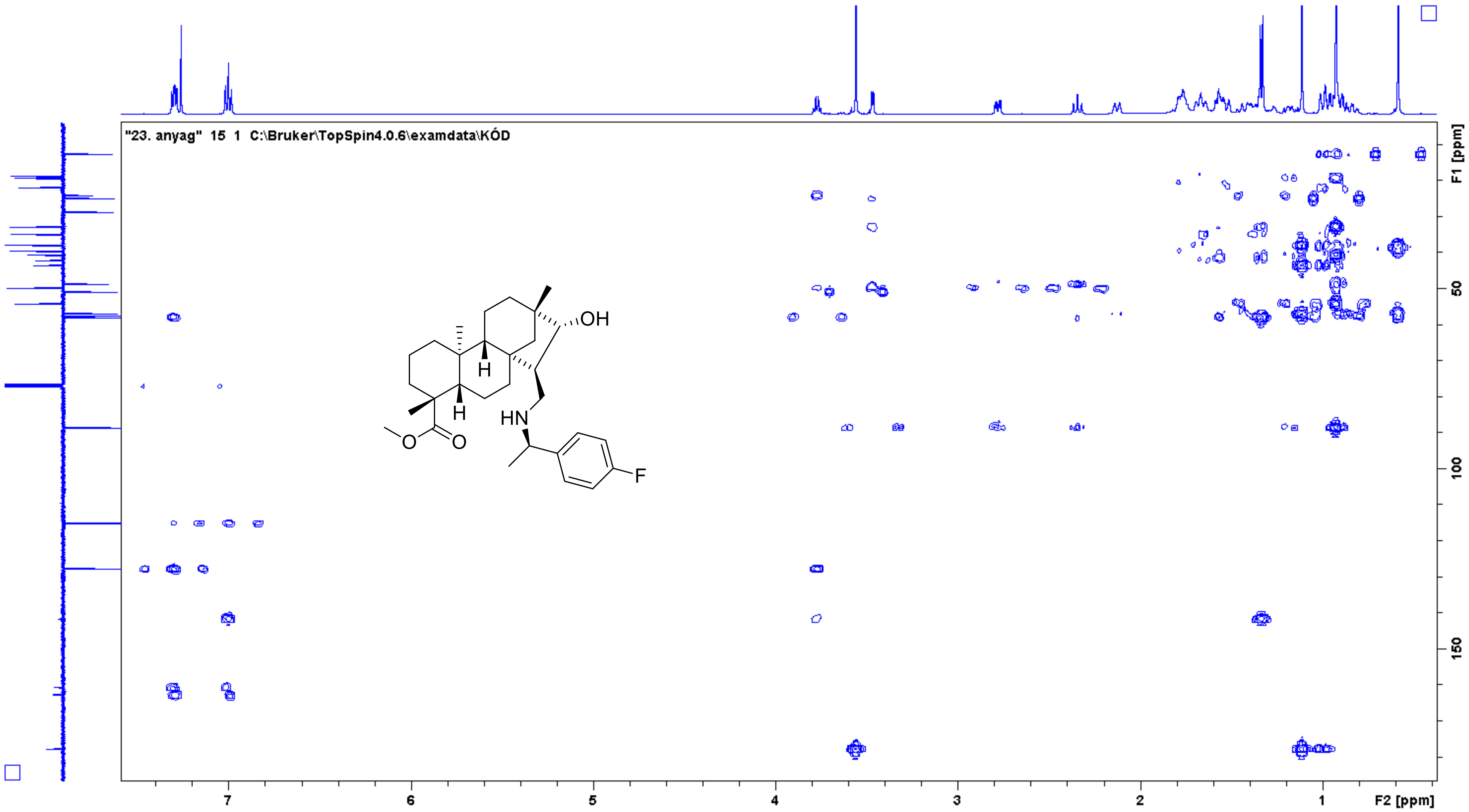

Figure S109

^{19}F-NMR of compound (4*R*,4a*S*,6a*S*,7*R*,8*R*,9*S*,11b*S*)-Methyl 7-((((*R*)-1-(4-fluorophenyl)ethyl)amino)methyl)-8-hydroxy-4,9,11b-trimethyltetradecahydro-6a,9-methanocyclohepta[a]naphthalene-4-carboxylate **(23)**:

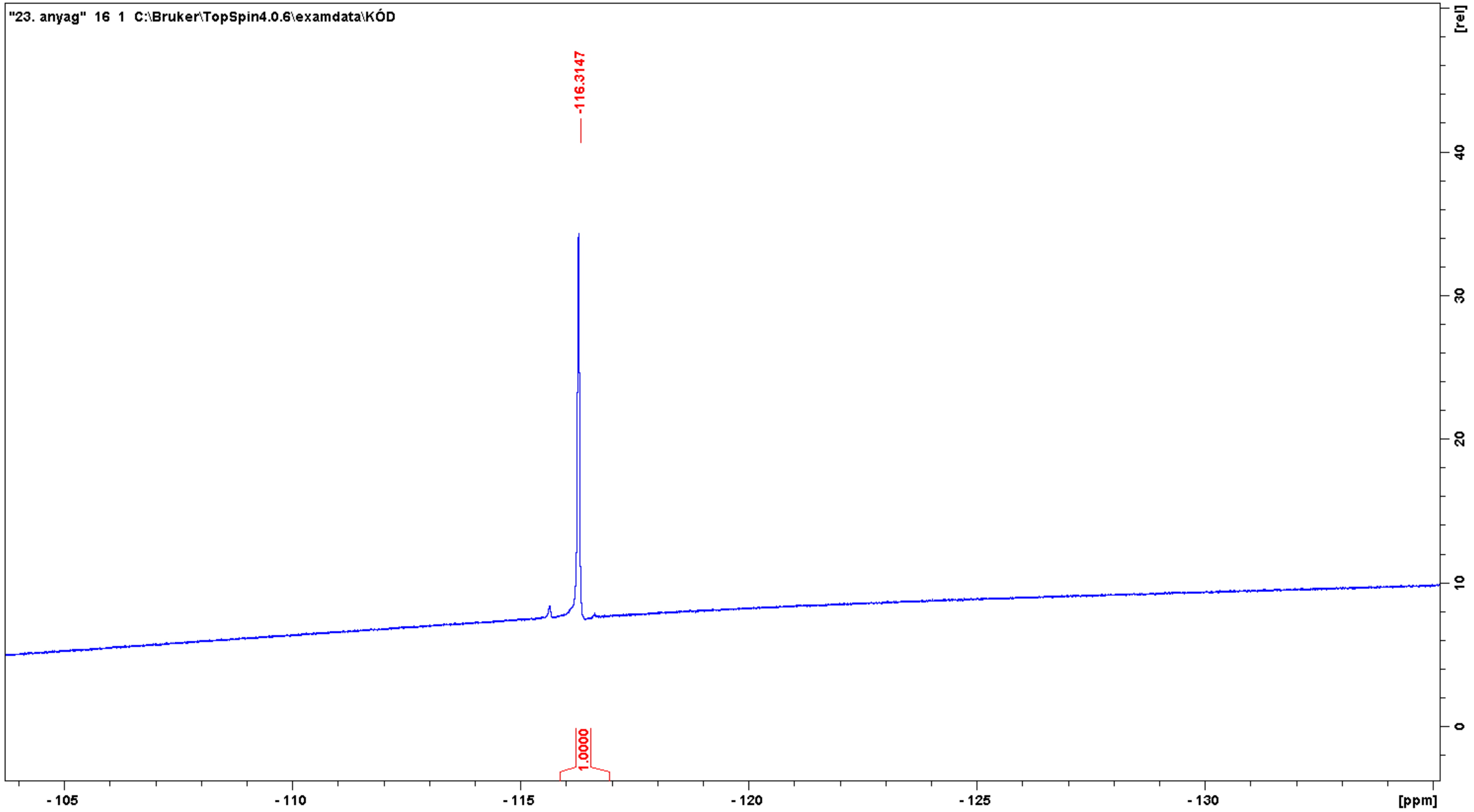

Figure S110

^{1}H-NMR of compound (4*R*,4a*S*,6a*S*,7*R*,8*R*,9*S*,11b*S*)-Methyl 8-hydroxy-4,9,11b-trimethyl-7-((((*R*)-1-phenylpropyl)amino)methyl)tetradecahydro-6a,9-methanocyclohepta[a]naphthalene-4-carboxylate **(24)**:

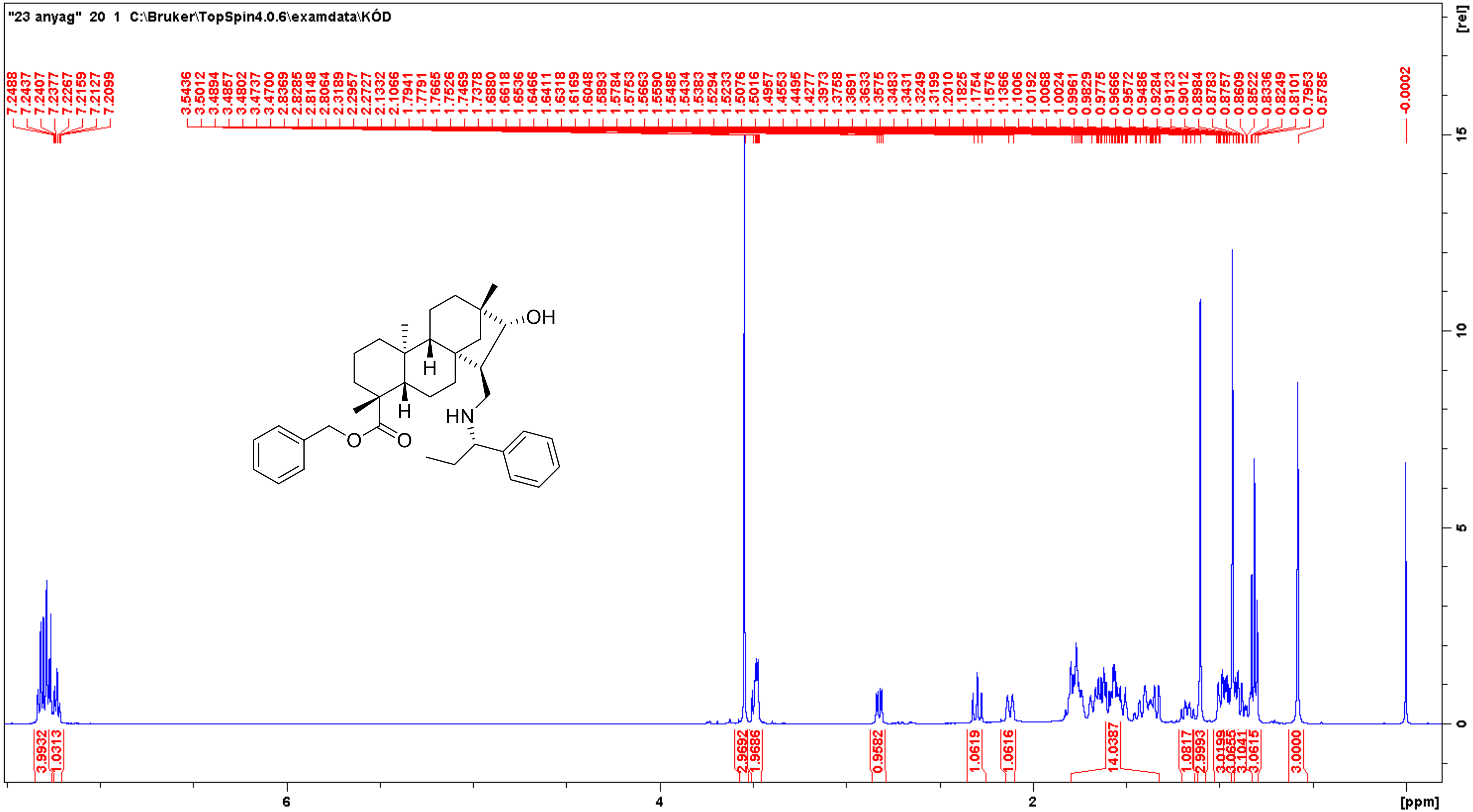

Figure S111

^{13}C-NMR of compound (4*R*,4a*S*,6a*S*,7*R*,8*R*,9*S*,11b*S*)-Methyl 8-hydroxy-4,9,11b-trimethyl-7-((((*R*)-1-phenylpropyl)amino)methyl)tetradecahydro-6a,9-methanocyclohepta[a]naphthalene-4-carboxylate **(24)**:

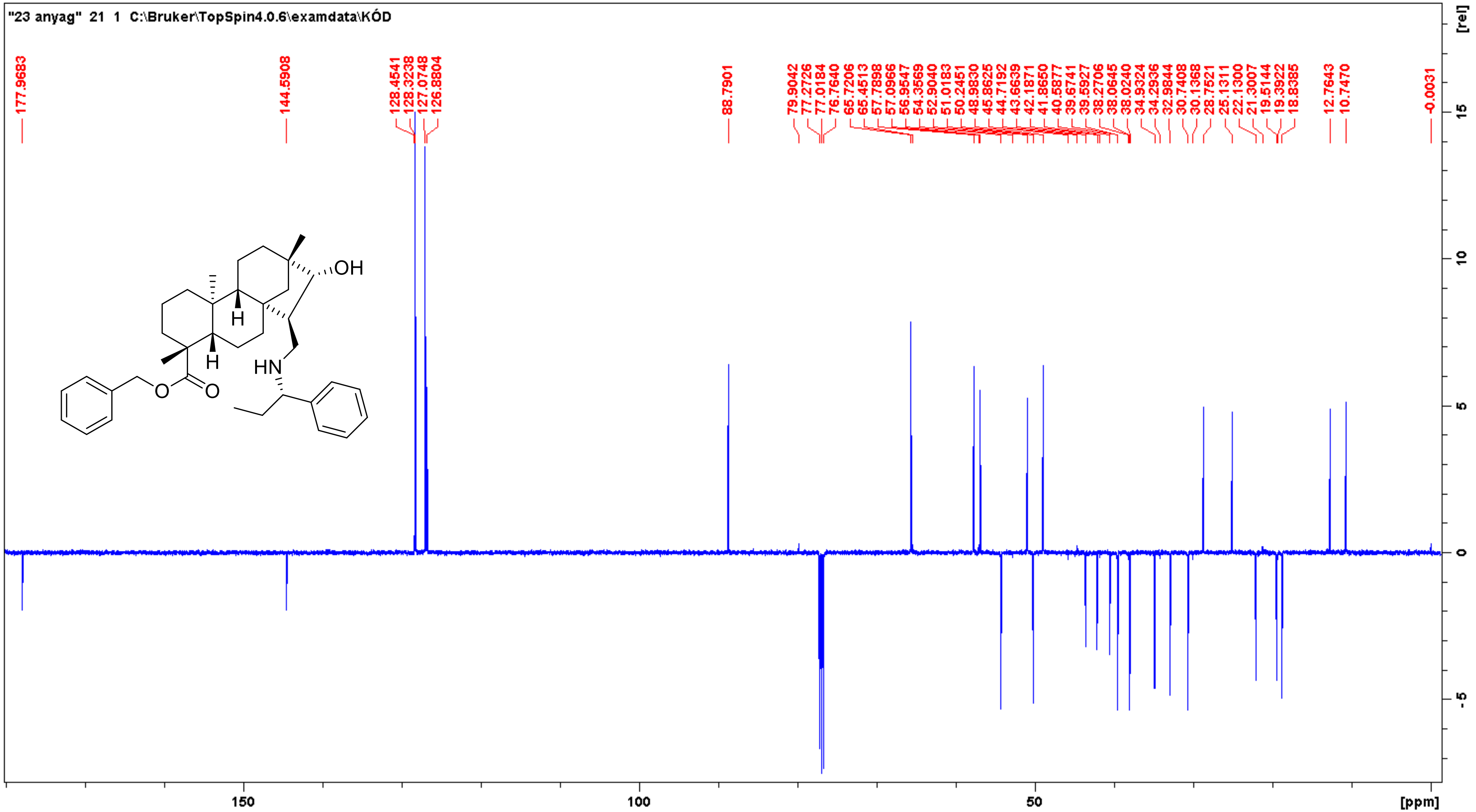

Figure S112

COSY of compound (4*R*,4a*S*,6a*S*,7*R*,8*R*,9*S*,11b*S*)-Methyl 8-hydroxy-4,9,11b-trimethyl-7-((((*R*)-1-phenylpropyl)amino)methyl)tetradecahydro-6a,9-methanocyclohepta[a]naphthalene-4-carboxylate **(24)**:

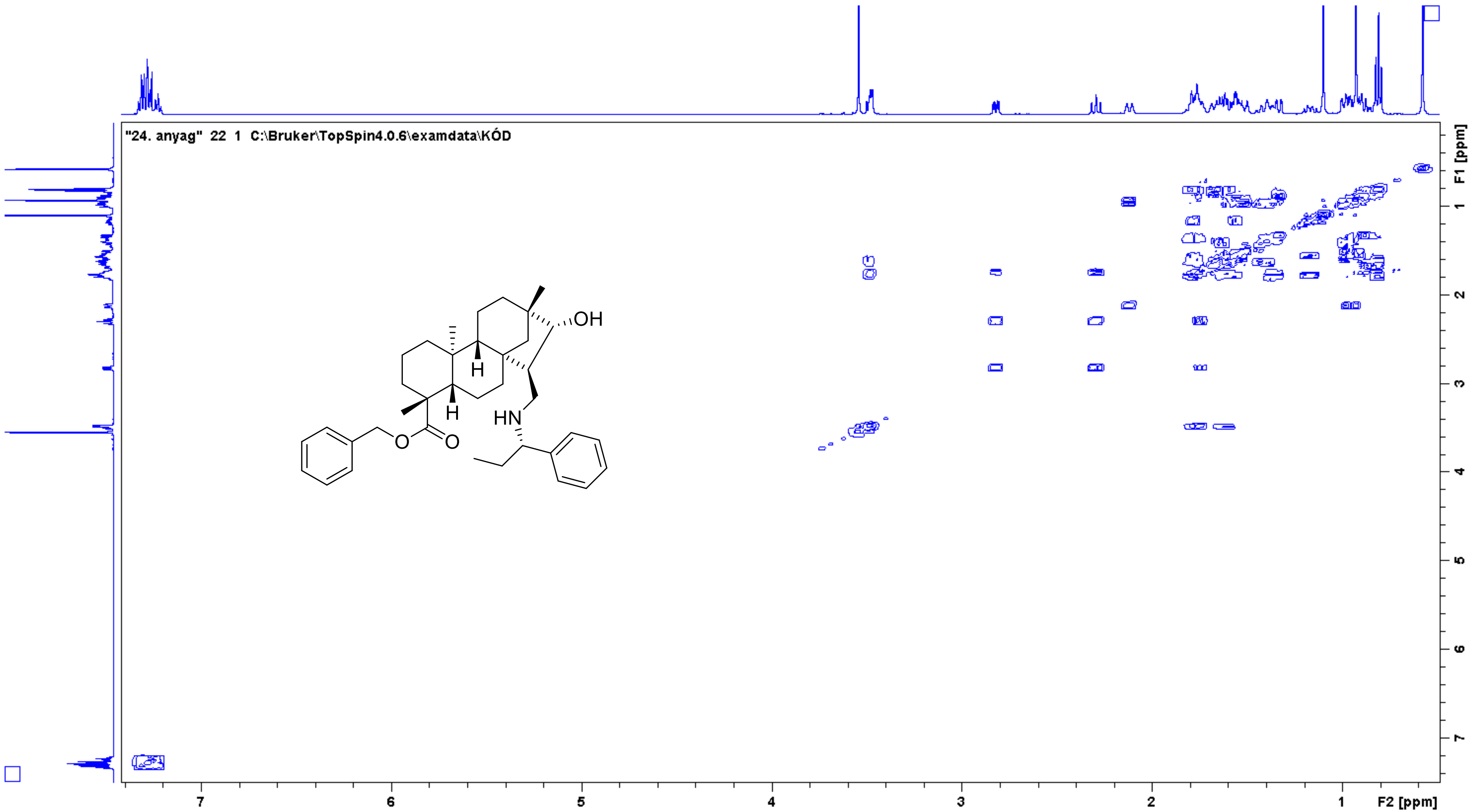

Figure S113

NOESY of compound (4*R*,4a*S*,6a*S*,7*R*,8*R*,9*S*,11b*S*)-Methyl 8-hydroxy-4,9,11b-trimethyl-7-((((*R*)-1-phenylpropyl)amino)methyl)tetradecahydro-6a,9-methanocyclohepta[a]naphthalene-4-carboxylate **(24)**:

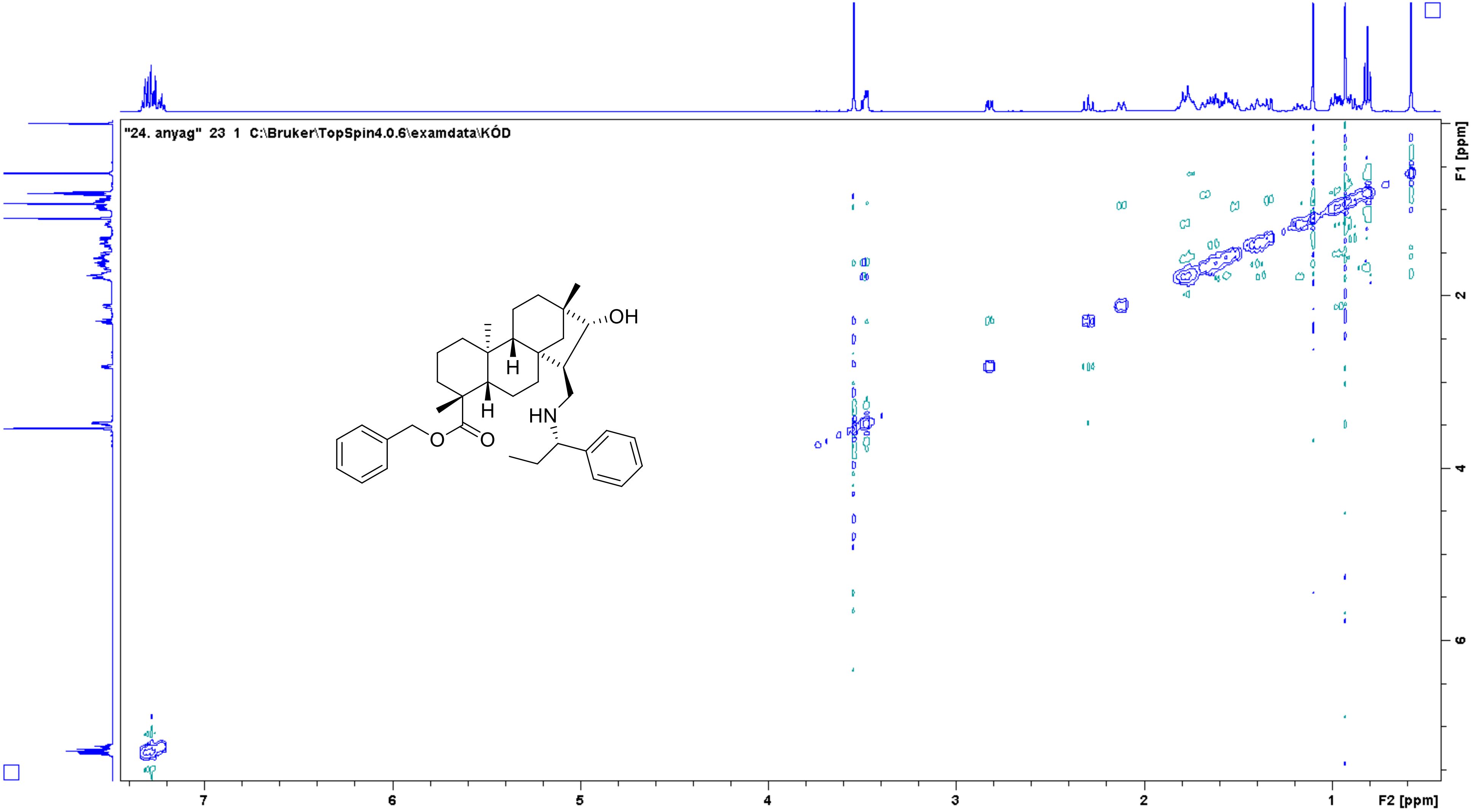

Figure S114

HSQC of compound (4*R*,4a*S*,6a*S*,7*R*,8*R*,9*S*,11b*S*)-Methyl 8-hydroxy-4,9,11b-trimethyl-7-((((*R*)-1-phenylpropyl)amino)methyl)tetradecahydro-6a,9-methanocyclohepta[a]naphthalene-4-carboxylate **(24)**:

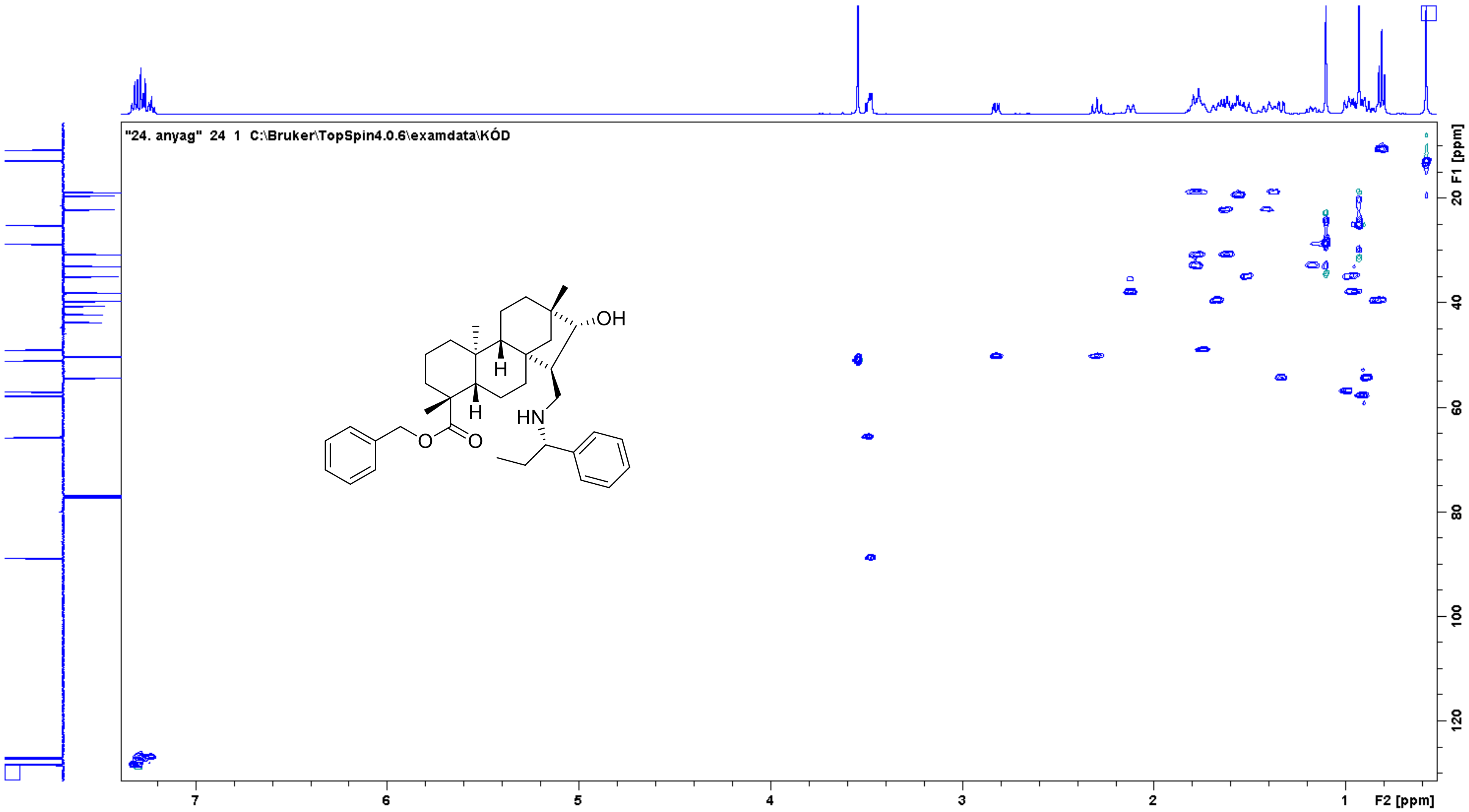

Figure S115

HMBC of compound (4*R*,4a*S*,6a*S*,7*R*,8*R*,9*S*,11b*S*)-Methyl 8-hydroxy-4,9,11b-trimethyl-7-((((*R*)-1-phenylpropyl)amino)methyl)tetradecahydro-6a,9-methanocyclohepta[a]naphthalene-4-carboxylate **(24)**:

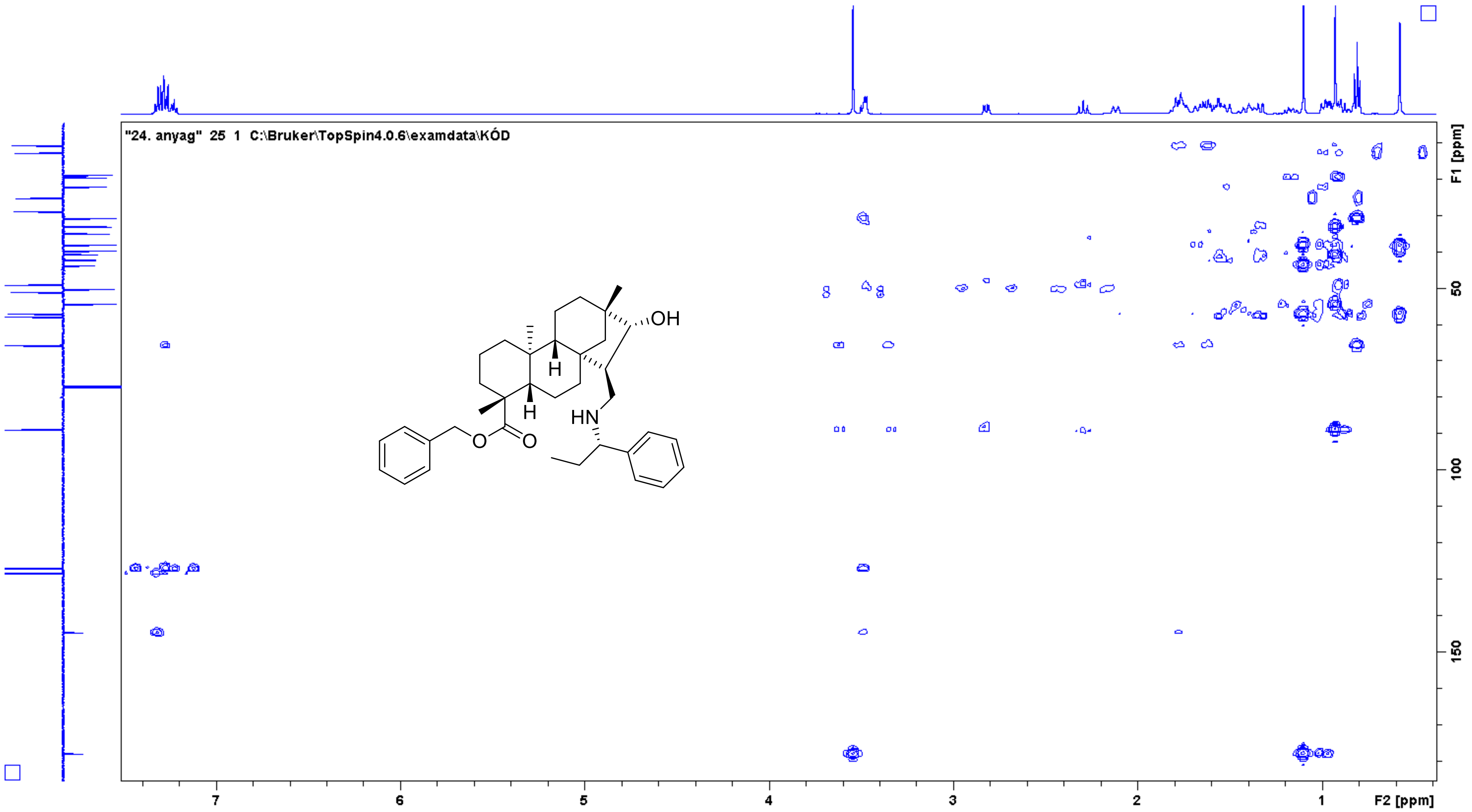

Figure S116

^{1}H-NMR of compound (4*R*,4a*S*,6a*S*,7*R*,8*R*,9*S*,11b*S*)-Methyl 8-hydroxy-4,9,11b-trimethyl-7-((((*S*)-1-phenylpropyl)amino)methyl)tetradecahydro-6a,9-methanocyclohepta[a]naphthalene-4-carboxylate **(25)**:

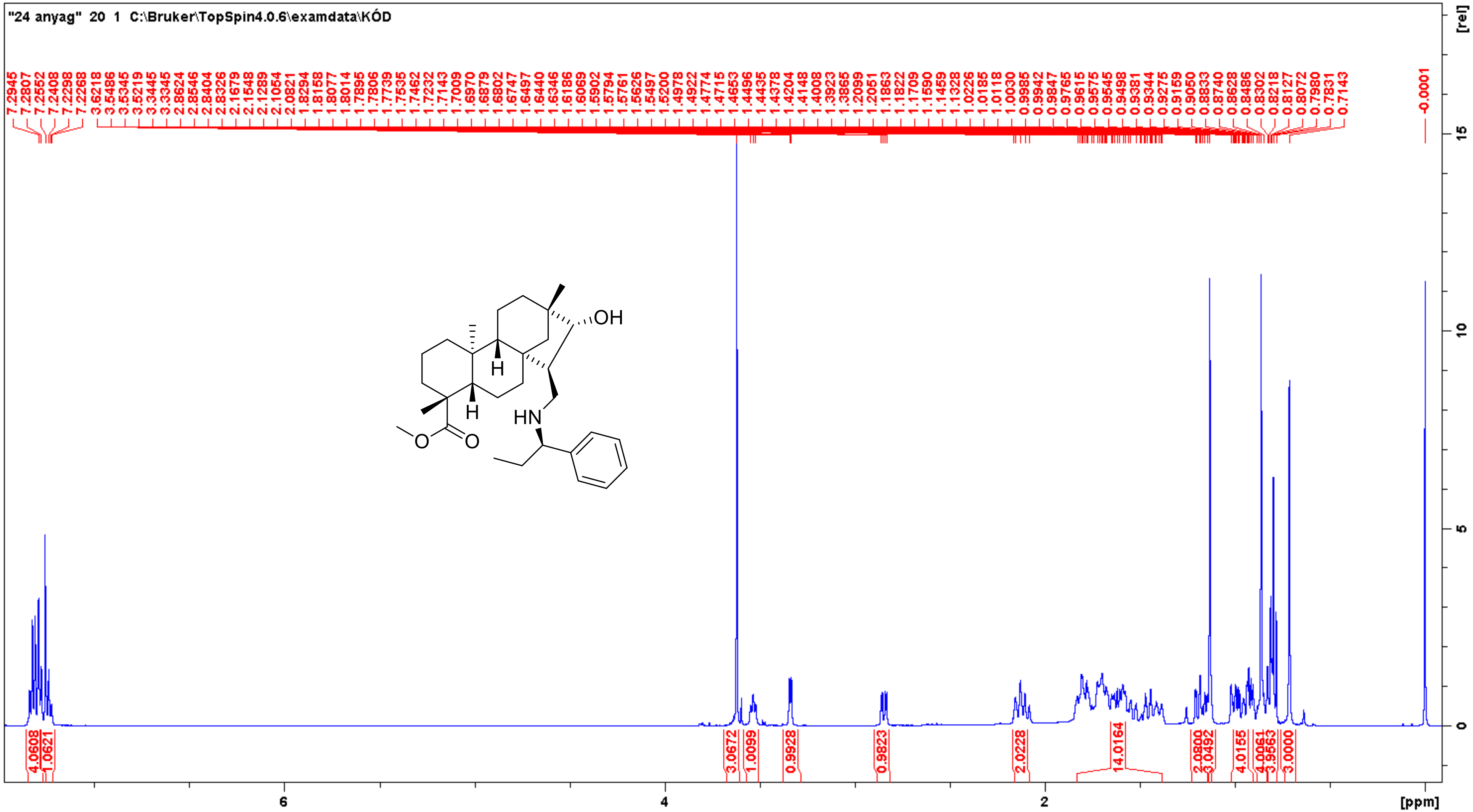

Figure S117

^{13}C-NMR of compound (4*R*,4a*S*,6a*S*,7*R*,8*R*,9*S*,11b*S*)-Methyl 8-hydroxy-4,9,11b-trimethyl-7-((((*S*)-1-phenylpropyl)amino)methyl)tetradecahydro-6a,9-methanocyclohepta[a]naphthalene-4-carboxylate **(25)**:

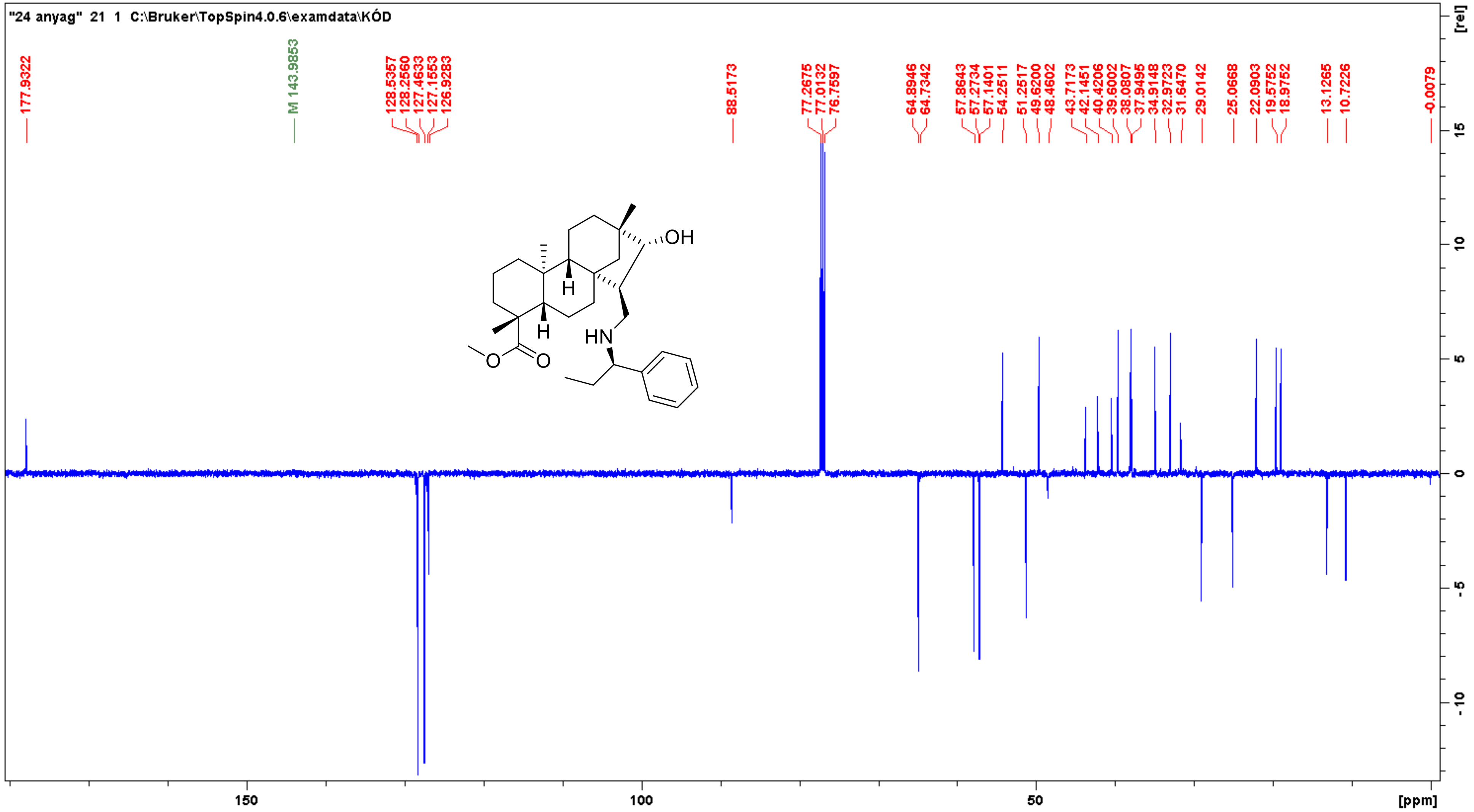

Figure S118

COSY of compound (4*R*,4a*S*,6a*S*,7*R*,8*R*,9*S*,11b*S*)-Methyl 8-hydroxy-4,9,11b-trimethyl-7-((((*S*)-1-phenylpropyl)amino)methyl)tetradecahydro-6a,9-methanocyclohepta[a]naphthalene-4-carboxylate **(25)**:

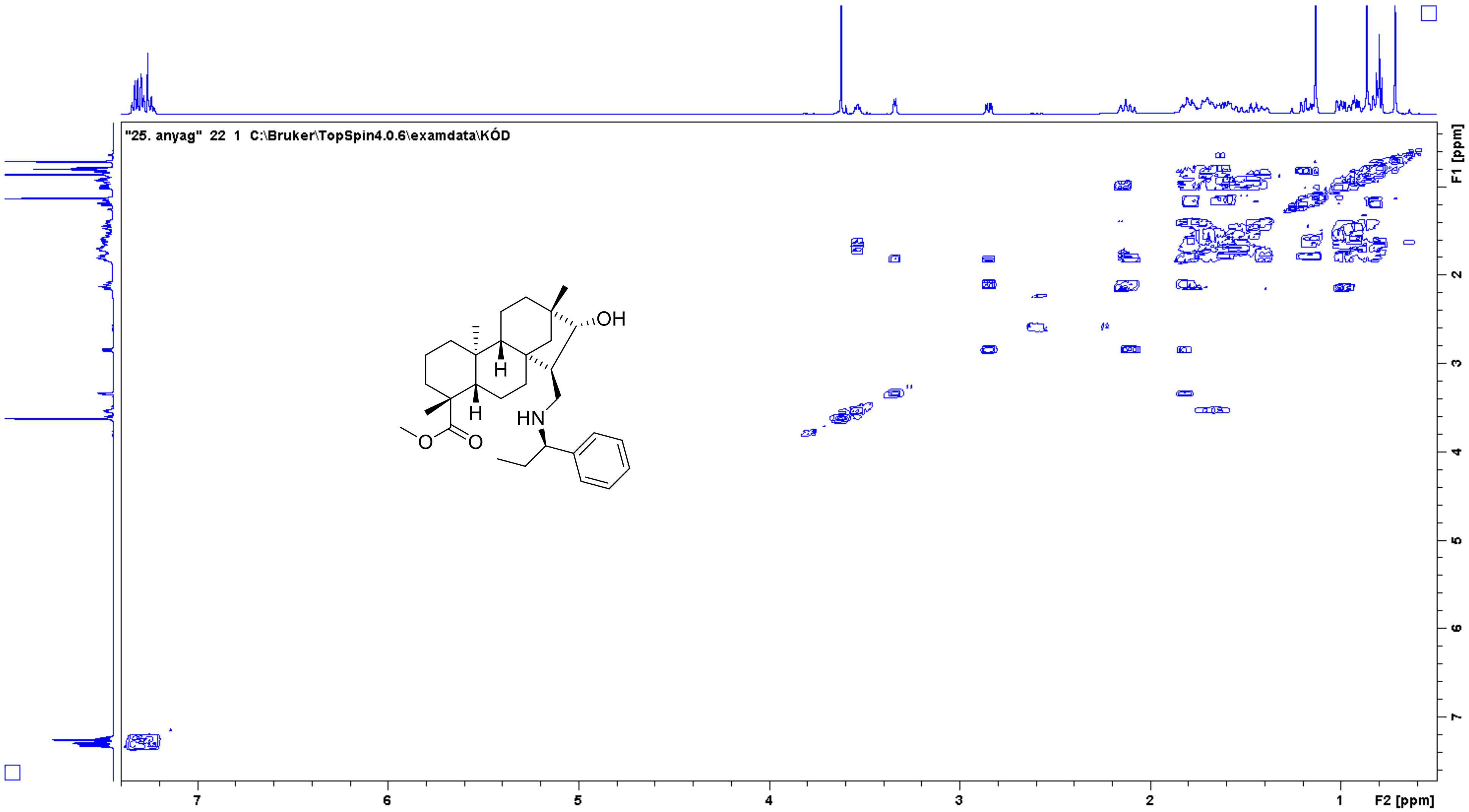

Figure S119

NOESY of compound (4*R*,4a*S*,6a*S*,7*R*,8*R*,9*S*,11b*S*)-Methyl 8-hydroxy-4,9,11b-trimethyl-7-((((*S*)-1-phenylpropyl)amino)methyl)tetradecahydro-6a,9-methanocyclohepta[a]naphthalene-4-carboxylate **(25)**:

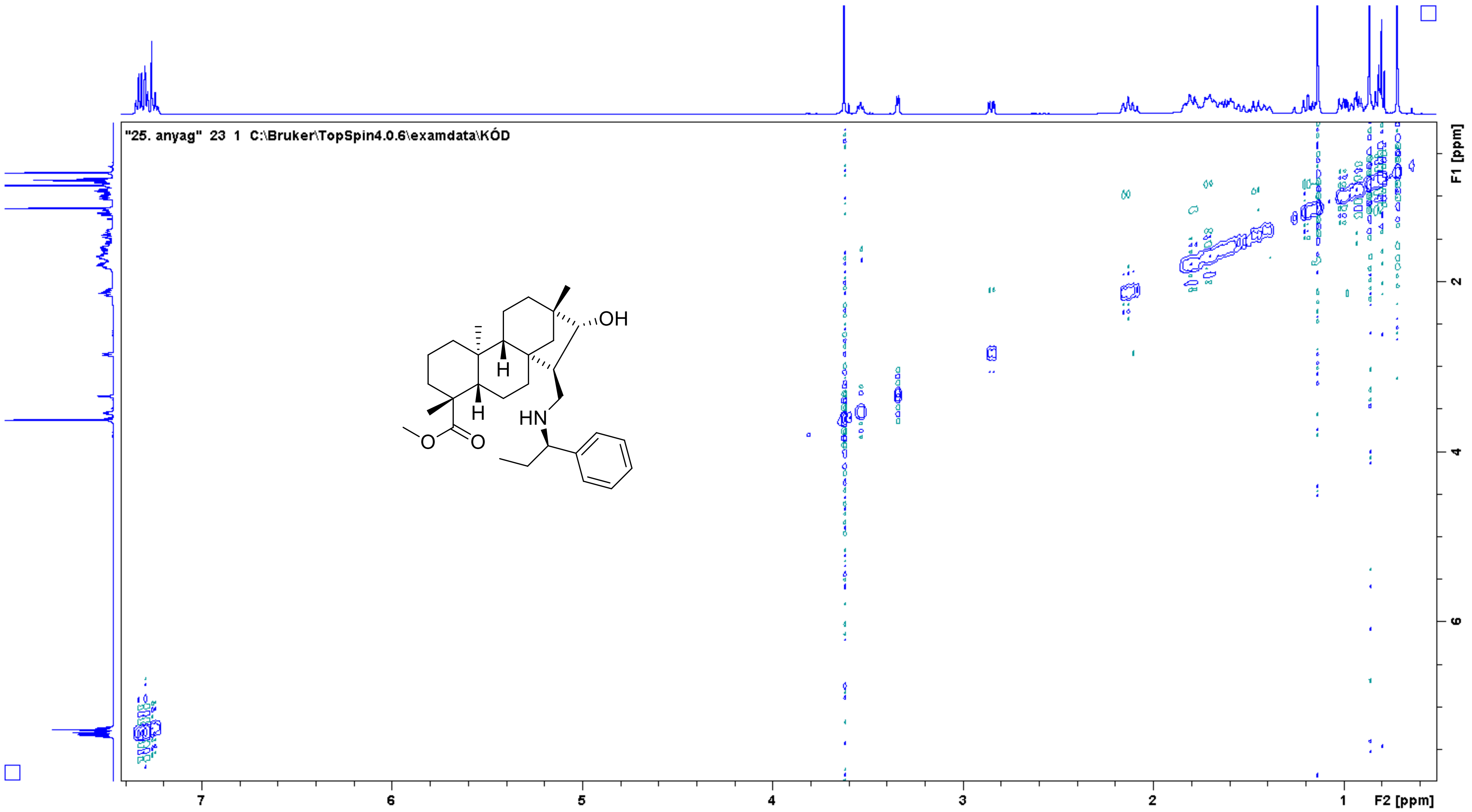

Figure S120

HSQC of compound (4*R*,4a*S*,6a*S*,7*R*,8*R*,9*S*,11b*S*)-Methyl 8-hydroxy-4,9,11b-trimethyl-7-((((*S*)-1-phenylpropyl)amino)methyl)tetradecahydro-6a,9-methanocyclohepta[a]naphthalene-4-carboxylate **(25)**:

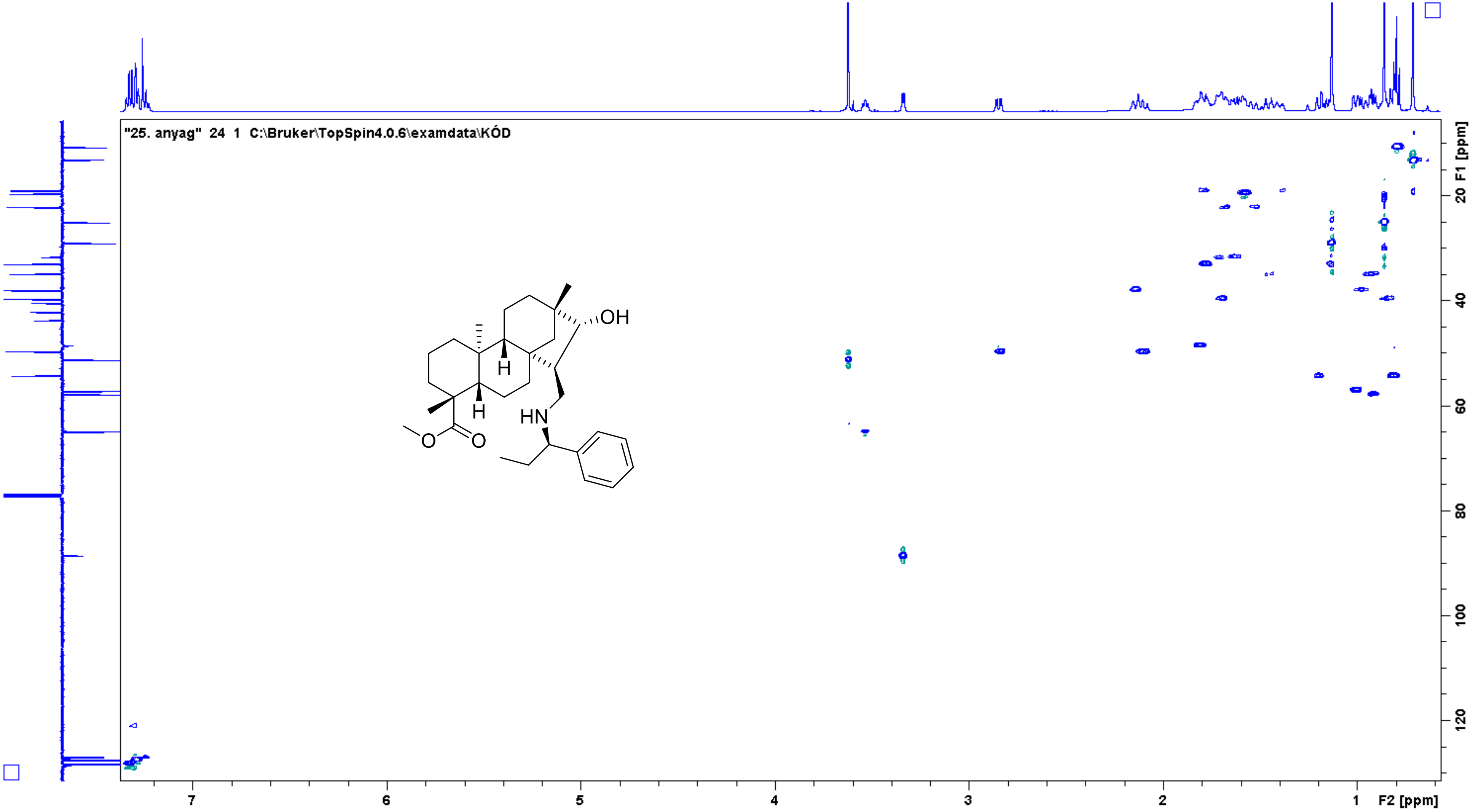

Figure S121

HMBC of compound (4*R*,4a*S*,6a*S*,7*R*,8*R*,9*S*,11b*S*)-Methyl 8-hydroxy-4,9,11b-trimethyl-7-((((*S*)-1-phenylpropyl)amino)methyl)tetradecahydro-6a,9-methanocyclohepta[a]naphthalene-4-carboxylate **(25)**:

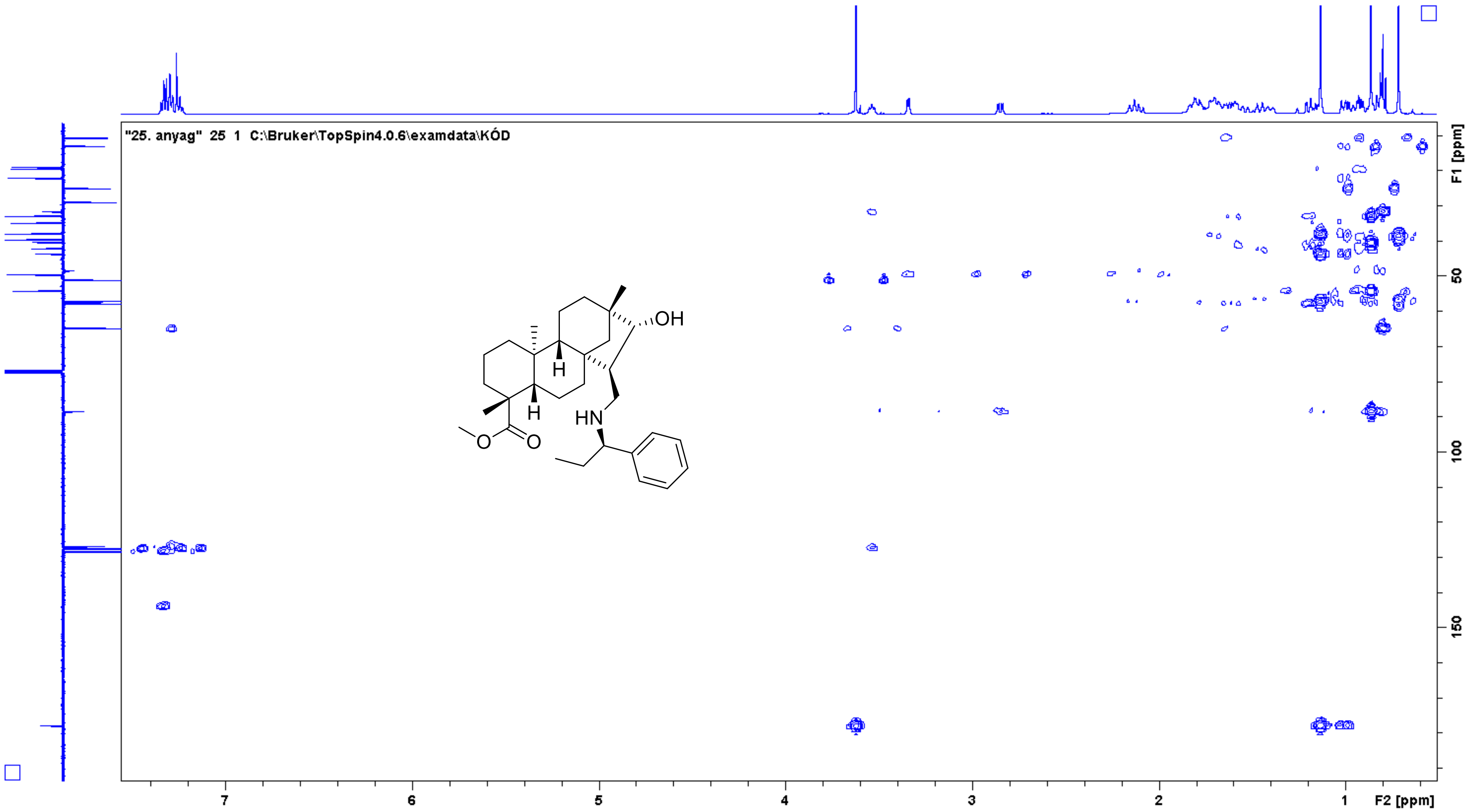

Figure S122

^{1}H-NMR of compound (4*R*,4a*S*,6a*S*,7*R*,8*R*,9*S*,11b*S*)-Methyl 8-hydroxy-4,9,11b-trimethyl-7-((((*S*)-1-(naphthalen-2-yl)ethyl)amino)methyl)tetradecahydro-6a,9-methanocyclohepta[a]naphthalene-4-carboxylate **(26)**:

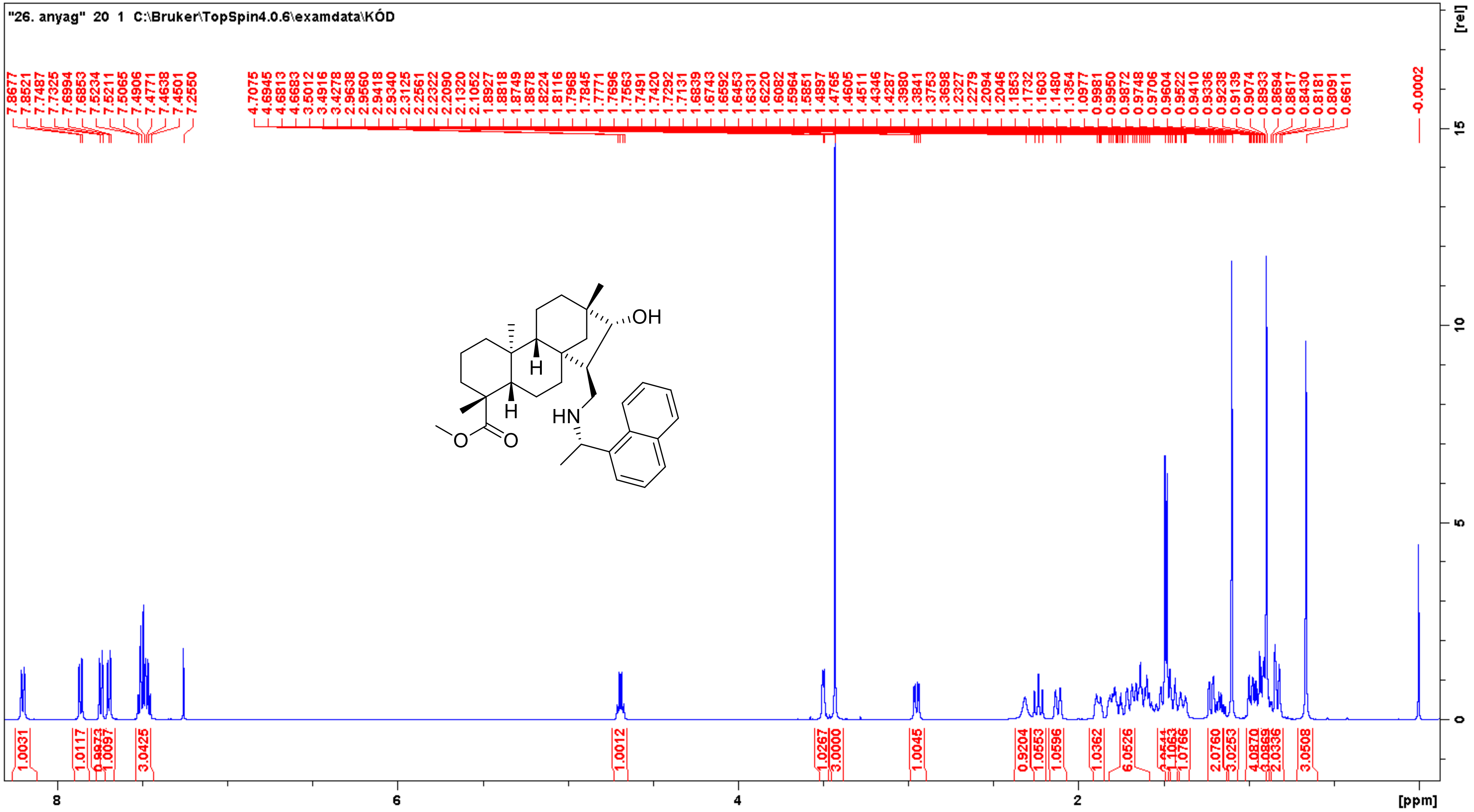

Figure S123

^{13}C-NMR of compound (4*R*,4a*S*,6a*S*,7*R*,8*R*,9*S*,11b*S*)-Methyl 8-hydroxy-4,9,11b-trimethyl-7-((((*S*)-1-(naphthalen-2-yl)ethyl)amino)methyl)tetradecahydro-6a,9-methanocyclohepta[a]naphthalene-4-carboxylate **(26)**:

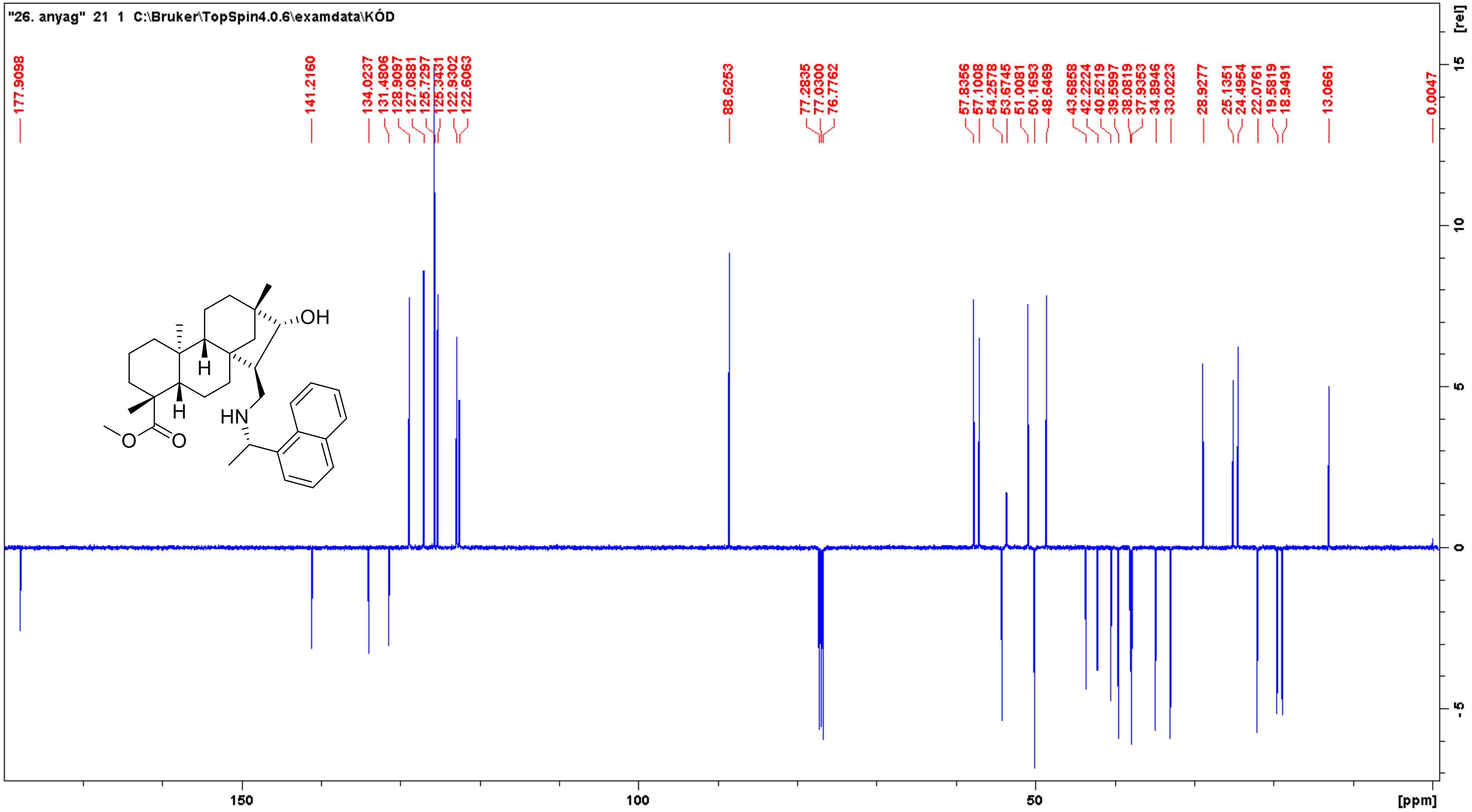

Figure S124

COSY of compound (4*R*,4a*S*,6a*S*,7*R*,8*R*,9*S*,11b*S*)-Methyl 8-hydroxy-4,9,11b-trimethyl-7-((((*S*)-1-(naphthalen-2-yl)ethyl)amino)methyl)tetradecahydro-6a,9-methanocyclohepta[a]naphthalene-4-carboxylate **(26)**:

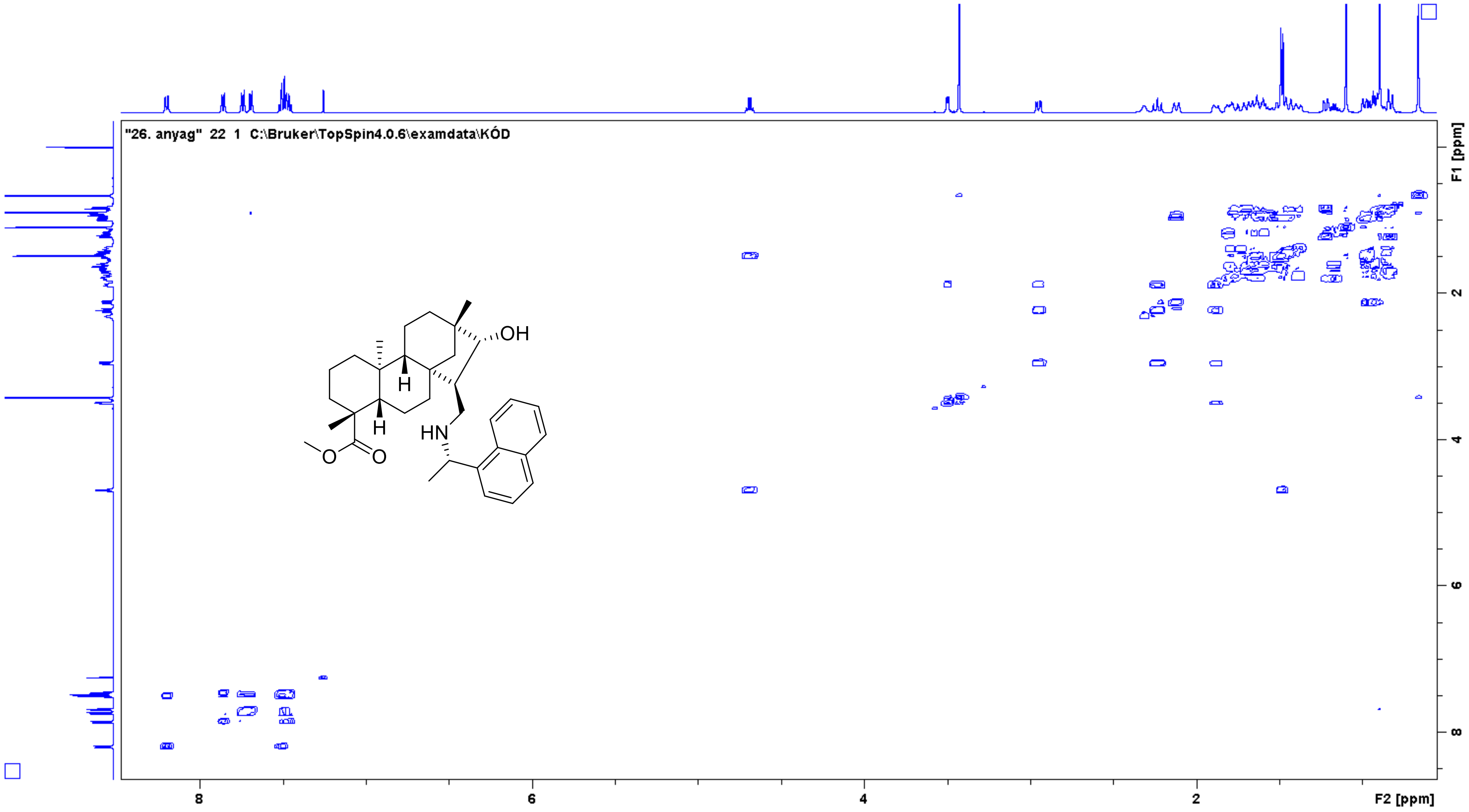

Figure S125

NOESY of compound (4*R*,4a*S*,6a*S*,7*R*,8*R*,9*S*,11b*S*)-Methyl 8-hydroxy-4,9,11b-trimethyl-7-((((*S*)-1-(naphthalen-2-yl)ethyl)amino)methyl)tetradecahydro-6a,9-methanocyclohepta[a]naphthalene-4-carboxylate **(26)**:

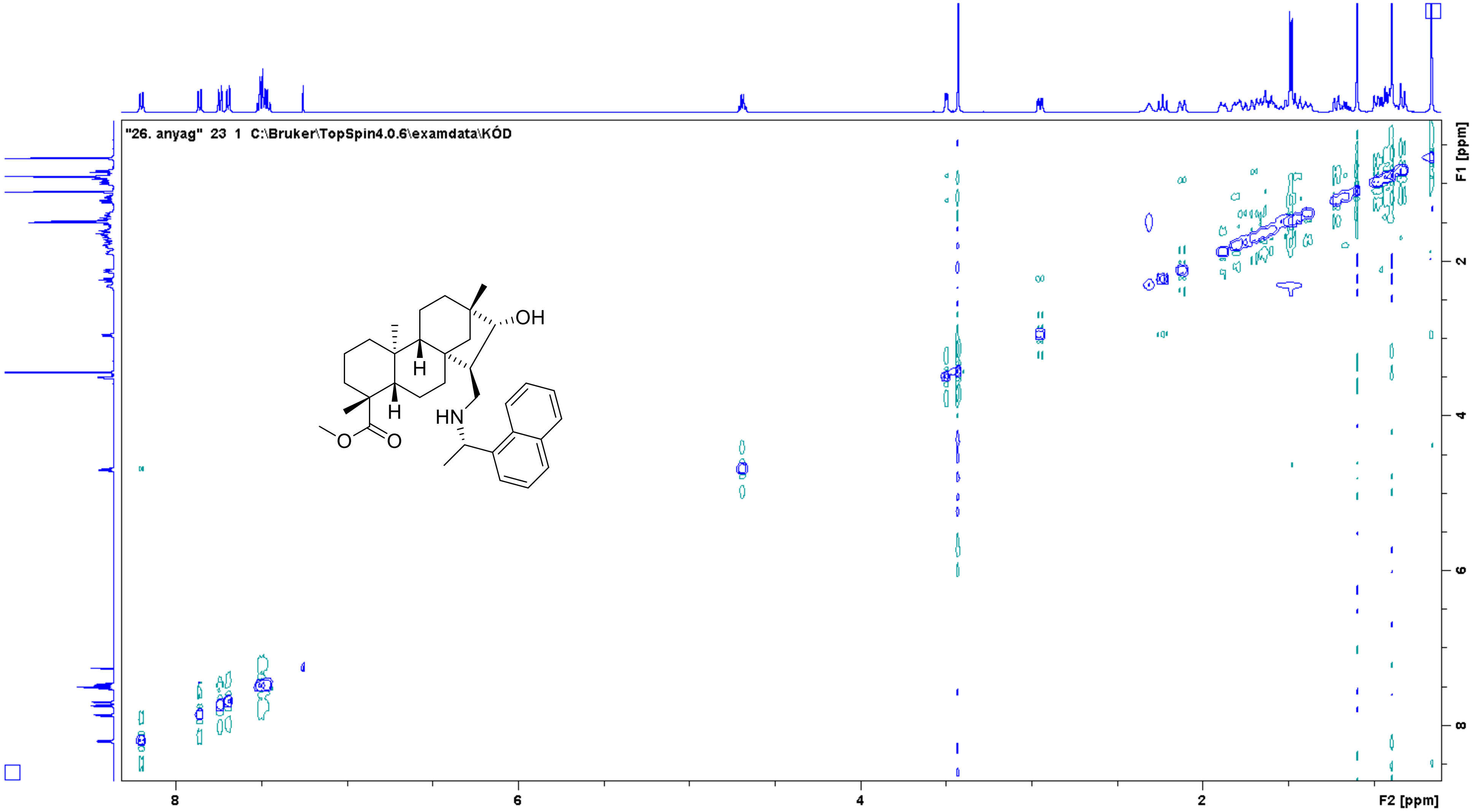

Figure S126

HSQC of compound (4*R*,4a*S*,6a*S*,7*R*,8*R*,9*S*,11b*S*)-Methyl 8-hydroxy-4,9,11b-trimethyl-7-((((*S*)-1-(naphthalen-2-yl)ethyl)amino)methyl)tetradecahydro-6a,9-methanocyclohepta[a]naphthalene-4-carboxylate **(26)**:

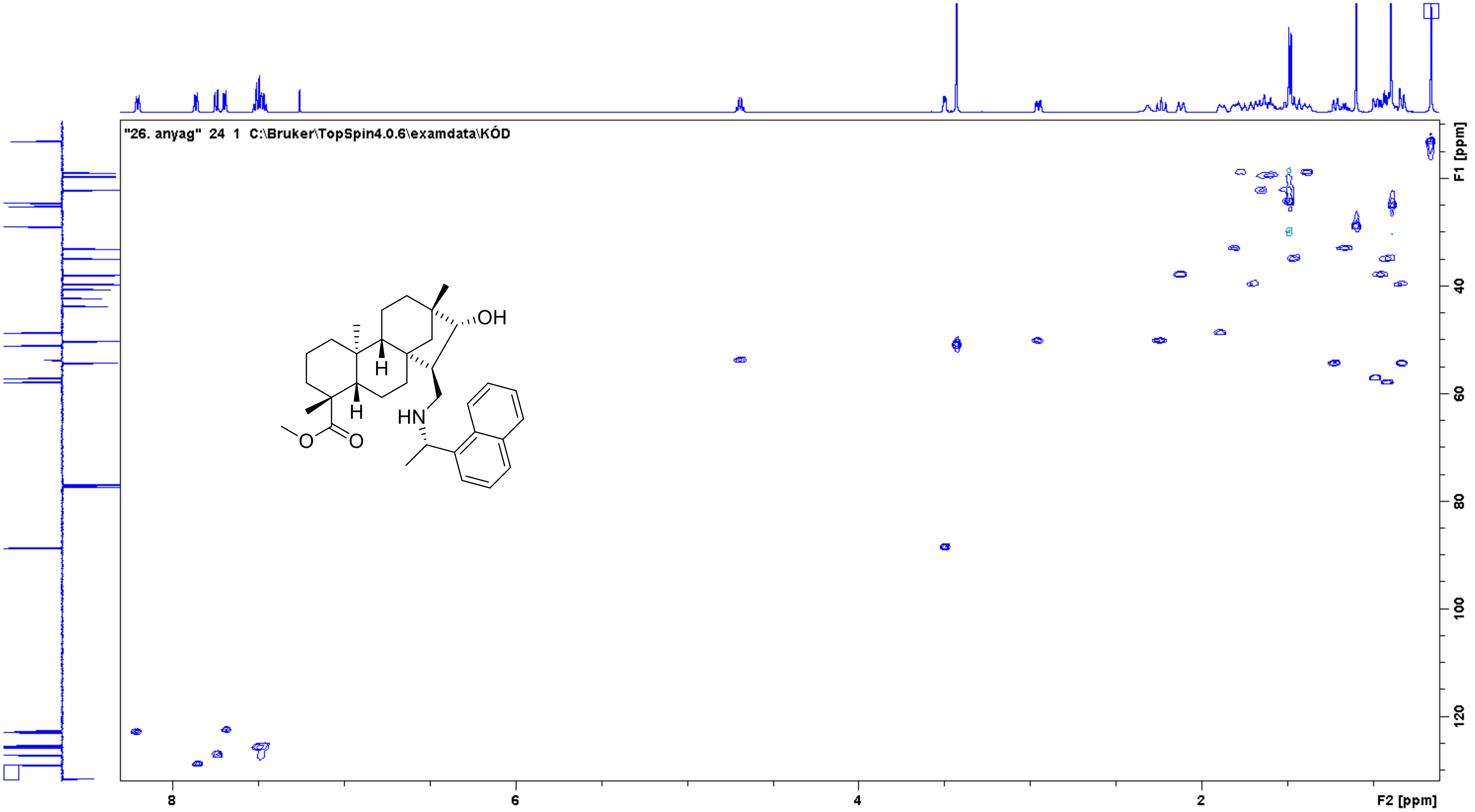

Figure S127

HMBC of compound (4*R*,4a*S*,6a*S*,7*R*,8*R*,9*S*,11b*S*)-Methyl 8-hydroxy-4,9,11b-trimethyl-7-((((*S*)-1-(naphthalen-2-yl)ethyl)amino)methyl)tetradecahydro-6a,9-methanocyclohepta[a]naphthalene-4-carboxylate **(26)**:

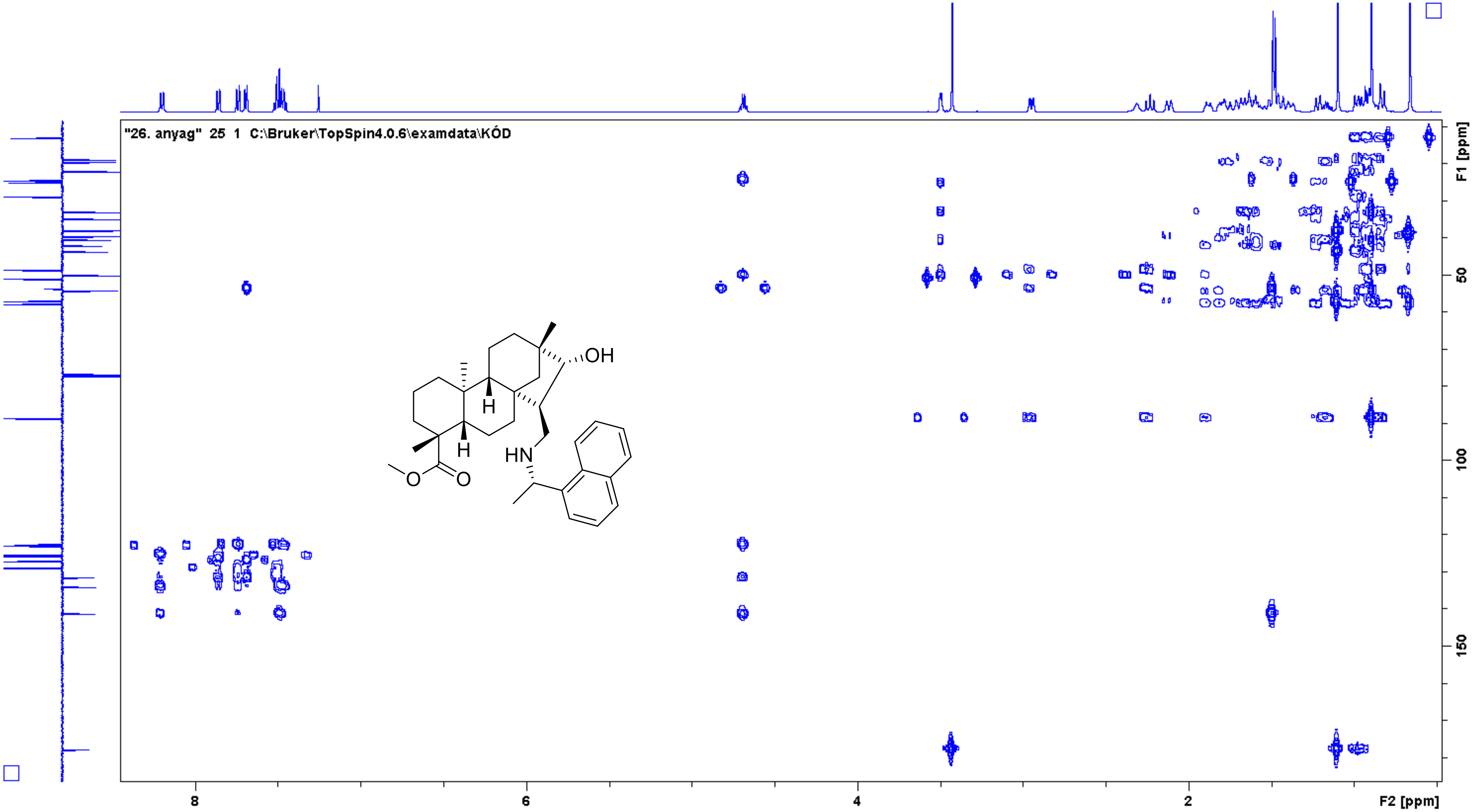

Figure S128

^{1}H-NMR of compound (4*R*,4a*S*,6a*S*,7*R*,8*R*,9*S*,11b*S*)-Methyl 8-hydroxy-4,9,11b-trimethyl-7-((((*R*)-1-(naphthalen-2-yl)ethyl)amino)methyl)tetradecahydro-6a,9-methanocyclohepta[a]naphthalene-4-carboxylate **(27)**:

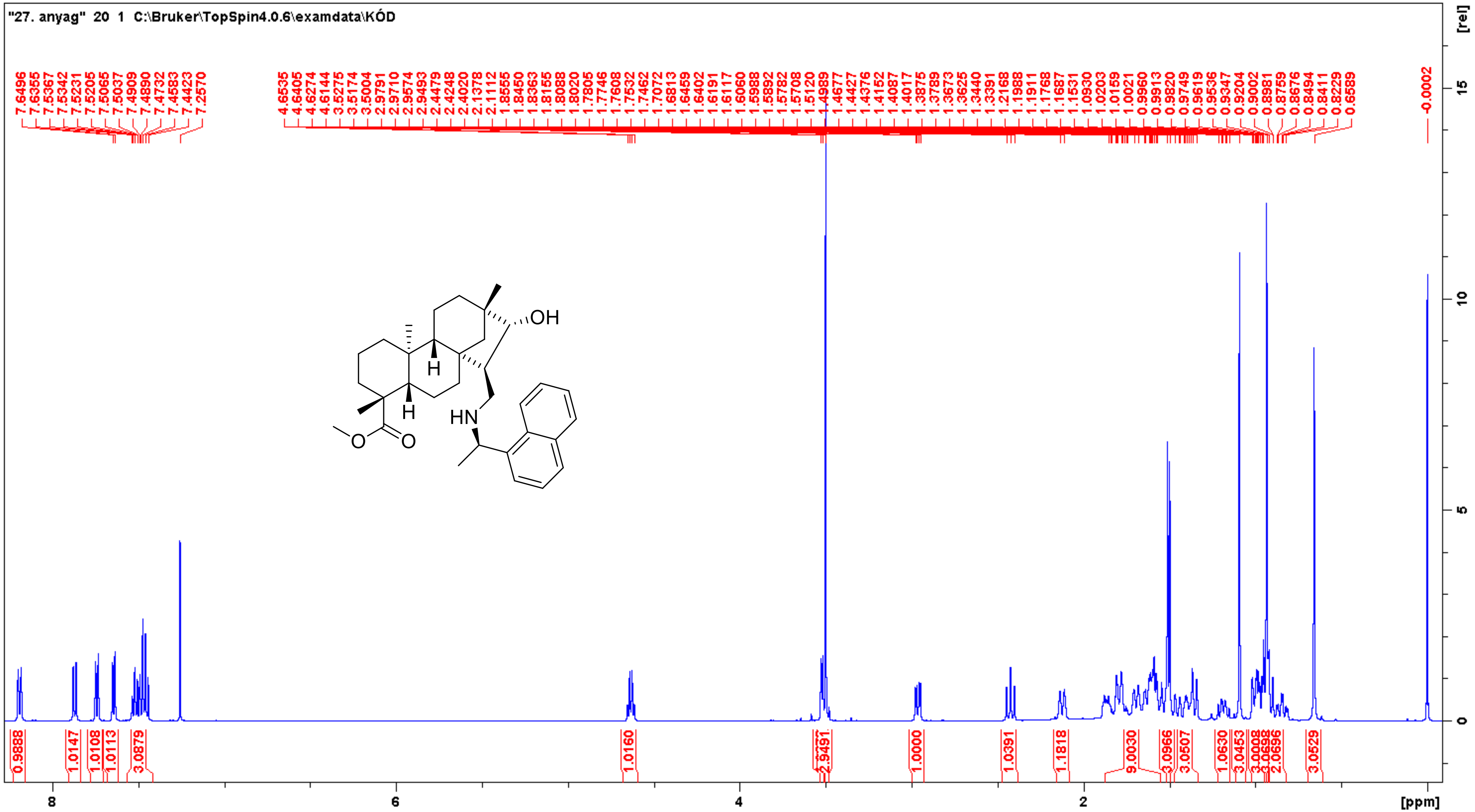

Figure S129

^{13}C-NMR of compound (4*R*,4a*S*,6a*S*,7*R*,8*R*,9*S*,11b*S*)-Methyl 8-hydroxy-4,9,11b-trimethyl-7-((((*R*)-1-(naphthalen-2-yl)ethyl)amino)methyl)tetradecahydro-6a,9-methanocyclohepta[a]naphthalene-4-carboxylate **(27)**:

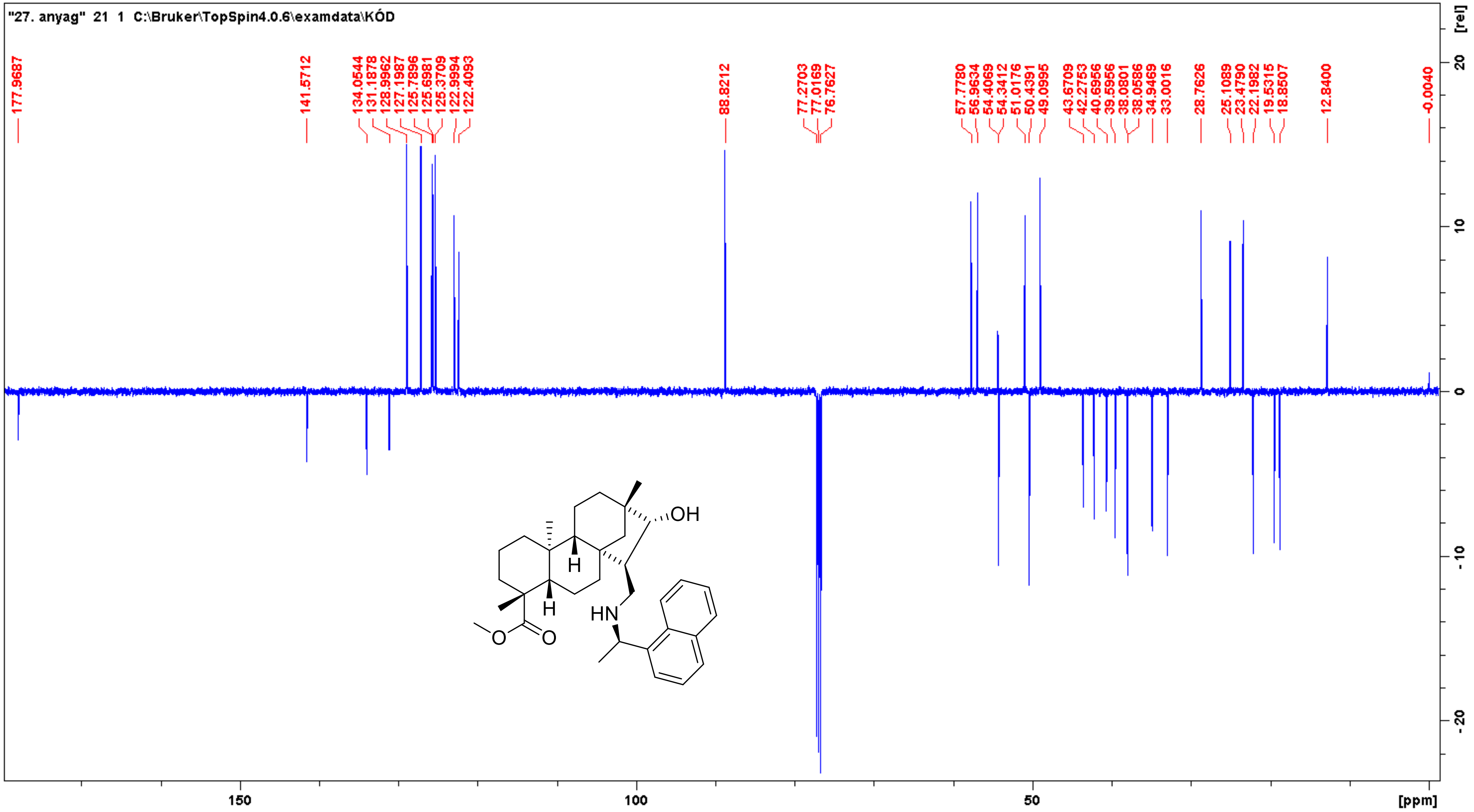

Figure S130

COSY of compound (4*R*,4a*S*,6a*S*,7*R*,8*R*,9*S*,11b*S*)-Methyl 8-hydroxy-4,9,11b-trimethyl-7-((((*R*)-1-(naphthalen-2-yl)ethyl)amino)methyl)tetradecahydro-6a,9-methanocyclohepta[a]naphthalene-4-carboxylate **(27)**:

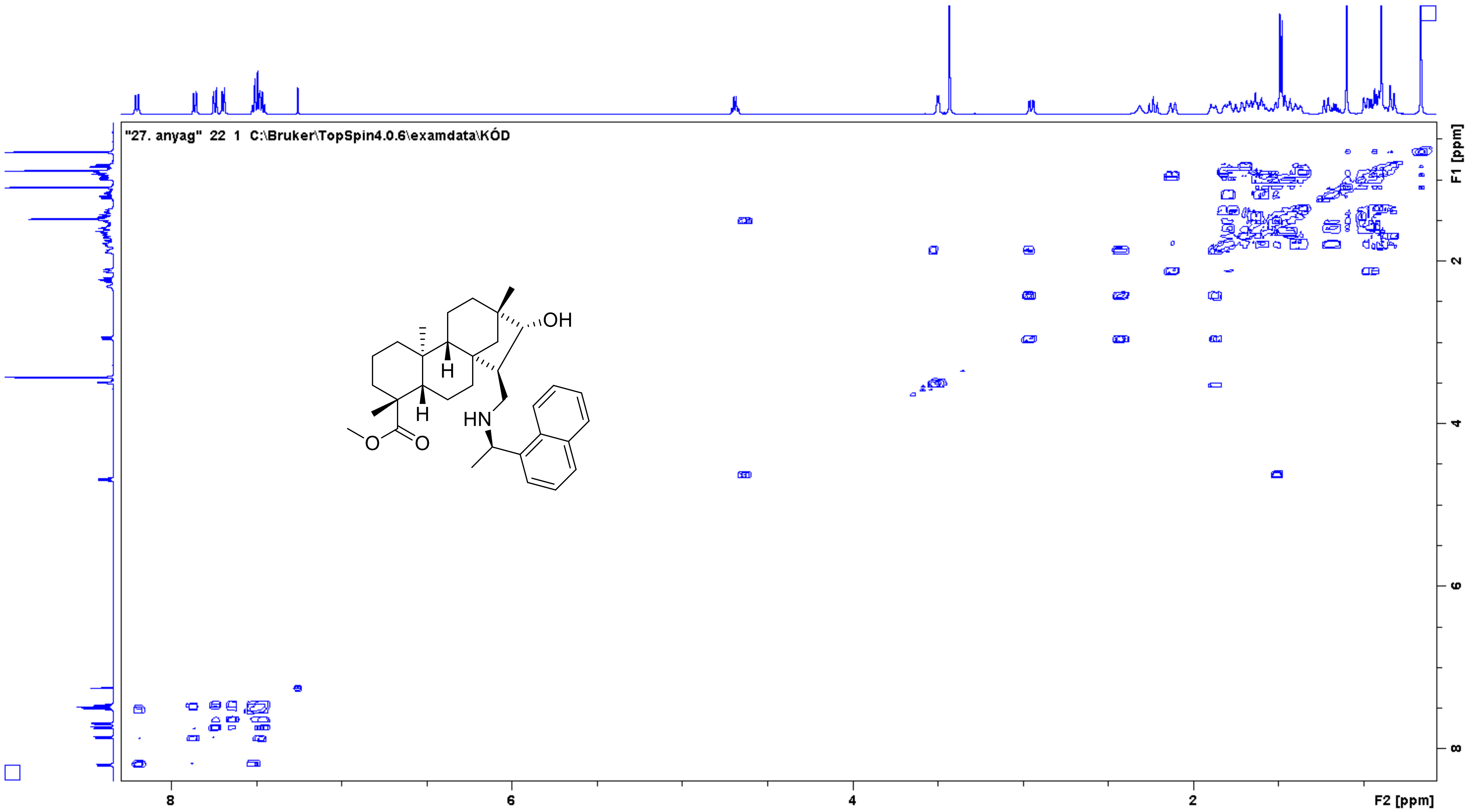

Figure S131

NOESY of compound (4*R*,4a*S*,6a*S*,7*R*,8*R*,9*S*,11b*S*)-Methyl 8-hydroxy-4,9,11b-trimethyl-7-((((*R*)-1-(naphthalen-2-yl)ethyl)amino)methyl)tetradecahydro-6a,9-methanocyclohepta[a]naphthalene-4-carboxylate **(27)**:

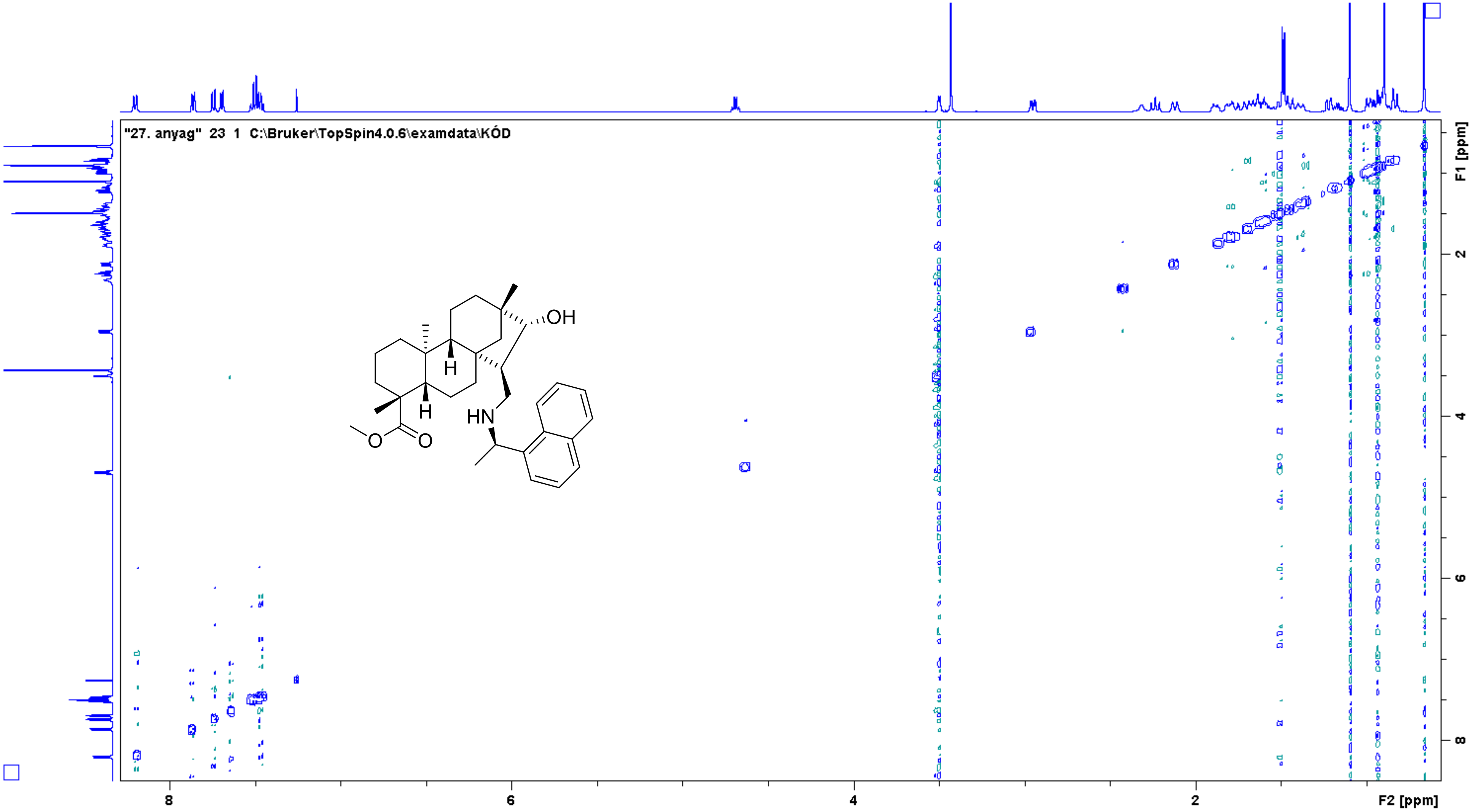

Figure S132

HSQC of compound (4*R*,4a*S*,6a*S*,7*R*,8*R*,9*S*,11b*S*)-Methyl 8-hydroxy-4,9,11b-trimethyl-7-((((*R*)-1-(naphthalen-2-yl)ethyl)amino)methyl)tetradecahydro-6a,9-methanocyclohepta[a]naphthalene-4-carboxylate **(27)**:

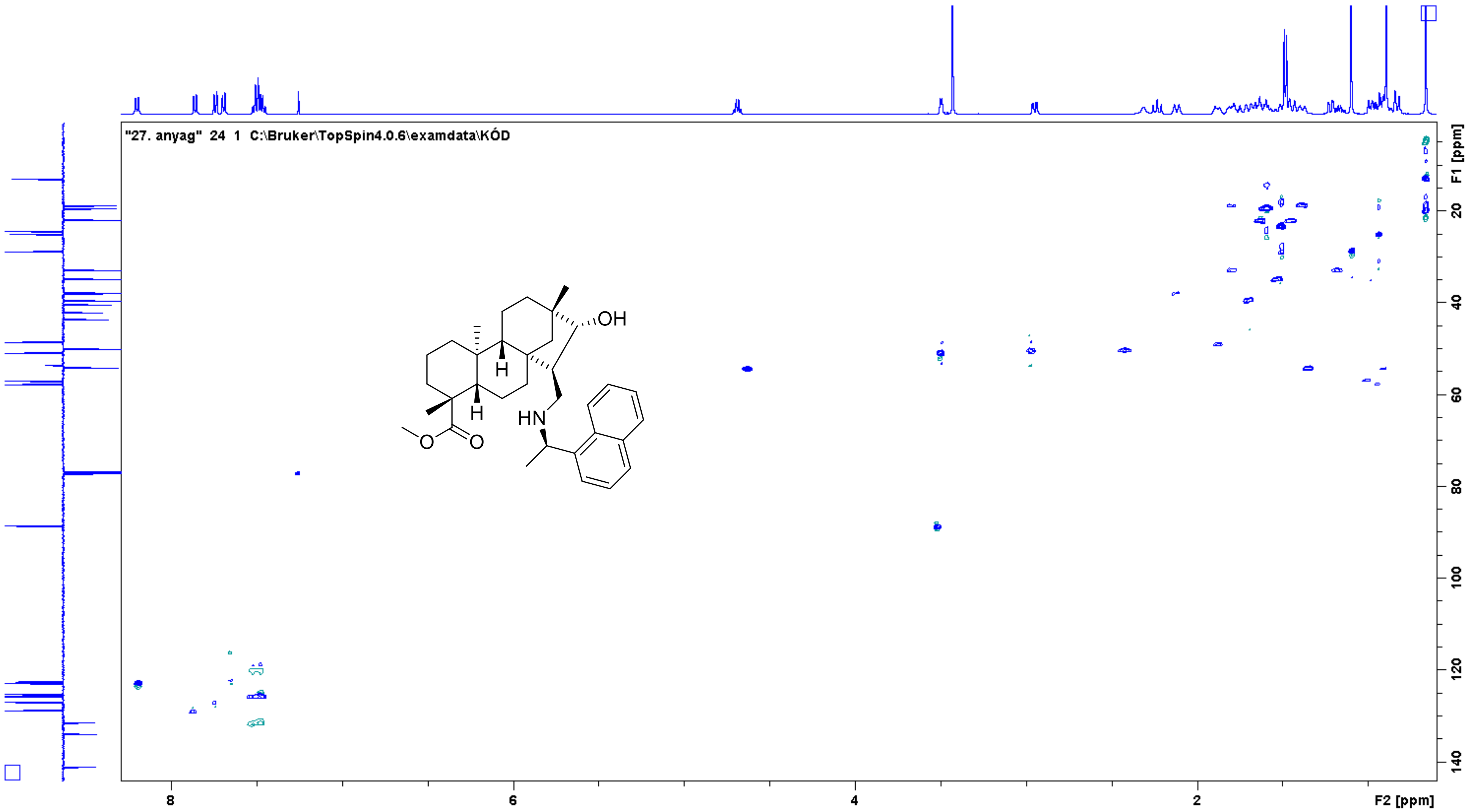

Figure S133

HMBC of compound (4*R*,4a*S*,6a*S*,7*R*,8*R*,9*S*,11b*S*)-Methyl 8-hydroxy-4,9,11b-trimethyl-7-((((*R*)-1-(naphthalen-2-yl)ethyl)amino)methyl)tetradecahydro-6a,9-methanocyclohepta[a]naphthalene-4-carboxylate **(27)**:

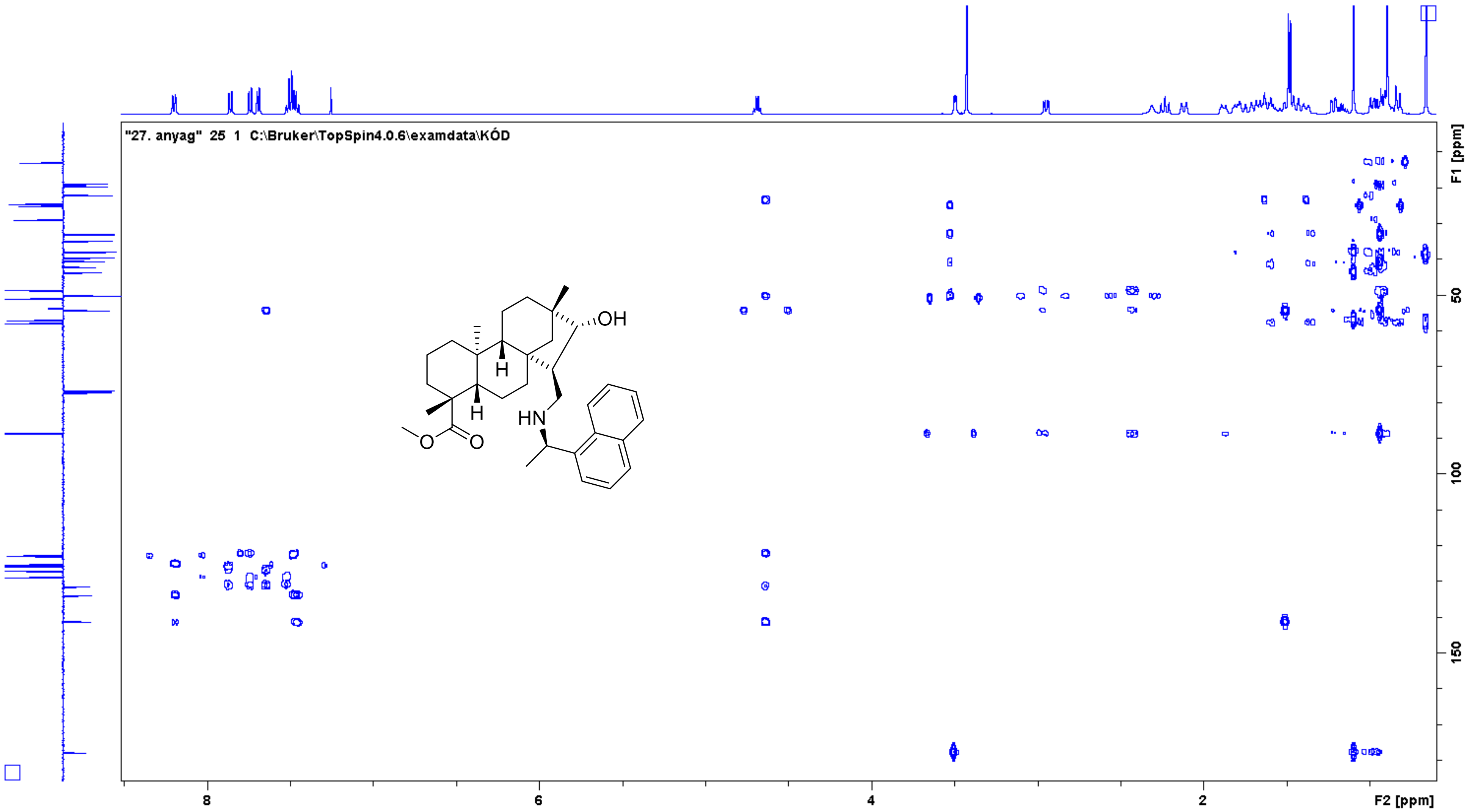

Figure S134

^{1}H-NMR of compound (4*R*,4a*S*,6a*S*,7*R*,8*R*,9*S*,11b*S*)-Methyl 7-(((3-(1H-imidazol-1-yl)propyl)amino)methyl)-8-hydroxy-4,9,11b-trimethyltetradecahydro-6a,9-methanocyclohepta[a]naphthalene-4-carboxylate **(28)**:

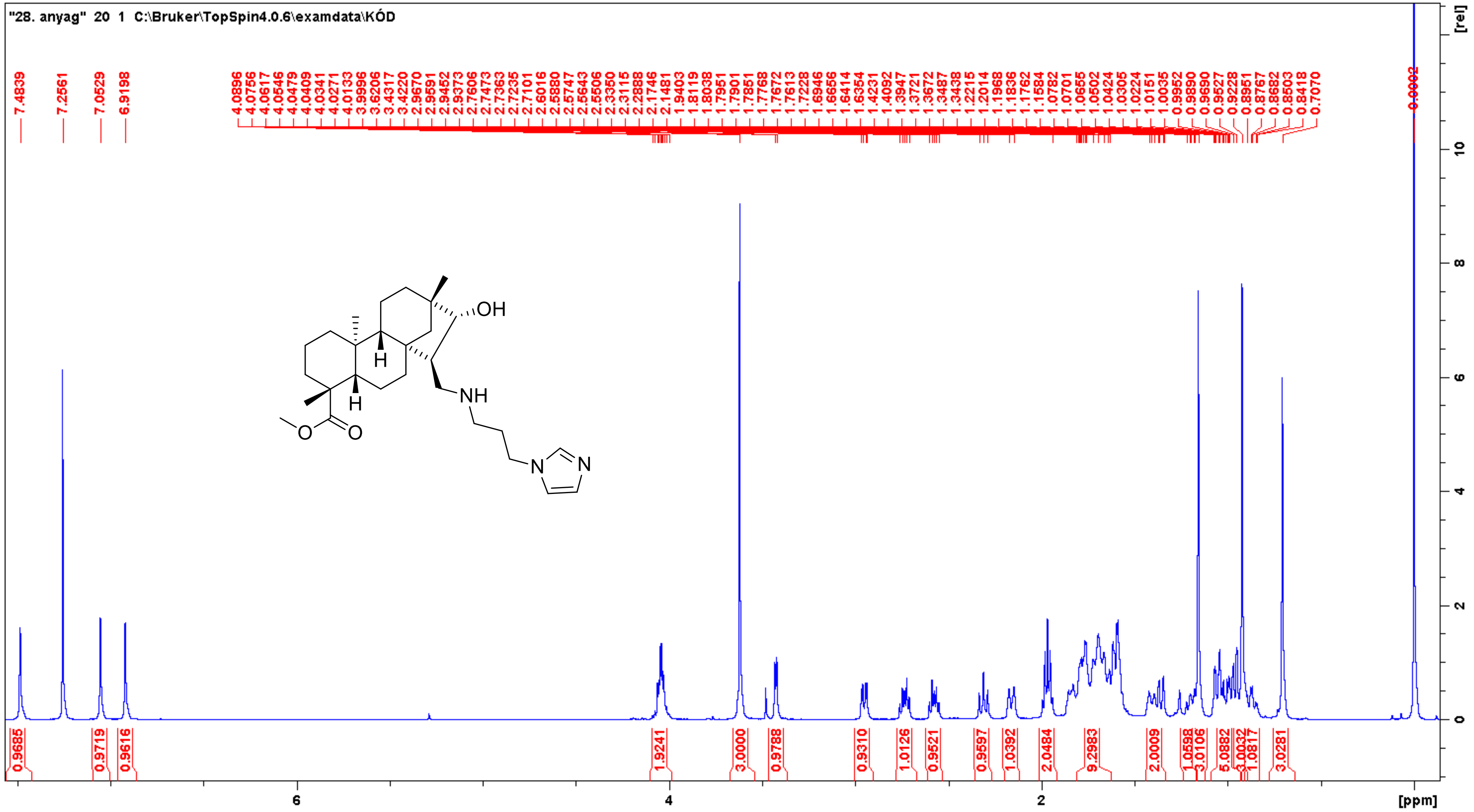

Figure S135

^{13}C-NMR of compound (4*R*,4a*S*,6a*S*,7*R*,8*R*,9*S*,11b*S*)-Methyl 7-(((3-(1H-imidazol-1-yl)propyl)amino)methyl)-8-hydroxy-4,9,11b-trimethyltetradecahydro-6a,9-methanocyclohepta[a]naphthalene-4-carboxylate **(28)**:

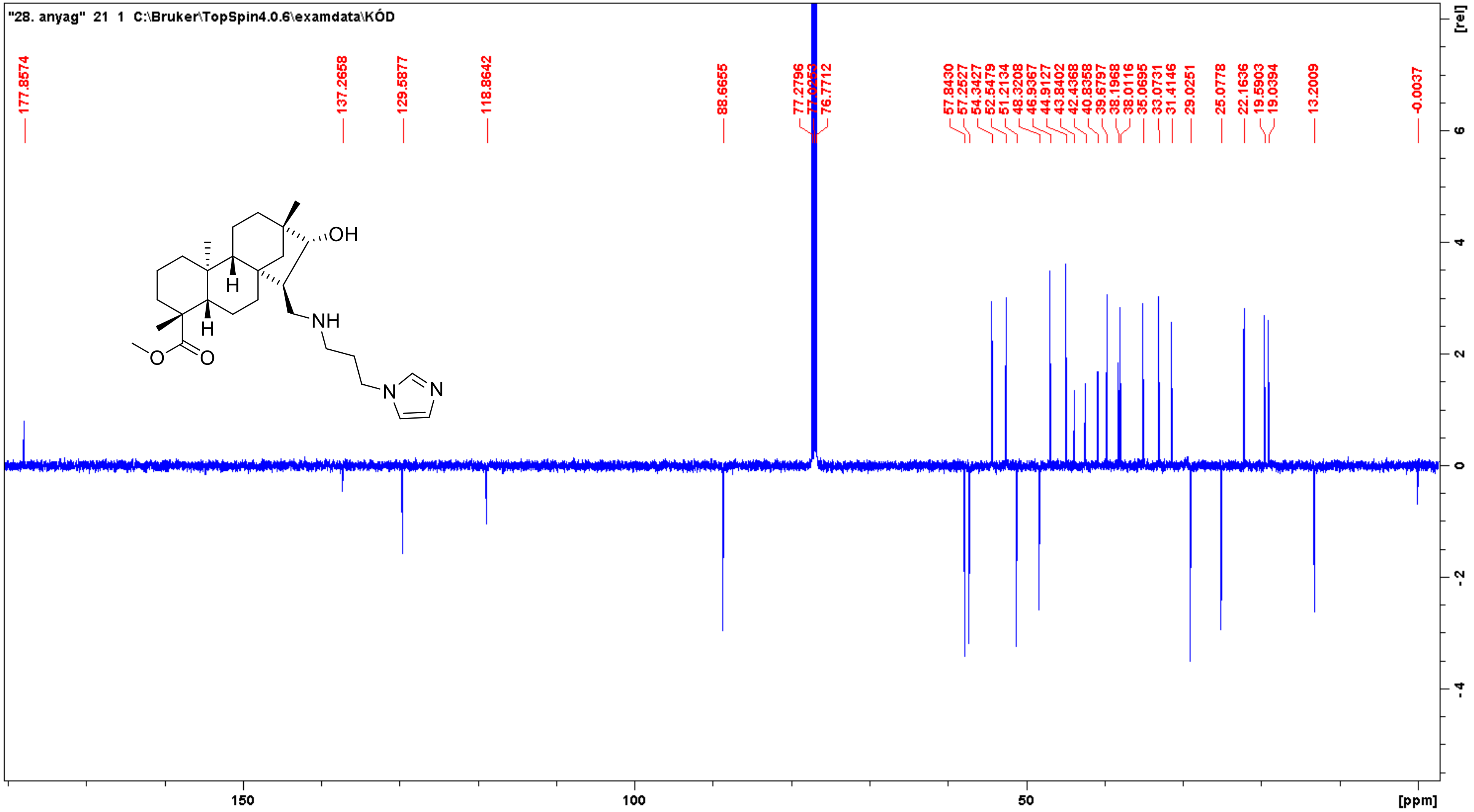

Figure S136

COSY of compound (4*R*,4a*S*,6a*S*,7*R*,8*R*,9*S*,11b*S*)-Methyl 7-(((3-(1H-imidazol-1-yl)propyl)amino)methyl)-8-hydroxy-4,9,11b-trimethyltetradecahydro-6a,9-methanocyclohepta[a]naphthalene-4-carboxylate **(28)**:

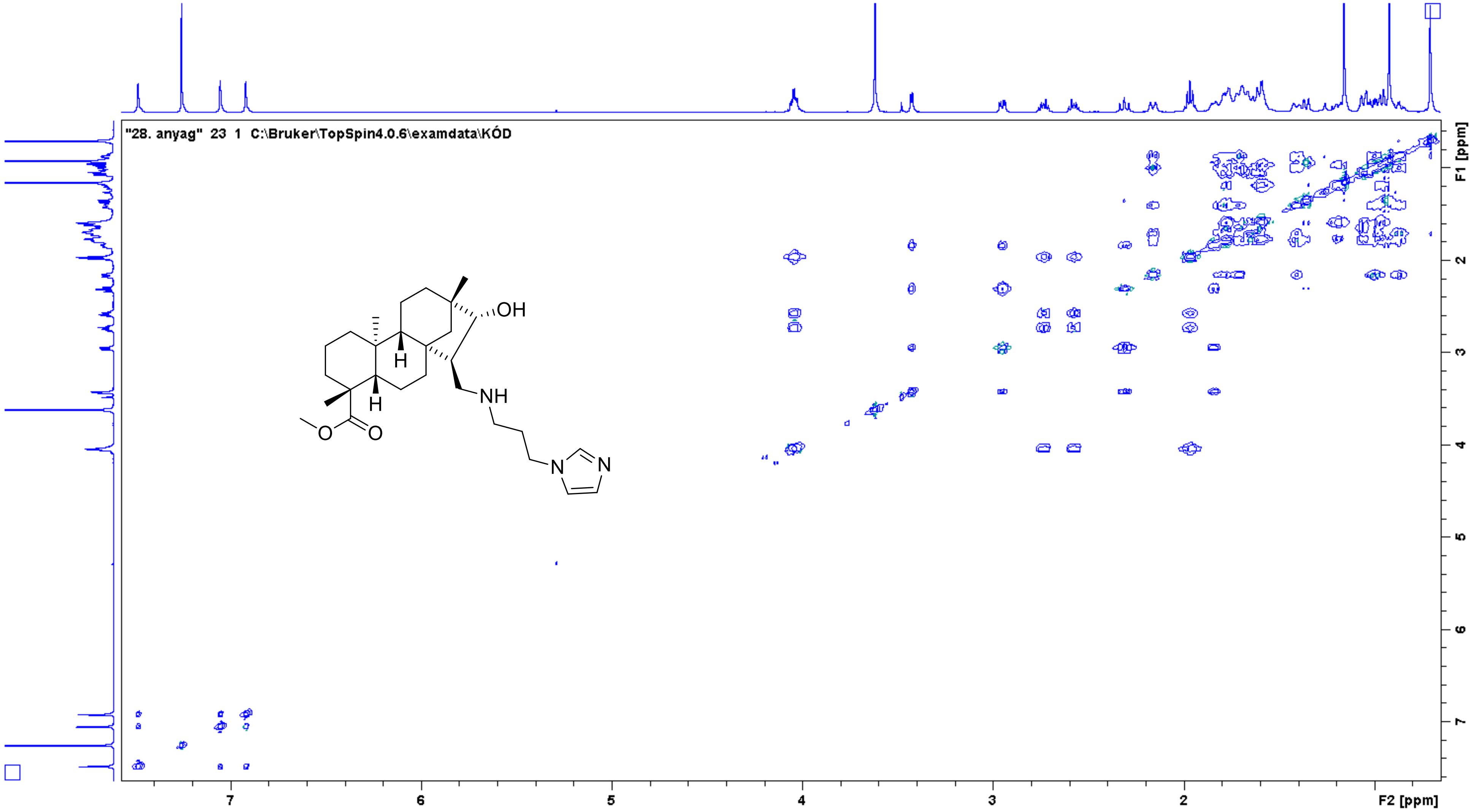

Figure S137

NOESY of compound (4*R*,4a*S*,6a*S*,7*R*,8*R*,9*S*,11b*S*)-Methyl 7-(((3-(1H-imidazol-1-yl)propyl)amino)methyl)-8-hydroxy-4,9,11b-trimethyltetradecahydro-6a,9-methanocyclohepta[a]naphthalene-4-carboxylate **(28)**:

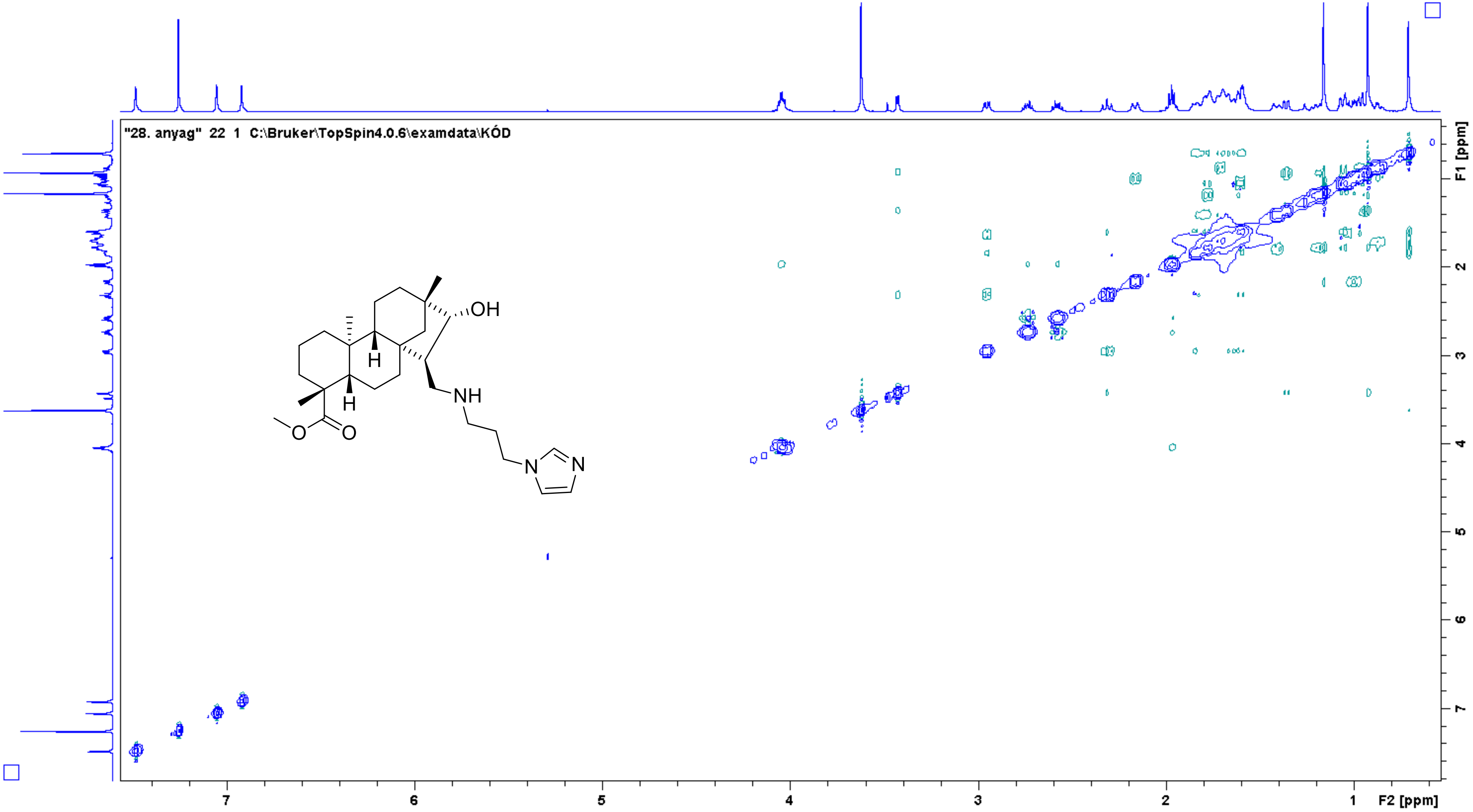

Figure S138

HSQC of compound (4*R*,4a*S*,6a*S*,7*R*,8*R*,9*S*,11b*S*)-Methyl 7-(((3-(1H-imidazol-1-yl)propyl)amino)methyl)-8-hydroxy-4,9,11b-trimethyltetradecahydro-6a,9-methanocyclohepta[a]naphthalene-4-carboxylate **(28)**:

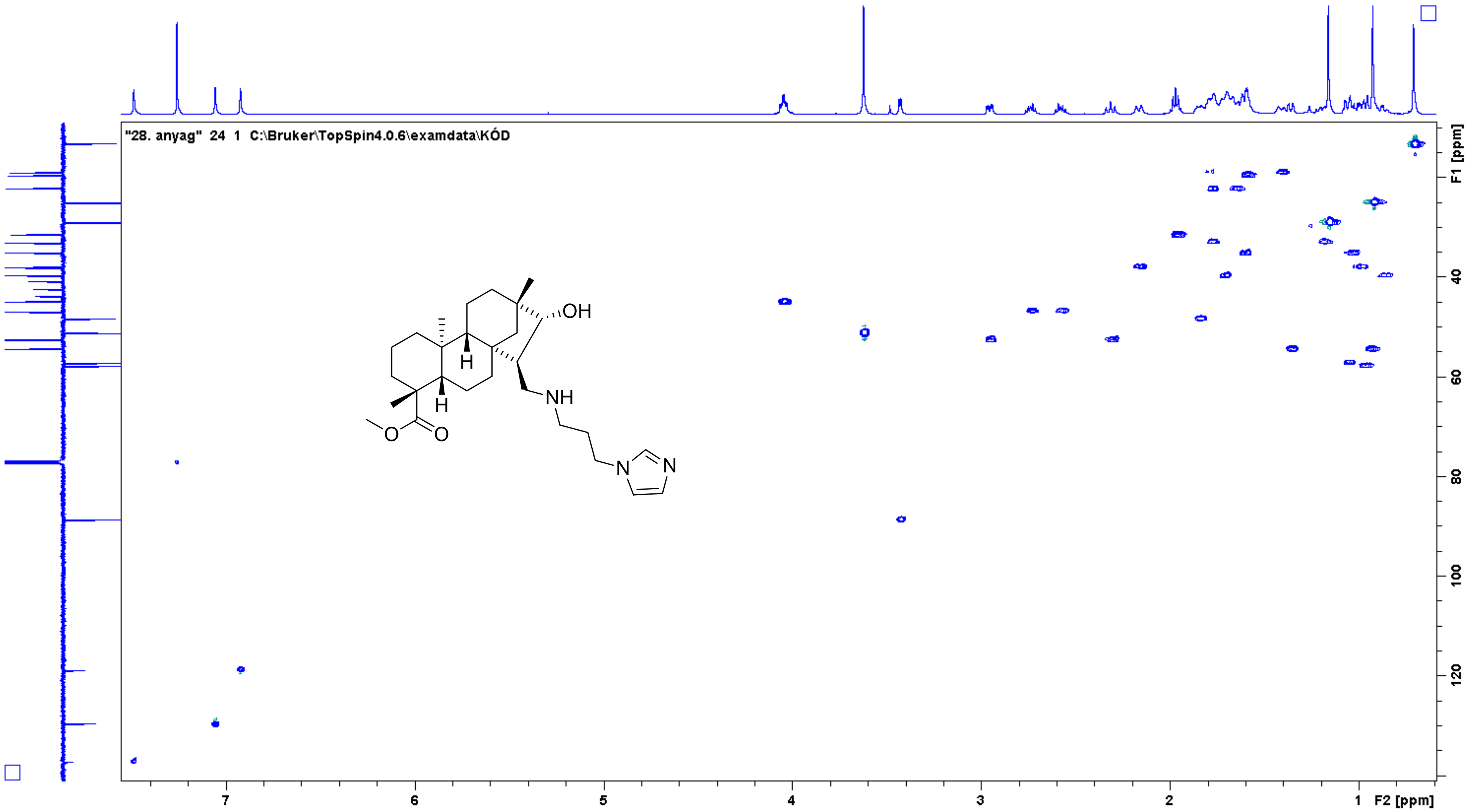

Figure S139

HMBC of compound (4*R*,4a*S*,6a*S*,7*R*,8*R*,9*S*,11b*S*)-Methyl 7-(((3-(1H-imidazol-1-yl)propyl)amino)methyl)-8-hydroxy-4,9,11b-trimethyltetradecahydro-6a,9-methanocyclohepta[a]naphthalene-4-carboxylate **(28)**:

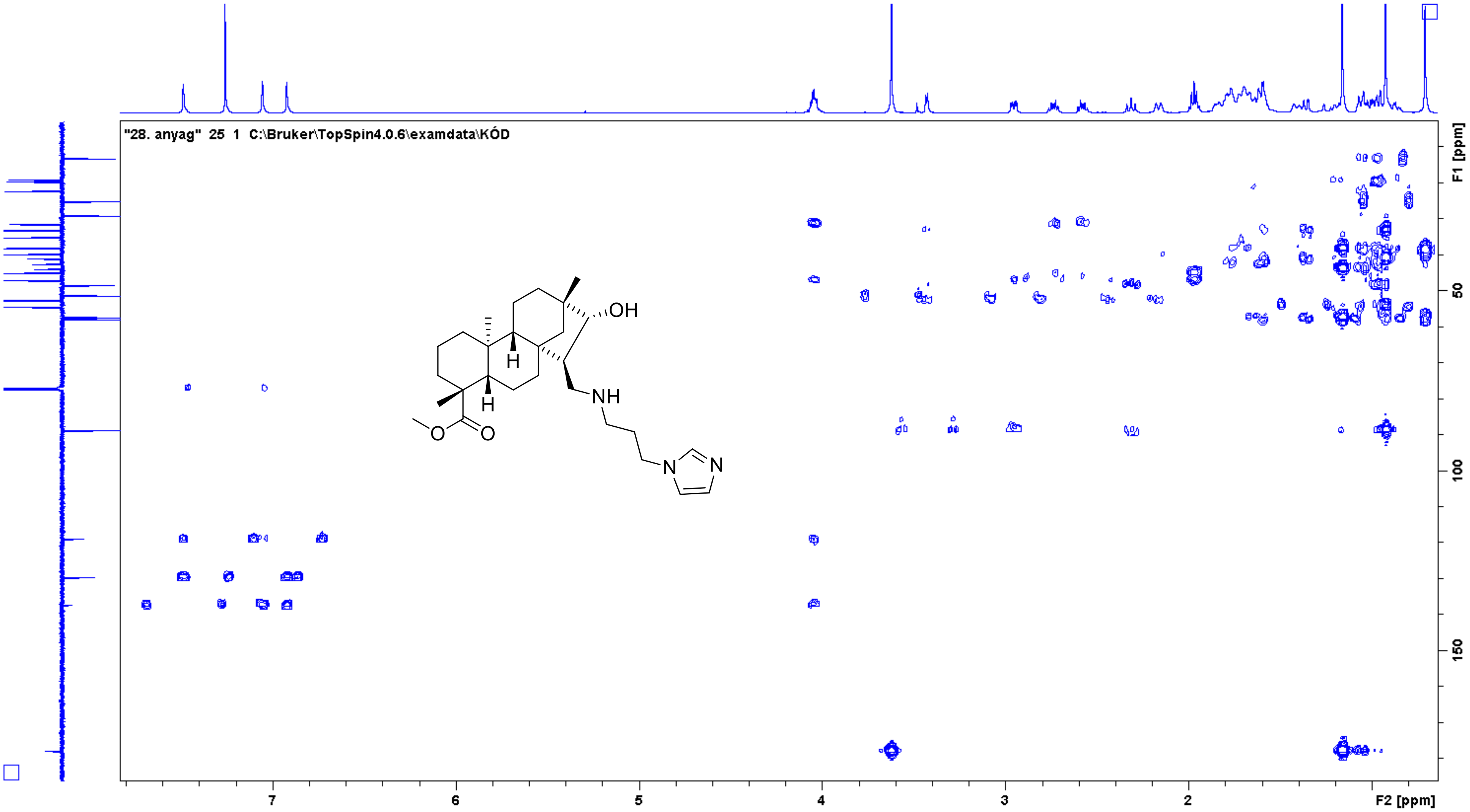

Supplement: Supplementary file 1 [file pharmaceuticals-17-00262-s001.zip › pharmaceuticals-2882746-supplementary.pdf]
